# Fluorination of Aza-BODIPY for Cancer Cell Plasma Membrane-Targeted Imaging and Therapy

**DOI:** 10.1021/acsami.4c17943

**Published:** 2025-01-02

**Authors:** Anfeng Li, Fang Wang, Yu Li, Xingxing Peng, Yanqi Liu, Lijun Zhu, Pei He, Tingting Yu, Daiqin Chen, Mojie Duan, Xin Zhou, Zhong-Xing Jiang, Shizhen Chen

**Affiliations:** † State Key Laboratory of Magnetic Resonance and Atomic and Molecular Physics, National Center for Magnetic Resonance in Wuhan, Wuhan Institute of Physics and Mathematics, Innovation Academy for Precision Measurement Science and Technology, Chinese Academy of Sciences-Wuhan National Laboratory for Optoelectronics, Huazhong University of Science and Technology, Wuhan 430071, China; ‡ University of Chinese Academy of Sciences, Beijing 100049, China; § Interdisciplinary Institute of NMR and Molecular Sciences, School of Chemistry and Chemical Engineering, The State Key Laboratory of Refractories and Metallurgy, Wuhan University of Science and Technology, Wuhan 430081, China

**Keywords:** fluorination, photosensitizer, plasma membrane, ^19^F MRI, fluorescence, photodynamic therapy, pyroptosis

## Abstract

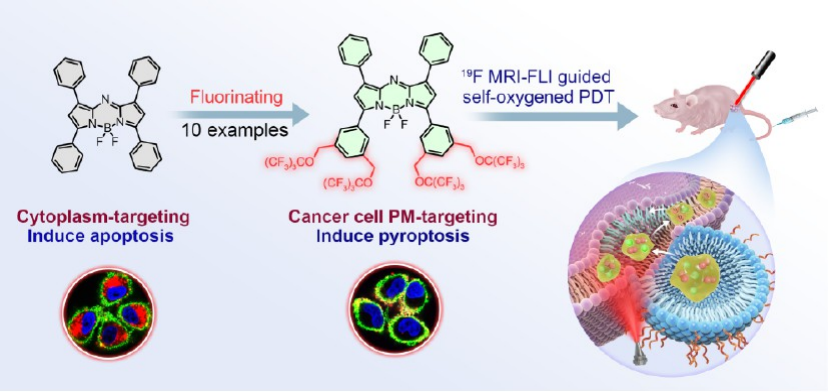

Photodynamic therapy (PDT) holds great potential in cancer treatment, leveraging photosensitizers (PSs) to deliver targeted therapy. Fluorination can optimize the physicochemical and biological properties of PSs for better PDT performance. Here, we report some high-performance multifunctional PSs specifically designed for cancer PDT by fluorinating aza-BODIPY with perfluoro-*tert*-butoxymethyl (PFBM) groups. Fluorination plays several roles, including enhancing selective cancer cell uptake, plasma membrane (PM) targeting, and inducing pyroptosis. It also enables fluorescence imaging (FLI) and fluorine-19 magnetic resonance imaging (^19^F MRI) as well as facilitates oxygen delivery and oxygen partial pressure (pO_2_) measurements. Comparative physicochemical and biological studies, along with molecular dynamics simulations, reveal that fluorinated PSs selectively eradicate cancer cells by oxidizing PM phospholipids with singlet oxygen (^1^O_2_) and inducing pyroptosis, which enables effectively suppressed tumor growth by self-oxygenated ^19^F MRI-FLI-guided PDT in mice. This study demonstrates a fluorination strategy for tailoring high-performance multifunctional cancer PM-targeting materials for cancer therapy and beyond.

## 1 Introduction

Cancer remains a significant global health challenge, with 20 million new cases and 9.7 million deaths reported worldwide in 2022.^1^ Conventional treatments like surgery, radiotherapy, and chemotherapy are often invasive, entail side effects, and may lead to recurrence.^2^ In response, innovative cancer therapies such as immunotherapy, phototherapy, and chemodynamic therapy have emerged, showing promising results. Type II PDT stands out among these, employing PS, oxygen, and light to generate ^1^O_2_ and eliminate cancer cells.^3^ PDT offers noninvasiveness, high spatiotemporal selectivity, and minimal drug resistance, making it a compelling option for cancer treatment.

However, the effectiveness of PDT is often hindered by challenges such as hypoxic tumor microenvironment (TME),^4^ difficulties in targeted PS delivery,^5^ and limited tissue penetration of light.^6^ To address these obstacles, researchers have turned to perfluorocarbons (PFCs) to transport oxygen, alleviate hypoxia, and extend the half-life of ^1^O_2_.^7,8^ Moreover, due to the short lifetime and diffusion distance of ^1^O_2_,^9,10^ organelle-targeted PSs, especially those targeting mitochondria (Mito),^11^ PM,^12−14^ and endoplasmic reticulum (ER),^15^ have shown improved PDT outcomes by minimizing the distance between ^1^O_2_ and the target.

The PM, critical for cell integrity and substance exchange,^16,17^ represents an attractive target for cancer therapy.^18^ Disruption of the PM can effectively induce cancer cell death by bypassing endocytosis, enzymatic metabolism, and unfavorable intracellular conditions.^14^ However, current PM-targeting PSs, typically lipid mimics bearing charged hydrophilic groups^12,19,20^ or long hydrophobic alkyl chains,^21,22^ often lack selectivity for cancer cells, resulting in indiscriminate damage to normal cells. Furthermore, ^1^O_2_ generated by current PSs at the outer PM surface must diffuse into the central layer to oxidize phospholipids,^23,24^ a process hindered by the short lifetime of ^1^O_2_, diffusion distance, and lower local oxygen levels.^25^ Perfluorocarbons, known to accumulate in the central layer of the PM,^26,27^ offer promise in enhancing the PDT efficacy by targeting unsaturated phospholipid tails in this region with enriched oxygen and longer-lived ^1^O_2_.

**Scheme 1:**
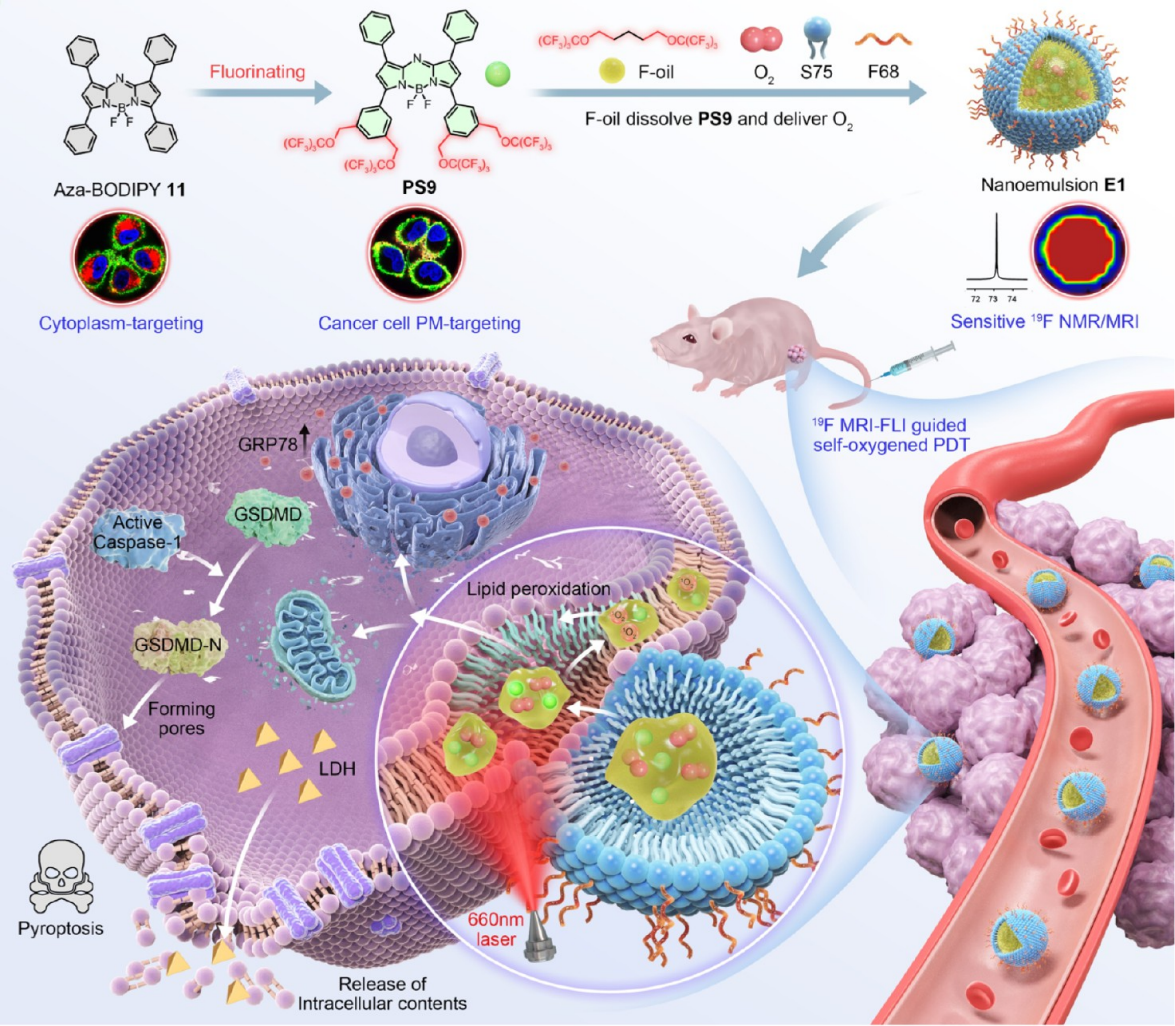
Fluorinating Aza-BODIPY for Cancer Cell PM-Targeted, ^19^F MRI-FLI-Guided, and Self-Oxygenated PDT of Lung Cancer. The structures of aza-BODIPY **11**, **PS9**, and **F-oil** are shown here, and those of **PS1**-**PS10** and **11**-**13** can be found in Figure S1

Herein, we have selectively fluorinated 1,3,5,7-tetraphenyl-aza-BODIPY to create a panel of novel PSs, namely, **PS1**-**PS10**, which exhibit high cancer cell PM-targeting capability (Scheme 1 and Figure S1). Through a systematic investigation of their structure–property relationship, we found that **PS9** demonstrates particularly favorable physicochemical properties and high efficacy in cancer cell PM-targeted, ^19^F MRI-FLI-guided, and self-oxygenated PDT of lung cancer by disrupting the PM and inducing pyroptosis in both cellular and murine models. The strategic use of PFBM groups for fluorination mitigates the ACQ of fluorescence (FL) and enhances PDT efficacy. Furthermore, fluorination facilitates oxygen delivery, alleviates hypoxia, and boosts ^1^O_2_ generation for cancer cell ablation.^28,29^ Multiple PFBMs also generate a robust ^19^F signal, enabling sensitive “hot-spot” ^19^F MRI tracking of the PS and quantitative ^19^F *R*_1_ measurement of pO_2_ to optimize PDT.^30−32^ Additional enhancements in oxygen delivery, optical properties, and ^19^F MRI sensitivity are achieved using **F-oil**(30) to dissolve the PSs. Aza-BODIPY analogues **11**–**13**^33^ serve as controls to elucidate the impact of fluorination on PM targeting, PDT efficacy, and FL capabilities. These comparative studies underscore the benefits of fluorination in enhancing PDT efficacy through precise PM targeting, improved oxygen delivery, and accurate measurement, highlighting the potential of fluorinated theranostics in cancer therapy.

## 2 Results and Discussion

### 2.1 Synthesis, Property, and Screening of Photosensitizers

**Figure 1:**
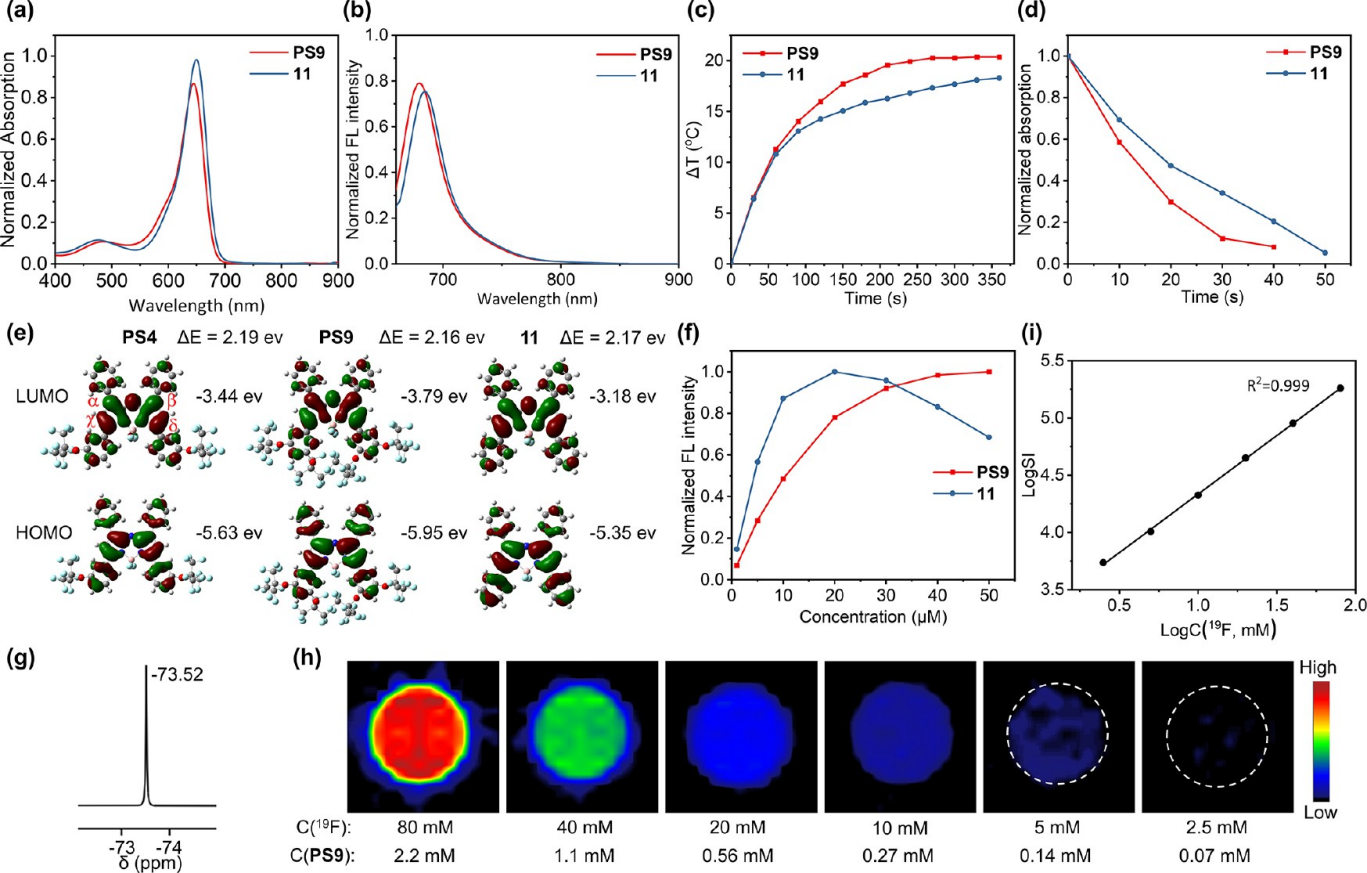
Normalized UV–vis absorption (a) and FL emission (b) spectra of **PS9** and **11**. Time-dependent temperature changes (c) and maximum absorption intensities of DPBF (d) of **PS9** and **11** under 660 nm laser irradiation at 0.5 W cm^–2^. Calculated HOMO and LUMO of the **PS4**, **PS9**, and **11** (e). Normalized concentration-dependent FL intensity of **PS9** and **11** (f). Partial ^19^F NMR spectrum (g), ^19^F MRI phantom images (h), and plot of LogSI versus LogC (^19^F) (i) of **PS9**. CHCl_3_ was used as the solvent. The concentrations of PSs in (a–d) were 10, 10, 20, and 1 μM, respectively. **PS9** was selected as a representative fluorinated PS and **11** as a typical nonfluorinated PS, while the corresponding figure for the rest of PSs in Figure S1 can be found in the Supporting Information.

First, **PS1**-**PS10** were synthesized on multihundred-milligram scales, and their structures were confirmed using ^1^H/^13^C/^19^F NMR and high-resolution mass spectrometry (Scheme S1). Then, the structure–property relationship of the fluorinated PSs was comparatively investigated. **PS1**-**PS10** exhibited intense absorption and emission with notable Stokes shifts (Figures 1a,b and S2). Introducing PFBM groups into the phenyl rings caused negligible wavelength shifts compared to those of unsubstituted aza-BODIPY **11**. However, **PS5** and **PS6**, containing electron-donating methoxy groups at the *para*-positions of the phenyl rings, showed significant red shifts in absorption and emission. Furthermore, **PS5** showed a larger red shift (a 43 nm red shift in absorption and a 50 nm shift in emission), consistent with the well-documented effect of electron-donating groups on the lower-sphere of aza-BODIPY, which enhances electron density delocalization.^34,35^ Interestingly, upper-sphere fluorinated PSs usually exhibited higher molar extinction coefficients (ε) and fluorescence quantum yields (ϕ_f_) compared to those of lower-sphere fluorinated counterparts (Table S1). An exception is **PS8**, whose ϕ_f_ is lower than that of **PS9**, likely due to decreased rigidity and coplanarity caused by the four bulky PFBM groups on the upper-sphere phenyl rings,^33^ as confirmed by the larger dihedral angles in **PS8** (Table S2). Upon 5 min of irradiation with a 660 nm laser at 0.5 W cm^–2^, the PSs exhibited temperature increments (Δ*T*) up to 20.8 °C (Figures 1c and S3), demonstrating a high photothermal conversion capability. The reactive oxygen species (ROS)-generating capability of the PSs was validated by time-dependent UV–vis absorption spectra using DPBF (a ROS probe; Figures 1d and S4), with **PS9** showing the fastest consumption of DPBF within 35 s. ROS quantum yield (ϕ_Δ_) revealed higher values for PSs with lower-sphere fluorination (Table S1). Trifluoromethylation in **PS7**, consistent with previous findings,^33^ significantly improved ϕ_Δ_.

To assess the impact of fluorination, the HOMO and LUMO energy levels of the PSs were calculated using density functional theory (DFT, Figures 1e and S5). Upper-sphere fluorinated PSs exhibited localized HOMO and LUMO energy levels mainly in the aza-BODIPY core and lower sphere, enhancing ε and ϕ_f_ compared to aza-BODIPY **11**. Lower-sphere fluorination had minimal effect on HOMO and LUMO energy gaps (Δ*E*),^36,37^ but significantly promoted coplanar conformation of aromatic groups. This structural feature reduced the energy lost via nonradiative transitions and increased the quantum yield, thereby enhancing the ϕ_Δ_ for **PS4** and **PS9** (Figure S5 and Table S2).^38,39^

Among the PSs, **PS9** demonstrated significant potential for imaging, PDT, and photothermal therapy (PTT, ε = 81130, ϕ_f_ = 0.26, ϕ_Δ_ = 0.22, Δ*T* = 20.4 °C). While **PS7** exhibited higher ϕ_f_ (0.38) and ϕ_Δ_ (0.47), its low synthetic yield (3%) and limited photothermal conversion efficiency (Δ*T* = 15.1 °C) rendered it less practical. Moreover, compared with aza-BODIPY **11**, **PS9** showed no apparent ACQ of FL at concentrations up to 50 μM (Figure 1f). With 36 magnetically equivalent fluorines, **PS9** exhibited an intense singlet ^19^F NMR peak at −73.52 ppm and a high transverse relaxation time (*T*_2_)-to-longitudinal relaxation time (*T*_1_) ratio of 0.72 (Figure 1g and Table S3), ideal for sensitive ^19^F MRI.^40^**PS9** was effectively imaged at 0.14 mM with a signal-to-noise ratio (SNR) of 5.37 and a short data acquisition time of 256 s (Figure 1h). Importantly, the logarithmic signal intensity (LogSI) was directly proportional to fluorine concentration (LogC(^19^F), Figure 1i), facilitating accurate **PS9** quantification by ^19^F MRI. These combined features establish **PS9** as the optimal candidate for ^19^F MRI-FLI-guided phototherapy applications.

### 2.2 Formulation and Optimizations

**Figure 2:**
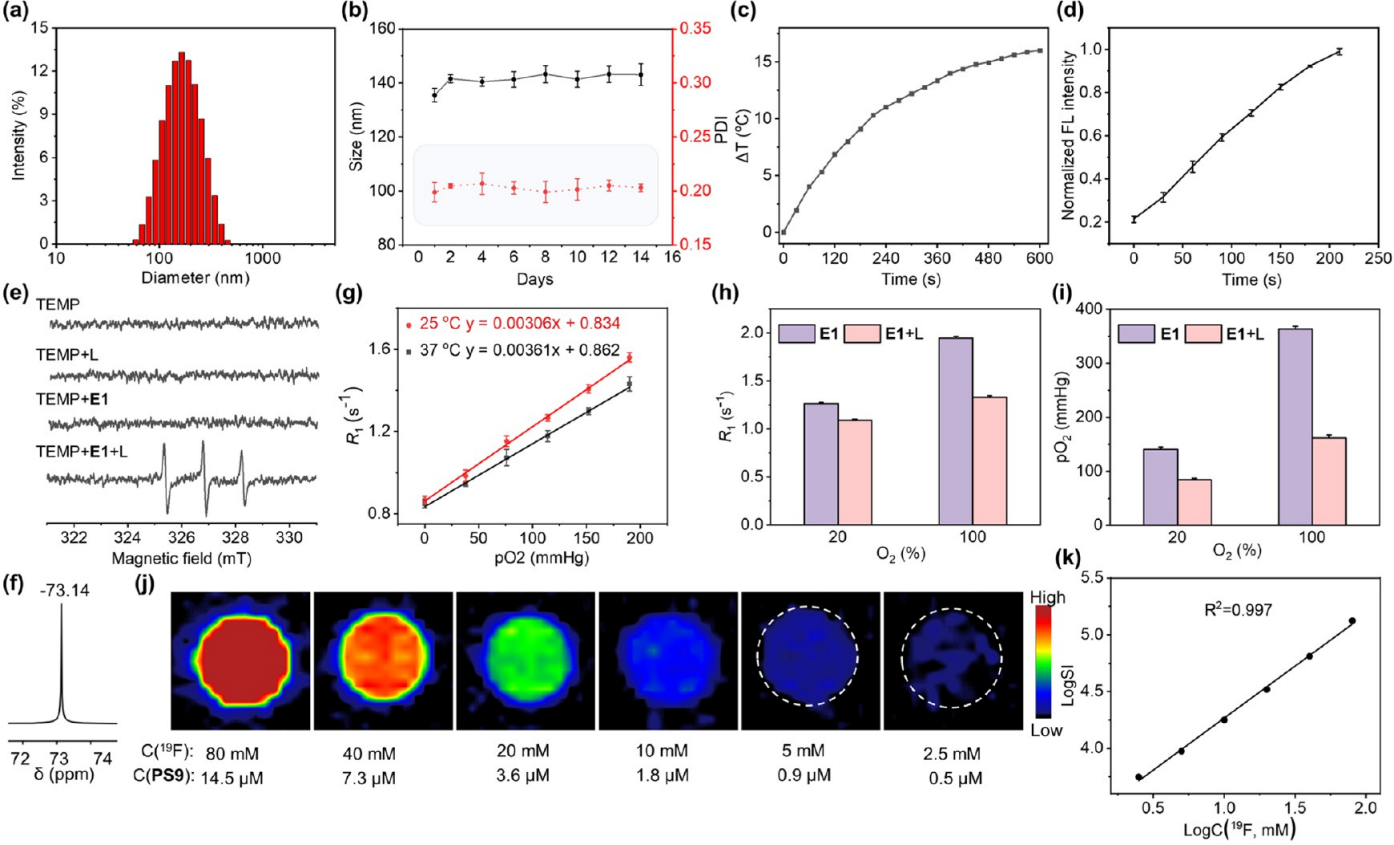
DLS (a), particle size and PDI monitored by DLS (b) of **E1**. Temperature increases of **E1** (c, C_**PS9**_ = 20 μM) and normalized SOSG FL intensity at 525 nm of **E1** (d, C_**PS9**_ = 1 μM) under 660 nm laser irradiation at 0.5 W cm^–2^. EPR spectra of **E1** and controls under the indicated conditions (e). Partial ^19^F NMR spectrum of **E1** (f). Plot of ^19^F *R*_1_ versus pO_2_ of **E1** (g). ^19^F *R*_1_ (h) and calibrated pO_2_ (i) of **E1** samples under the indicated conditions. ^19^F MRI phantom images (j) and plot of LogSI versus LogC(^19^F) (k) of **E1**.

Despite its low water solubility due to high fluorine content (F% = 48%), **PS9** exhibited high solubility in fluorinated solvent **F-oil** (up to 6.5 mM), which creates an oxygen-rich fluorous phase beneficial for PDT. Nanoemulsion **E1**, formulated with **PS9**, **F-oil**, phospholipid S75, and Pluronic F68 using a thin-film dispersion method, exhibited monodisperse particles (∼133 nm in diameter, polydispersity index (PDI) of 0.19) as measured by dynamic light scattering (DLS, Figure 2a). Cryo-electron microscopy (Cryo-EM, Figure S6) confirmed the spherical morphology of **E1**, which maintained stability with minimal changes in size and PDI over 14 days (Figure 2b).

Control nanoemulsions, including **E2** (without **PS9**), **E3** (with aza-BODIPY **11** instead of **PS9**), **E4** (with soybean oil instead of **F-oil**), and **E5-E7** (with **PS4**, **PS8**, and **PS10** instead of **PS9**, respectively), were also prepared (Table S4). First, the optical properties of nanoemulsions **E1**-**E7** were examined (Figure S7a,b). After formulation, their maximum absorption wavelengths remained similar to those of the PSs, around 650 nm. Moreover, due to the dispersion of PSs in **F-oil**, the fluorescence was not quenched in the aqueous system, with the maximum emission wavelengths observed around 670 nm, indicating their applicability for cellular and in vivo fluorescence imaging. Upon laser irradiation, **E1** exhibited a temperature increase of 16 °C (Figure 2c). Time-dependent UV–vis absorption spectra of DPBF indicated **E1**’s high ROS-generating ability, depleting DPBF within 40 s (Figure S7c). Using SOSG as an ^1^O_2_ probe, **E1** generated a high concentration of ^1^O_2_ after 210 s of laser irradiation (Figures 2d and S7d), confirming its efficacy as a type II PDT agent. Electron paramagnetic resonance (EPR, Figure 2e) spectroscopy further confirmed ^1^O_2_ generation by **E1** under laser irradiation, validating its well-maintained photothermal and photodynamic efficiencies post-formulation.

The high fluorine content in **E1** endowed it with oxygen delivery capabilities, real-time pO_2_ measurement, and “hot spot” image tracking. **E1** exhibited a unified intense singlet ^19^F NMR peak at −73.14 ppm, enhancing ^19^F MRI sensitivity and avoiding imaging artifacts from chemical shifts (Figure 2f). The ^19^F *R*_1_ of **E1** provided real-time quantification of oxygen,^41^ showing excellent linear relationships with pO_2_ at room and body temperatures (Figure 2g). Notably, measurement of pO_2_ is critical for optimizing PDT by adjusting the **PS9** dose and laser irradiation duration. After 10 min of 660 nm laser irradiation at 0.5 W cm^–2^, **E1** samples saturated with 20% and 100% oxygen showed significant reductions in ^19^F *R*_1_ (Figure 2h), indicating dramatic oxygen consumption. Using the standard curve fitted from Figure 2g, pO_2_ in **E1** samples decreased from 140 and 363 mmHg to 84 and 161 mmHg, respectively (Figure 2i), suggesting still a lot of O_2_ in the nanoemulsion for PDT. Meanwhile, the intense singlet ^19^F NMR peak from **PS9** and **F-oil** enabled quantitative detection of **PS9** by ^19^F MRI at a low concentration of 0.5 μM with an SNR of 3.34 and a short data acquisition time of 256 s (Figure 2j,k), a 280-fold improvement in the detectable concentration.

### 2.3 In Vitro Assays

**Figure 3:**
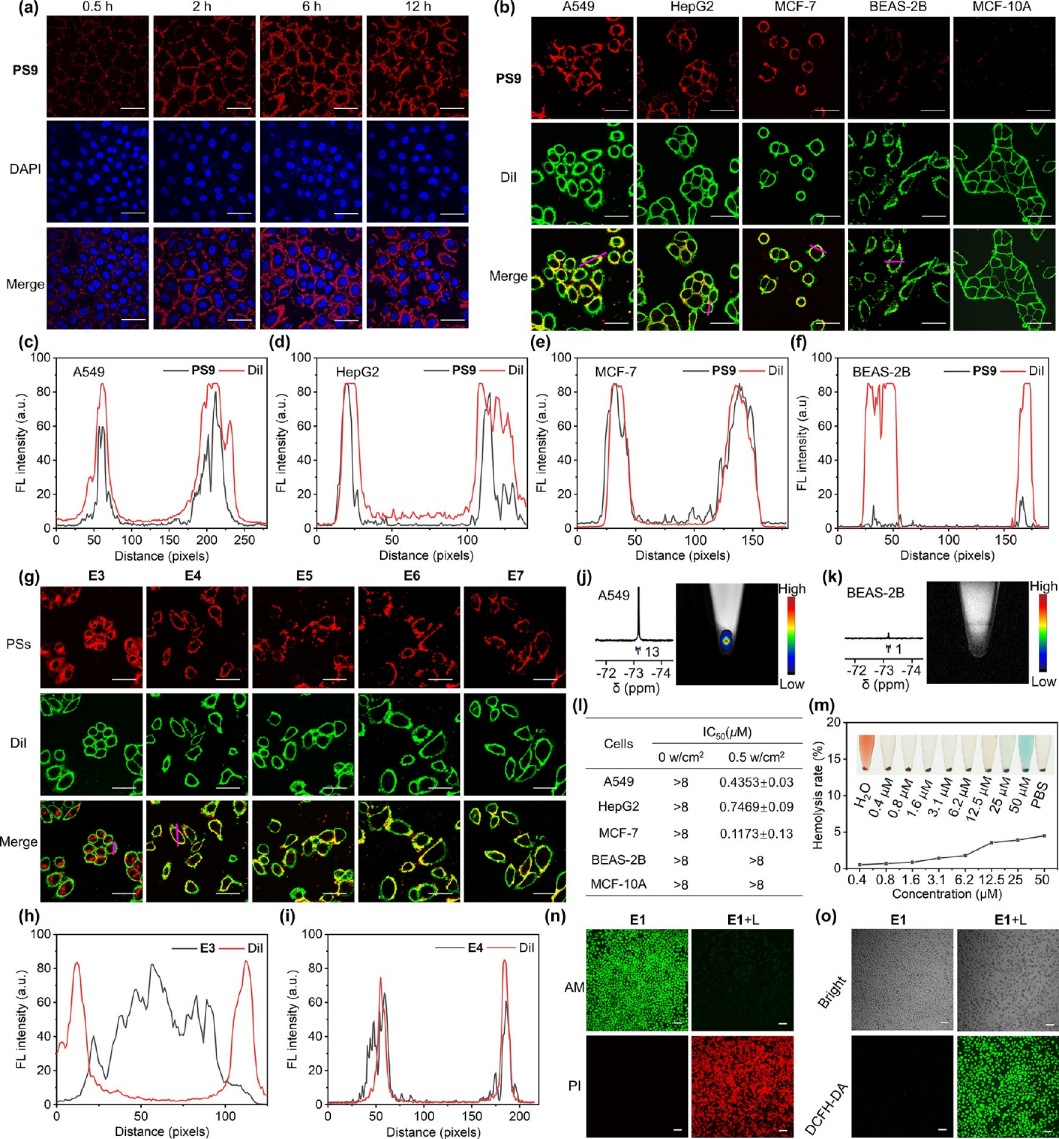
Confocal microscopy images of **E1**-treated A549 cells (a). Colocalization images of **PS9** with DiI in **E1**-treated cells (b) and the corresponding FL intensity correlation plot along the magenta lines in the confocal images of A549 (c), HepG2 (d), MCF-7 (e), and BEAS-2B cells (f). Colocalization images of PS with DiI in **E3**-**E7**-treated A549 cells (g) and the corresponding FL intensity correlation plot of **E3** (h) and **E4** (i). ^19^F NMR and ^19^F MRI of 1 × 10^7^**E1**-treated A549 (j) and BEAS-2B cells (k). IC_50_ of **E1**-treated cells (l). Hemolysis assay of **E1** (m). Confocal microscopy images of **E1**-treated A549 cells with AM-PI double staining (n) or DCFH-DA staining (o). Scale bars: 50 μm for (a), (b), and (g), 100 μm for (n) and (o).

We investigated the organelle-targeting ability of the photosensitizers across a range of human cancer and normal cells. Time-dependent FL intensity analysis of **E1**-treated A549 cells demonstrated gradual uptake of **PS9** on the PM, reaching peak levels at 6 h (Figures 3a and S8). Co-incubation of A549 cells with **E1** and the PM stain DiI confirmed PM targeting through colocalization of DiI and **PS9** in confocal microscopy images (Figure 3b), supported by FL intensity correlation analysis (Figure 3c). Similar observations were noted in HepG2 and MCF-7 cells, whereas the minimal FL intensity of **PS9** was observed on the PMs of normal cells, including BEAS-2B and MCF-10A cells (Figures 3b,d–f and S9), highlighting selective targeting of cancer cell PMs. Staining of **E1**-treated A549 cells with the ER, mitochondria, and lysosomal dyes revealed no colocalization of **PS9** with these organelles (Figure S10). Treatment of A549 cells with **E3** showed localization of **11** in the cytoplasm only (Figure 3g,h), while treatment with **E4** containing soybean oil instead of **F-oil** still showed clear FL of **PS9** on the PM (Figure 3g,i). Similarly, **PS4**, **PS8**, and **PS10** exhibited FL exclusively on the PM of **E5-E7**-treated A549 cells (Figures 3g, S11, and S12), emphasizing the general ability of fluorinated PSs to target cancer cell PMs. Quantitative ^19^F NMR analysis of **E1**-treated cells further confirmed cancer cell targeting, revealing a 13-fold higher ^19^F NMR signal intensity in A549 cells compared to that in BEAS-2B cells (Figure 3j,k). Notably, cancer cell targeting was also visualized using ^19^F MRI (Figures 3j,k), enabling the selective ^19^F MRI detection of cancer cells.

The phototherapy efficacy of **E1** was then evaluated in the cells. Using the CCK-8 cytotoxicity assay, we observed no significant cytotoxicity of **E1** in all cells under dark conditions. However, upon 10 min of 660 nm laser irradiation at 0.5 W cm^–2^, high cytotoxicity was observed in cancer cells (including A549, HepG2, and MCF-7 cells, Figure S13a-c), indicating the high phototherapy efficacy of **E1** with minimal dark cytotoxicity. Conversely, **E1**-treated BEAS-2B and MCF-10A cells exhibited significantly lower cytotoxicity (Figure S13d,e). Additionally, IC_50_ measurements demonstrated at least a 10-fold higher IC_50_ against normal cells compared to cancer cells after laser irradiation (Figure 3l), highlighting the selective phototoxicity of **E1** against cancer cells. **E2** without **PS9** showed negligible photocytotoxicity against A549 cells, while unformulated **PS9** or **E3** containing **11** exhibited lower photocytotoxicity compared to **E1** (Figure S13f-h), underscoring the importance of cancer cell PM targeting and formulation in enhancing phototherapy efficacy.

Notably, the cytotoxicity of **E1** toward A549 cells under hypoxic condition remained high, whereas nanoemulsions **E4**, in which the **F-oil** was replaced with soybean oil, exhibited low cytotoxicity toward A549 cells under hypoxic conditions (Figure S13i,j). This highlights the critical role of oxygen-carrying **F-oil** in mitigating hypoxia in tumor environments, thereby enhancing the efficacy of PDT. Furthermore, a hemolysis assay showed no significant hemolysis (<5%) up to 50 μM **PS9** (Figure 3m), confirming high biocompatibility. Using calcein acetoxymethyl ester-propidium iodide (AM-PI) double staining, we observed negligible green FL of AM from live A549 cells and strong red FL of PI from dead cells post-laser irradiation, indicating high PDT efficacy, with minimal dark cytotoxicity (Figure 3n). Finally, the bright green FL of DCFH-DA in **E1**-treated A549 cells after laser irradiation revealed substantial ROS generation by **E1** (Figure 3o).

**Figure 4:**
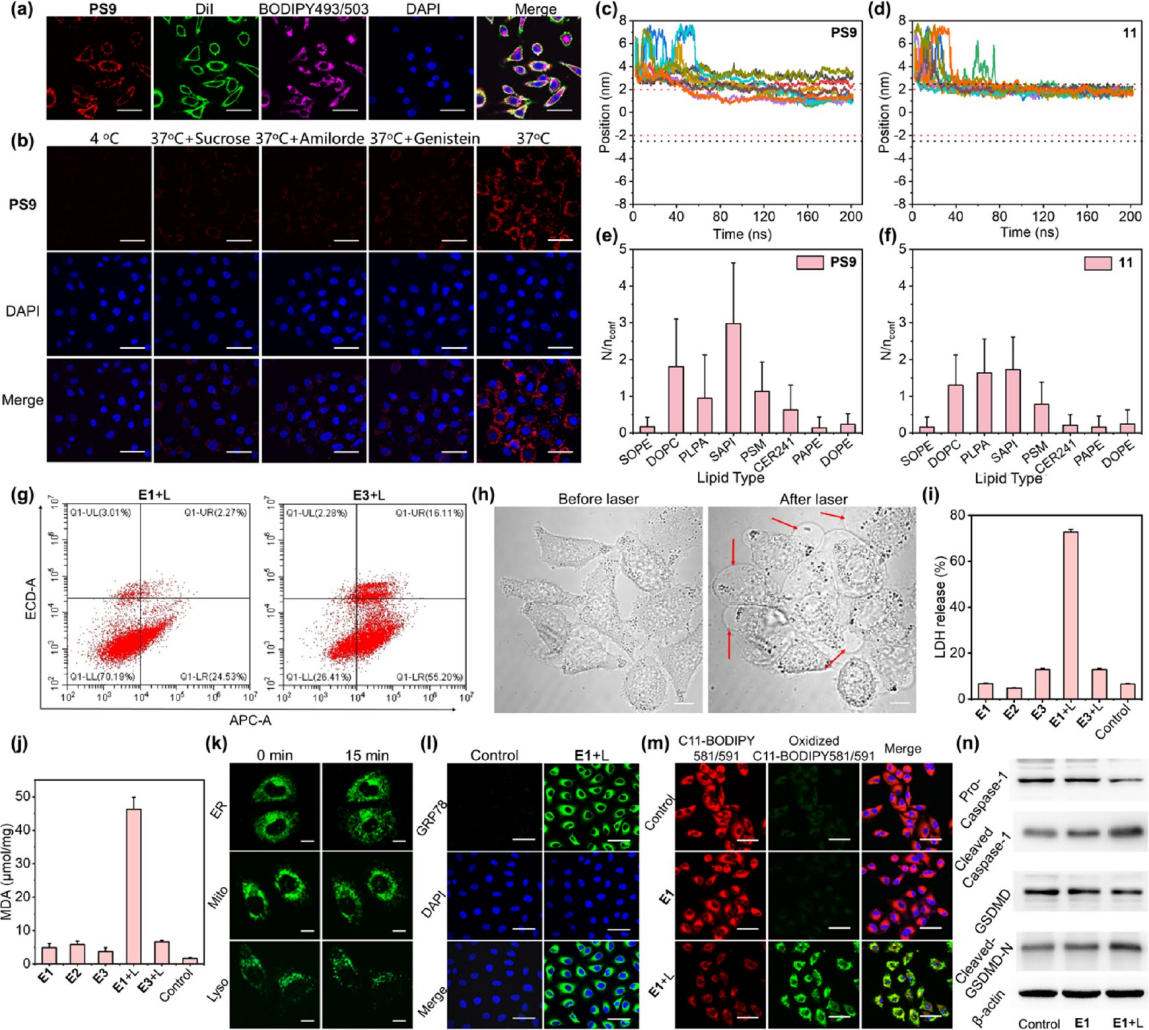
Confocal microscopy images of **PS9** with DiI, BODIPY 493/503, and DAPI in **E8**-treated A549 cells (a). Confocal microscopy images of A549 cells incubated with **E1** at 4 °C or in the presence of endocytosis inhibitors (b). MD simulations of the distribution of 10 **PS9** (c) and **11** (d) molecules in A549 cell PM and the number of phospholipids that interact with **PS9** (e) and **11** (f). Flow cytometry profiles of A549 cells treated with **E1** and **E3** and stained with Annexin V-APC/7-AAD after laser irradiation (g). Bright-field microscopy images of **E1**-treated A549 cells before and after laser irradiation (h). Quantitative analysis of LDH in culture medium (i) and MDA from the lysate (j) of A549 cells under the indicated conditions. Confocal microscopy images of **E1**-treated A549 cells stained with ER-Tracker, MitoTracker, and LysoTracker after laser irradiation (k), with immunofluorescence staining GRP78-Alexa 488 (l), and with LPO staining C11-BODIPY 581/591 (m). Western blot of **E1** and laser-treated A549 cell lysate with controls (n). 10 min time for 660 nm laser irradiation at 0.5 W cm^–2^ was used in all cases. Control groups were cells without drug and laser treatments. Scale bars: 50 μm for (a), (b), (l), and (m), 10 μm for (h) and (k).

Then, the mechanism underlying the cancer cell PM targeting was investigated. Confocal images of **E8**-treated A549 cells containing both **PS9** and BODIPY 493/503 showed the FL of **PS9** on the PM and the FL of BODIPY 493/503 in the cytoplasm (Figure 4a), suggesting nanoemulsion dissociation during uptake. Incubation of A549 cells with **E1** at 4 °C resulted in over 90% reduction in the FL intensity of **PS9**, indicating an active cellular uptake pathway (Figures 4b and S14). Co-incubation of A549 cells with **E1** and endocytosis inhibitors (sucrose, amiloride, and genistein)^42,43^ reduced the FL intensity of **PS9** by 59–70%, confirming endocytosis as the primary uptake pathway (Figures 4b and S14). These findings align closely with the contact-facilitated drug delivery (CFDD) model proposed by Wickline et al.^44^ According to CFDD, **E1** first interacts with PM and releases its contents into PM, while the bulky **PS9** and fluorous **F-oil** face challenges crossing from the hydrophobic PM into the hydrophilic cytoplasm, resulting in their entrapment in the PM (Figure 4b).^45^

Molecular dynamics simulations were conducted to further explore cancer cell PM targeting. Using the PMEMD module of AMBER v20 software,^46,47^ we investigated the penetration depth of **PS9** and **11** in an A549 PM model constructed by the CHARMM-GUI tool,^48−50^ according to membrane phospholipid phenotype.^51^ Simulations indicated that **PS9** tends to penetrate deeply and reside in the central layer of the PM, while **11** predominantly remains in the surface layer (Figure 4c,d). Additionally, simulations of PS-phospholipid affinity demonstrated stronger interactions of **PS9** with monounsaturated phosphatidylcholines in the A549 cells PM, which is present at lower levels in normal cells.^51,52^ This further supports the selective entrapment of **PS9** in cancer cell plasma membranes. (Figure 4e,f).

Next, the PDT mechanism of **E1** was investigated. Flow cytometric analysis of **E1** and laser-treated A549 cells revealed that apoptosis was a minor pathway of cell death, whereas **E3** and laser-treated A549 cells predominantly underwent apoptosis (Figure 4g), indicating that **PS9** targeting the PM leads to a distinct PDT mechanism compared to that of **11**. Bright-field microscopy showed evident blebbing and PM integrity loss in **E1** and laser-treated A549 cells (Figure 4h), resulting in leakage of intracellular components, such as lactate dehydrogenase (LDH) into the extracellular space. Compared to the dramatic LDH increase in **E1** and laser-treated A549 cells (Figure 4i), a marginal LDH increase was observed in **E3** and laser-treated A549 cells, or **E1**-treated A549 cells in the dark, underscoring that PDT with **PS9**-induced PM collapse and subsequent LDH release.

Using the lipid peroxidation (LPO) MDA assay, a significant rise in lipid peroxides was detected in **E1** and laser-treated A549 cells (Figure 4j), indicative of PM phospholipid oxidation by ^1^O_2_ generated from **PS9**. Confocal microscopy images of **E1**-treated A549 cells with organelle staining revealed ER collapse and mitochondrial fission post-laser irradiation (Figure 4k), characteristic of ER stress-induced cell death. The intense green FL of GRP78-Alexa 488, an ER stress marker, in **E1** and laser-treated A549 cells confirmed ER stress-induced cell death (Figure 4l). Additionally, the prominent green FL of oxidized C11-BODIPY 581/591, an LPO probe, was observed in the cytoplasm of **E1** and laser-treated A549 cells (Figure 4m). Western blot analysis demonstrated an increased expression of cleaved Caspase-1 and GSDMD-N in **E1** and laser-treated A549 cells (Figure 4n), indicating that PM-targeted phospholipid oxidation by **PS9** PDT triggers pyroptosis in A549 cells.

### 2.4 In Vivo Assays

**Figure 5:**
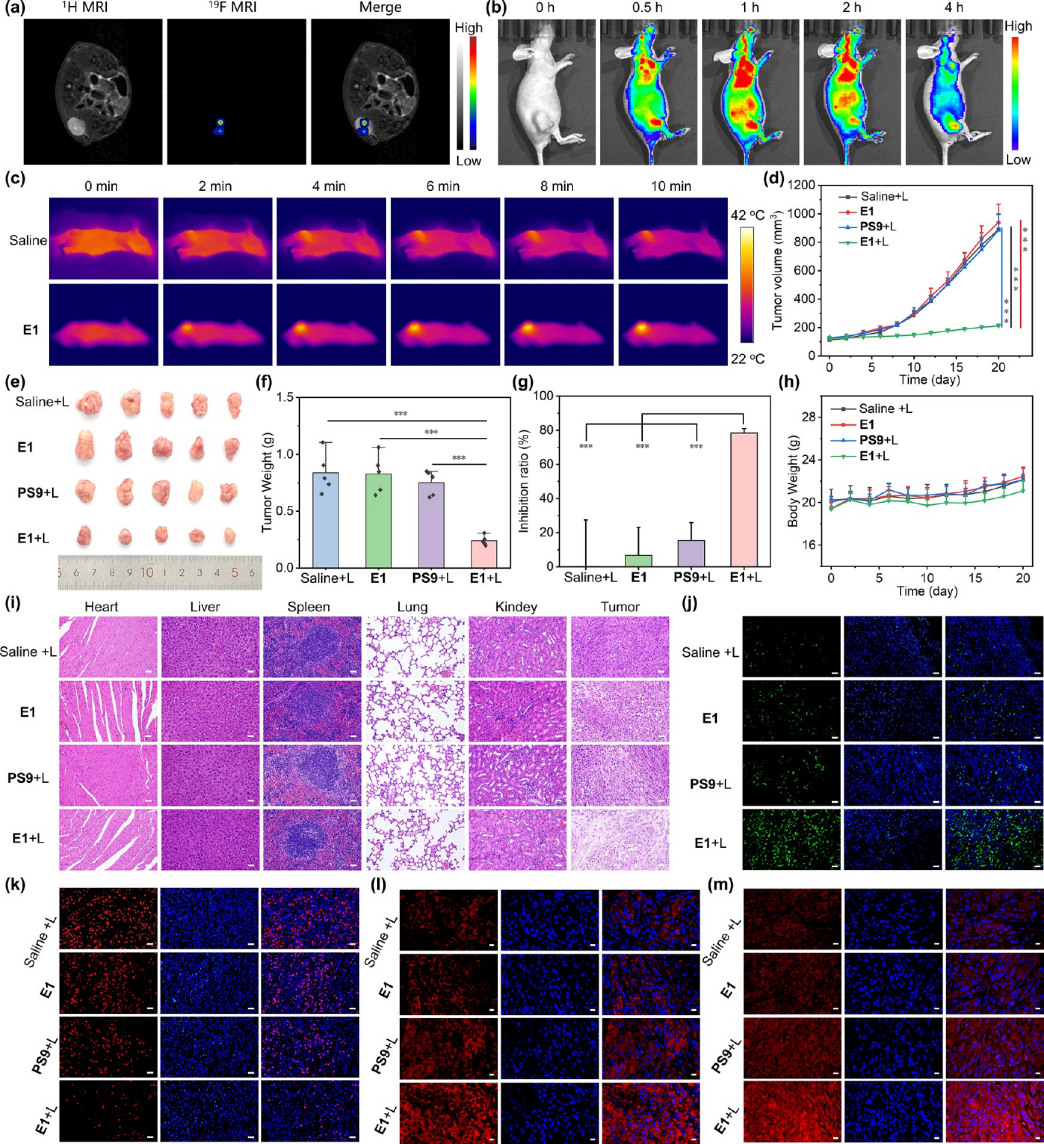
^1^H/^19^F MRI (a), whole-body FLI (b), and thermal images after laser irradiation (c) of nude mice bearing A549 tumors after the *i.v.* injection of **E1**. Tumor growth curves (d). Photos (e) and weights (f) of tumors harvested after treatments, tumor inhibition rates (g), and body weight curves (h) of the 4 treatment groups of mice. H&E staining of internal organs and tumor tissue collected from the 4 treatment groups of mice (i). Representative TUNEL staining (j), *K*_i_-67 staining (k), immunofluorescence staining of cleaved caspase-1 (l), and GSDMD-N (m) of tumor tissues collected from the treatment groups of mice. Scale bars: 50 μm for (i), (j), and (k), 20 μm for (l) and (m). Data were presented as mean ± standard deviation (*n* = 5, asterisks indicate statistical significance, ***p < 0.001).

Acute toxicity assessment via intravenous (*i.v.*) injection of **E1** at 0.8 mg/kg **PS9** into BALB/c nude mice showed no significant body weight loss or abnormalities in behavior and blood count analysis (Figure S15 and Table S5). “Hot spot” ^19^F MRI images and intense FLI signals in the tumor region of nude mice bearing A549 tumors demonstrated effective tumor targeting by **E1** (Figure 5a,b). Thermal imaging of **E1** and laser-treated mice indicated a moderate temperature increase of approximately 7 °C in the tumor region (Figure 5c), suggesting a minor role of PTT.

Mice bearing A549 tumors were divided into four groups and treated with saline, **PS9**, and **E1** (2 groups), respectively, followed by laser irradiation of 3 groups every 4 days (Figure S16). Tumor growth curves (Figure 5d) and post-treatment tumor analysis (Figure 5e,f) showed a significant inhibition rate of 80% in **E1** and laser-treated mice, whereas inhibition rates were below 20% in **PS9** and laser-treated mice or **E1**-treated mice without laser irradiation (Figure 5g). Importantly, no significant body weight loss was observed in any of the treatment groups (Figure 5h).

Histological hematoxylin and eosin (H & E) staining of internal organs collected post-therapy revealed no abnormalities associated with **E1** treatment (Figure 5i), while evident tissue damage was observed in the tumors of **E1** and laser-treated mice. Strong FL in the TUNEL staining of tumor tissues indicated efficient tumor apoptosis induced by the PDT of **PS9** (Figure 5j). *K*_i_-67 staining showed significantly reduced expression of *K*_i_-67 in **E1** and laser-treated tumor tissues, indicative of the antiproliferative effects of PDT (Figure 5k). Immunofluorescence staining of pyroptosis biomarkers in tumor tissues demonstrated higher levels of cleaved caspase-1 (Figure 5l) and cleaved-GSDMD-N (Figure 5m) in **E1** and laser-treated mice, confirming PDT-induced pyroptosis. Therefore, the dual imaging capabilities of ^19^F MRI-FLI, coupled with the effective tumor targeting and potent PDT efficacy of **E1**, highlight its potential for highly efficient lung cancer therapy in preclinical models.

## 3 Conclusions

In conclusion, we have developed a novel fluorination strategy for high-performance multifunctional photosensitizers specifically designed for enhanced cancer PDT. This strategy involves the selective fluorination of aza-BODIPY with multiple perfluoro-*tert*-butoxymethyl groups, resulting in a series of fluorinated aza-BODIPYs that exhibit selective uptake by various cancer cells and remain localized in their PM, demonstrating the robustness and generality of this fluorination approach. Our comprehensive structure–property relationship studies have shown that these fluorinated aza-BODIPYs not only inherit high FL, PTT, and PDT efficiencies but also display high capabilities of ACQ-free FL, sensitive “hot spot” ^19^F MRI, oxygen delivery, and pO_2_ measurement, thereby establishing themselves as versatile high-performance dual-modal imaging agents and photosensitizers. Through comparative cellular studies and molecular dynamics simulations, we have elucidated how the unique physicochemical properties conferred by fluorination facilitate the precise targeting of cancer cell PM. This targeting capability enhances the oxidation of phospholipids within the PM, selectively inducing pyroptosis in cancer cells while maintaining excellent biocompatibility with normal cells. Importantly, these properties endow the photosensitizers with targeted PM delivery, ^19^F MRI-FL dual imaging guidance, and self-oxygenation capabilities for PDT, effectively inhibiting the growth of A549 lung tumor in a murine model. This comprehensive study provides mechanistic insights into how fluorination enhances PDT efficacy through precise plasma membrane targeting, oxygen delivery, and dual imaging guidance. However, fluorination has impacted the solubility of aza-BODIPYs, making them poorly soluble in most solvents. Furthermore, the absorption and emission wavelengths of fluorinated aza-BODIPYs do not extend beyond 900 nm, which limits their imaging capability in deeper tissues. Overall, our findings underscore the potential of fluorination in advancing the development of high-performance multimodal imaging agents, photosensitizers, and bioactive agents for cancer therapy and beyond.

## 4 Experimental Procedure

### 4.1 Synthesis and Characterization of Fluorinated PSs

Take the synthesis of **PS1** as an example. The mixture of **1b** (0.65 g, 1.26 mmol) and ammonium acetate (3.39 g, 43.97 mmol) was stirred at 120 °C for 5 h. After cooling to room temperature, the reaction mixture was washed with water, extracted with dichloromethane (DCM), and recrystallized from DCM and methanol to give crude product (0.22 g), which was used in the next step without further purification. Under an argon atmosphere, the intermediate mentioned above and *N*,*N*-diisopropylethylamine (DIPEA, 0.99 g, 6.98 mmol) were dissolved in dried DCM, and the resulting solution was stirred at room temperature for 20 min. Then, the boron trifluoride diethyl etherate complex (BF_3_·Et_2_O, 0.60 g, 4.65 mmol) was added, and the resulting solution was stirred at room temperature for 24 h. After the reaction mixture was quenched with water, the organic layer was collected and the aqueous layer was extracted with DCM. The combined organic layer was dried over anhydrous Na_2_SO_4_ and concentrated under vacuum. The residue was purified by flash chromatography to give compound **PS1** as a brown solid (66 mg, yield 10%).^**1**^**H NMR** (500 MHz, CDCl_3_) δ 8.09–8.02 (m, 8H), 7.52–7.48 (m, 6H), 7.44 (d, *J* = 8.1 Hz, 4H), 7.06 (s, 2H), 5.11 (s, 4H). ^**19**^**F NMR** (471 MHz, CDCl_3_) δ −73.36 (s, 18F), −134.54 – −134.75 (m, 2F). ^**13**^**C NMR** (126 MHz, CDCl_3_) δ 160.0, 145.8, 143.6, 136.2, 132.8, 131.6, 131.2, 129.8, 128.8, 128.0, 129.73, 120.6 (q, *J* = 291.6 Hz), 119.4, 80.8–79.4 (m), 71.1. **HRMS** (MALDI-TOF) *m*/*z*: [M]^+^ calcd for C_42_H_24_BF_20_N_3_O_2_^+^: 993.1643, found 993.1635.

### 4.2 FL Quantum Yield Measurements

Aza-BODIPY **11** (ϕ_f_ = 0.34, in CHCl_3_) was used as the reference for calculating the quantum yield.^53^ PSs were dissolved in CHCl_3_ at concentrations corresponding to UV absorption values of 0.01–0.05 to minimize reabsorption effects. Quantum yields were calculated using the following formula (1):^54^
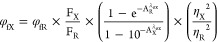
1

where X and R represent the PSs and the known standard reference substance **11,** respectively, F denotes the integrated area of the FL spectrum, A (λ_ex_) represents the absorbance at the excitation wavelength, and η represents the refractive index of the solvent.

### 4.3 Detection of ROS Generation of PSs

The generation of singlet oxygen from **PS1**-**PS10** and aza-BODIPYs **11**-**13** was investigated by using DPBF as an indicator. A solution containing 1 μM PS was mixed with 40 μM DPBF in CHCl_3_. The absorbance changes of DPBF at 415 nm under irradiation (660 nm) at 0.5 W cm^–2^ were recorded. The final result represents the average of three parallel tests. Tetraphenylporphyrin (TPP) in CHCl_3_ (ϕ_Δ_ = 0.55) was used as the reference.^55^ The photooxidation of DPBF was monitored between 0 and 4 min, depending on the efficiency of the PSs. Singlet oxygen quantum yields were calculated using the following formula (2):^56^
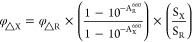


where X and R represent the PSs and the known standard reference substance TPP, respectively, ϕ_Δ_ denotes the quantum yield of singlet oxygen, S is the slope of the plot representing the change in absorbance of DPBF (at 415 nm) over irradiation time, and (1–10^–A^) is the absorption correction factor, derived from the absorbance at the irradiation wavelength.

### 4.4 Preparation of E1-E8

The preparation of **E1** was used as a typical procedure: Phospholipid S75 (60 mg), **F-oil** (100 mg), and **PS9** (2 mg) were dissolved in DCM and evaporated under vacuum. Then, 3 mL of a deionized water solution containing 30 mg of F68 was added, followed by ultrasonic treatment for 15 min. The mixture was then filtered through a 0.22 μm filter membrane to afford emulsion **E1**. Similarly, **E2**, **E3**, and **E5**-**E7** were prepared by using the same procedure. Preparation of **E4**: To a solution of phospholipid S75 (60 mg), soybean oil (100 mg), and **PS9** (1.5 mg) in DCM, 3 mL of a deionized water solution containing 30 mg of F68 was added. After stirring for 4 h, the mixture was subjected to ultrasound treatment for 20 min and the DCM was allowed to evaporate. Then, the mixture was processed with an ultrasonic cell disruptor for an additional 5 min. **E4** was obtained by filtering through a 0.22 μm filter membrane, and **E8** was prepared using the same procedure.

### 4.5 Detection of the ROS Generation of E1

The ROS production of **E1** was evaluated by using DPBF and SOSG probes. **E1** (C_**PS9**_ = 1 μM) was mixed with DPBF (40 μM) in deionized water, and the absorbance changes at 415 nm were recorded under 660 nm irradiation at 0.5 W cm^–2^. A mixture of **E1** (C_**PS9**_ = 1 μM) and SOSG (3 μM) in deionized water was irradiated with a 660 nm laser (0.5 W cm^–2^) for varying durations, and the fluorescence spectra (λ_ex_/λ_em_ = 504/525 nm) were recorded immediately after irradiation. The type of generated ROS was further identified using EPR. TEMPO was chosen as a singlet oxygen scavenger. A 100 μM solution of **E1** in deionized water was irradiated with a 660 nm laser (0.5 W cm^–2^) for 1 min. TEMPO was then added to achieve a final concentration of 100 mM and mixed thoroughly for the EPR measurements.

### 4.6 Intracellular Uptake and Localization

A549, HepG2, MCF-7, BEAS-2B, and MCF-10A cells were seeded into 35 mm confocal dishes at a density of about 1.5 × 10^5^ cells per well and incubated overnight. For time-dependent uptake studies, the cells were incubated with **E1** for varying durations (0.5, 2, 6, and 12 h). Following incubation, the cells were washed three times with PBS and fixed with 4% PFA for 15 min. After staining with 200 μL of DAPI for 10 min, images were captured using a confocal laser scanning microscope (CLSM). For colocalization studies, the cells were incubated with **E1** for 6 h and then stained with DiI, MitoTracker Green, LysoTracker Green, or ER-Tracker Blue-White DPX. The cells were then washed three times with PBS, and fresh PBS was added before visualization with CLSM.

### 4.7 LDH Release Assays

A549 cells were seeded at a density of 1 × 10^4^ cells per well in a 96-well cell culture plate and cultured for 24 h. Following this, the cells were incubated with various emulsions for 6 h and then washed with 100 μL of fresh medium. The laser-treated group was irradiated with a 660 nm laser (0.5 W cm^–2^) for 10 min and allowed to grow for an additional 24 h. LDH release was measured according to the manufacturer’s instructions.

### 4.8 Intracellular LPO Detection

Intracellular LPO levels were assessed by using C11-BODIPY 581/591 as a probe. A549 cells were seeded in 35 mm confocal dishes at 37 °C for 24 h and incubated with **E1** for 6 h. After exposure to a 660 nm laser (0.5 W cm^–2^, 10 min), the cells were cultured for an additional 12 h and then stained with C11-BODIPY 581/591 and DAPI for 30 min. Images were captured using CLSM. The production of MDA was evaluated using an MDA Assay Kit. A549 cells were plated onto a 6-well plate and incubated at 37 °C for 24 h. After incubation with different emulsions for 6 h and washing with 1 mL of fresh medium, the laser-treated group was irradiated with a 660 nm laser (0.5 W cm^–2^, 10 min) and allowed to continue growing for 24 h. Intracellular LPO levels were measured according to the manufacturer’s instructions.

### 4.9 In Vitro ^19^F MRI

The MRI phantom study was conducted on a 9.4T scanner (Bruker) using the RARE (rapid acquisition with refocused echoes) sequence. Phantom samples with varying concentrations of **PS9** in CHCl_3_ (80, 40, 20, 10, 5, and 2.5 mM) were prepared. ^19^F MR images were acquired with the following parameters: center frequency = 376.527883 MHz, repetition time (TR) = 4000 ms, echo time (TE) = 3 ms, field of view (FOV) = 30 × 30 mm, slice thickness (SI) = 20 mm, matrix size = 32 × 32, RARE factor = 8, number of averages = 8, with a total acquisition time of 256 s.

### 4.10 In Vivo ^19^F MRI

For in vivo ^19^F MRI, mice bearing A549 tumors were intravenously injected with 100 μL of **E1** (C_**PS9**_ = 2.5 mg/kg, C_F_ = 6 mmol/kg). ^19^F MRI was performed using the RARE sequence with the following parameters: center frequency = 376.527883 MHz, TR = 4000 ms, TE = 3 ms, FOV = 37 × 37 mm, SI = 15 mm, matrix size = 32 × 32, RARE factor = 4, and number of averages = 64.

### 4.11 In Vivo Therapeutic Efficacy Evaluation

When the subcutaneous tumors reached approximately 100 mm^3^, the mice were randomly divided into four groups: (a) saline + L; (b) **PS9** (0.8 mg/kg) + L; (c) **E1** (C_**PS9**_ = 0.8 mg/kg, C_F_ = 1.8 mmol/kg); (d) **E1** (C_**PS9**_ = 0.8 mg/kg, C_F_ = 1.8 mmol/kg) + L. The mice received intravenous injections of **E1** on days 0, 4, and 8, followed by irradiation with a 660 nm laser (0.5 W cm^–2^, 10 min) 1 h post-injection. The body weight and tumor volume of mice were recorded every 2 days. After 20 days of treatment, the mice were euthanized, and the major organs and tumors were harvested for analysis, including H&E staining, *K*_i_-67 staining, and TUNEL staining.

### 4.12 Statistical Analysis

Data are presented as mean ± standard deviation from *n* ≥ 3 replicates. Asterisks indicate significant differences (*p < 0.05, **p < 0.01, ***p < 0.001) as determined by an unpaired Student’s two-sided *t*-test.

## Supporting Information Available

Additional experimental details, characterization, and photophysical data of **PS1**-**PS10**; formulation and characterization of **E1**-**E8**; and supplementary scheme, figures, tables, and results *in vitro/vivo* for this work (PDF)

## Abbreviations

PDT: Photodynamic therapy
PFBM: perfluoro-*tert*-butoxymethyl
PM: plasma membrane
FLI: fluorescence imaging
^19^F MRI: fluorine-19 magnetic resonance imaging
PTT: photothermal therapy
ACQ: aggregation-caused fluorescence quenching
SNR: signal-to-noise ratio
EPR: electron paramagnetic resonance
LDH: lactate dehydrogenase
