# Supplementary material for: Fluorination of Aza-BODIPY for Cancer Cell Plasma Membrane-Targeted Imaging and Therapy
Source: ACS Appl Mater Interfaces. 2025 Jan 2;17(2):3013–25. doi: 10.1021/acsami.4c17943 (PMC11744507; doi:10.1021/acsami.4c17943)
Supplement: Supplementary file 1 — am4c17943_si_001.pdf [file am4c17943_si_001.pdf]

Supporting Information

Fluorination of Aza-BODIPY for Cancer Cell Plasma Membrane-Targeted Imaging and Therapy

Anfeng Li,[a§] Fang Wang[a§] Yu Li,[a§] Xingxing Peng,[a] Yanqi Liu,[a] Lijun Zhu,[a] Pei He,[a] Tingting Yu,[a] Daiqin Chen,[a,b] Mojie Duan,[c] Xin Zhou,[a,b] Zhong-Xing Jiang,[a,b] and Shizhen Chen,[a,b]**

[a] State Key Laboratory of Magnetic Resonance and Atomic and Molecular Physics, National Center for Magnetic Resonance in Wuhan, Wuhan Institute of Physics and Mathematics, Innovation Academy for Precision Measurement Science and Technology, Chinese Academy of Sciences-Wuhan National Laboratory for Optoelectronics, Huazhong University of Science and Technology, Wuhan 430071, China.

[b] University of Chinese Academy of Sciences, Beijing 100049, China

[c] Interdisciplinary Institute of NMR and Molecular Sciences, School of Chemistry and Chemical Engineering, The State Key Laboratory of Refractories and Metallurgy, Wuhan University of Science and Technology, Wuhan 430081, China

[*] Email: zxjiang@apm.ac.cn, chenshizhen@apm.ac.cn

[§] A.L., F.W., and Y.L. contributed equally to this work

Table of Contents

1. General information S3
2. Reagents and materials S3
3. Supplementary Figures, Scheme, and Tables S4
4. Synthesis, characterization, and properties of compounds S19
   4.1 Synthesis of compounds S19
   4.2 FL quantum yield measurements S30
   4.3 Photothermal properties measurement of PSs S31
   4.4 Detection of ROS generation of PSs S31
   4.5 In vitro ^{19}F MRI S31
5. Preparation, characterization, and properties of **E1-E8** S31
   5.1 Preparation of **E1-E8** S31
   5.2 Detection the ROS generation of **E1** S32
6. Cell culture and animal models S32
   6.1 Intracellular uptake and localization S32
   6.2 In vitro cytotoxicity assays of **E1** S33
   6.3 Live/dead cell staining assays of **E1** S33
   6.4 Intracellular ROS imaging in A549 cells of **E1** S33
   6.5 In vitro endocytosis pathway study of **E1** S33
   6.6 LDH release assays S33
   6.7 Intracellular lipid peroxidation (LPO) detection S33
   6.8 Evaluation of glucose-regulated protein 78 (GRP78) S34
   6.9 In vivo ^{19}F MRI S34
   6.10 In vivo therapeutic efficacy evaluation S34
7. Statistical analysis S34
8. ^{1}H/^{19}F/^{13}C NMR and HRMS spectra of compounds S35
9. References S97

1. General information

^{1}H, ^{13}C, and ^{19}F NMR spectra were recorded on a Bruker 400 MHz, 500 MHz, or 600 MHz. ^{1}H NMR spectra were referenced to tetramethylsilane (s, 0.00 ppm) using $CDCl_3$ or tetrahydrofuran-d_8 as solvent. ^{13}C NMR spectra were referenced to solvent carbons (77.16 ppm for $CDCl_3$, 67.21 ppm, and 25.31 ppm for tetrahydrofuran-d_8). ^{19}F NMR spectra were referenced to 2% hexafluorobenzene (s, -164.90 ppm) in $CDCl_3$. The splitting patterns for ^{1}H NMR and ^{19}F NMR spectra were denoted as follows: s = singlet, d = doublet, t = triplet, q = quartet, dd = double doublet, m = multiplet. High-resolution mass spectra were recorded on a 4.7 Tesla FT-MS using electron spray ionization or electron ionization. MALDI-TOF mass spectra were recorded on a Bruker Ultraflex III TOF/TOF spectrometer. Flash chromatography was performed on 200-300 mesh silica gel with ethyl acetate/petroleum ether as eluent. The UV-Vis absorption and fluorescence emission spectra were measured using a UV-2600 UV-Vis spectrophotometer (Shimadzu, Japan) and an F-4700 spectrofluorophotometer (Hitachi, Japan), respectively. The average particle size and PDI of nanoemulsions were measured by DLS with Zetasizer of Malvern (Malvern, Nano ZS 90, UK). The morphology of the nanoemulsion was observed by TEM (JEM-2100, JEOL). A 660 nm laser was used for photothermal conversion and ROS generation experiments. Confocal laser scanning microscopy imaging was performed on a Nikon confocal laser scanning microscope (CLSM). ^{19}F MRI was performed on a Bruker BioSpec 9.4T MRI system. The temperature of the magnet room was maintained at 24 °C throughout the MRI experiment. Small animal fluorescence imaging was performed using the IVIS imaging system (PerkinElmer, United States). Photothermal imaging was measured with a thermal imaging camera (Zhejiang Dali Technology, China).

2. Reagents and materials

Phospholipid S75 was purchased from Lipoid GmbH (Germany). Pluronic F68 (average MW = 8350 Da.) was purchased from Adamas (Shanghai, China). Medicinal-grade soybean oil was purchased from Aladdin (Shanghai, China). DAFH-DA and SOSG were purchased from Thermo Fisher (USA). CCK-8, cell plasma membrane staining kit with DiI, DAPI, Calcein/PI cell viability/cytotoxicity assay kit, LDH assay kit, lipid peroxidation MDA assay kit, 4% paraformaldehyde fix solution (4% PFA), immunol staining blocking buffer, Mito-Tracker green, Lyso-Tracker Green, rabbit polyclonal GRP78 antibody, and Alexa Fluor 488-labeled goat anti-rabbit IgG(H+L) were all purchased from Beyotime (Shanghai, China). ER-Tracker Blue-White DPX was purchased from Solarbio (Beijing, China). Recombinant anti-cleaved N-terminal GSDMD antibody [EPR20829-408] was purchased from Abacm (USA).

3. Supplementary Figures, Scheme, and Tables

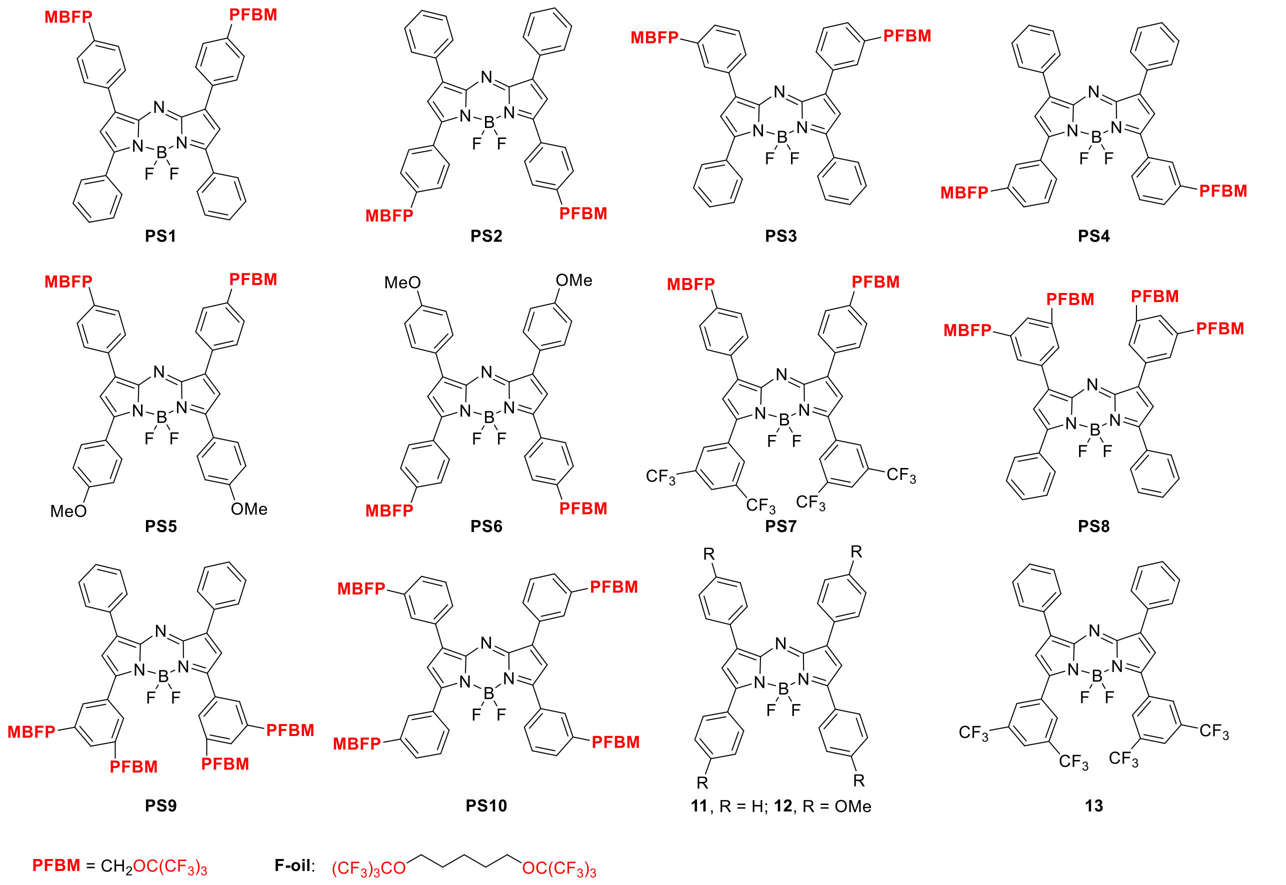

Figure S1. Structures of **PS1-PS10**, **11-13**, and **F-oil**.

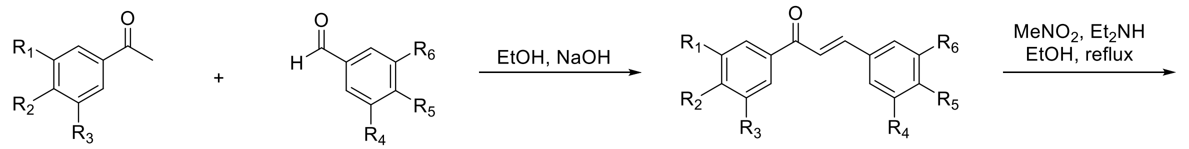

B1: R_1=R_3=H, R_2=PFBM
B2: R_1=PFBM, R_2=R_3=H
B3: R_1=R_3=PFBM, R_2=H
B4: R_1=R_2=R_3=H
B5: R_1=R_3=CF_3, R_2=H
B6: R_1=R_3=H, R_2=OMe

C1: R_4=R_6=H, R_5=PFBM
C2: R_4=PFBM, R_5=R_6=H
C3: R_4=R_6=PFBM, R_5=H
C4: R_4=R_6=H, R_5=OMe
C5: R_4=R_5=R_6=H

1a: R_1=R_2=R_3=R_4=R_6=H, R_5=PFBM, 86% from **B4** & **C1**
2a: R_1=R_3=R_4=R_5=R_6=H, R_2=PFBM, 78% from **B1** & **C5**
3a: R_1=R_2=R_3=R_5=R_6=H, R_4=PFBM, 70% from **B4** & **C2**
4a: R_2=R_3=R_4=R_5=R_6=H, R_1=PFBM, 44% from **B2** & **C5**
5a: R_1=R_3=R_4=R_6=H, R_2=OMe, R_5=PFBM, 91% from **B6** & **C1**
6a: R_1=R_3=R_4=R_6=H, R_2=PFBM, R_5=OMe, 53% from **B1** & **C4**
7a: R_1=R_3=CF_3, R_2=R_4=R_6=H, R_5=PFBM, 37% from **B5** & **C1**
8a: R_1=R_2=R_3=R_5=H, R_4=R_6=PFBM, 68% from **B4** & **C3**
9a: R_2=R_4=R_5=R_6=H, R_1=R_3=PFBM, 22% from **B3** & **C5**
10a: R_2=R_3=R_5=R_6=H, R_1=R_4=PFBM, 57% from **B2** & **C2**
11a: R_1=R_2=R_3=R_4=R_5=R_6=H, 73% from **B4** & **C5**
12a: R_1=R_3=R_4=R_6=H, R_2=R_5=OMe,84% from **B6** & **C4**
13a: R_1=R_3=CF_3, R_2=R_4=R_5=R_6=H, 63% from **B5** & **C5**

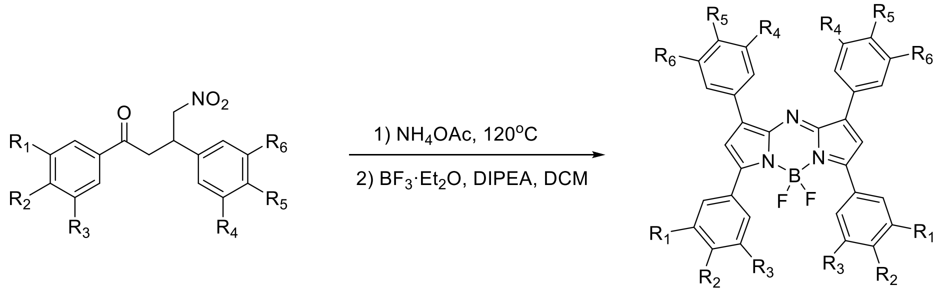

1b: R_1=R_2=R_3=R_4=R_6=H, R_5=PFBM, 94% from **1a**
2b: R_1=R_3=R_4=R_5=R_6=H, R_2=PFBM, 94% from **2a**
3b: R_1=R_2=R_3=R_5=R_6=H, R_4=PFBM, 90% from **3a**
4b: R_2=R_3=R_4=R_5=R_6=H, R_1=PFBM, 76% from **4a**
5b: R_1=R_3=R_4=R_6=H, R_2=OMe, R_5=PFBM, 94% from **5a**
6b: R_1=R_3=R_4=R_6=H, R_2=PFBM, R_5=OMe, 93% from **6a**
7b: R_1=R_3=CF_3, R_2=R_4=R_6=H, R_5=PFBM, 69% from **7a**
8b: R_1=R_2=R_3=R_5=H, R_4=R_6=PFBM, 66% from **8a**
9b: R_2=R_4=R_5=R_6=H, R_1=R_3=PFBM, 81% from **9a**
10b: R_2=R_3=R_5=R_6=H, R_1=R_4=PFBM, 72% from **10a**
11b: R_1=R_2=R_3=R_4=R_5=R_6=H, 81% from **11a**
12b: R_1=R_3=R_4=R_6=H, R_2=R_5=OMe,97% from **12a**
13b: R_1=R_3=CF_3, R_2=R_4=R_5=R_6=H, 64% from **13a**

PS1: R_1=R_2=R_3=R_4=R_6=H, R_5=PFBM, 10% from **1b**
PS2: R_1=R_3=R_4=R_5=R_6=H, R_2=PFBM, 2% from **2b**
PS3: R_1=R_2=R_3=R_5=R_6=H, R_4=PFBM, 13% from **3b**
PS4: R_2=R_3=R_4=R_5=R_6=H, R_1=PFBM, 15% from **4b**
PS5: R_1=R_3=R_4=R_6=H, R_2=OMe, R_5=PFBM, 5% from **5b**
PS6: R_1=R_3=R_4=R_6=H, R_2=PFBM, R_5=OMe, 3% from **6b**
PS7: R_1=R_3=CF_3, R_2=R_4=R_6=H, R_5=PFBM, 3% from **7b**
PS8: R_1=R_2=R_3=R_5=H, R_4=R_6=PFBM, 13% from **8b**
PS9: R_2=R_4=R_5=R_6=H, R_1=R_3=PFBM, 20% from **9b**
PS10: R_2=R_3=R_5=R_6=H, R_1=R_4=PFBM, 5% from **10b**
11: R_1=R_2=R_3=R_4=R_5=R_6=H, 22% from **11b**
12: R_1=R_3=R_4=R_6=H, R_2=R_5=OMe, 28% from **12b**
13: R_1=R_3=CF_3, R_2=R_4=R_5=R_6=H, 16% from **13b**

Scheme S1. Synthesis of **PS1-PS10** and **11-13**.

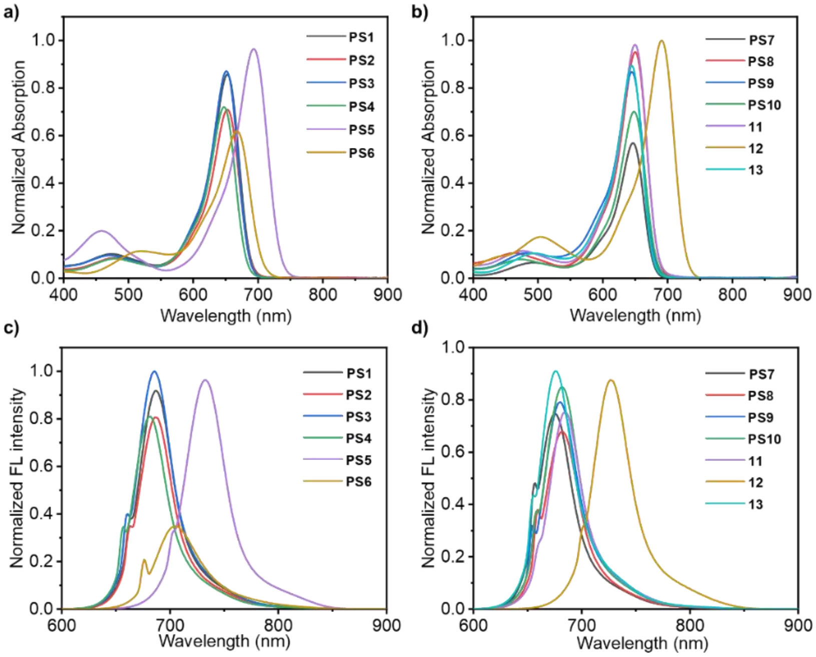

Figure S2. Normalized UV-Vis absorption (a, b) and FL emission (c, d) spectra of **PS1-PS10** and **11-13** (10 μM in $CHCl_3$).

Table S1. Photophysical properties of **PS1-PS10** and **11-13** in $CHCl_3$.

	$\lambda_{abs}{}^{a}$ (nm)	$\lambda_{Ex}{}^{b}$ (nm)	$\lambda_{Em}{}^{c}$ (nm)	Stokes (nm)	ε ($M^{-1}cm^{-1}$)	ϕ_f	ϕ_Δ
**PS1**	653	653	687	34	80580	0.33	0.08
**PS2**	653	653	686	33	66730	0.31	0.10
**PS3**	651	651	686	35	82700	0.28	0.10
**PS4**	647	647	682	35	63780	0.21	0.12
**PS5**	693	693	732	39	91560	0.37	0.02
**PS6**	667	667	704	37	56450	0.11	0.15
**PS7**	647	647	675	28	54860	0.36	0.47
**PS8**	650	650	682	32	90670	0.22	0.05
**PS9**	645	645	680	35	81130	0.26	0.22
**PS10**	648	648	682	34	66610	0.23	0.11
**11**	650	650	685	35	86520	0.34*	0.10
**12**	691	691	727	36	93720	0.20	0.05
**13**	645	645	676	31	86250	0.37	0.72

a: The maximum absorption wavelength. *b*: Excitation wavelength. *c*: The maximum emission wavelength. ε: Molar absorption coefficient. ϕ_f: Fluorescence quantum yield. ϕ_Δ: Singlet oxygen quantum yield. *literature data presented in parentheses.[1]

Table S2. The dihedral angles (α, β, χ, δ, shown in Figure S6) between the peripheral phenyl groups and the core of **PS1-PS10** and **11-13**.

	α	β	χ	δ
**PS1**	21.912	22.977	28.689	28.472
**PS2**	22.526	22.864	28.223	29.306
**PS3**	22.625	20.089	27.843	27.484
**PS4**	20.166	19.565	25.374	25.184
**PS5**	24.057	24.264	27.509	27.397
**PS6**	22.338	20.080	29.048	29.435
**PS7**	21.365	23.427	24.180	23.947
**PS8**	27.056	19.437	26.910	27.266
**PS9**	21.301	20.204	24.109	25.751
**PS10**	19.214	24.690	30.295	27.834
**11**	23.855	23.869	26.083	25.981
**12**	22.410	22.412	26.855	26.852
**13**	23.783	23.788	28.965	28.965

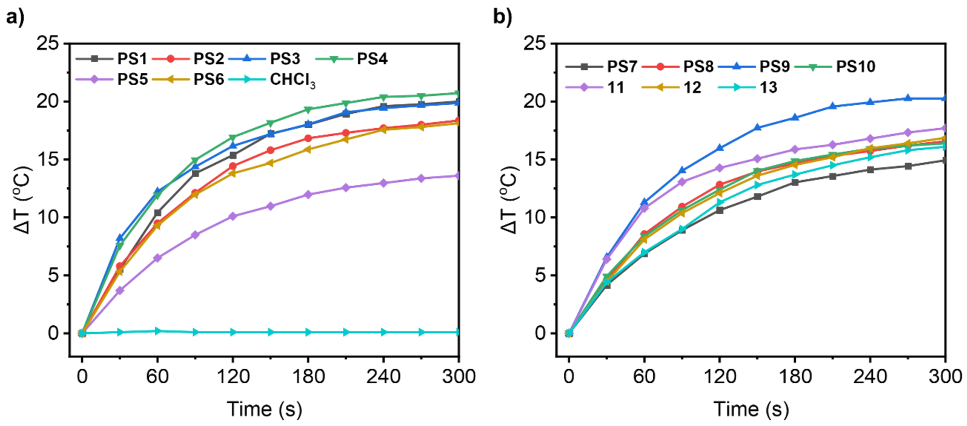

Figure S3. Temperature changes of 20 μM **PS1-PS6** (a), **PS7-PS10** and **11-13** (b) in $CHCl_3$ under the irradiation of 660 nm laser at 0.5 Wcm^{-2} for 5 min.

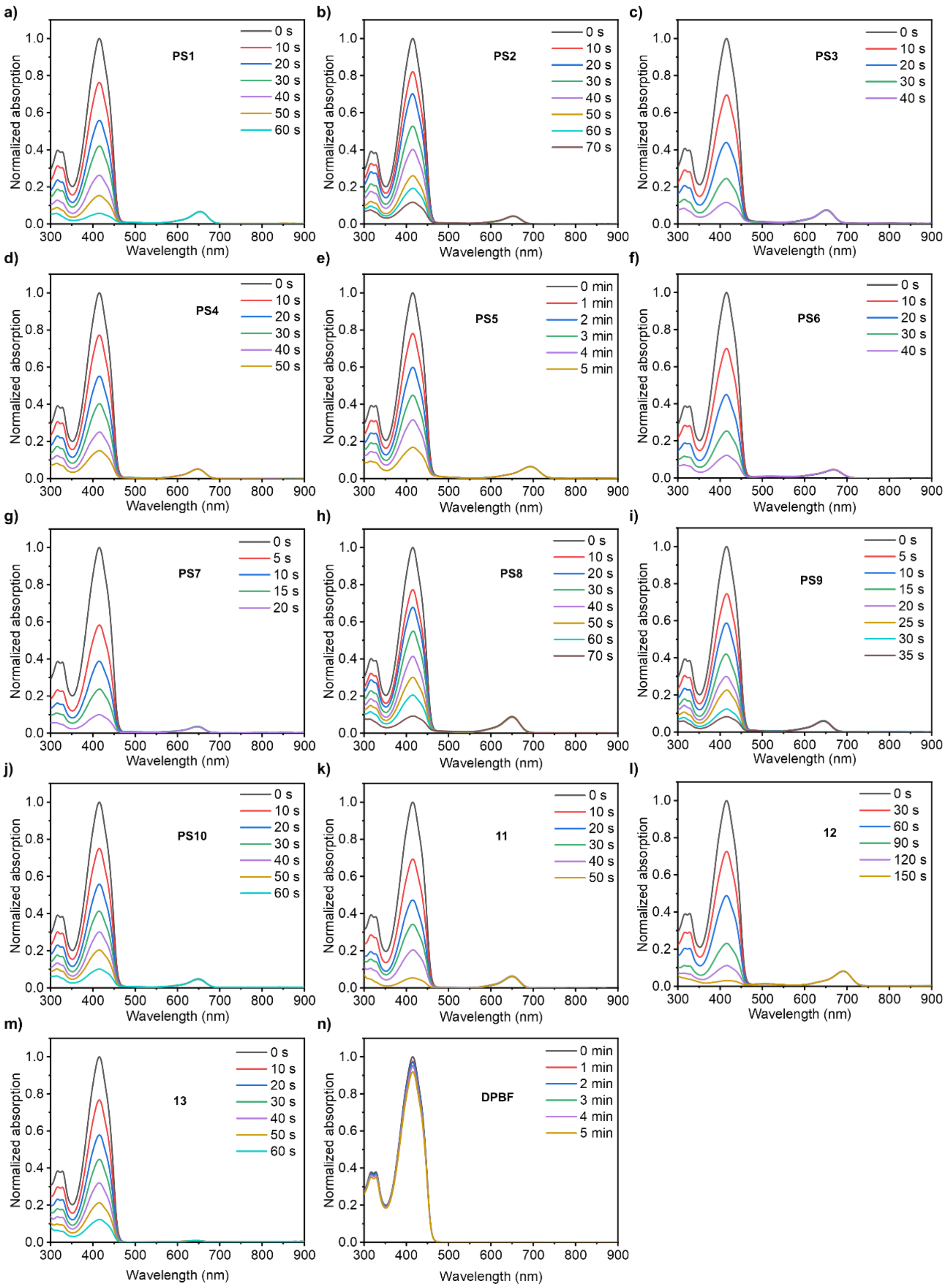

Figure S4. Time-dependent UV-Vis absorption spectra of DPBF (40 μM) with **PS1** (a, 1 μM), **PS2** (b, 1 μM), **PS3** (c, 1 μM), **PS4** (d, 1 μM), **PS5** (e, 1 μM), **PS6** (f, 1 μM), **PS7** (g, 1 μM), **PS8** (h, 1 μM), **PS9** (i, 1 μM), **PS10** (j, 1 μM), **11** (k, 1 μM), **12** (l, 1 μM), **13** (m, 0.1 μM), and without PS (n) respectively in $CHCl_3$ under the irradiation of 660 nm laser at 0.5 Wcm^{-2}.

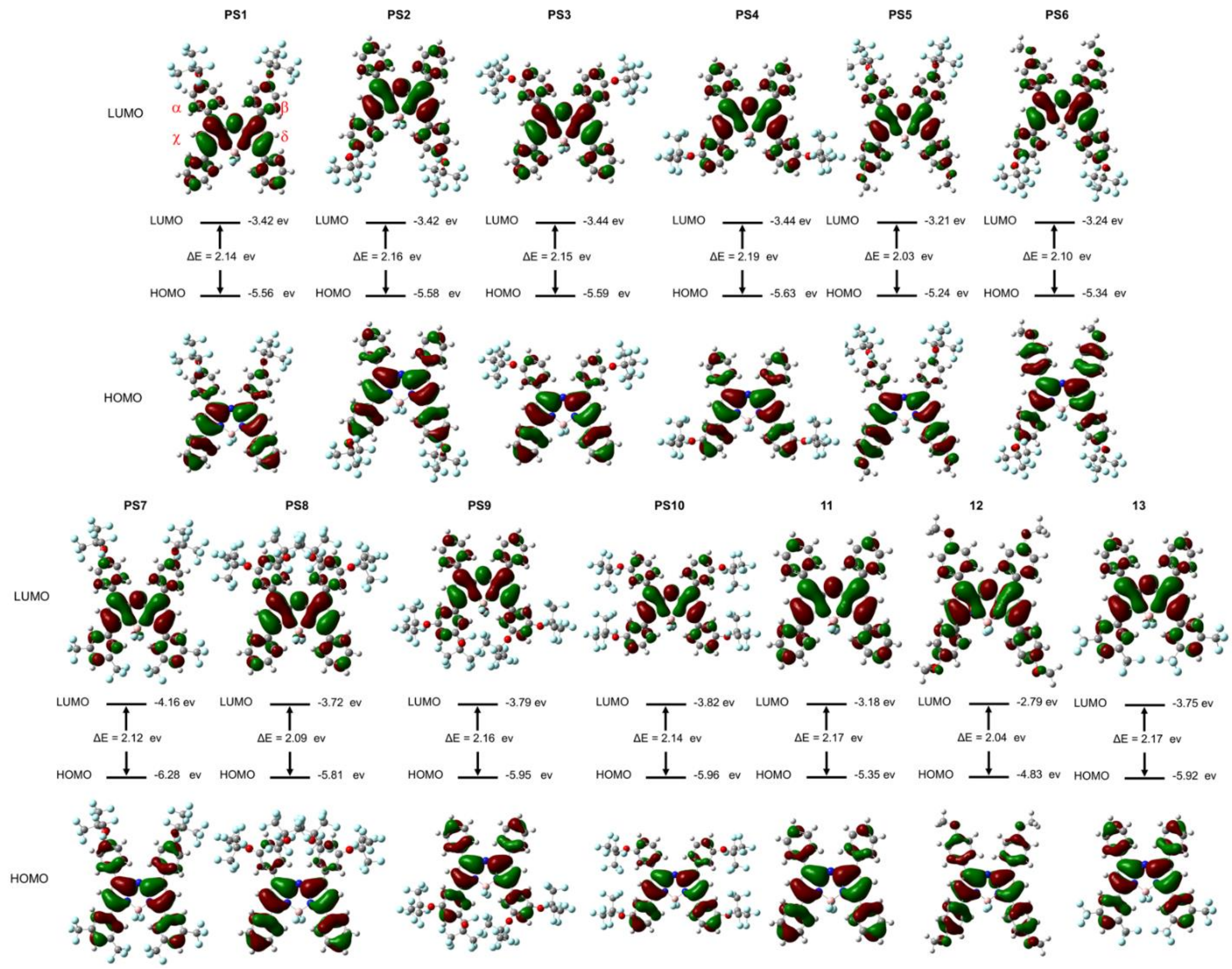

Figure S5. Calculated HOMO and LUMO of **PS1-PS10** and **11-13** by Gaussian 09.

Table S3. The relaxation times and T_2/T_1 ratios of **PS1-PS10**.

Comp.	PS1	PS2	PS3	PS4	PS5	PS6	PS7	PS8	PS9	PS10
T_1 (s)	1.33	1.35	1.37	1.41	1.28	1.28	1.30	1.09	1.23	1.31
T_2 (s)	1.04	1.04	1.08	1.19	1.19	0.96	1.09	0.87	0.88	1.05
T_2/T_1	0.78	0.77	0.78	0.84	0.93	0.75	0.84	0.80	0.72	0.80

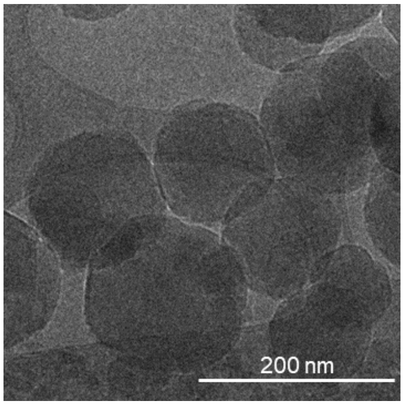

Figure S6. Cryo-EM image of **E1** (scale bar: 200 nm).

Table S4. Formulation ingredients, diameter, and PDI of **E1**-**E7**.

	S75	F68	PS	soybean oil or **F-oil**	Size (nm)	PDI
**E1**	60 mg	30 mg	**PS9** (2 mg)	**F-oil**	133	0.19
**E2**	60 mg	30 mg	None	**F-oil**	91	0.22
**E3**	60 mg	30 mg	**11** (1 mg)	**F-oil**	104	0.28
**E4**	60 mg	30 mg	**PS9** (1.5 mg)	soybean oil	171	0.14
**E5**	60 mg	30 mg	**PS4** (1.5 mg)	**F-oil**	83	0.28
**E6**	60 mg	30 mg	**PS8** (1.5 mg)	**F-oil**	153	0.30
**E7**	60 mg	30 mg	**PS10** (1.5 mg)	**F-oil**	148	0.23
**E8**	60 mg	30 mg	**PS9** (1 mg) **BODIPY**493/503 (0.5 mg)	**F-oil**	-	-

F-oil: 100 mg, soybean oil: 100 mg, H_2O: 3 mL.

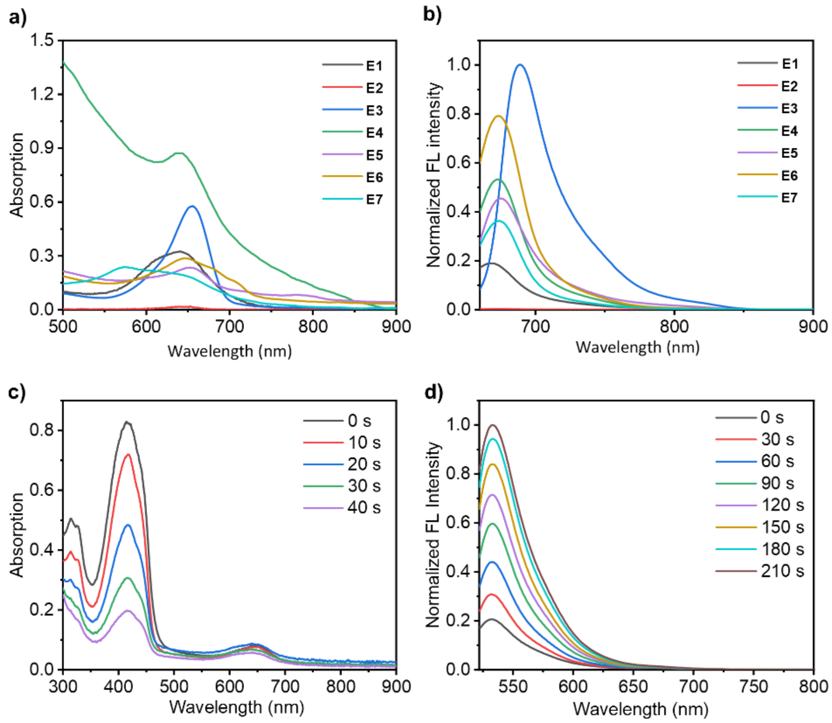

Figure S7. UV-Vis absorption (a) and Normalized FL emission spectra of **E1-E7** (b). Time-dependent UV-Vis absorption spectra of DPBF (40 µM) and **E1** (1 µM for **PS9**) solution (c) and the normalized FL intensity of **E1** and SOSG solution (d) under the irradiation of 660 nm laser at 0.5 Wcm^{-2}.

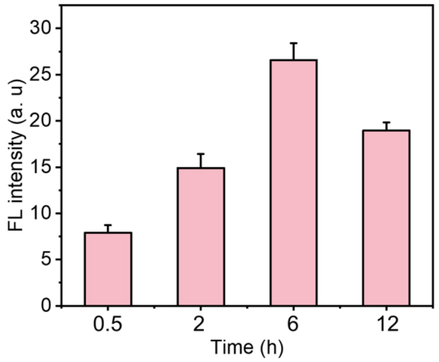

Figure S8. Quantitative analysis of FL intensity of **E1**-treated A549 cells at different incubating times.

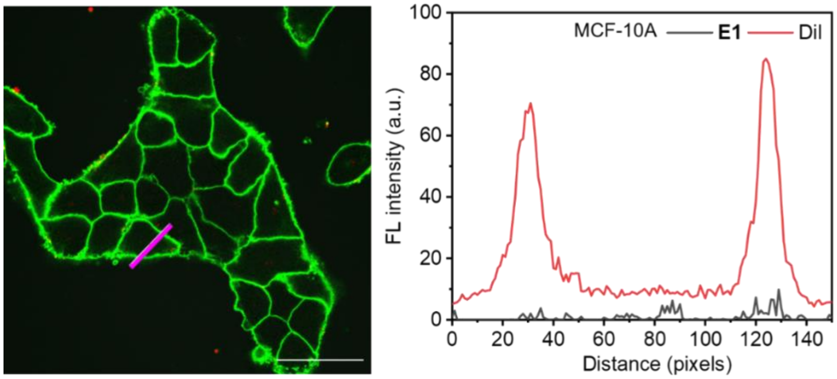

Figure S9. The line-plot graphs of the fluorescence intensity profiles of DiI and **PS9** in **E1**-treated MCF-10A cells along the magenta lines in the CLSM images. Scale bars: 50 μm.

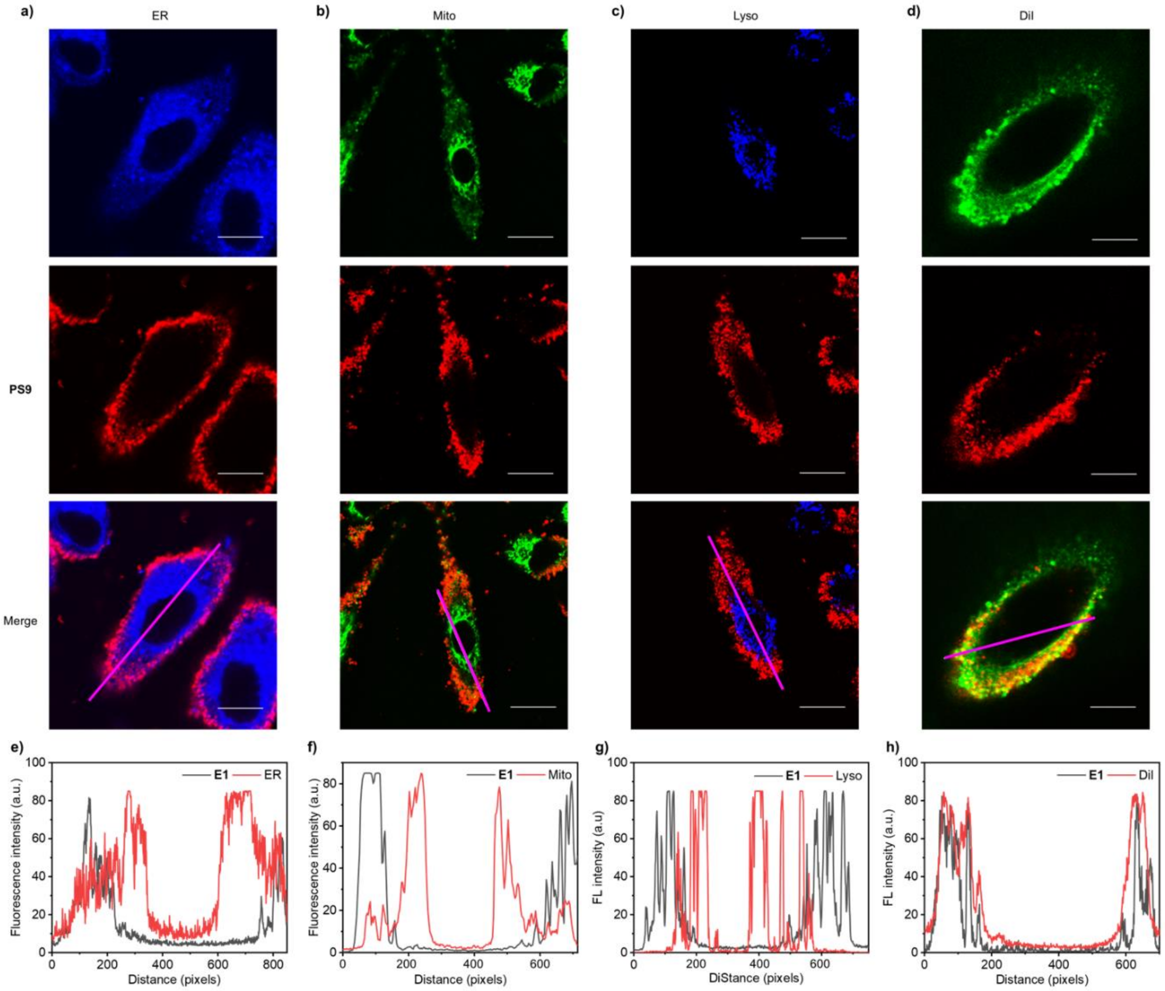

Figure S10. Colocalization images of **PS9** with ER-Tracker (a), Mito-Tracker (b), Lyso-Tracker (c), and DiI (d) in **E1**-treated A549 cells. The line-plot graphs of the fluorescence intensity profiles of **PS9** with the ER-Tracker (e), the Mito-Tracker (f), the Lyso-Tracker(g), and the DiI (h) along the magenta lines in the CLSM images. Scale bars: 10 μm.

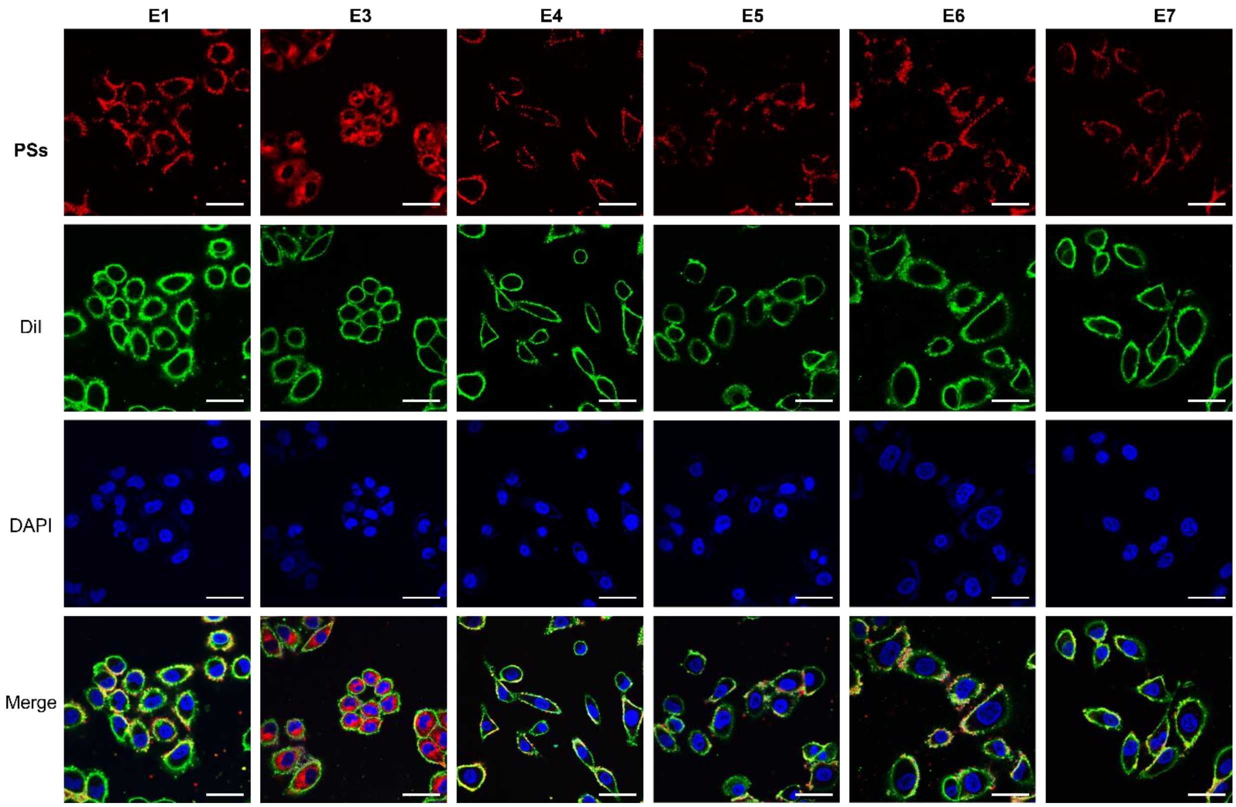

Figure S11. Confocal microscopy images of A549 cells treated with **E1**, **E3** - **E7**. Scale bars: 50 μm.

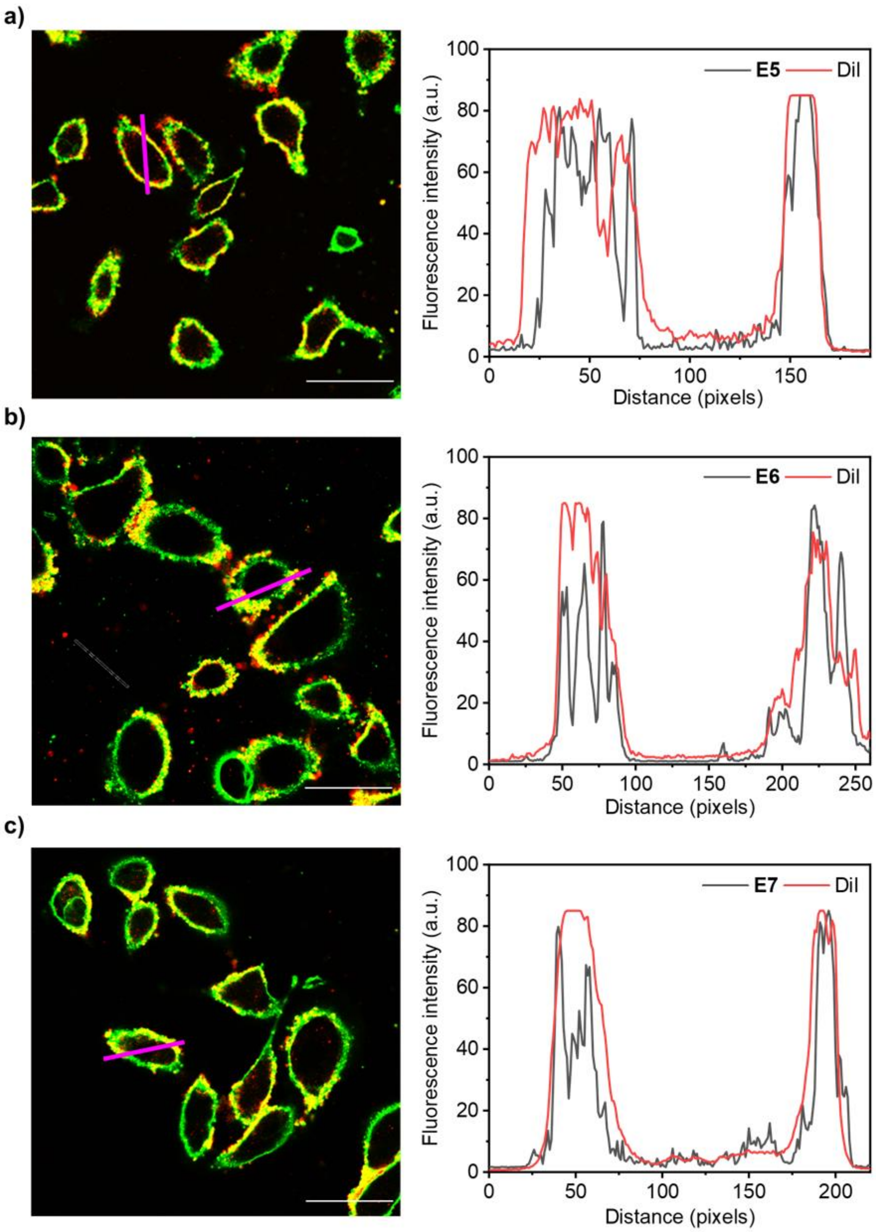

Figure S12. The line-plot graphs of the fluorescence intensity profiles of DiI and **E5** (a), **E6** (b), and **E7** (c) in A549 cells along the magenta lines in the CLSM images. Scale bars: 50 μm.

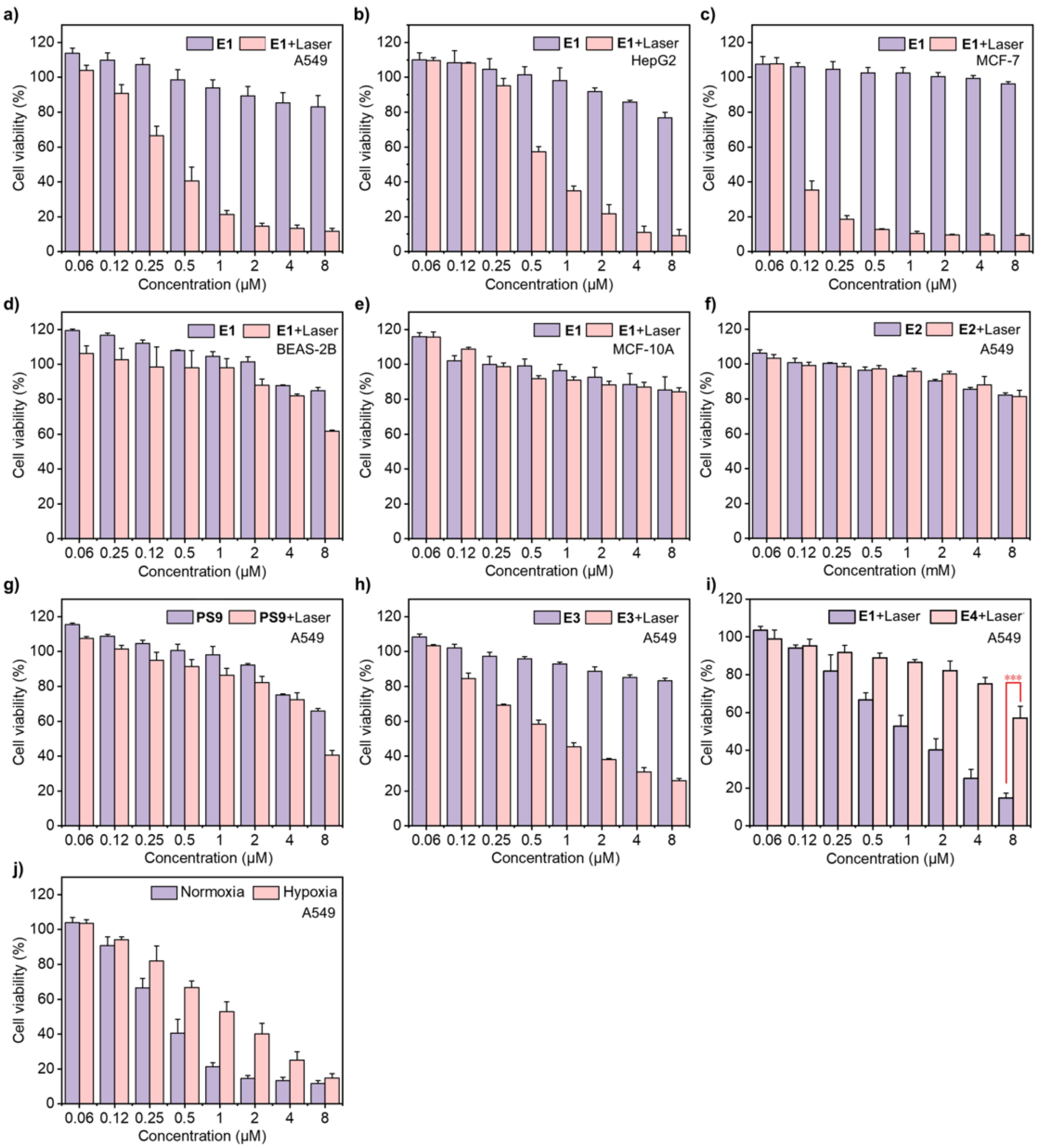

Figure S13. Cytotoxicity assay of **E1** against A549 (a), HepG2 (b), MCF-7 (c), BEAS-2B (d), and MCF-10A (e) cells. Cytotoxicity assay of **E2** (f), unformulated **PS9** (g) and **E3** (h) against A549 cell in the dark or after laser irradiation. Cytotoxicity assay of **E1** and **E4** against A549 cell after laser irradiation under hypoxic conditions (i). Cytotoxicity assay of **E1** against A549 cell after laser irradiation under normoxic and hypoxic conditions (j). Data were presented as mean ± standard deviation (n = 3, asterisks indicate statistical significance, ***$p < 0.001$).

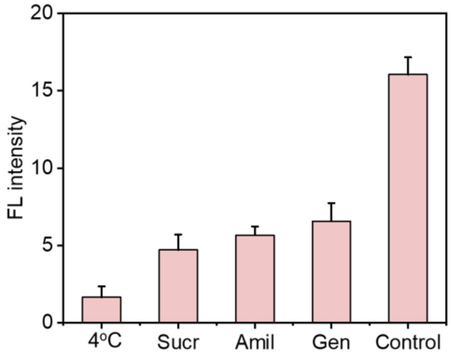

Figure S14. Quantitative analysis of FL intensity of **PS9** in A549 cells incubated with nanoemulsion **E1** and endocytosis inhibitors or decrease the culture temperature to 4 °C.

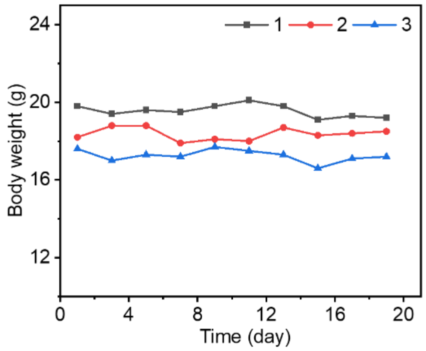

Figure S15. Body weight curves of 3 BALB/c nude mice receiving intravenous injection of **E1**.

Table S5. Blood biochemical analysis of **E1**-treated mice and controls (PBS-treated).

Group	Reference values	Control	Control	Control	**E1**	**E1**	**E1**
WBC (10^9/L)	0.8-6.8	2.7	5.5	2.8	6.3	1.9	1.7
RBC (10^{12}/L)	6.36-9.42	7.58	7.94	9.26	7.76	7.90	7.77
HGB (g/L)	110-143	124	113	133	124	123	130
MCH (pg)	15.8-19	16.0	16.6	16.7	16.4	16.2	16.5
PLT (10^9/L)	450-1590	543	520	565	482	473	491

The tested indexes include the number of white blood cells (WBC), number of red blood cells (RBC), concentration of hemoglobin (HGB), mean corpuscular hemoglobin (MCH), and number of blood platelets (PLT).

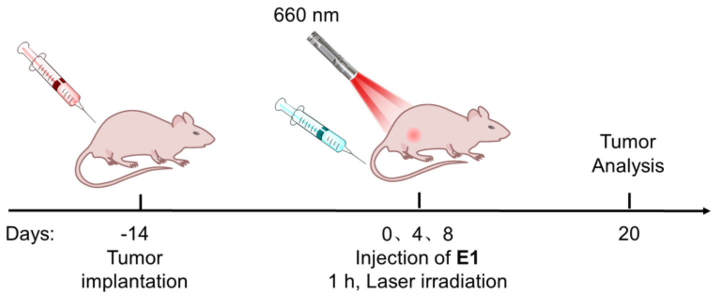

Figure S16. Schematic diagram of the treatment schedules.

4. Synthesis, characterization, and properties of compounds

4.1 Synthesis of compounds

1-(4-(Bromomethyl)phenyl)ethan-1-one (A1): Under an argon atmosphere, 4-methylacetophenone (10.00 g, 74.53 mmol), NBS (16.58 g, 93.16 mmol), and AIBN (2.45 g, 14.92 mmol) were dissolved in anhydrous acetonitrile. The mixture was refluxed for 4 h. After cooling to room temperature, the mixture was washed with water, extracted with dichloromethane (DCM), and concentrated under vacuum to yield the crude product. The crude product was purified by flash chromatography, yielding compound **A1** as a white solid (15.46 g, 97%). **^{1}H NMR** (400 MHz, $CDCl_3$) δ 7.93 (d, *J* = 8.3 Hz, 2H), 7.48 (d, *J* = 8.3 Hz, 2H), 4.50 (s, 2H), 2.60 (s, 3H).

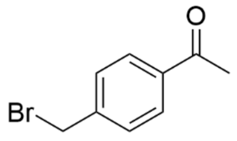

1-(3-(Bromomethyl)phenyl)ethan-1-one (A2). Following the same procedure as for **A1**, compound **A2** was obtained as a white solid in 69% yield (220.30 mg) from 3-methyl acetophenone (200.00 mg, 1.49 mmol). **^{1}H NMR** (500 MHz, $CDCl_3$) δ 7.96 (s, 1H), 7.86 (d, *J* = 7.8 Hz, 1H), 7.58 (d, *J* = 7.8 Hz, 1H), 7.43 (t, *J* = 7.8 Hz, 1H), 4.51 (s, 2H), 2.59 (s, 3H).

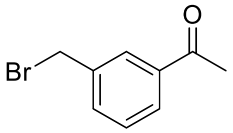

1-(3,5-Bis(bromomethyl)phenyl)ethan-1-one (A3). Following the same procedure as for **A1**, compound **A3** was obtained as a white solid in a 48% yield (5.01 g) from 3,5-dimethylacetophenone (5.00 g, 33.7 mmol). **^{1}H NMR** (500 MHz, $CDCl_3$) δ 7.89 (s, 2H), 7.62 (s, 1H), 4.51 (s, 4H), 2.61 (s, 3H).

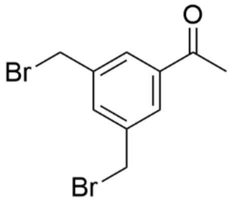

3,5-Bis(bromomethyl)benzyl acetate (A4). 1,3,5- tribromomethylbenzene (6.00 g, 16.81 mmol), glacial acetic acid (1.51 g, 25.22mmol), and diisopropylethylamine (DIPEA, 6.52 g, 50.44 mmol) were dissolved in acetonitrile and stirred at room temperature for 24 h. The mixture was then washed with saturated brine, extracted with DCM, dried over anhydrous Na_2SO_4, and evaporated under vacuum to yield the crude product, which was purified by flash chromatography to afford compound **A4** as a white solid (1.83 g, 32%). **^{1}H NMR** (500 MHz, $CDCl_3$) δ 7.38 (s, 1H), 7.31 (s, 2H), 5.08 (s, 2H), 4.46 (s, 4H), 2.12 (s, 3H).

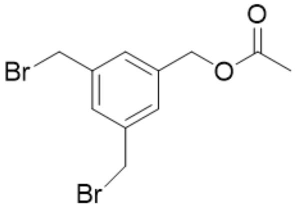

3,5-Bis(perfluoro-*tert*-butoxymethyl)benzyl acetate (A5). Under an argon atmosphere, compound **A4** (2.08, 6.19 mmol) and potassium perfluoro-*tert*-butoxide (3.74 g, 13.62 mmol) were dissolved in dry DMF and stirred at room temperature for 24 h. The mixture was then washed with water and extracted three times using EtOAc. The combined organic layer was dried over anhydrous Na_2SO_4 and evaporated under vacuum to yield the crude product, which was purified by flash chromatography to afford compound **A5** as a white solid (3.91 g, 99% yield). **^{1}H NMR** (500 MHz, $CDCl_3$) δ 7.32 (s, 1H), 7.30 (s, 2H), 5.13 (s, 2H), 5.07 (s, 4H), 2.12 (s, 3H). **^{19}F NMR** (471

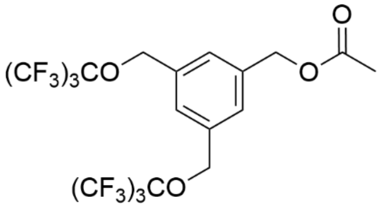

MHz, $CDCl_3$) δ -73.34 (s). **^{13}C NMR** (126 MHz, $CDCl_3$) δ 170.8, 137.3, 136.1, 127.3, 126.2, 120.4 (q, *J* = 293.1 Hz), 81.1 – 79.1 (m), 70.6, 65.5, 20.9. **HRMS** (EI) m/z: $[M]^+$ calcd for $C_{19}H_{12}F_{18}O_4^+$: 646.0443, found 646.0458.

(3,5-Bis(perfluoro-*tert*-butoxymethyl)phenyl)methanol (A6). To a solution of compound **A5** (3.91 g, 6.05 mmol) in methanol, a solution of sodium hydroxide (0.96 g, 24.00 mmol) in water was added. The mixture was stirred for 24 h and then neutralized with 2 N hydrochloric acid. The solution was extracted with DCM, dried over anhydrous Na_2SO_4, and purified by column chromatography to yield **A6** as a white solid (3.29 g, 90%). **^{1}H NMR** (600 MHz, $CDCl_3$) δ 7.33 (s, 2H), 7.28 (s, 1H), 5.06 (s, 4H), 4.74 (s, 2H). **^{19}F NMR** (471 MHz, $CDCl_3$) δ -73.23 (s).**^{13}C NMR** (126 MHz, $CDCl_3$) δ 142.1, 135.9, 126.3, 125.9, 120.4 (q, *J* = 292.8 Hz), 80.6 – 79.4 (m), 70.8, 64.7. **HRMS** (EI) m/z: $[M]^+$ calcd for $C_{17}H_{10}F_{18}O_3^+$: 604.0337, found 604.0349.

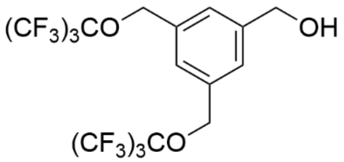

1-(4-(Perfluoro-*tert*-butoxymethyl)phenyl)ethan-1-one (B1). Under an argon atmosphere, compound **A1** (8.85 g, 41.54 mmol) and potassium perfluoro-*tert*-butoxide (12.53 g, 45.71 mmol) were dissolved in dry DMF and stirred at room temperature for 24 h. The mixture was then washed with water and extracted three times using EtOAc. The combined organic layer was dried over anhydrous Na_2SO_4 and evaporated under vacuum to yield the crude product, which was purified by flash chromatography to afford compound **B1** as a white solid (9.98 g, yield 65%). **^{1}H NMR** (400 MHz, $CDCl_3$) δ 7.98 (d, *J* = 8.2 Hz, 2H), 7.44 (d, *J* = 8.2 Hz, 2H), 5.10 (s, 2H), 2.61 (s, 3H). **^{19}F NMR** (376 MHz, $CDCl_3$) δ -73.33 (s). **^{13}C NMR** (126 MHz, $CDCl_3$) δ 197.6, 139.9, 137.5, 128.9, 127.5, 120.5 (q, *J* = 293.6 Hz), 80.9 – 79.0 (m), 70.6, 26.8. **HRMS** (MALDI-TOF) m/z: $[M+H]^+$ calcd for $C_{13}H_{10}F_9O_2^+$: 369.0531, found 369.0543.

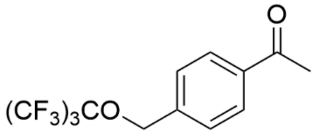

1-(3-(Perfluoro-*tert*-butoxymethyl)phenyl)ethan-1-one (B2). Following the same procedure as for **B1**, **B2** was prepared as a white solid in 66% yield (4.71 g) from **A2** (4.14 g, 19.43 mmol). **^{1}H NMR** (500 MHz, $CDCl_3$) δ 7.96 (d, *J* = 7.8 Hz, 1H), 7.92 (s, 1H), 7.57 (d, *J* = 7.8 Hz, 1H), 7.51 (t, *J* = 7.8 Hz, 2H), 5.10 (s, 2H), 2.62 (s, 3H). **^{19}F NMR** (471 MHz, $CDCl_3$) δ -73.27 (s). **^{13}C NMR** (126 MHz, $CDCl_3$) δ 197.6, 137.6, 135.5, 132.2, 129.2, 128.8, 127.5, 120.4 (q, *J* = 293.5 Hz), 80.4 – 79.3 (m), 70.8, 26.7. **HRMS** (EI) m/z: $[M]^+$ calcd for $C_{13}H_9F_9O_2^+$: 368.0453, found 368.0451.

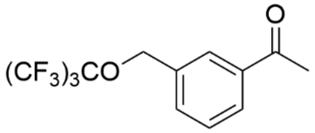

1-(3,5-Bis(perfluoro-*tert*-butoxymethyl)phenyl)ethan-1-one (B3). Following the same procedure as for **B1**, **B3** was prepared as a white solid in 85% yield (7.00 g) from A**3** (4.09 g, 13.37 mmol). **^{1}H NMR** (600 MHz, $CDCl_3$) δ 7.89 (s, 2H), 7.59 (s, 1H), 5.13 (s, 4H), 2.62 (s, 3H). **^{19}F NMR** (471 MHz, $CDCl_3$) δ -73.31 (s). **^{13}C NMR** (126 MHz, $CDCl_3$) δ 197.0, 138.0, 136.4, 130.6, 127.4, 120.4 (q, *J* = 292.5 Hz), 80.0 – 79.4 (m), 70.4, 26.6. **HRMS** (MALDI-TOF) m/z: $[M+H]^+$ calcd for $C_{18}H_{11}F_{18}O_3^+$: 617.0415, found 617.0418.

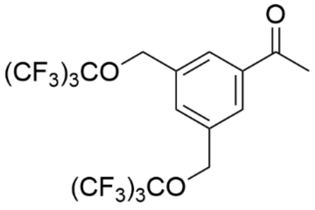

4-(Perfluoro-*tert*-butoxymethyl)benzaldehyde (C1). Following the same procedure as for **B1**, **C1** was prepared as a clear liquid in 91% yield (7.15 g) from 4-(bromomethyl) benzaldehyde (4.42 g, 22.21 mmol). **^{1}H NMR** (400 MHz, $CDCl_3$) δ 10.03 (s, 1H), 7.91 (d, *J* = 8.2 Hz, 2H), 7.51 (d, *J* = 8.2 Hz, 2H), 5.12 (s, 2H). **^{19}F NMR** (376 MHz, $CDCl_3$) δ -73.26 (s). **^{13}C NMR** (101 MHz, $CDCl_3$) δ 191.7, 141.3, 136.5, 130.0, 127.6, 120.3 (q, *J* = 293.7 Hz), 80.9 – 78.9 (m), 70.4. **HRMS** (EI) m/z: [M-H$^+$] calcd for $C_{13}H_9F_9O_2^+$: 353.0219, found 353.0216.

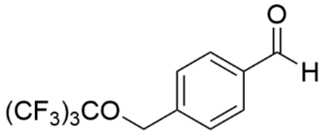

3-(Perfluoro-*tert*-butoxymethyl)benzaldehyde (C2). Following the same procedure as for **B1**, **C2** was prepared as a clear liquid in 95% yield (4.24 g) from 3-(bromomethyl) benzaldehyde (2.50 g, 12.56 mmol). **^{1}H NMR** (500 MHz, $CDCl_3$) δ 10.04 (s, 1H), 7.89 (d, *J* = 7.7 Hz, 1H), 7.85 (s, 1H), 7.63 (d, *J* = 7.7 Hz, 1H), 7.59 (t, *J* = 7.7 Hz, 1H), 5.12 (s, 2H). **^{19}F NMR** (471 MHz, $CDCl_3$) δ -73.18 (s). **^{13}C NMR** (126 MHz, $CDCl_3$) δ 191.8, 136.8, 136.0, 133.5, 130.2, 129.6, 128.6, 120.4 (q, *J* = 294.1 Hz), 81.9 – 78.2 (m), 70.5. **HRMS** (EI) m/z: [M-H$^+$] calcd for $C_{13}H_9F_9O_2^+$: 353.0219, found 353.0217.

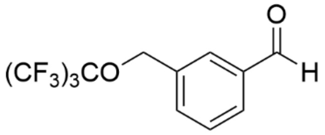

3,5-Bis(perfluoro-*tert*-butoxymethyl)benzaldehyde (C3). Under an argon atmosphere, compound **A6** (3.43 g, 5.68 mmol) and Dess-Martin periodinane (6.02 g, 14.19 mmol) were dissolved in dry DCM and stirred for 8 h at room temperature. Upon completion of the reaction, the mixture was washed with saturated Na_2CO_3 solution, extracted with DCM, dried over anhydrous Na_2SO_4, and purified by column chromatography after evaporating the organic solvent under vacuum to yield **C3** as a white solid (3.27 g, 95%).**^{1}H NMR** (500 MHz, $CDCl_3$) δ 10.05 (s, 1H), 7.83 (s, 2H), 7.65 (s, 1H), 5.15 (s, 4H). **^{19}F NMR** (471 MHz, $CDCl_3$) δ -73.23 (s). **^{13}C NMR** (126 MHz, $CDCl_3$) δ 191.1, 137.2, 136.9, 131.8, 128.6, 123.9 – 116.9 (q, *J* = 292.6 Hz), 83.6 – 78.8 (m), 70.1. **HRMS** (EI) m/z: [M]$^+$ calcd for $C_{17}H_8F_{18}O_3^+$: 602.0181, found 602.0189.

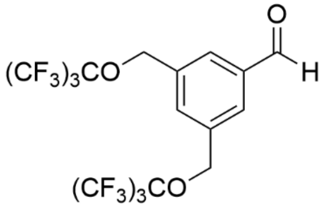

(*E*)-3-(4-(Perfluoro-*tert*-butoxymethyl)phenyl)-1-phenylprop-2-en-1-one (1a). Acetophenone **B4** (0.51 g 4.24 mmol) and KOH (0.48 g, 8.49 mmol) were dissolved in EtOH. The mixture was stirred for 5 min, and then **C1** (1.50 g, 4.24 mmol) was added in one portion. After stirring for 24 h at room temperature, the mixture was neutralized with 2 N HCl. After filtration, the residue was collected and re-dissolved in EtOAc. The solution was dried over anhydrous Na_2SO_4 and concentrated under vacuum to give the crude product, which was recrystallized from EtOAc and EtOH to give **1a** as a faint yellow solid (1.66 g, 86%). **^{1}H NMR** (400 MHz, $CDCl_3$) δ 8.06 – 8.01 (m, 2H), 7.81 (d, *J* = 15.7 Hz, 1H), 7.67 (d, *J* = 8.2 Hz, 2H), 7.63 – 7.47 (m, 4H), 7.40 (d, *J* = 8.2 Hz, 2H), 5.07 (s, 2H). **^{19}F NMR** (376 MHz, $CDCl_3$ δ -73.30 (s). **^{13}C NMR** (101 MHz, $CDCl_3$) δ 190.5, 144.0, 138.2, 137.2, 135.5, 133.1, 128.8, 128.8, 128.6, 128.2, 122.8, 120.5 (q, *J* = 292.9 Hz), 82.2 – 78.8 (m), 70.9. **HRMS** (ESI) m/z: [M+H]$^+$ calcd for $C_{20}H_{14}F_9O_2^+$: 457.0854, found 457.0848.

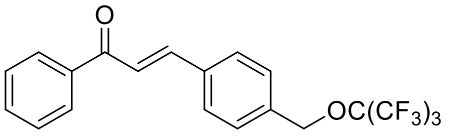

(*E*)-1-(4-(Perfluoro-*tert*-butoxymethyl)phenyl)-3-phenylprop-2-en-1-one (2a). Following the same procedure as for **1a** except that the base was replaced with NaOH (0.65 g, 16.30 mmol), **2a** was prepared as a faint yellow solid in 78% yield (2.93 g) from **B1** (3.00 g, 8.15 mmol) and benzaldehyde **C5** (0.95 g, 8.95 mmol). **^{1}H NMR** (400 MHz, $CDCl_3$) δ 8.05 (d, *J* = 8.1 Hz, 2H), 7.83 (d, *J* = 15.7 Hz, 1H), 7.65 (m, 2H), 7.58 – 7.47 (m, 3H), 7.45 – 7.40 (m, 3H), 5.13 (s, 2H). **^{19}F NMR** (376 MHz, $CDCl_3$) δ -73.31 (s). **^{13}C NMR** (101 MHz, $CDCl_3$) δ 190.0, 145.3, 139.6, 138.5, 134.8, 130.8, 129.1, 129.0, 128.6, 127.6, 121.9, 117.6 (q, *J* = 293.2 Hz), 80.7 – 79.3 (m), 70.6. **HRMS** (ESI) m/z: $[M+H]^+$ calcd for $C_{20}H_{14}F_9O_2^+$: 457.0854, found 457.0840.

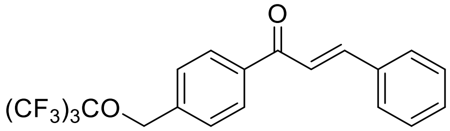

(*E*)-3-(3-(Perfluoro-*tert*-butoxymethyl)phenyl)-1-phenylprop-2-en-1-one (3a). Following the same procedure as for **1a** except that the reaction was carried out in ice bath and the base was replaced with NaOH (0.45 g, 11.29 mmol), **3a** was prepared as a faint yellow solid in 70% yield (1.80 g) from acetophenone **B4** (0.68 g, 5.65 mmol) and **C2** (2 g, 5.65 mmol). **^{1}H NMR** (500 MHz, $CDCl_3$) δ 8.05 – 8.00 (m, 2H), 7.81 (d, *J* = 15.7 Hz, 1H), 7.66 (d, *J* = 7.7 Hz, 1H), 7.63 – 7.49 (m, 2H), 7.54 – 7.50 (m, 3H), 7.47 (t, *J* = 7.7 Hz, 1H), 7.41 (d, *J* = 7.7 Hz, 1H), 5.13 (s, 2H). **^{19}F NMR** (471 MHz, $CDCl_3$) δ -73.23 (s). **^{13}C NMR** (126 MHz, $CDCl_3$) δ 190.4, 144.0, 138.1, 135.8, 135.5, 133.0, 129.6, 129.5, 128.7, 128.7, 128.66, 127.6, 122.8, 120.5 (q, *J* = 293.58 Hz), 80.6 – 79.4 (m), 70.9 (q, *J* = 1.9 Hz). **HRMS** (ESI) m/z: $[M+H]^+$ calcd for $C_{20}H_{14}F_9O_2^+$: 457.0854, found 457.0855.

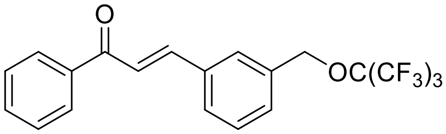

(*E*)-1-(3-(Perfluoro-*tert*-butoxymethyl)phenyl)-3-phenylprop-2-en-1-one (4a). Following the same procedure as for **1a**, **4a** was prepared as a faint yellow solid in 44% yield (1.71 g) from **B2** (3.06 g, 8.31 mmol) and benzaldehyde **C5** (1.06 g, 9.97 mmol). **^{1}H NMR** (500 MHz, $CDCl_3$) δ 8.05 – 8.00 (m, 2H), 7.81 (d, *J* = 15.7 Hz, 1H), 7.67 – 7.64 (m, 2H), 7.60 (d, *J* = 7.7 Hz, 1H), 7.56 (t, *J* = 7.7 Hz, 1H), 7.51 (d, *J* = 15.7 Hz, 1H), 7.46 – 7.42 (m, 3H), 5.09 (s, 2H). **^{19}F NMR** (471 MHz, $CDCl_3$) δ -73.25 (s). **^{13}C NMR** (126 MHz, $CDCl_3$) δ 190.1, 145.4, 138.7, 135.6, 134.8, 131.9, 130.8, 129.2, 129.1, 128.9, 128.5, 127.7, 121.9, 120.5 (q, *J* = 293.6 Hz), 80.1 – 79.9 (m), 70.9. **HRMS** (ESI) m/z: $[M+H]^+$ calcd for $C_{20}H_{14}F_9O_2^+$: 457.0854, found 457.0849.

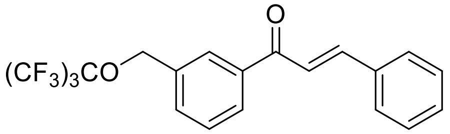

(*E*)-3-(4-(Perfluoro-*tert*-butoxymethyl)phenyl)-1-(4-methoxyphenyl)prop-2-en-1-one (5a). 4'-Methoxyacetophenone **B6** (1.27 g, 8.47 mmol) and KOH (1.42 g, 25.41 mmol) were dissolved in EtOH. The mixture was stirred for 5 min, and then **C1** (3.00 g, 8.47 mmol) was added in one portion. After stirring for 24 h at room temperature, the mixture was neutralized with 2 N HCl. After filtration, the residue was collected and redissolved in EtOAc. The solution was dried over anhydrous Na_2SO_4 and concentrated under vacuum to give the crude product, which was recrystallized from EtOAc and EtOH to give **5a** as a faint yellow solid (3.77 g, 91%). **^{1}H NMR** (400 MHz, $CDCl_3$) δ 8.04 (d, *J* = 8.9 Hz, 2H), 7.79 (d, *J* = 15.7 Hz, 1H), 7.66 (d, *J* = 8.3 Hz, 2H), 7.56 (d, *J* = 15.7 Hz, 1H), 7.39 (d, *J* = 8.3 Hz, 2H), 6.99 (d, *J* = 8.9 Hz, 2H), 5.07 (s, 2H), 3.89 (s, 3H). **^{19}F NMR** (376 MHz, $CDCl_3$) δ -73.33 (s). **^{13}C NMR** (151 MHz, $CDCl_3$) δ 188.5,

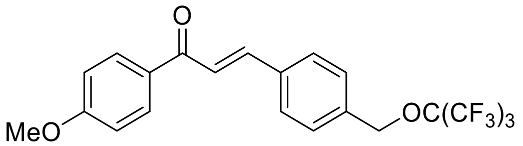

163.6, 143.0, 136.9, 135.6, 131.0, 130.9, 128.6, 128.1, 122.6, 120.4 (q, *J* = 293.3 Hz),113.9, 81.3 – 78.8 (m), 70.8, 55.5. **HRMS** (ESI) m/z: $[M+H]^+$ calcd for $C_{21}H_{16}F_9O_3^+$: 487.0950, found 487.0945.

(*E*)-1-(4-(Perfluoro-*tert*-butoxymethyl)phenyl)-3-(4-methoxyphenyl)prop-2-en-1-one (6a).

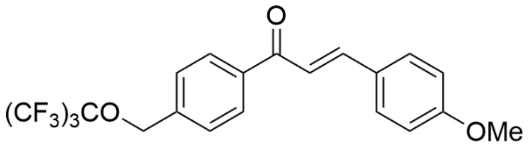

Following the same procedure as for **5a** except that the base was replaced with NaOH (1.33 g, 33.40 mmol), **6a** was prepared as a faint yellow solid in 53% yield (3.96 g) from **B1** (4.10 g, 11.13 mmol) and 4-methoxy-benzaldehyde **C4** (1.82 g, 13.36 mmol). ^{1}**H NMR** (400 MHz, $CDCl_3$) δ 8.03 (d, *J* = 8.3 Hz, 2H), 7.79 (d, *J* = 15.6 Hz, 1H), 7.60 (d, *J* = 8.3 Hz, 2H), 7.47 (d, *J* = 8.3 Hz, 2H), 7.39 (d, *J* = 15.6 Hz, 1H), 6.93 (d, *J* = 8.3 Hz, 2H), 5.12 (s, 2H), 3.84 (s, 3H). ^{19}**F NMR** (376 MHz, $CDCl_3$) δ -73.34 (s). ^{13}**C NMR** (151 MHz, $CDCl_3$) δ 190.1, 162.0, 145.2, 139.3, 138.9, 130.4, 128.9, 127.63, 127.56, 120.5 (q, *J* = 293.0 Hz), 119.7, 114.6, 81.2 – 79.3 (m), 70.7, 55.5. **HRMS** (ESI) m/z: $[M+H]^+$ calcd for $C_{21}H_{16}F_9O_3^+$: 487.0950, found 487.0958.

(*E*)-1-(3,5-Bis(trifluoromethyl)phenyl)-3-(4-(perfluoro-*tert*-butoxymethyl)phenyl)prop-2-en-1-one(7a).

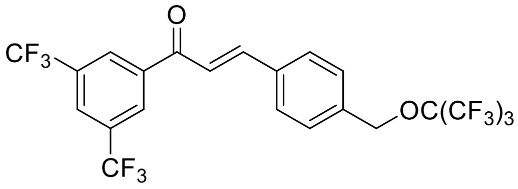

Following the same procedure as for **1a** except that the base was replaced with NaOH (0.59 g, 14.84 mmol) and the reaction time was reduced to 40 min, **7a** was prepared as a yellowish oil in 37% yield (1.62 g) from 3,5-bis(trifluoromethyl) acetophenone **B5** (1.90 g, 7.42 mmol) and **C1** (2.63 g, 7.42 mmol). ^{1}**H NMR** (500 MHz, $CDCl_3$) δ 8.44 (s, 2H), 8.10 (s, 1H), 7.91 (d, *J* = 15.6 Hz, 1H), 7.72 (d, *J* = 8.2 Hz, 2H), 7.52 (d, *J* = 15.6 Hz, 1H), 7.44 (d, *J* = 8.2 Hz, 2H), 5.10 (s, 2H). ^{19}**F NMR** (376 MHz, $CDCl_3$) δ -66.02 (s, 6F), -73.28 (s, 9F). ^{13}**C NMR** (126 MHz, $CDCl_3$) δ 187.3, 146.5, 139.7, 138.1, 134.7, 132.5 (q, *J* = 34.0 Hz), 129.1, 128.5 (q, *J* = 3.8 Hz), 128.2, 126.1 – 126.0 (m), 123.0 (q, *J* = 272.9 Hz), 120.9, 120.4 (q, *J* = 292.4 Hz), 80.9 – 79.1 (m), 70.7. **HRMS** (MALDI-TOF) m/z: $[M+H]^+$ calcd for $C_{22}H_{12}F_{15}O_2^+$: 593.0592, found 593.0592.

(*E*)-3-(3,5-Bis(perfluoro-*tert*-butoxymethyl)phenyl)-1-phenylprop-2-en-1-one (8a).

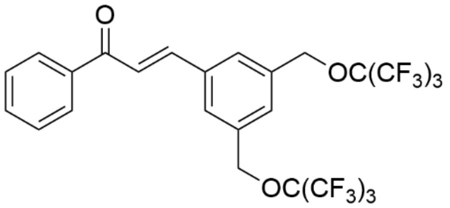

Following the same procedure as for **1a**, **8a** was prepared as a faint yellow solid in 68% yield (1.20 g) from acetophenone **B4** (0.30 g, 2.49 mmol) and **C3** (1.50 g, 2.49 mmol). ^{1}**H NMR** (500 MHz, $CDCl_3$) δ 8.03 (d, *J* = 7.3 Hz, 2H), 7.80 (d, *J* = 15.7 Hz, 1H), 7.62 – 7.54 (m, 4H), 7.51 (t, *J* = 7.6 Hz, 2H), 7.43 (s, 1H), 5.12 (s, 4H). ^{19}**F NMR** (471 MHz, $CDCl_3$) δ -73.15 (s). ^{13}**C NMR** (126 MHz, $CDCl_3$) δ 190.1, 143.2, 138.0, 136.6, 136.0, 133.1, 128.7, 128.6, 128.1, 127.4, 123.3, 120.5 (q, *J* = 292.8 Hz), 80.1 – 79.5 (m), 70.5. **HRMS** (ESI) m/z: $[M+H]^+$ calcd for $C_{25}H_{15}F_{18}O_3^+$: 705.0728, found 705.0754.

(*E*)-1-(3,5-Bis(perfluoro-*tert*-butoxymethyl)phenyl)-3-phenylprop-2-en-1-one (9a). Following the same procedure as for **1a**, **9a** was prepared as a faint yellow solid in 22% yield (1.05 g) from **B3** (4.00 g, 6.49 mmol) and benzaldehyde **C5** (0.83 g, 7.79 mmol). **^{1}H NMR** (500 MHz, $CDCl_3$) δ 7.94 (s, 2H), 7.83 (d, *J* = 15.7 Hz, 1H), 7.68 – 7.60 (m, 3H), 7.48 (d, *J* = 15.7 Hz, 1H), 7.46 – 7.42 (m, 3H), 5.16 (s, 4H). **^{19}F NMR** (471 MHz, $CDCl_3$) δ -73.28 (s). **^{13}C NMR** (126 MHz, $CDCl_3$) δ 189.6, 146.0, 139.1, 136.4, 134.6, 131.0, 130.2, 129.1, 128.6, 127.6, 121.5, 120.4 (q, *J* = 291.2 Hz), 80.0 – 79.7 (m), 70.5. **HRMS** (ESI) m/z: $[M+H]^+$ calcd for $C_{25}H_{15}F_{18}O_3^+$: 705.0728, found 705.0734.

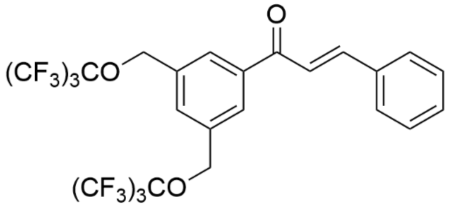

(*E*)-1,3-Bis(3-(perfluoro-*tert*-butoxymethyl)phenyl)prop-2-en-1-one (10a). Following the same procedure as for **1a**, **10a** was prepared as a faint yellow solid in 57% yield (2.59 g) from **B2** (2.38 g, 6.46 mmol) and **C2** (2.75 g, 7.76 mmol). **^{1}H NMR** (500 MHz, $CDCl_3$) δ 8.02 (d, *J* = 7.6 Hz, 1H), 7.98 (s, 1H), 7.82 (d, *J* = 15.7 Hz, 1H), 7.65 (d, *J* = 7.7 Hz, 1H), 7.60 (d, *J* = 7.6 Hz, 2H), 7.56 (t, *J* = 7.6 Hz, 1H), 7.52 (d, *J* = 15.7 Hz, 1H), 7.47 (t, *J* = 7.6 Hz, 1H), 7.41 (d, *J* = 7.6 Hz, 1H), 5.13 (s, 2H), 5.09 (s, 2H). **^{19}F NMR** (471 MHz, $CDCl_3$) δ -73.26 (s). **^{13}C NMR** (126 MHz, $CDCl_3$) δ 189.9, 144.6, 138.5, 135.8, 135.6, 135.3, 132.0, 129.8, 129.5, 129.3, 128.9, 128.8, 127.7, 127.5, 122.5, 120.5 (q, *J* = 293.1 Hz), 80.6 – 79.4 (m), 70.9. **HRMS** (ESI) m/z: $[M+H]^+$ calcd for $C_{25}H_{15}F_{18}O_3^+$: 705.0728, found 705.0727.

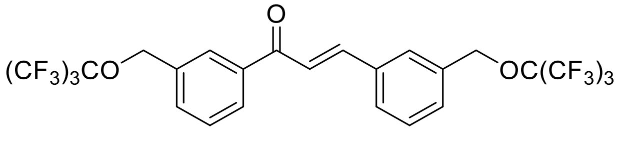

3-(4-(Perfluoro-*tert*-butoxymethyl)phenyl)-4-nitro-1-phenylbutan-1-one (1b). Compound **1a** (1.66 g, 3.64 mmol), nitromethane (2.22 g, 36.38 mmol) and KOH (41 mg, 0.73 mmol) were dissolved in EtOH and the mixture was refluxed for 5 h. The solution was cooled to room temperature, acidified with 2 N HCl, and extracted with DCM. The organic phase was dried over anhydrous Na_2SO_4 and evaporated under vacuum to give the crude product, which was purified by flash chromatography to give compound **1b** (1.77 g, yield 94%) as a yellowish oil. **^{1}H NMR** (500 MHz, $CDCl_3$) δ 7.92 (d, *J* = 6.7 Hz, 2H), 7.58 (t, *J* = 7.4 Hz, 1H), 7.46 (t, *J* = 7.7 Hz, 2H), 7.34 – 7.30 (m,, 4H), 5.00 (s, 2H), 4.84 (dd, *J* = 12.6, 6.5 Hz, 1H), 4.70 (dd, *J* = 12.6, 8.1 Hz, 1H), 4.30 – 4.21 (m, 1H), 3.53 – 3.39 (m, 2H). **^{19}F NMR** (376 MHz, $CDCl_3$) δ -73.27 (s). **^{13}C NMR** (126 MHz, $CDCl_3$) δ 196.8, 140.0, 136.4, 134.6, 133.8, 128.9, 128.5, 128.1, 128.0, 120.5 (q, *J* = 293.1 Hz), 80.7 – 79.6 (m), 79.5, 70.9, 41.5, 39.1. **HRMS** (ESI) m/z: $[M+Na]^+$ calcd for $C_{21}H_{16}F_9NNaO_4^+$: 540.0828, found 540.0834.

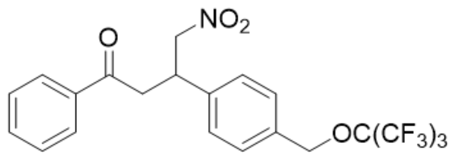

1-(4-(Perfluoro-*tert*-butoxymethyl)phenyl)-4-nitro-3-phenylbutan-1-one (2b). Following the same procedure as for **1b**, **2b** was prepared as yellowish oil in 94% yield (3.69 g) from **2a** (3.45 g, 7.56 mmol). **^{1}H NMR** (400 MHz, $CDCl_3$) δ 7.94 (d, *J* = 8.3 Hz, 2H), 7.43 (d, *J* = 8.3 Hz, 2H), 7.37 – 7.31 (m, 2H), 7.30 – 7.26 (m, 3H), 5.10 (s, 2H), 4.83 (dd, *J* = 12.5, 6.7 Hz, 1H), 4.69 (dd, *J* = 12.5, 7.9 Hz, 1H), 4.27 – 4.20 (m, 1H), 3.54 – 3.38 (m, 2H). **^{19}F**

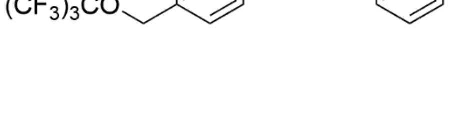

NMR (376 MHz, $CDCl_3$) δ -73.34 (s). **^{13}C NMR** (151 MHz, $CDCl_3$) δ 196.4, 140.4, 139.1, 136.7, 129.2, 128.6, 128.1, 127.58, 127.57, 120.5 (q, *J* = 295.0 Hz), 80.1 – 79.6 (m), 79.7, 70.5, 41.7, 39.4. **HRMS** (ESI) m/z: $[M+Na]^+$ calcd for $C_{21}H_{16}F_9NNaO_4^+$: 540.0828, found 540.0837.

3-(3-(Perfluoro-*tert*-butoxymethyl)phenyl)-4-nitro-1-phenylbutan-1-one (3b). Following the same procedure as for **1b**, **3b** was prepared as a yellowish oil in 76% yield (1.48 g) from **3a** (1.71 g, 3.75 mmol). **^{1}H NMR** (500 MHz, $CDCl_3$) δ 7.89 (d, *J* = 8.0 Hz, 2H), 7.55 (t, *J* = 7.3 Hz, 1H), 7.46 – 7.41 (m, 2H), 7.37 – 7.32 (m, 1H), , 7.28 – 7.21 (m, 3H), 4.83 (dd, *J* = 12.5, 6.8 Hz, 1H), 4.70 (dd, *J* = 12.5, 7.8 Hz, 1H), 4.24 – 4.22 (m, 1H), 3.53 – 3.39 (m, 2H). **^{19}F NMR** (471 MHz, $CDCl_3$) δ -73.28 (s). **^{13}C NMR** (126 MHz, $CDCl_3$) δ 196.4, 139.0, 136.8, 135.7, 132.7, 129.3, 129.2, 128.5, 128.0, 127.5, 127.2, 120.4 (q, *J* = 293.2 Hz), 80.6 – 79.62 (m), 79.55, 70.7 (q, *J* = 1.9 Hz), 41.6, 39.3. **HRMS** (ESI) m/z: $[M+Na]^+$ calcd for $C_{21}H_{16}F_9NNaO_4^+$: 540.0828, found 540.0819.

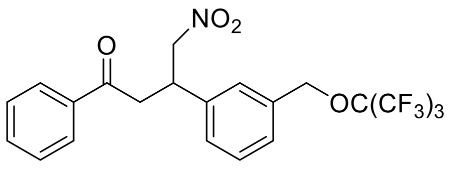

1-(3-(Perfluoro-*tert*-butoxymethyl)phenyl)-4-nitro-3-phenylbutan-1-one (4b). Following the same procedure as for **1b**, **4b** was prepared as a yellowish oil in 90% yield (1.08 g) from **4a** (1.06 g, 2.32 mmol). **^{1}H NMR** (600 MHz, $CDCl_3$) δ 7.91 (d, *J* = 7.8 Hz, 1H), 7.86 (s, 1H), 7.58 (d, *J* = 7.8 Hz, 1H), 7.51 (t, *J* = 7.8 Hz, 1H), 7.37 – 7.32 (m, 2H), 7.29 – 7.26 (m, 3H), 5.00 (s, 2H), 4.83 (dd, *J* = 12.5, 6.8 Hz, 1H), 4.70 (dd, *J* = 12.5, 7.8 Hz, 1H), 4.26 – 4.20 (m, 1H), 3.52 – 3.39 (m, 2H). **^{19}F NMR** (471 MHz, $CDCl_3$) δ -73.28 (s). **^{13}C NMR** (126 MHz, $CDCl_3$) δ 196.7, 139.8, 136.3, 135.8, 133.7, 129.6, 128.8, 128.1, 127.9, 127.2, 126.7, 123.9 – 116.9 (q, *J* = 293.2 Hz), 80.5 – 79.5 (m), 79.4, 71.0, 41.5, 39.2. **HRMS** (ESI) m/z: $[M+Na]^+$ calcd for $C_{21}H_{16}F_9NNaO_4^+$: 540.0828, found 540.0831.

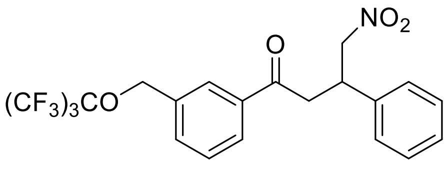

3-(4-(Perfluoro-*tert*-butoxymethyl)phenyl)-1-(4-methoxyphenyl)-4-nitrobutan-1-one (5b). Following the same procedure as for **1b**, **5b** was prepared as a yellowish oil in 94% yield (4.02 g) from **5a** (3.77 g, 7.75 mmol). **^{1}H NMR** (400 MHz, $CDCl_3$) δ7.90 (d, *J* = 8.9 Hz, 2H), 7.32 – 7.29 (m, 4H), 6.92 (d, *J* = 8.9 Hz, 2H), 5.00 (s, 2H), 4.84 (dd, *J* = 12.6, 6.4 Hz, 1H), 4.68 (dd, *J* = 12.6, 8.3 Hz, 1H), 4.20–4.27 (m, 1H), 3.86 (s, 3H), 3.47 – 3.31 (m, 2H). **^{19}F NMR** (376 MHz, $CDCl_3$) δ -73.30 (s). **^{13}C NMR** (151 MHz, $CDCl_3$) δ 195.2, 164.0, 140.2, 134.5, 130.5, 129.5, 128.4, 128.0, 120.4 (q, *J* = 292.9 Hz), 114.0, 80.6 – 79.7 (m), 79.6, 70.9, 55.6, 41.2, 39.2. **HRMS** (ESI) m/z: $[M+H]^+$ calcd for $C_{22}H_{19}F_9NO_5^+$: 548.1114, found 548.1164.

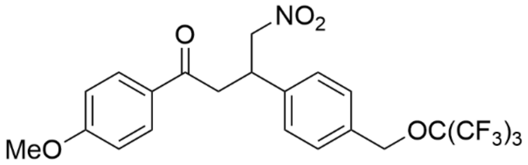

1-(4-(Perfluoro-*tert*-butoxymethyl)phenyl)-3-(4-methoxyphenyl)-4-nitrobutan-1-one (6b). Following the same procedure as for **1b**, **6b** was prepared as a yellowish oil in a 93% yield (4.02 g) from a **6a** (3.81 g, 7.83 mmol). **^{1}H NMR** (400 MHz, $CDCl_3$) δ 7.93 (d, *J* = 8.3 Hz, 2H), 7.42 (d, *J* = 8.3 Hz, 2H), 7.20 (d, *J* = 8.5 Hz, 2H), 6.85 (d, *J* = 8.5 Hz, 2H), 5.10 (s, 2H), 4.79 (dd, *J* = 12.4, 6.6 Hz, 1H), 4.64 (dd, *J* = 12.4, 8.1 Hz, 1H), 4.24 – 4.10 (m, 1H), 3.74 (s, 3H), 3.50 – 3.33 (m, 2H). **^{19}F NMR** (376 MHz, $CDCl_3$) δ -73.35 (s). **^{13}C NMR** (101 MHz,

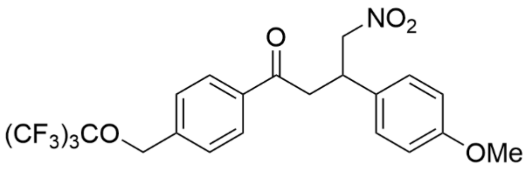

$CDCl_3$) δ 196.6, 159.1, 140.3, 136.6, 131.0, 128.6, 128.5, 127.5, 120.4 (q, *J* = 292.8 Hz), 114.4, 81.4 – 78.7 (m), 79.9, 70.5, 55.1, 41.7, 38.6. **HRMS** (ESI) m/z: $[M+H]^+$ calcd for $C_{22}H_{19}F_9NO_5^+$: 548.1114, found 548.1104.

1-(3,5-Bis(trifluoromethyl)phenyl)-3-(4-(perfluoro-*tert*-butoxymethyl)phenyl)-4-nitrobutan-1-one(7b). Following the same procedure as for **1b**, **7b** was prepared as a yellowish oil in 69% yield (2.04 g) from **7a** (2.69 g, 4.54 mmol). **^{1}H NMR** (400 MHz, $CDCl_3$) δ 8.32 (s, 2H), 8.08 (s, 1H), 7.36 – 7.33 (m, 4H), 5.00 (s, 2H), 4.83 (dd, *J* = 12.7, 7.2 Hz, 1H), 4.73 (dd, *J* = 12.7, 7.4 Hz, 1H), 4.32 – 4.25 (m, 1H), 3.59 – 3.48 (m, 2H). **^{19}F NMR** (376 MHz, $CDCl_3$) δ -66.14 (s, 6F), -73.29 (s, 9F). **^{13}C NMR** (151 MHz, $CDCl_3$) δ 194.2, 139.2, 137.8, 135.1, 132.7 (q, *J* = 34.1 Hz), 128.6, 128.2, 128.0, 127.0 – 126.9 (m), 122.9 (q, *J* = 273.0 Hz), 120.5 (q, *J* = 293.0 Hz), 80.1 – 70.6 (m), 79.1, 70.8, 41.9, 39.0.

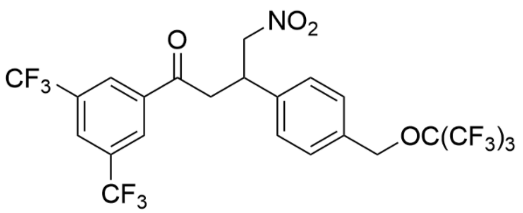

3-(3,5-Bis(perfluoro-*tert*-butoxymethyl)phenyl)-4-nitro-1-phenylbutan-1-one (8b). Following the same procedure as for **1b**, **8b** was prepared as a yellowish oil in 66% yield (0.80 g) from **8a** (1.10 g, 1.56 mmol). **^{1}H NMR** (500 MHz, $CDCl_3$) δ 7.91 (d, *J* = 7.4 Hz, 2H), 7.59 (t, *J* = 7.4 Hz, 1H), 7.46 (t, *J* = 7.4 Hz, 2H), 7.29 (s, 1H), 7.23 (s, 2H), 5.05 (s, 4H), 4.85 (dd, *J* = 12.7, 6.4 Hz, 1H), 4.70 (dd, *J* = 12.7, 8.1 Hz, 1H), 4.30 – 4.25 (m, 1H), 3.54 – 3.39 (m, 2H). **^{19}F NMR** (471 MHz, $CDCl_3$) δ -73.32 (s). **^{13}C NMR** (126 MHz, $CDCl_3$) δ 196.5, 140.4, 136.6, 136.2, 133.8, 128.8, 128.0, 126.7, 125.7, 120.4 (q, *J* = 292.5 Hz), 81.1 – 78.9 (m), 79.2, 70.5, 41.3, 39.0. **HRMS** (MALDI-TOF) m/z: $[M+H]^+$ calcd for $C_{26}H_{18}F_{18}NO_5^+$: 766.0892, found 766.0894.

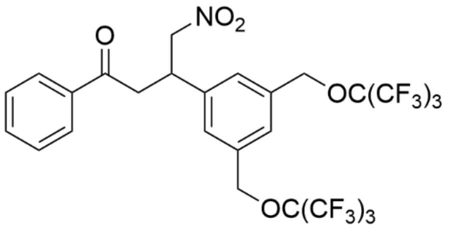

1-(3,5-Bis(perfluoro-*tert*-butoxymethyl)phenyl)-4-nitro-3-phenylbutan-1-one (9b). Following the same procedure as for **1b**, **9b** was prepared as a yellowish oil in 81% yield (0.92 g) from **9a** (1.05 g, 1.49 mmol). **^{1}H NMR** (500 MHz, $CDCl_3$) δ 7.83 (s, 2H), 7.62 (s, 1H), 7.35 – 7.32 (m, 2H), 7.29 – 7.26 (m, 3H), 5.11 (s, 4H), 4.82 (dd, *J* = 12.5, 6.9 Hz, 1H), 4.70 (dd, *J* = 12.5, 7.6 Hz, 1H), 4.25 – 4.20 (m, 1H), 3.53 – 3.39 (m, 2H). **^{19}F NMR** (471 MHz, $CDCl_3$) δ -73.27 (s). **^{13}C NMR** (126 MHz, $CDCl_3$) δ 195.9, 138.9, 137.2, 136.6, 131.0, 129.2, 128.1, 127.5, 127.1, 120.4 (q, *J* = 292.6 Hz), 81.0 – 79.5 (m), 79.5, 70.3, 41.6, 39.3. **HRMS** (MALDI-TOF) m/z: $[M+H]^+$ calcd for $C_{26}H_{18}F_{18}NO_5^+$: 766.0892, found 766.0894.

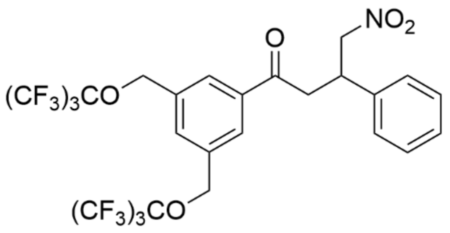

1,3-Bis(3-(perfluoro-*tert*-butoxymethyl)phenyl)-4-nitrobutan-1-one (10b). Following the same procedure as for **1b**, **10b** was prepared as a yellowish oil in 72% yield (1.99 g) from **10a** (2.55 g, 3.62 mmol). **^{1}H NMR** (500 MHz, $CDCl_3$) δ 7.91 (d, *J* = 7.6 Hz, 1H), 7.86 (s, 1H), 7.58 (d, *J* = 7.6 Hz, 1H), 7.51 (t, *J* = 7.6 Hz, 1H),

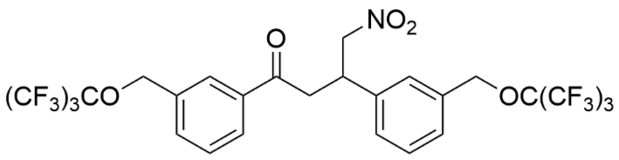

7.38 (t, *J* = 7.6 Hz, 1H), 7.32 – 7.26 (m, 2H), 7.24 (s, 1H), 5.07 (s, 2H), 5.02 (s, 2H), 4.84 (dd, *J* = 12.6, 6.7 Hz, 1H), 4.71 (dd, *J* = 12.6, 7.8 Hz, 1H), 4.28 – 4.23 (m, 1H), 3.53 – 3.40 (m, 2H). **^{19}F NMR** (471 MHz, $CDCl_3$) δ -73.30 (s). **^{13}C NMR** (126 MHz, $CDCl_3$) δ 196.2, 139.6, 136.7, 135.9, 135.7, 132.7, 129.6, 129.4, 128.4, 127.9, 127.2, 127.1, 126.6, 120.4 (q, *J* = 293.4 Hz), 80.9 – 79.1 (m), 79.3, 70.9, 70.6, 41.5, 39.1. **HRMS** (MALDI-TOF) m/z: $[M+H]^+$ calcd for $C_{26}H_{18}F_{18}NO_5^+$: 766.0892, found 766.0894.

5,5-Difluoro-1,9-bis(4-(perfluoro-*tert*-butoxymethyl)phenyl)-3,7-diphenyl-5*H*-4λ^4,5λ^4-dipyrrolo[1,2-*c*:2',1'-f][1,3,5,2]triazaborinine (PS1).

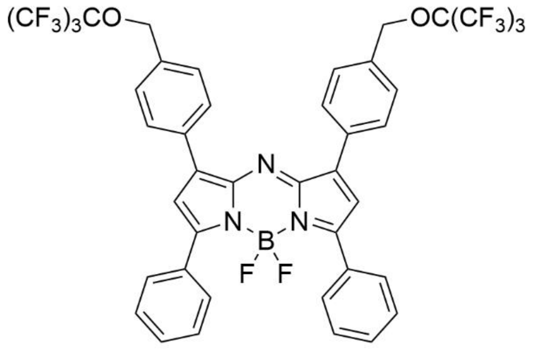

The mixture of **1b** (0.65 g, 1.26 mmol) and ammonium acetate (3.39 g, 43.97 mmol) was stirred at 120 °C for 5 h. After cooling to room temperature, the reaction mixture was washed with water, extracted with DCM, and recrystallized from DCM and MeOH to give crude product (0.22 g), which was used in the next step without further purification. Under an argon atmosphere, the intermediate mentioned above and DIPEA (0.99 g, 6.98 mmol) were dissolved in dried DCM, the resulting solution was stirred at room temperature for 20 min. Then boron trifluoride diethyl etherate complex ($BF_3 \cdot Et_2O$, 0.60 g, 4.65 mmol) was added, and the resulting solution was stirred at room temperature for 24 h. After quenching the reaction mixture with water, the organic layer was collected, and the aqueous layer was extracted with DCM. The combined organic layer was dried over anhydrous Na_2SO_4 and concentrated under vacuum. The residue was purified by flash chromatography to give compound **PS1** as a brown solid (66 mg, yield 10%).**^{1}H NMR** (500 MHz, $CDCl_3$) δ 8.09 – 8.02 (m, 8H), 7.52 – 7.48 (m, 6H), 7.44 (d, *J* = 8.1 Hz, 4H), 7.06 (s, 2H), 5.11 (s, 4H). **^{19}F NMR** (471 MHz, $CDCl_3$) δ -73.36 (s, 18F), -134.54 – -134.75 (m, 2F). **^{13}C NMR** (126 MHz, $CDCl_3$) δ 160.0, 145.8, 143.6, 136.2, 132.8, 131.6, 131.2, 129.8, 128.8, 128.0, 129.73, 120.6 (q, *J* = 291.6 Hz), 119.4, 80.8 – 79.4 (m), 71.1. **HRMS** (MALDI-TOF) m/z: $[M]^+$ calcd for $C_{42}H_{24}BF_{20}N_3O_2^+$: 993.1643, found 993.1635.

5,5-Difluoro-3,7-bis(4-(perfluoro-*tert*-butoxymethyl)phenyl)-1,9-diphenyl-5H-4λ^4,5λ^4-dipyrrolo[1,2-*c*:2',1'-f][1,3,5,2]triazaborinine (PS2).

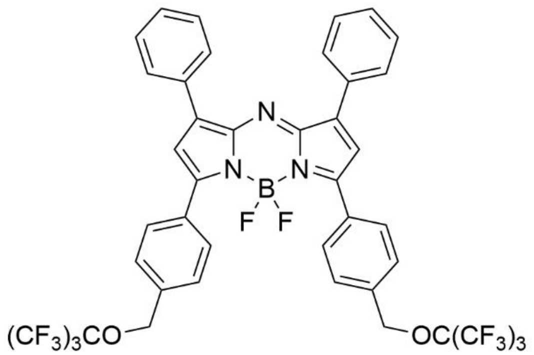

Following the same procedure as for **PS1**, **PS2** was prepared as a dark solid in 2% yield (44 mg) from **2b** (2.00 g, 3.86 mmol). **^{1}H NMR** (400 MHz, $CDCl_3$) δ 8.12 – 8.03 (m, 8H), 7.51 – 7.43 (m, 10H), 7.05 (s, 2H), 5.11 (s, 4H). **^{19}F NMR** (376 MHz, $CDCl_3$) δ -73.28 (s, 18F), -134.31 – -134.56 (m, 2F). **^{13}C NMR** (151 MHz, $CDCl_3$) δ 158.9, 145.8, 144.6, 137.6, 132.2, 131.9, 129.94 – 128.88 (m), 129.7, 129.4, 128.7, 127.5, 120.4 (q, *J* = 292.8 Hz), 119.1, 80.6 – 79.2 (m), 70.7. **HRMS** (MALDI-TOF) m/z: $[M]^+$ calcd for $C_{42}H_{24}BF_{20}N_3O_2^+$: 993.1643, found 993.1638.

5,5-Difluoro-1,9-bis(3-(perfluoro-*tert*-butoxymethyl)phenyl)-3,7-diphenyl-5H-4λ^4,5λ^4-dipyrrolo[1,2-*c*:2',1'-f][1,3,5,2]triazaborinine (PS3). Following the same procedure as for **PS1** except that the reaction was carried out at 40 °C, **PS3** was prepared as a dark solid in 13% yield (0.14 g) from **3b** (1.05 g, 2.03 mmol). **^{1}H NMR** (500 MHz, $CDCl_3$) δ 8.06 – 8.04 (m, 6H), 7.90 (s, 2H), 7.51 – 7.49 (m, 7H), 7.46 (t, *J* = 7.6 Hz, 1H), 7.05 (s, 2H), 5.08 (s, 4H). **^{19}F NMR** (471 MHz, $CDCl_3$) δ -73.29 (s, 18F), -134.61 – -134.80 (m,, 2F). **^{13}C NMR** (151 MHz, $CDCl_3$) δ 159.8, 145.6, 143.5, 135.4, 132.7, 131.4, 131.2, 129.9, 129.68 – 129.63 (m), 129.0, 128.7, 128.6, 128.3, 123.3 – 117.5 (q, *J* = 293.0 Hz), 119.5, 80.8 – 79.0 (m), 71.1. **HRMS** (MALDI-TOF) m/z: [M]$^+$ calcd for $C_{42}H_{24}BF_{20}N_3O_2^+$: 993.1643, found 993.1644.

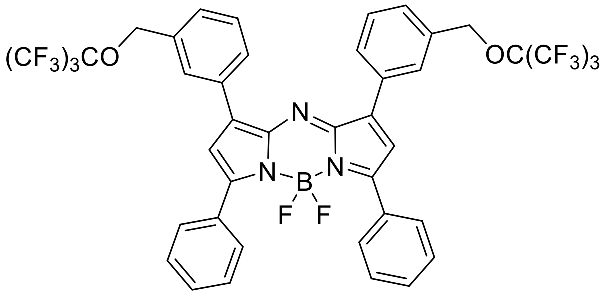

5,5-Difluoro-3,7-bis(3-(perfluoro-*tert*-butoxymethyl)phenyl)-1,9-diphenyl-5H-4λ^4,5λ^4-dipyrrolo[1,2-*c*:2',1'-f][1,3,5,2]triazaborinine (PS4). Following the same procedure as for **PS1** except that the reaction was carried out at 40 °C, **PS4** was prepared as a dark solid in 15% yield (0.17 g) from **4b** (1.20 g, 2.32 mmol). **^{1}H NMR** (500 MHz, $CDCl_3$) δ 8.08 – 8.05 (m, 6H), 7.98 (s, 2H), 7.55 – 7.41 (m, 10H), 7.02 (s, 2H), 5.11 (s, 4H). **^{19}F NMR** (471 MHz, $CDCl_3$) δ -73.27 (s, 18F), -134.40 – -134.60 (m, 2F). **^{13}C NMR** (126 MHz, $CDCl_3$) δ 159.1, 145.8, 144.7, 135.3, 132.2, 132.1, 130.0, 129.8, 129.5, 129.1, 128.7, 128.7 – 128.6 (m), 120.5 (q, *J* = 293.1 Hz), 119.1, 80.6 – 79.4 (m), 71.0. **HRMS** (MALDI-TOF) m/z: [M]$^+$ calcd for $C_{42}H_{24}BF_{20}N_3O_2^+$: 993.1643, found 993.1647.

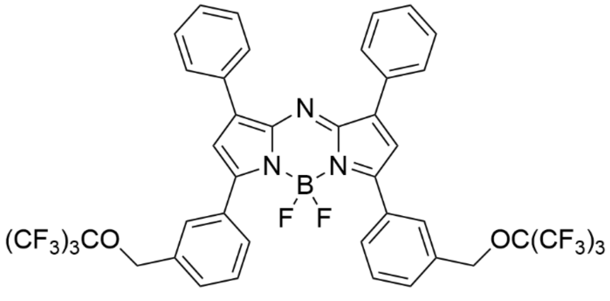

5,5-Difluoro-1,9-bis(4-(perfluoro-*tert*-butoxymethyl)phenyl)-3,7-bis(4-methoxyphenyl)-5H-4λ^4,5λ^4-dipyrrolo[1,2-*c*:2',1'-f][1,3,5,2]triazabori--nine (PS5). Following the same procedure as for **PS1**, **PS5** was prepared as a dark solid in 5% yield (51 mg) from **5b** (0.96 g, 1.75 mmol). **^{1}H NMR** (500 MHz, $CDCl_3$) δ 8.09 (d, *J* = 7.9 Hz, 4H), 8.06 (d, *J* = 7.9 Hz, 4H), 7.42 (d, *J* = 7.9 Hz, 4H), 7.06 (s, 2H), 7.02 (d, *J* = 7.9 Hz, 4H)., 5.10 (s, 4H), 3.90 (s, 6H). **^{19}F NMR** (376 MHz, $CDCl_3$) δ -73.42 (s, 18F), -135.16 – -135.41 (m, 2F). **^{13}C NMR** (126 MHz, $CDCl_3$) δ 162.1, 158.4, 145.4, 142.4, 135.8, 133.0, 131.78 – 131.70 (m), 129.6, 127.8, 124.0, 120.5 (q, *J* = 293.6 Hz), 118.9, 114.4, 80.57 – 79.04 (m), 71.0, 55.5. **HRMS** (MALDI-TOF) m/z: [M]$^+$ calcd for $C_{44}H_{28}BF_{20}N_3O_4^+$: 1053.1855, found 1053.1840.

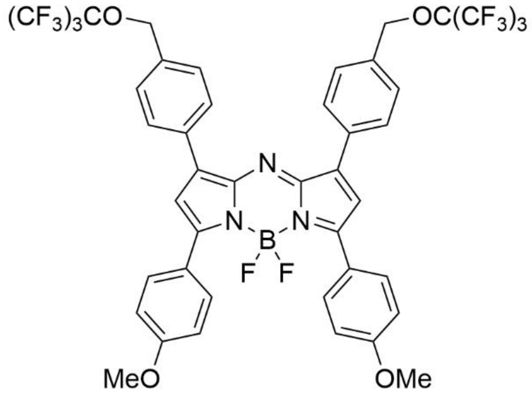

5,5-Difluoro-3,7-bis(4-(perfluoro-*tert*-butoxymethyl)phenyl)-1,9-bis(4-methoxyphenyl)-5H-4λ⁴,5λ⁴-dipyrrolo[1,2-*c*:2',1'-f][1,3,5,2]triazabori--nine (PS6). Following the same procedure as for **PS1**, **PS6** was prepared as a dark solid in 3% yield (57 mg) from **6b** (1.61 g, 2.94 mmol). ^{1}**H NMR** (400 MHz, $CDCl_3$) δ 8.10 – 8.01 (m, 8H), 7.45 (d, *J* = 8.3 Hz, 4H), 7.01 (d, *J* = 8.9 Hz, 4H), 6.93 (s, 2H), 5.10 (s, 4H), 3.91 (s, 6H). ^{19}**F NMR** (376 MHz, $CDCl_3$) δ -73.31 (s, 18F), -133.81 – -134.07 (m, 2F). ^{13}**C NMR** (126 MHz, THF–d_8) δ 161.4, 158.3, 145.6, 143.8, 137.3, 132.3, 130.8, 129.87 – 129.79 (m), 127.4, 125.1, 120.6 (q, *J* = 293.6 Hz), 117.6, 114.1, 80.9 – 79.8 (m), 71.2, 54.7. **HRMS** (MALDI-TOF) m/z: $[M]^+$ calcd for $C_{44}H_{28}BF_{20}N_3O_4^+$: 1053.1855, found 1053.1843.

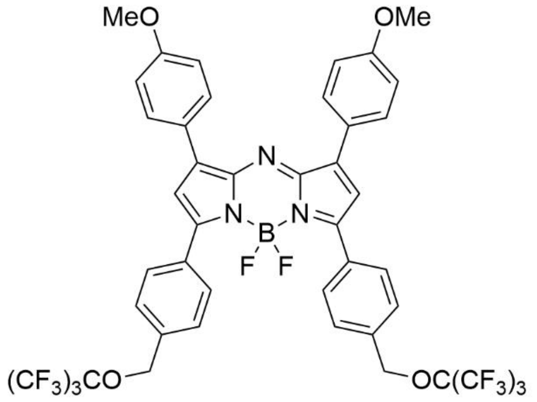

3,7-Bis(3,5-bis(trifluoromethyl)phenyl)-5,5-difluoro-1,9-bis(4-(perfluoro-*tert*-butoxymethyl)phenyl)- 5H-4λ⁴,5λ⁴-dipyrrolo[1,2-*c*:2',1'-f][1,3,5,2] triazaborinine (PS7). Following the same procedure as for **PS1**, **PS7** was prepared as a dark solid in 3% yield (60 mg) from **7b** (2.04 g, 3.44 mmol). ^{1}**H NMR** (500 MHz, $CDCl_3$) δ 8.54 (s, 4H), 8.09 (d, *J* = 8.0 Hz, 4H), 8.00 (s, 2H), 7.47 (d, *J* = 8.0 Hz, 4H), 7.15 (s, 2H), 5.14 (s, 4H). ^{19}**F NMR** (471 MHz, $CDCl_3$) δ -66.36 (s, 12F), -73.38 (s, 18F), -133.49 – -133.69 (m, 2F). ^{13}**C NMR** (151 MHz, $CDCl_3$) δ 157.0, 146.3, 145.6, 137.2, 132.8, 132.4 (q, *J* = 33.9 Hz), 131.9, 130.0, 129.6, 128.0, 124.5, 123.0 (q, *J* = 272.9 Hz), 120.5 (q, *J* = 293.2 Hz), 118.9, 80.4 – 79.7 (m), 70.8. **HRMS** (MALDI-TOF) m/z: $[M]^+$ calcd for $C_{46}H_{20}BF_{32}N_3O_2^+$: 1265.1139, found 1265.1123.

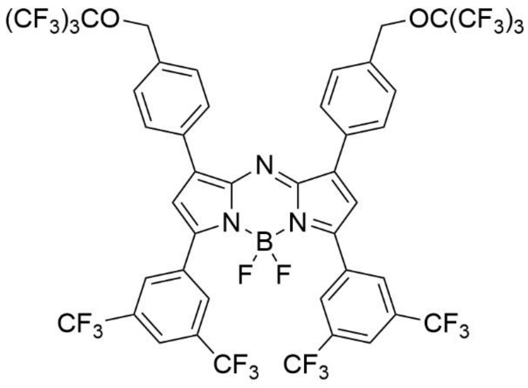

1,9-Bis(3,5-bis(perfluoro-*tert*-butoxymethyl)phenyl)-5,5-difluoro-3,7-diphenyl-5H-4λ⁴,5λ⁴-dipyrrolo[1,2-*c*:2',1'-f][1,3,5,2]triazaborinine (PS8). Following the same procedure as for **PS1** except that the reaction was carried out at 40 °C, **PS8** was prepared as a dark solid in 13% yield (108 mg) from **8b** (0.80 g, 1.04 mmol). ^{1}**H NMR** (500 MHz, $CDCl_3$) δ 8.08 –8.04 (m, 4H), 7.78 (s, 4H), 7.53 – 7.50 (m, 6H), 7.44 (s, 2H), 7.04 (s, 2H), 5.00 (s, 8H). ^{19}**F NMR** (471 MHz, $CDCl_3$) δ -73.41 (s, 36F), -134.24 – -134.43 (m, 2F). ^{13}**C NMR** (126 MHz, $CDCl_3$) δ 160.0, 145.7, 143.3, 135.9, 133.2, 131.33, 131.27, 129.74 – 129.68 (m), 128.8, 128.7, 120.3 (q, *J* = 293.6 Hz), 119.9, 81.7 – 77.6 (m), 70.5. **HRMS** (MALDI-TOF) m/z: $[M]^+$ calcd for $C_{52}H_{26}BF_{38}N_3O_4^+$: 1489.1412, found 1489.1393.

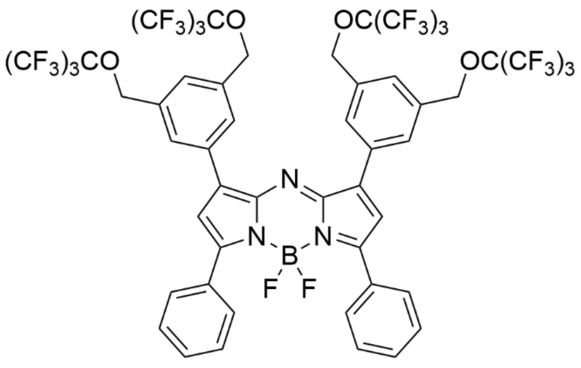

3,7-Bis(3,5-bis(perfluoro-*tert*-butoxymethyl)phenyl)-5,5-difluoro-1,9-diphenyl-5H-4λ^4,5λ^4-dipyrrolo[1,2-*c*:2',1'-f][1,3,5,2]triazaborinine (PS9). Following the same procedure as for **PS1** except that the reaction was carried out at 40 °C, **PS9** was prepared as a dark solid in 20% yield (369 mg) from **9b** (1.83 g, 2.39 mmol). **^{1}H NMR** (600 MHz, $CDCl_3$) δ 8.07 – 8.05 (m, 4H), 7.91 (s, 4H), 7.52 (s, 2H), 7.50 – 7.46 (m, 6H), 7.00 (s, 2H), 5.13 (s, 8H). **^{19}F NMR** (471 MHz, $CDCl_3$) δ -73.52 (s, 36F), -134.72 – -134.91 (m, 2F). **^{13}C NMR** (126 MHz, $CDCl_3$) δ 158.8, 146.0, 145.3, 136.3, 132.6, 132.1, 130.1, 129.6, 128.9, 128.6, 128.4, 120.5 (q, *J* = 292.8 Hz), 119.3, 80.5 – 79.5 (m), 70.6. **HRMS** (MALDI-TOF) m/z: $[M]^+$ calcd for $C_{52}H_{26}BF_{38}N_3O_4^+$: 1489.1412, found 1489.1400.

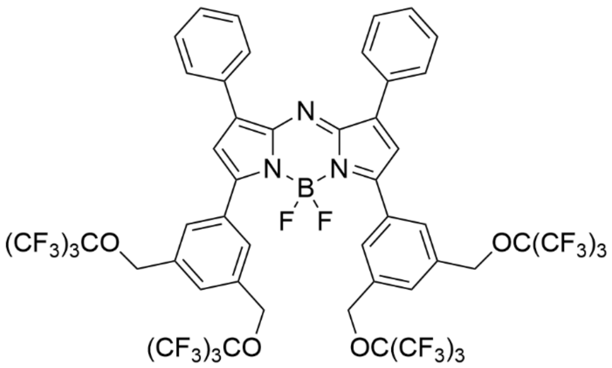

5,5-Difluoro-1,3,7,9-tetrakis(3-(perfluoro-*tert*-butoxymethyl)phenyl)-5H-4λ^4,5λ^4-dipyrrolo[1,2-*c*:2',1'-f][1,3,5,2]triazaborinine (PS10). Following the same procedure as for **PS1** except that the reaction was carried out at 40 °C, **PS10** was prepared as a dark solid in 15% yield (97 mg) from **10b** (1.98 g, 2.60 mmol). **^{1}H NMR** (500 MHz, $CDCl_3$) δ 8.06 – 7.84 (m, 4H), 7.98 (s, 2H), 7.90(s, 2H), 7.55 –7.45 (m, 7.7 Hz, 8H), 7.03 (s, 2H), 5.11 (s, 4H), 5.08 (s, 4H). **^{19}F NMR** (471 MHz, $CDCl_3$) δ -73.30 (s, 36F), -134.52 – -134.73 (m, 2F). **^{13}C NMR** (151 MHz, $CDCl_3$) δ 159.4, 145.8, 144.1, 135.6, 135.5, 132.6, 131.9, 130.3, 130.11 – 130.08 (m), 130.0, 129.3, 129.1, 128.9, 128.7 – 128.66 (m), 128.4, 120.5 (q, *J* = 292.39 Hz), 80.3 – 79.7 (m), 71.0, 70.97. **HRMS** (MALDI-TOF) m/z: $[M]^+$ calcd for $C_{52}H_{26}BF_{38}N_3O_4^+$: 1489.1412, found 1489.1394.

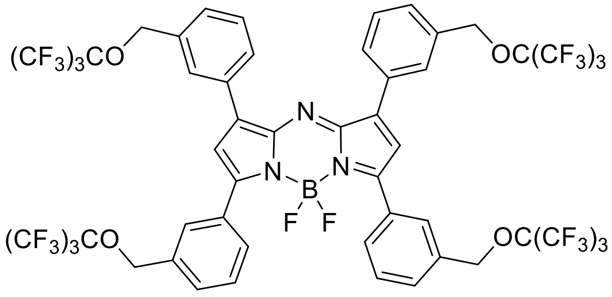

F-oil. 1,5-Dibromopentane **14** (10 g, 43.49 mmol) and potassium perfluoro-*tert*-butoxide (35.76 g, 130.45 mmol) were dissolved in dry DMF and stirred for 12 h at 80 °C. The solution was cooled to room temperature, washed with water, and the lower oily liquid was collected, purified by distillation to give compound **F-oil** as a clear liquid (19.23 g, 81%). **^{1}H NMR** (500 MHz, $CDCl_3$) δ 4.04 – 4.01 (m, 4H), 1.74 – 1.69 (m, 4H), 1.54 – 1.51 (m, 2H). **^{19}F NMR** (471 MHz, $CDCl_3$) δ -73.58 (s). **^{13}C NMR** (126 MHz, $CDCl_3$) δ 120.4 (q, *J* = 292.72 Hz), 80.4 – 79.2 (m), 69.4, 29.2, 21.4.

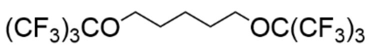

F-oil

Aza-BODIPYs **11-13** were synthesized in our previous work and the corresponding references were cited (*34*).

4.2 FL quantum yield measurements

Aza-BODIPY **11** (ϕ_f = 0.34, in $CHCl_3$) was used as the reference for calculating the quantum yield.[1] PSs were dissolved in $CHCl_3$ at concentrations corresponding to UV-absorption values of

0.01-0.05 to minimize reabsorption effects. Quantum yields were calculated using the following formula (1):[2]

$$\phi_{fX} = \phi_{fR} \times \frac{F_X}{F_R} \times \left(\frac{1-e^{-A_R^{\lambda ex}}}{1-10^{-A_X^{\lambda ex}}}\right) \times \left(\frac{{\eta_X}^2}{{\eta_R}^2}\right) \quad (1)$$

Where X and R represent the PSs and the known standard reference substance **11**, respectively. F denotes the integrated area of the FL spectrum. A (λ_{ex}) represents the absorbance at the excitation wavelength, and η represents the refractive index of the solvent.

4.3 Photothermal properties measurement of PSs

The temperature changes of the PSs in $CHCl_3$ (20 μM) under 660 nm laser irradiation (0.5 Wcm^{-2}) for 6 min were recorded with a thermal imager. The final result represents the average value of three parallel tests.

4.4 Detection of ROS generation of PSs

The generation of singlet oxygen from **PS1-PS10** and aza-BODIPYs **11-13** was investigated using DPBF as an indicator. A solution containing 1 μM of the PS was mixed with 40 μM of DPBF in $CHCl_3$. The absorbance changes of DPBF at 415 nm under irradiation (660 nm) at 0.5 Wcm^{-2} were recorded. The final result represents the average of three parallel tests. Tetraphenylporphyrin (TPP) in $CHCl_3$ ($\phi_\Delta = 0.55$)[3] was used as the reference. The photooxidation of DPBF was monitored between 0 to 4 min, depending on the efficiency of the PSs. Singlet oxygen quantum yields were calculated using the following formula (2):[4]

$$\phi_{\Delta X} = \phi_{\Delta R} \times \left(\frac{1-10^{-A_R^{660}}}{1-10^{-A_X^{660}}}\right) \times \left(\frac{S_X}{S_R}\right) \quad (2)$$

Where X and R represent the PSs and the known standard reference substance TPP, respectively. ϕ_Δ denotes the quantum yield of singlet oxygen, S is the slope of the plot representing the change in absorbance of DPBF (at 415 nm) over irradiation time, and $(1 - 10^{-A})$ is the absorption correction factor, derived from the absorbance at the irradiation wavelength.

4.5 In vitro ^{19}F MRI

The MRI phantom study was conducted on a 9.4T scanner (Bruker) using the RARE (rapid acquisition with refocused echoes) sequence. Phantom samples with varying concentrations of **PS9** in $CHCl_3$ (80, 40, 20, 10, 5, and 2.5 mM) were prepared. ^{19}F MR images were acquired with the following parameters: center frequency = 376.527883 MHz, repetition time (TR) = 4000 ms, echo time (TE) = 3 ms, field of view (FOV) = 30 mm × 30 mm, slice thickness (SI) = 20 mm, matrix size = 32 × 32, rare factor = 8, number of averages = 8, with a total acquisition time of 256 s.

5. Preparation, characterization, and properties of E1-E8

5.1 Preparation of E1-E8

The preparation of **E1** was used as a typical procedure: Phospholipid lipid S75 (60 mg), **F-oil** (100 mg), and **PS9** (2 mg) were dissolved in dichloromethane and evaporated under vacuum. Then, 3 mL of deionized water solution containing 30 mg of F68 was added, followed by ultrasonic treatment for 15 min. The mixture was then filtered through a 0.22 μm filter membrane to afford **E1**. Similarly, **E2**, **E3**, and **E5-E7** were prepared using the same procedure.

Preparation of **E4**: To a solution of Phospholipid lipid S75 (60 mg), soybean oil (100 mg), and **PS9** (1.5 mg) in dichloromethane, 3 mL of deionized water solution containing 30 mg of F68 was added. After stirred 4 h, the mixture was subjected to ultrasound treatment for 20 min and allowed dichloromethane to evaporate. Then, the mixture was processed with an ultrasonic cell disruptor for additional 5 min. **E4** was obtained by filtering through a 0.22 μm filter membrane, **E8** were prepared using the same procedure.

5.2 Detection the ROS generation of E1

The ROS production of **E1** was evaluated using DPBF and SOSG probes. **E1** (C_{PS9} = 1 μM) was mixed with 40 μM DPBF in deionized water, and the absorbance changes at 415 nm were recorded under 660 nm irradiation at 0.5 Wcm^{-2}. A mixture of **E1** (C_{PS9} =1 μM) and SOSG (3 μM) in deionized water was irradiated with a 660 nm laser (0.5 Wcm^{-2}) for varying durations, and the fluorescence spectra ($\lambda_{ex}/\lambda_{em}$ = 504 nm/525 nm) were recorded immediately after each irradiation.

The type of generated ROS was further identified using EPR. TEMPO was chosen as a singlet oxygen scavenger. A 100 μM solution of **E1** in deionized water was irradiated with a 660 nm laser (0.5 Wcm^{-2}) for 1 minute. TEMPO was then added to achieve a final concentration of 100 mM and mixed thoroughly for EPR measurements.

6. Cell culture and animal models

Cells were purchased from the Cell Bank of the Chinese Academy of Sciences (Shanghai, China) and cultured in Dulbecco's modified Eagle's medium (DMEM) supplemented with 10% fetal bovine serum (FBS) and 1% penicillin-streptomycin. The cells were incubated at 37°C in a humidified atmosphere containing 5% CO_2.

All animal experiments were conducted in accordance with the Guidelines for Animal Care and Use of Innovation Academy for Precision Measurement Science and Technology, Chinese Academy of Sciences (APM23042A). Female BALB/c nude mice, 5 weeks old, were purchased from Beijing Vital River Laboratory Animal Technology Co., Ltd. (Beijing, China) and fed with SPF-grade mouse chow. To establish the A549 tumor model, A549 cells (1×10^7) suspended in 0.1 mL of PBS was injected into the flank of female BALB/c nude mice. Tumor volume (V) was calculated using the formula: $V = (L/2) \times W^2$, where W is the width and L is the length of the tumor.

6.1 Intracellular uptake and localization

A549, HepG2, MCF-7, BEAS-2B, and MCF-10A cells were seeded into 35 mm confocal dishes at a density of about 1.5×10^5 cells per well and incubated overnight. For time-dependent uptake studies, the cells were incubated with **E1** for varying durations (0.5 h, 2 h, 6 h, and 12 h). Following incubation, the cells were washed three times with PBS and fixed with 4% PFA for 15 min. After staining with 200 μL of DAPI for 10 min, images were captured using CLSM.

For colocalization studies, the cells were incubated with **E1** for 6 h, and then stained with DiI, Mito-Tracker Green, Lyso-Tracker Green, or ER-Tracker Blue-White DPX. Cells were then washed three times with PBS, and fresh PBS was added before visualization with CLSM.

The excitation wavelengths were as follows: **E1**: λ_{ex} = 640 nm. DiI: λ_{ex} = 561 nm. Mito-Tracker: λ_{ex} = 488 nm. Lyso-Tracker: λ_{ex} = 488 nm. ER-Tracker: λ_{ex} = 405 nm. The collected FL images were analyzed using ImageJ software.

6.2 In vitro cytotoxicity assays of E1

A549, HepG2, MCF-7, BEAS-2B, and MCF-10A cells were seeded at 1×10^4 cells per well in a 96-well cell culture plate and cultured at 37 °C for 24 h. Then, the cells were incubated with varying concentrations of **E1** for 6 h and washed with 100 µL fresh medium. They were subsequently irradiated with a 660 nm laser (0.5 Wcm^{-2}, 10 min) and allowed to grow for an additional 24 h. Afterward, 100 µL of fresh medium containing 10% CCK-8 were added to each well, and the cells were incubated for 1 h at 37 °C. Finally, the absorbance at 450 nm was measured using a microplate reader to assess the cell viability. For the dark toxicity measurement of the photosensitizers, the same procedure was followed without laser irradiation.

6.3 Live/dead cell staining assays of E1

A549 cells were seeded onto 35 mm confocal dishes at a density of 1×10^5 cells and incubated with **E1** ($C_{\mathbf{PS9}}$ = 8 µM) for 6 h. Following exposure to a 660 nm laser (0.5 Wcm^{-2}) for 10 min, the cells were further cultured for 6 h and stained with AM and PI for 30 min. Finally, Confocal fluorescence imaging was performed. To assess the dark toxicity of A549 cells, the same procedure was followed without laser irradiation.

6.4 Intracellular ROS imaging in A549 cells of E1

DCFH-DA was utilized to detect ROS generation in A549 cells. The cells were plated onto 35 mmol confocal dishes and incubated with **E1** ($C_{\mathbf{PS9}}$ =8 µM) for 6 h, followed by staining with 10 µM DCFH-DA for 30 min. After washing three times with PBS, the laser-treated group was irradiated with a 660 nm laser (0.5 Wcm^{-2}) for 10 min. Confocal fluorescence imaging was then performed (λ_{ex} = 488 nm).

6.5 In vitro endocytosis pathway study of E1

A549 cells were seeded in 35 mm confocal dishes and incubated at 37 °C for 24 h. The cells were then treated with either 800 µM amiloride, 400 µM genistein, or 0.45 M sucrose for 30 min followed by incubation with **E1** for 6 h. To investigate the influence of temperature, some cells were kept at 4°C for 6 h during treatment. Following incubation, the cells were washed three times with PBS and fixed with 4% PFA for 15 min. After staining with 200 µL of DAPI for 10 min, images were captured using CLSM. The collected FL images were then analyzed using ImageJ software.

6.6 LDH release assays

A549 cells were seeded at a density of 1×10^4 cells per well in a 96-well cell culture plate and cultured for 24 h. Following this, the cells were incubated with various nanoemulsions for 6 h and then washed with 100 µL of fresh medium. The laser-treated group was irradiated with a 660 nm laser (0.5 Wcm^{-2}) for 10 min and allowed to grow for an additional 24 h. LDH release was measured according to the manufacturer's instructions.

6.7 Intracellular lipid peroxidation (LPO) detection

Intracellular lipid peroxidation levels were assessed using C11-BODIPY 581/591 as a probe. A549 cells were seeded in 35 mm confocal dishes at 37°C for 24 h and incubated with **E1** for 6 h. After exposure to a 660 nm laser (0.5 Wcm^{-2}, 10 min), the cells were cultured for an additional 12 h and then stained with C11-BODIPY 581/591 and DAPI for 30 min. Images were captured using CLSM. The production of MDA was evaluated using the MDA Assay Kit. A549 cells were plated onto a

6-well plate and incubated at 37°C for 24 h. After incubation with different nanoemulsions for 6 h and washing with 1 mL of fresh medium, the laser-treated group was irradiated with a 660 nm laser (0.5 Wcm^{-2}, 10 min) and allowed to continue growing for 24 h. Intracellular lipid peroxidation levels were measured according to the manufacturer's instructions.

6.8 Evaluation of glucose-regulated protein 78 (GRP78)

A549 cells were plated onto 35 mm confocal dishes and incubated at 37°C for 24 h. The cells were then treated with **E1** (C_{PS9} =8 μM) for 6 h and then washed with 1 mL of fresh medium. The laser-treated group was irradiated with a 660 nm laser (0.5 Wcm^{-2}) for 10 min and allowed to grow for an additional 24 h. Afterward, the cells were washed three times with PBS and fixed with 4% PFA for 15 min. Following another wash with buffer, the cells were treated with immunol staining blocking buffer for 1 h and then incubated with GRP78 Rabbit Polyclonal Antibody at 37 °C for 1 h. The cells were subsequently stained with Alexa Fluor 488-labeled Goat Anti-Rabbit IgG(H+L) for 1 h and washed three times with buffer. Finally, after staining with 200 μL of DAPI for 10 min, images were obtained using CLSM (λex = 488 nm).

6.9 In vivo ^{19}F MRI

For in vivo ^{19}F MRI, the mice bearing A549 tumors were intravenously injected with 100 μL of **E1** (C_{PS9} = 2.5 mg/kg, C_F = 6 mmol/kg). ^{19}F MRI was performed using the RARE sequence with the following parameters: center frequency = 376.527883 MHz, TR = 4000 ms, TE = 3 ms, FOV = 37 mm × 37 mm, SI = 15 mm, matrix size = 32 × 32, rare factor = 4, and number of averages = 64.

^{1}H MR images were acquired for the localization of the mice using the RARE sequence with the following parameters: TR = 1500 ms, TE = 11 ms, FOV = 30 mm × 30 mm, SI = 1 mm, matrix size = 256 × 256, rare factor = 8, and number of averages = 4.

6.10 In vivo therapeutic efficacy evaluation

When the subcutaneous tumors reached approximately 100 mm^3, the mice were randomly divided into four groups: (a) saline + L; (b) **PS9** (0.8 mg/kg) + L; (c) **E1** (C_{PS9} = 0.8 mg/kg, C_F = 1.8 mmol/kg); (d) **E1** (C_{PS9} = 0.8 mg/kg, C_F = 1.8 mmol/kg) + L. Mice received intravenous injections of **E1** on days 0, 4 and 8, followed by irradiation with a 660 nm laser (0.5 Wcm^{-2}, 10 min) 1 h post-injection. The body weight and tumor volume of mice were recorded every 2 days. After 20 days of treatment, the mice were euthanized, and the major organs and tumors were harvested for analysis, including H&E staining, Ki-67 staining, and TUNEL staining.

7. Statistical analysis

Data are presented as mean ± standard deviation from n ≥ 3 replicates. Asterisks indicate significant differences (*$p < 0.05$, **$p < 0.01$, ***$p < 0.001$) as determined by an unpaired Student’s two-sided t-test.

8. $^{1}H/^{19}F/^{13}C$ NMR and HRMS spectra of compounds

^{1}H NMR (400 MHz) of **A1** in $CDCl_3$

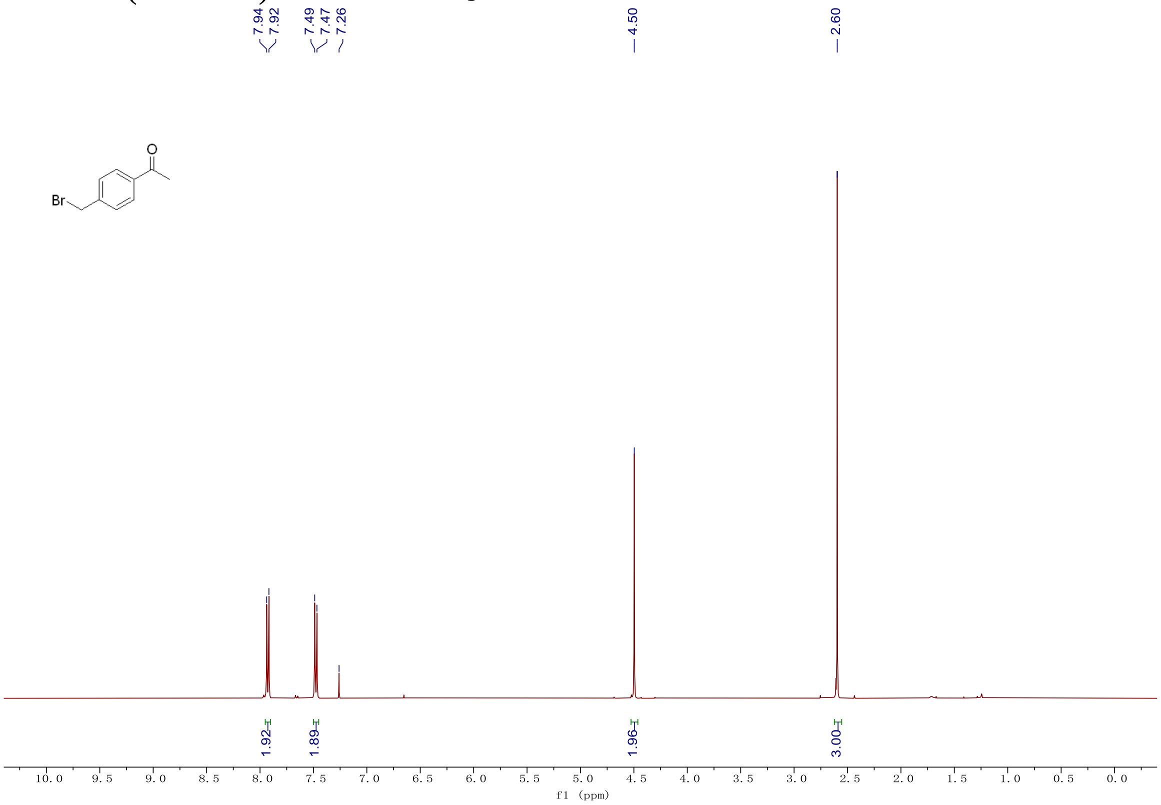

^{1}H NMR (500 MHz) of **A2** in $CDCl_3$

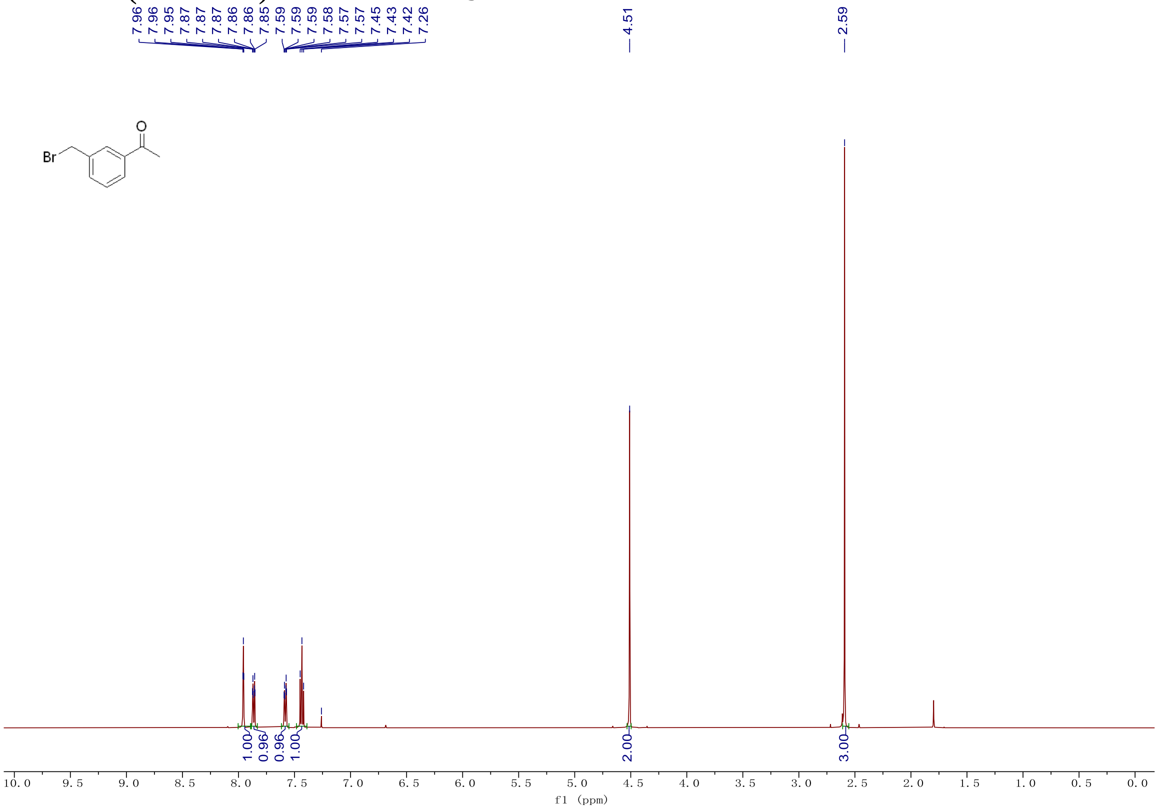

^{1}H NMR (500 MHz) of **A3** in $CDCl_3$

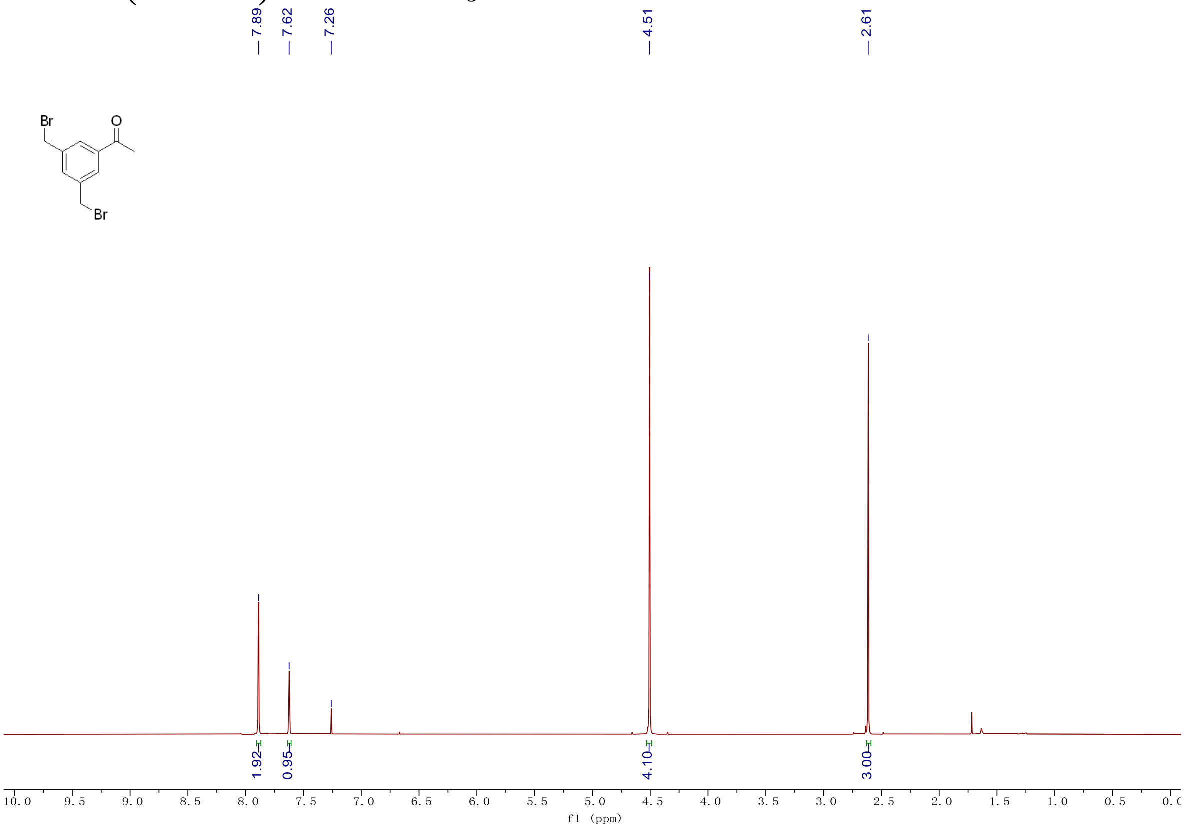

^{1}H NMR (500 MHz) of **A4** in $CDCl_3$

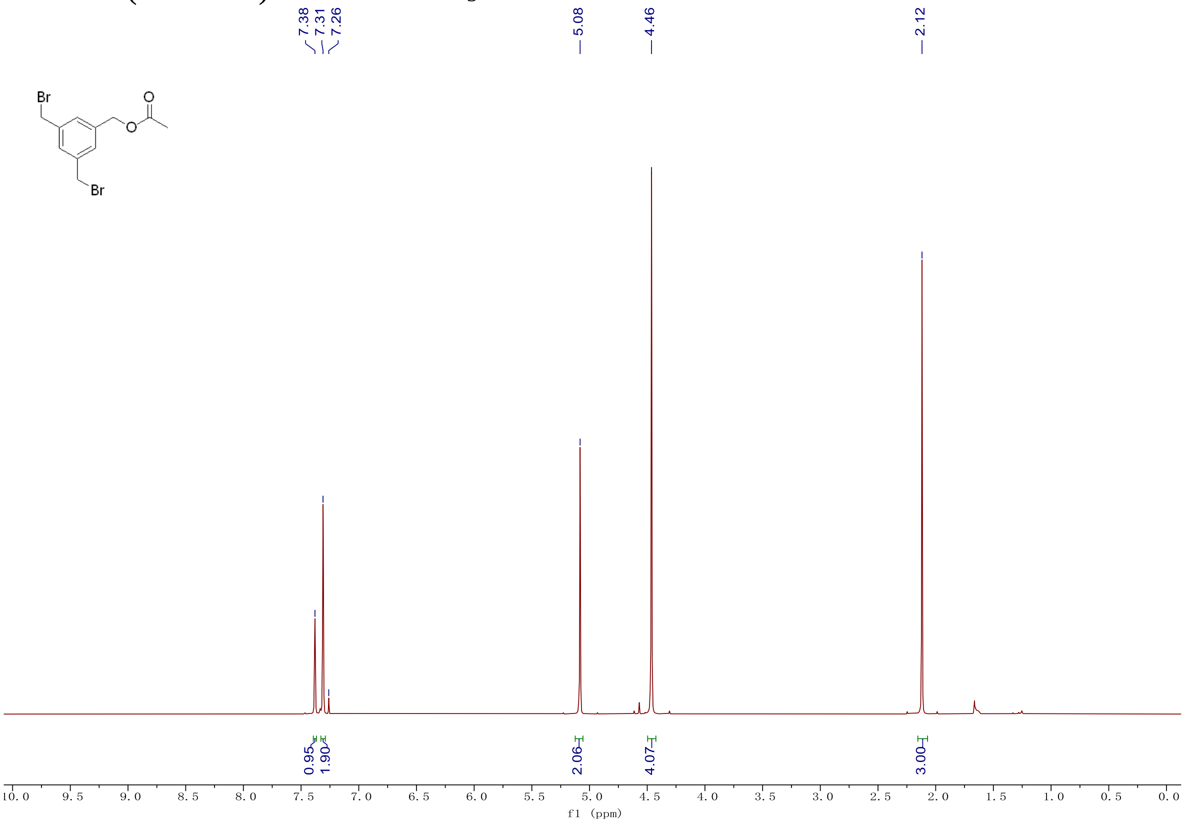

^{1}H NMR (500 MHz) of **A5** in $CDCl_3$

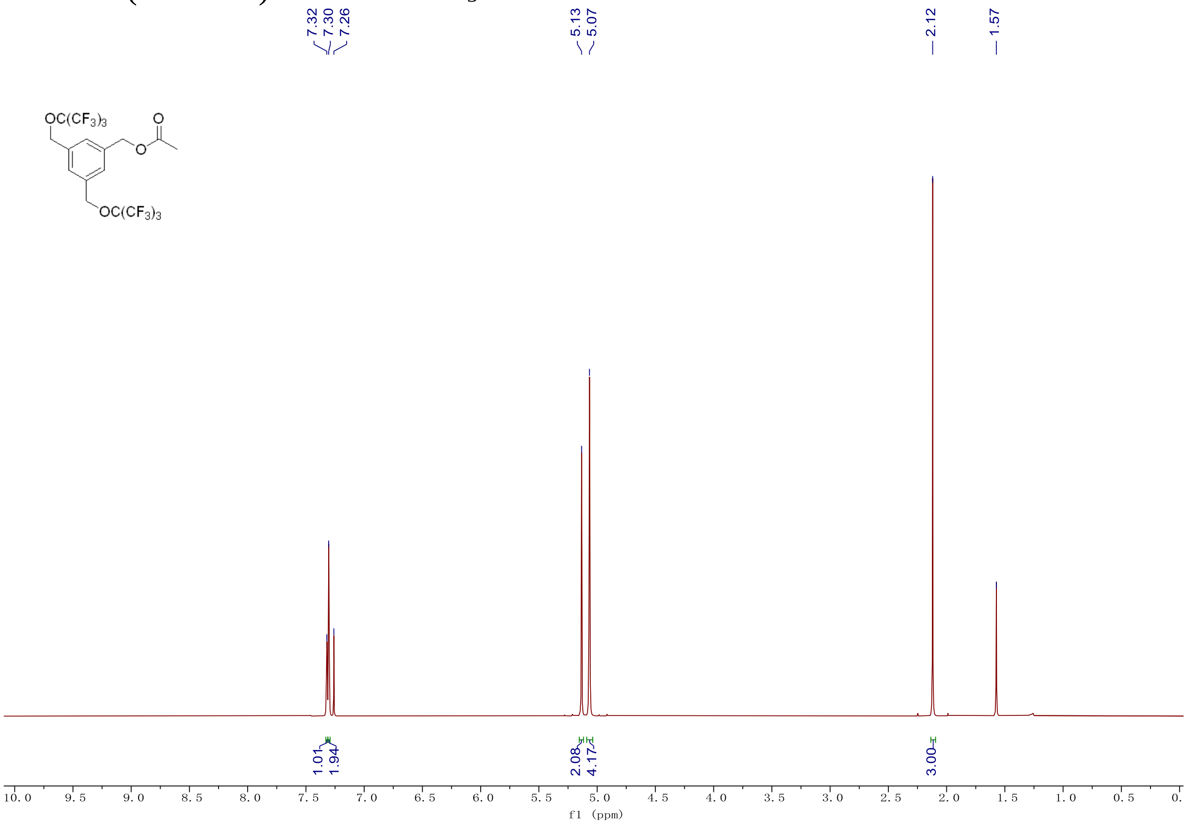

^{19}F NMR (471 MHz) of **A5** in $CDCl_3$

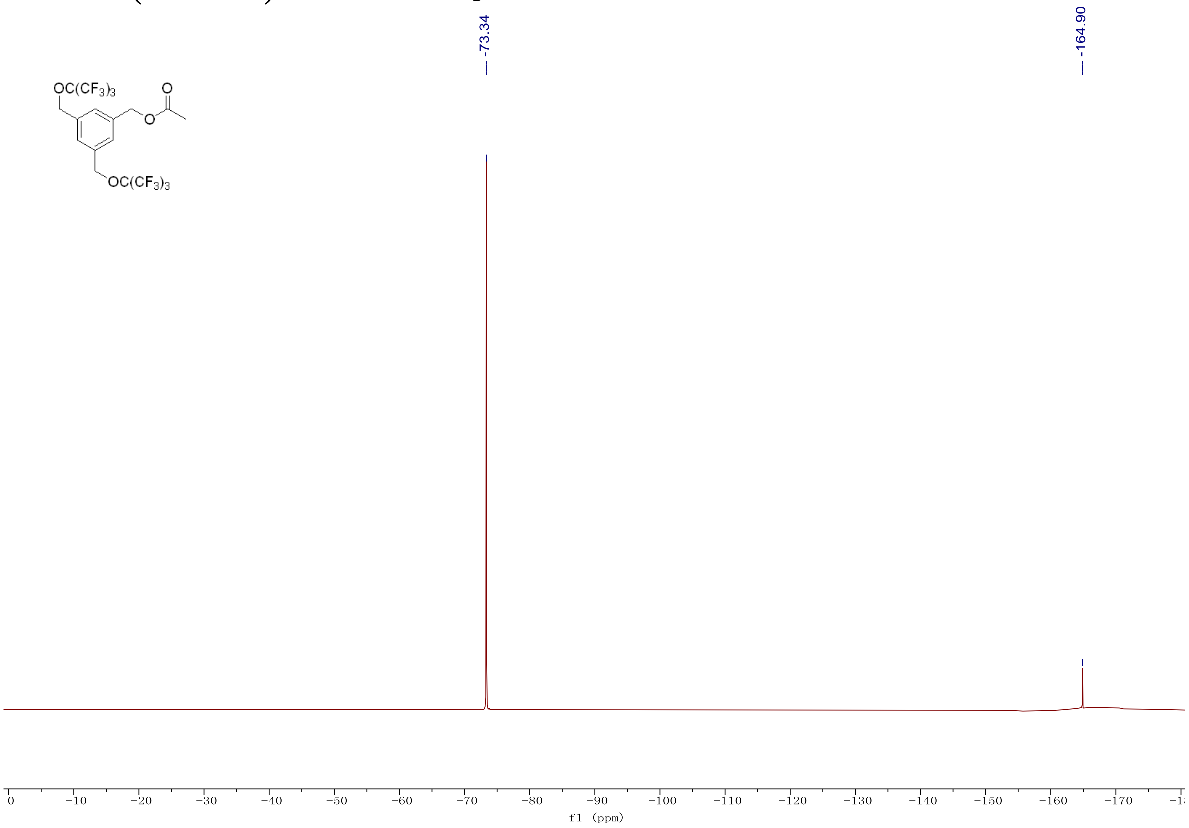

^{13}C NMR (126 MHz) of **A5** in $CDCl_3$

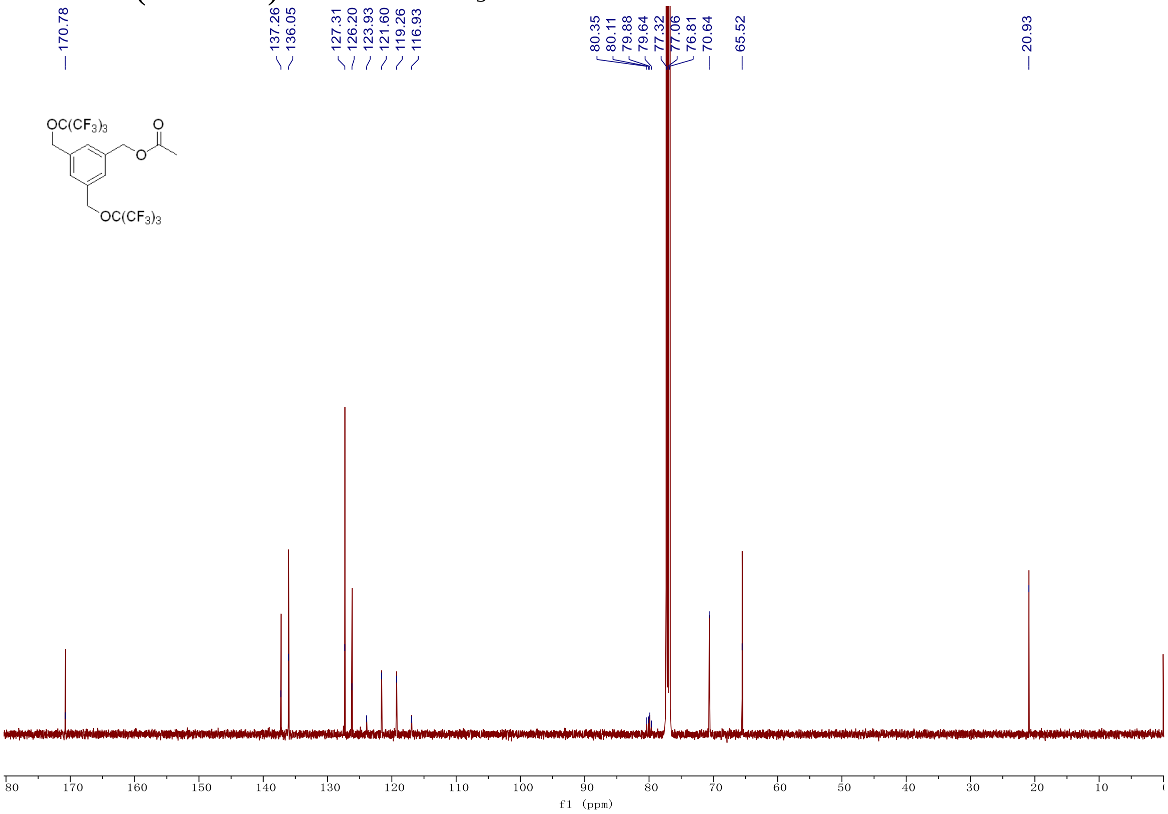

^{1}H NMR (600 MHz) of **A6** in $CDCl_3$

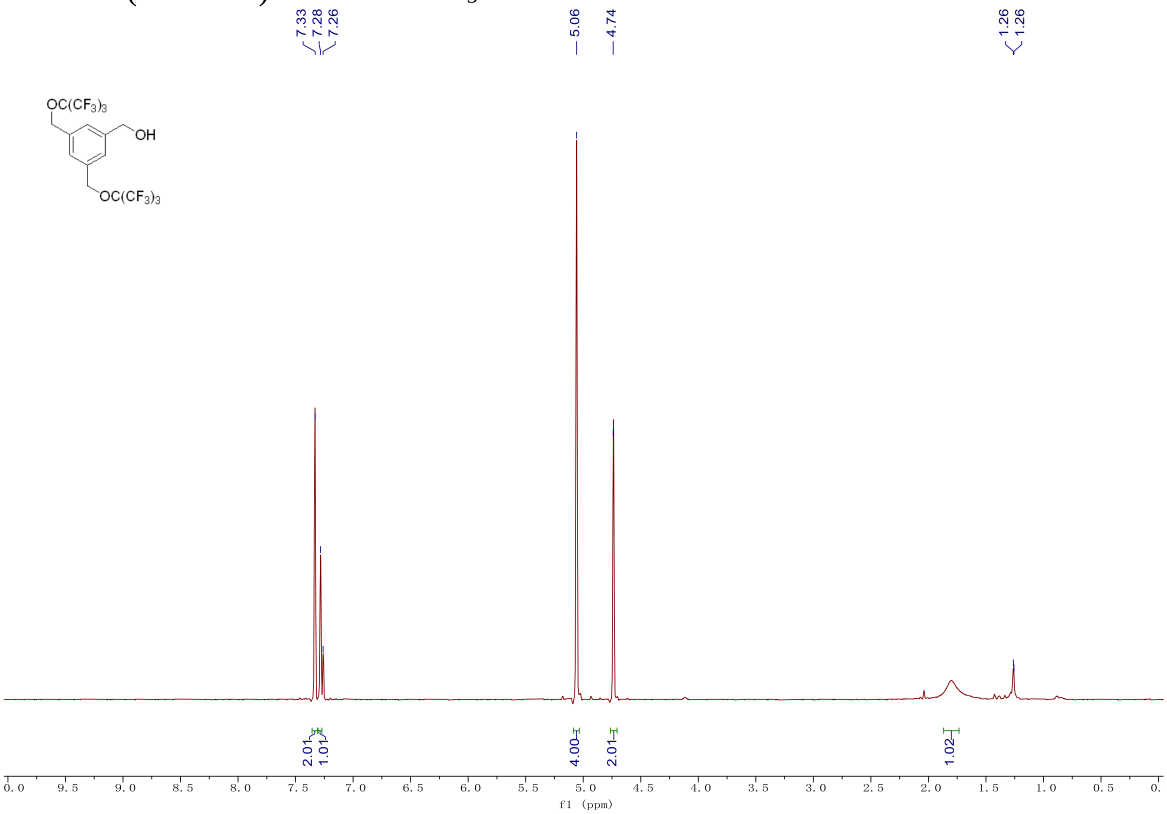

^{19}F NMR (471 MHz) of **A6** in $CDCl_3$

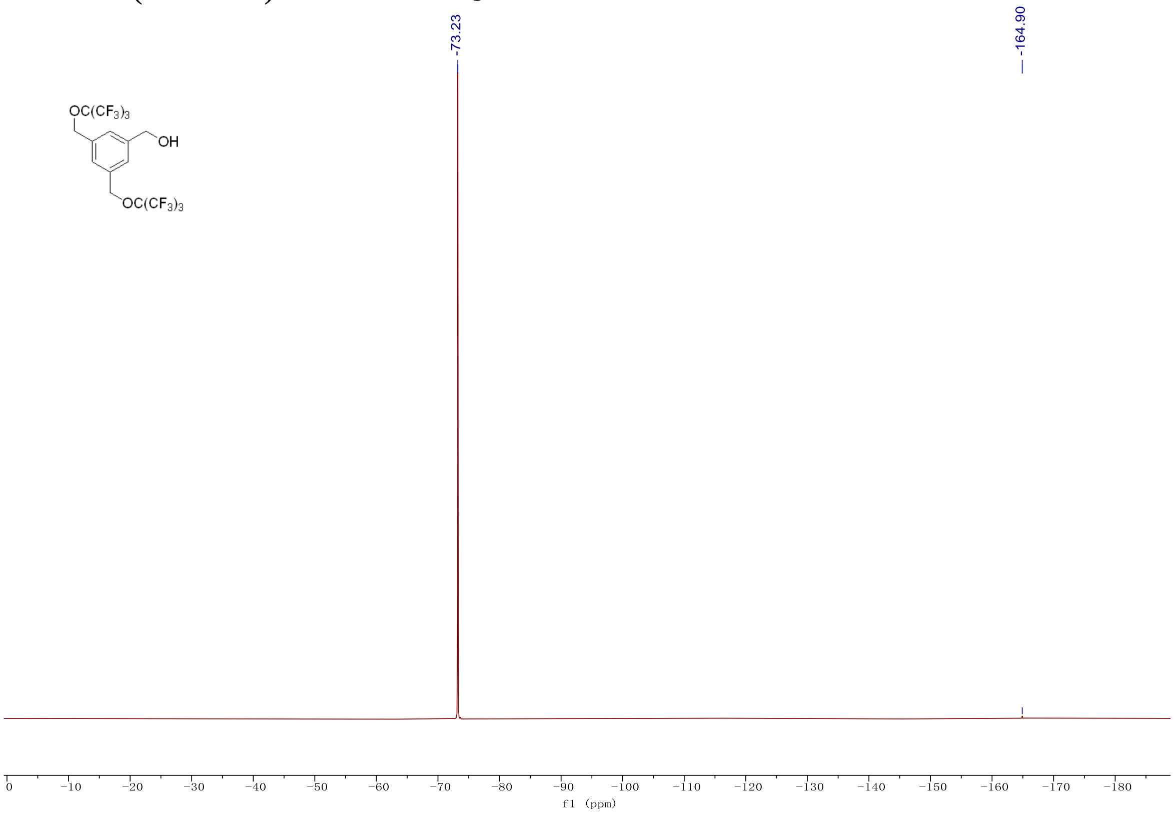

^{13}C NMR (126 MHz) of **A6** in $CDCl_3$

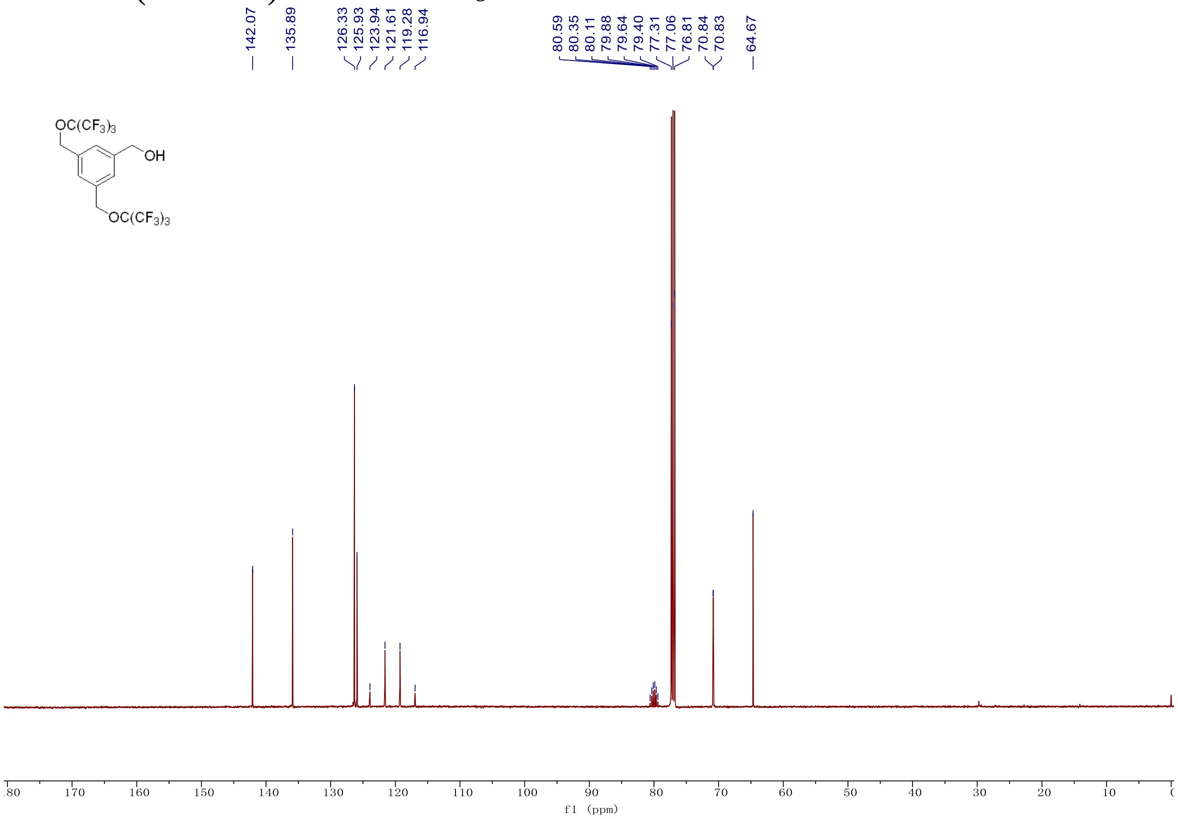

^{1}H NMR (400 MHz) of **B1** in $CDCl_3$

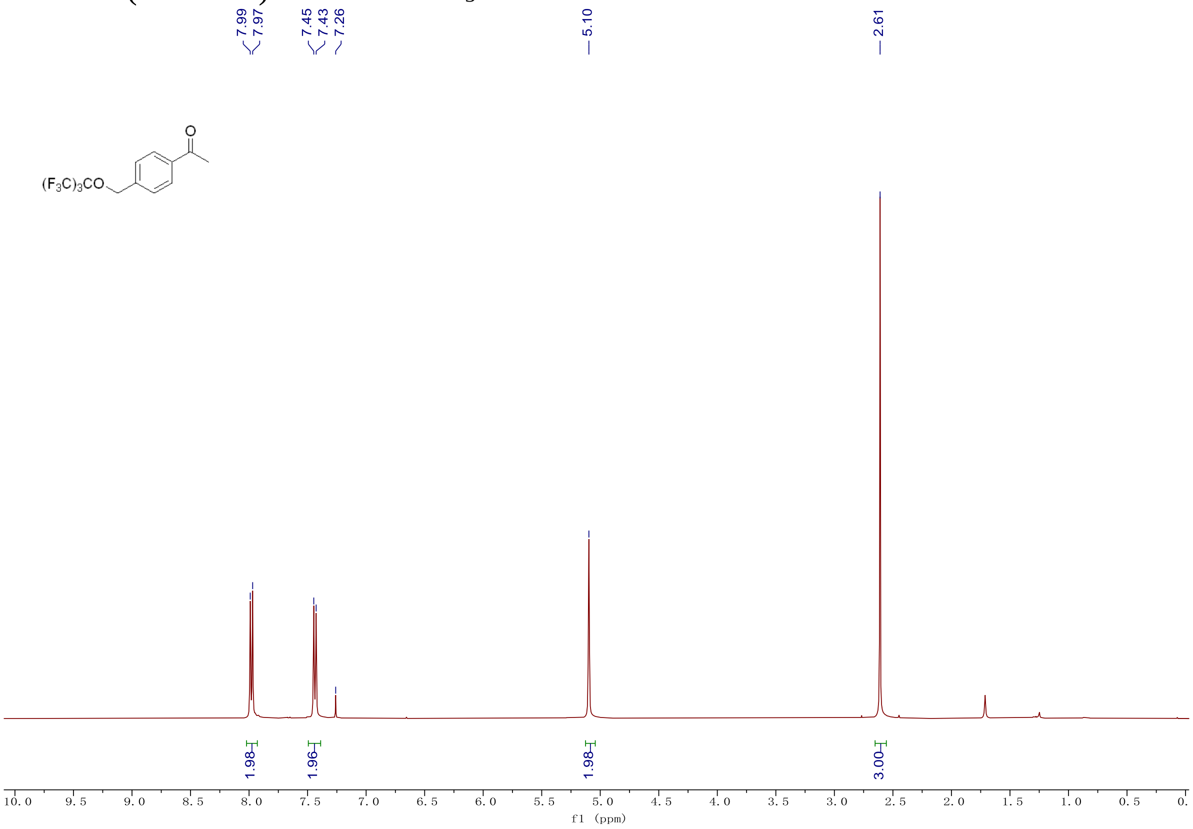

^{19}F NMR (376 MHz) of **B1** in $CDCl_3$

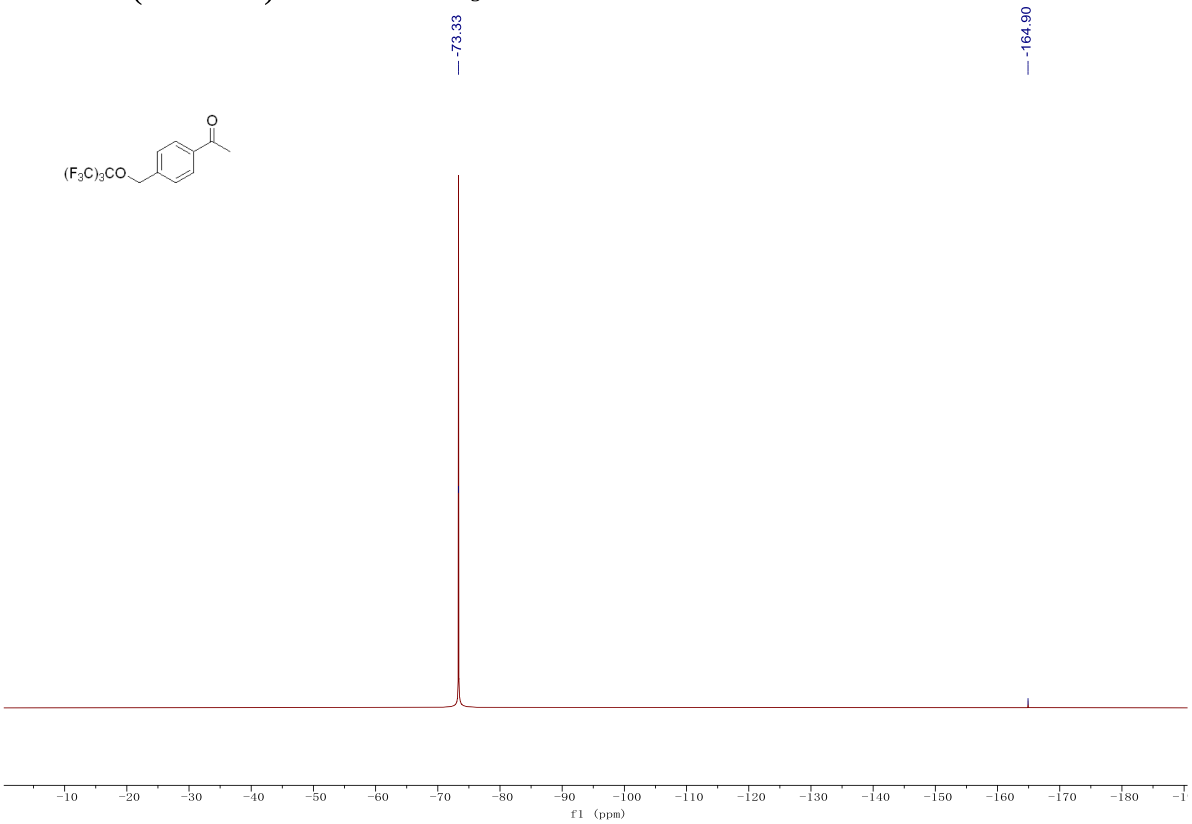

^{13}C NMR (126 MHz) of **B1** in $CDCl_3$

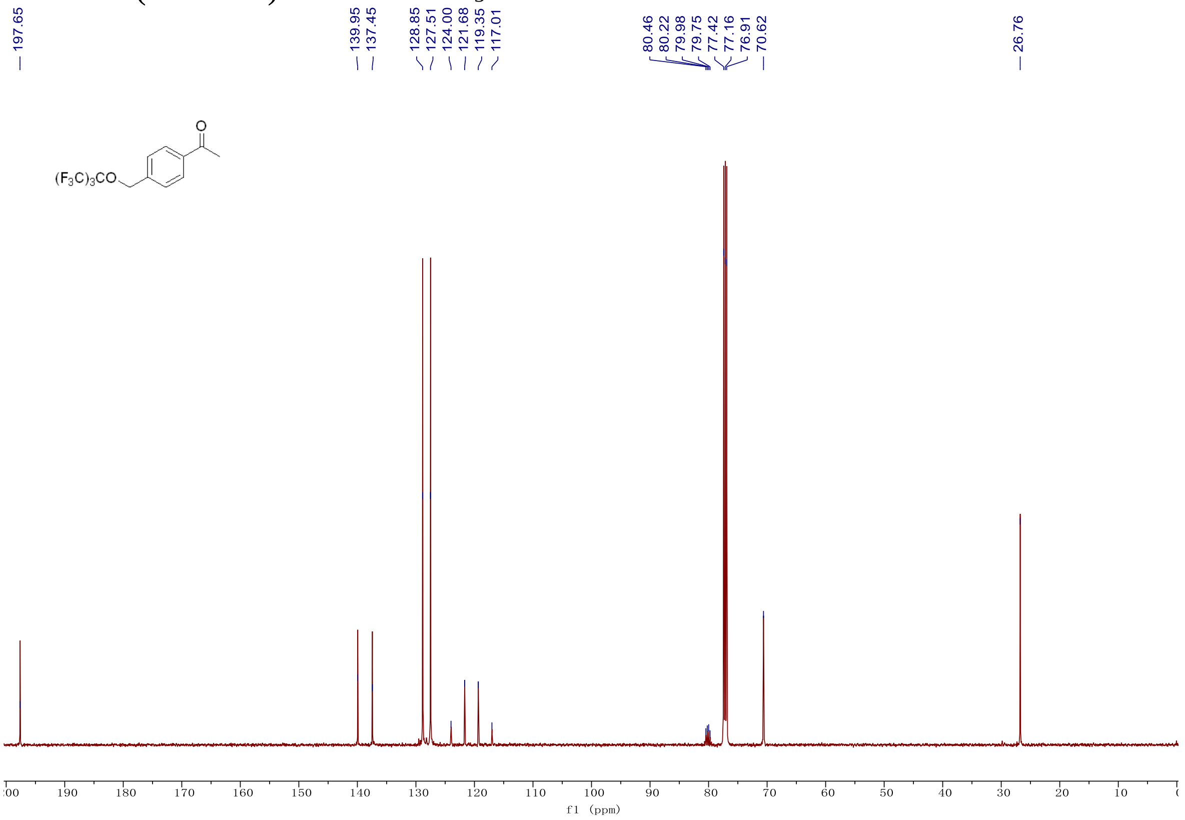

^{1}H NMR (500 MHz) of **B2** in $CDCl_3$

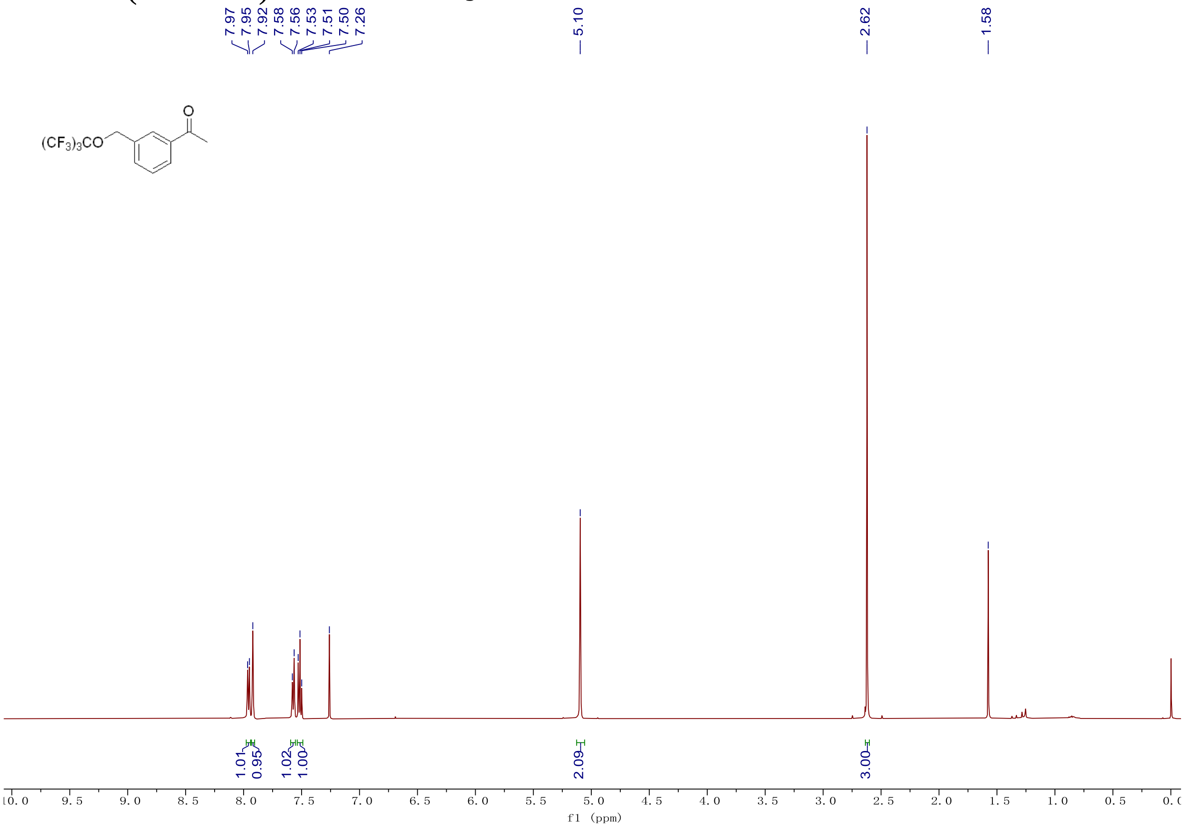

^{19}F NMR (471 MHz) of **B2** in $CDCl_3$

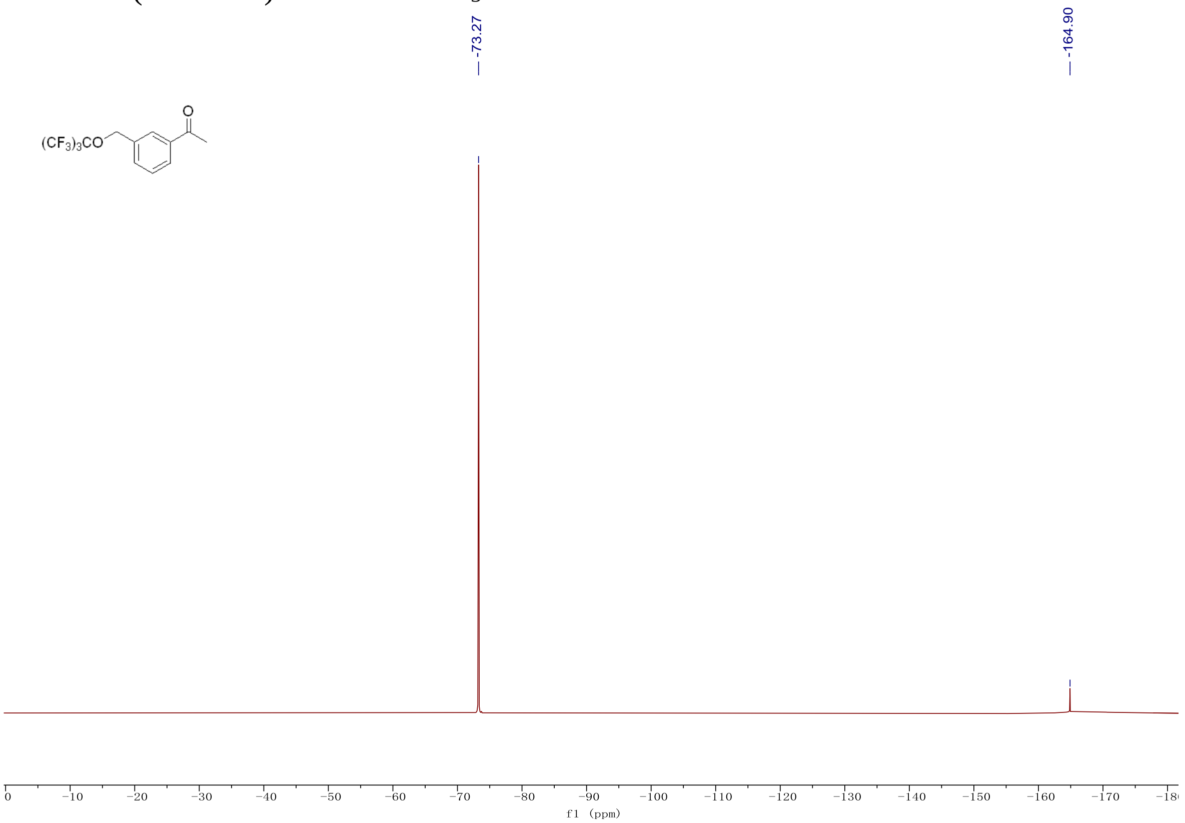

^{13}C NMR (126 MHz) of **B2** in $CDCl_3$

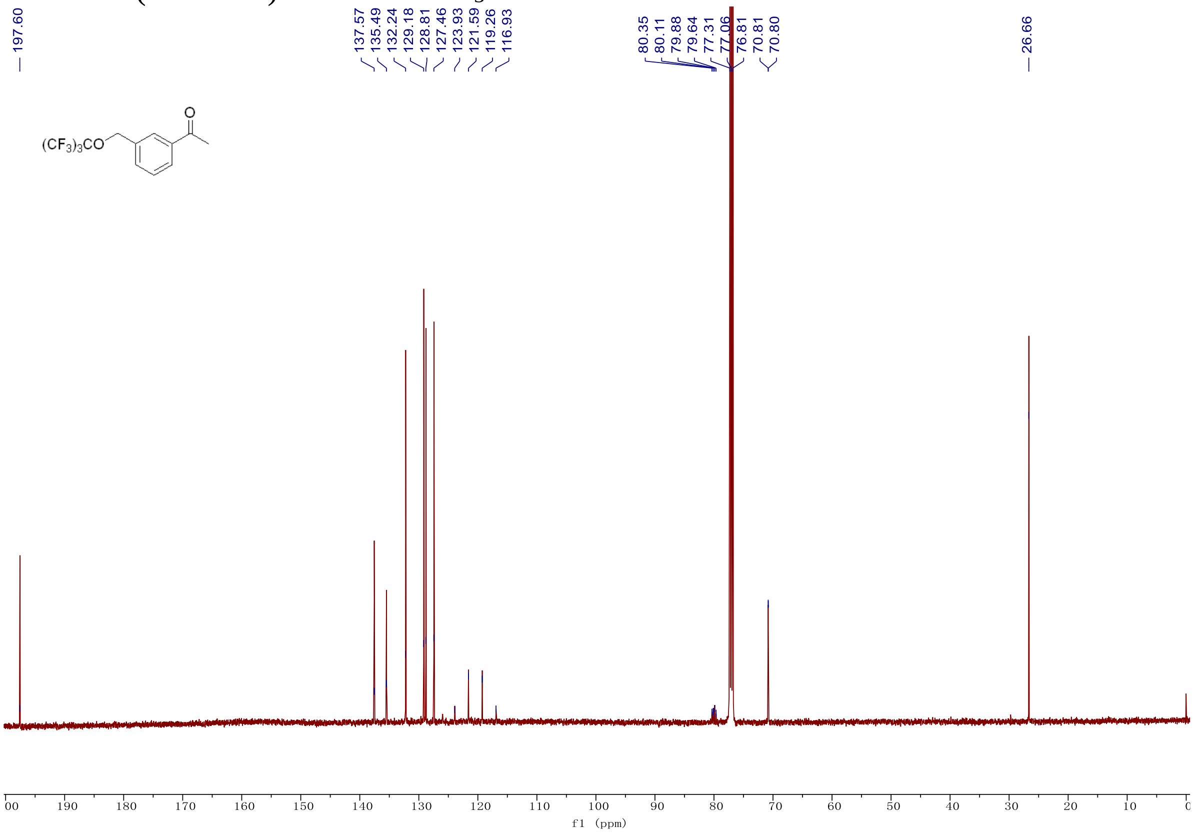

^{1}H NMR (600 MHz) of **B3** in $CDCl_3$

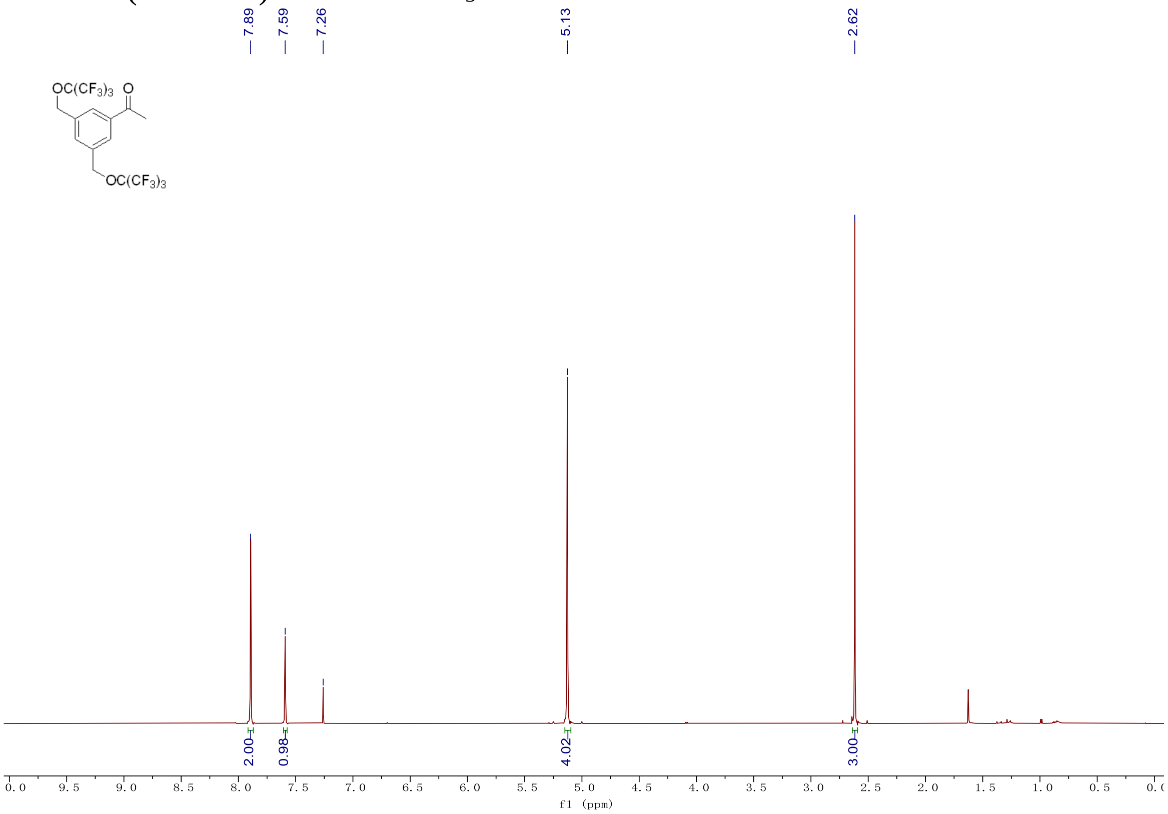

^{19}F NMR (471 MHz) of **B3** in $CDCl_3$

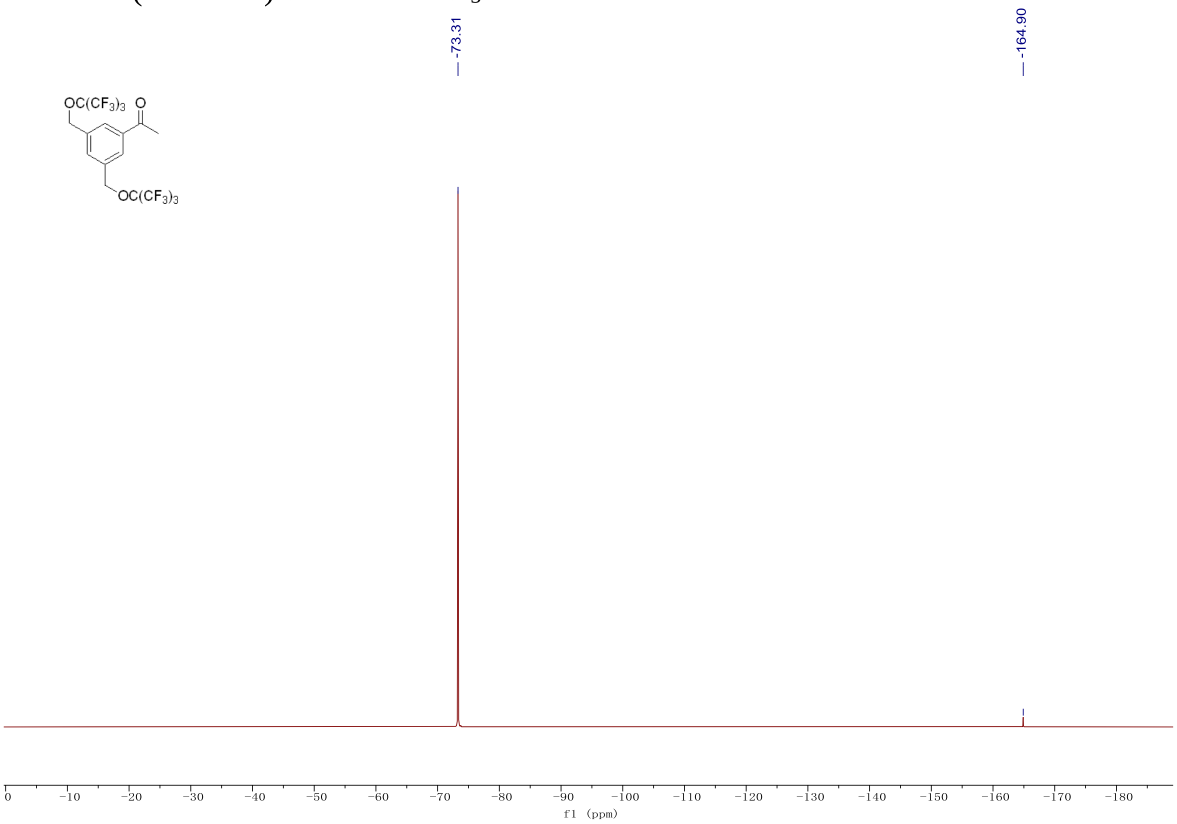

^{13}C NMR (126 MHz) of **B3** in $CDCl_3$

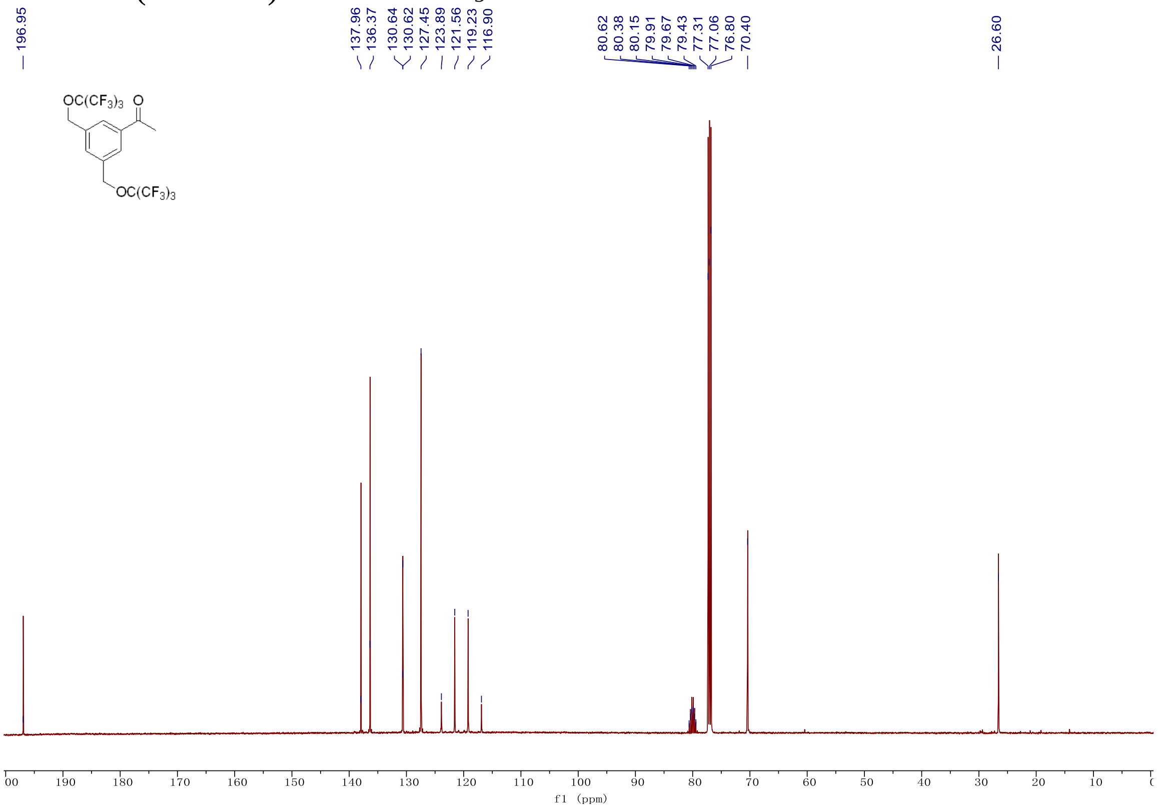

^{1}H NMR (400 MHz) of **C1** in $CDCl_3$

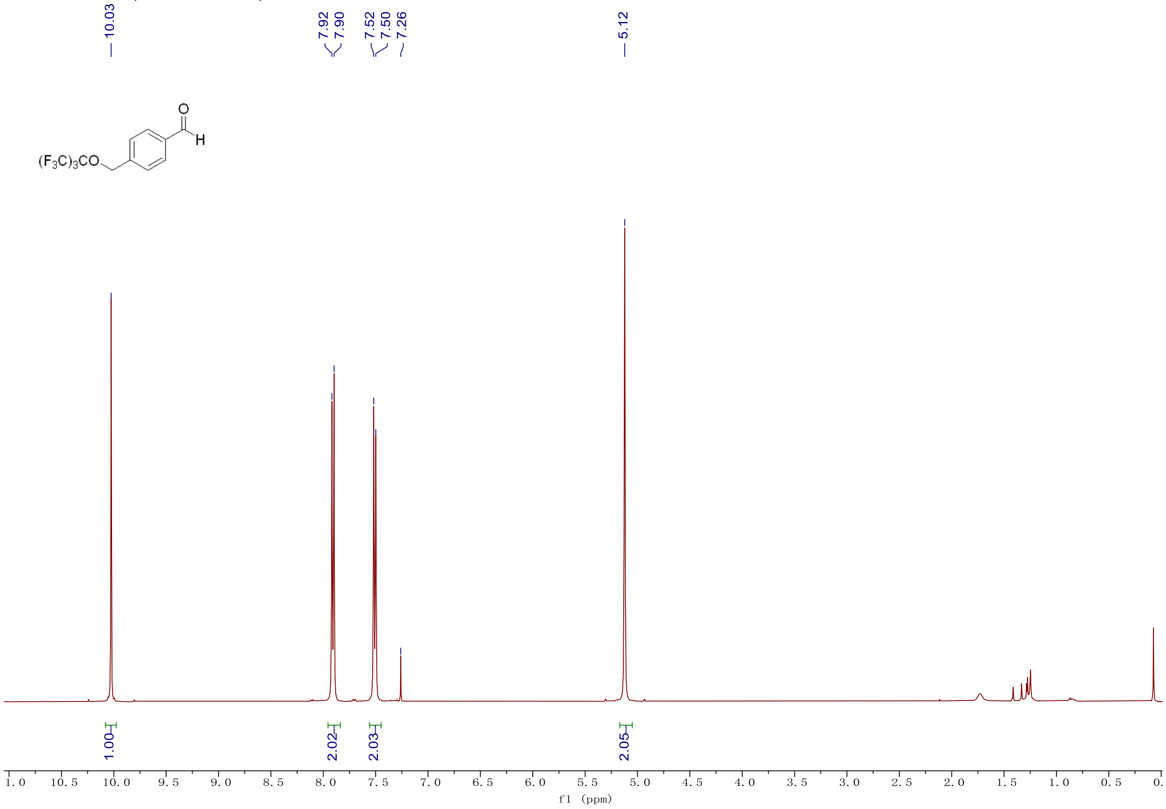

^{19}F NMR (376 MHz) of **C1** in $CDCl_3$

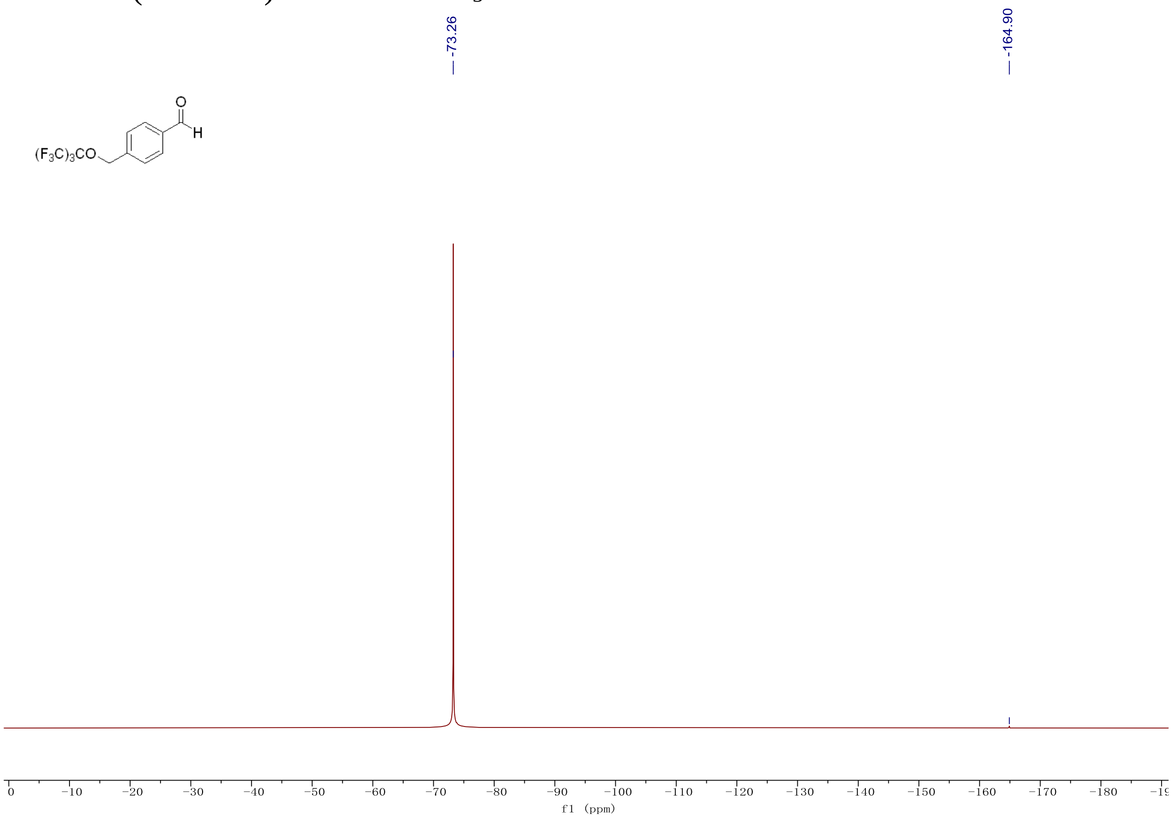

^{13}C NMR (101 MHz) of **C1** in $CDCl_3$

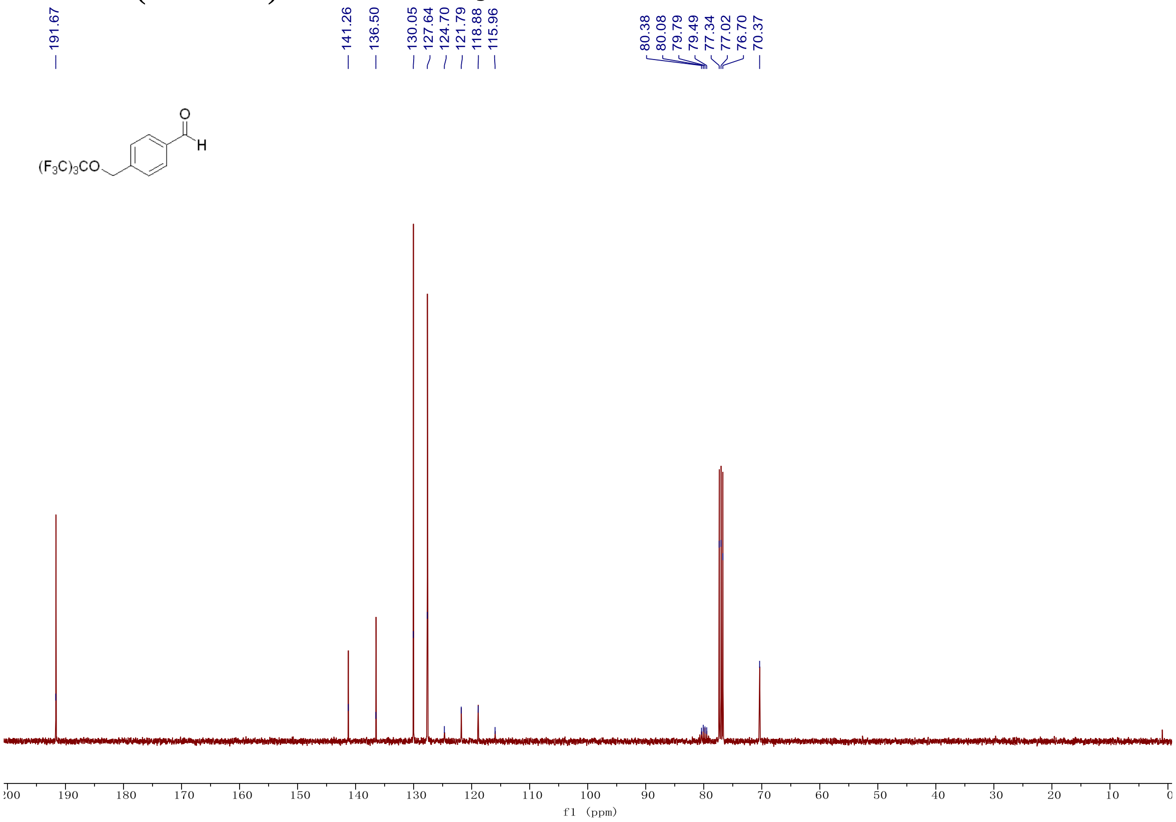

^{1}H NMR (500 MHz) of **C2** in $CDCl_3$

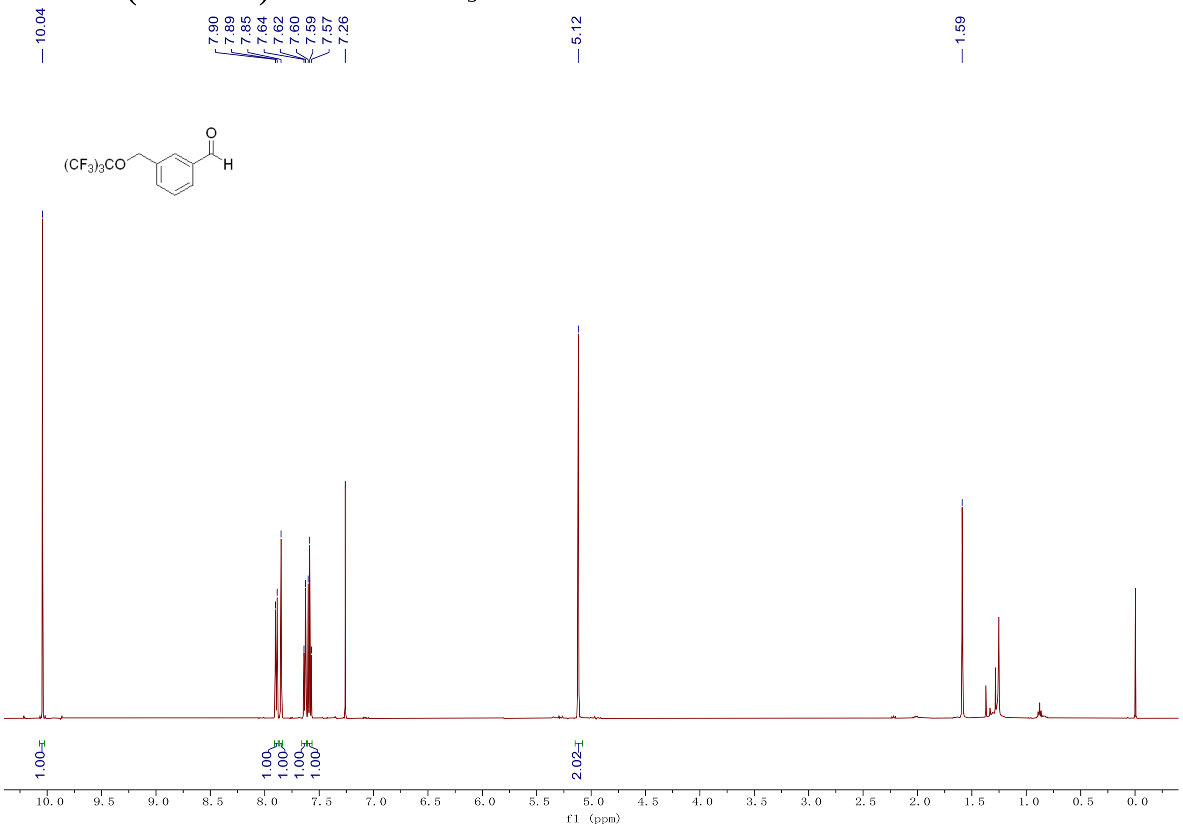

^{19}F NMR (471 MHz) of **C2** in $CDCl_3$

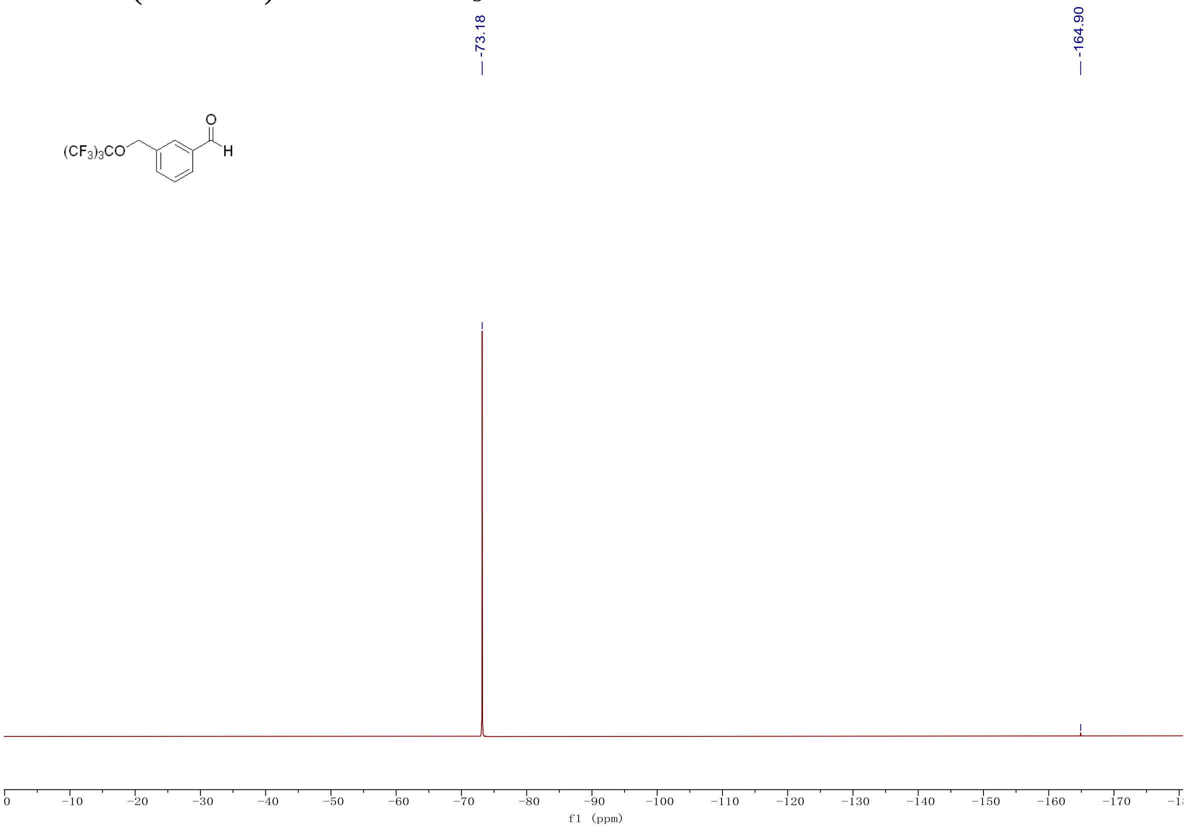

^{13}C NMR (126 MHz) of **C2** in $CDCl_3$

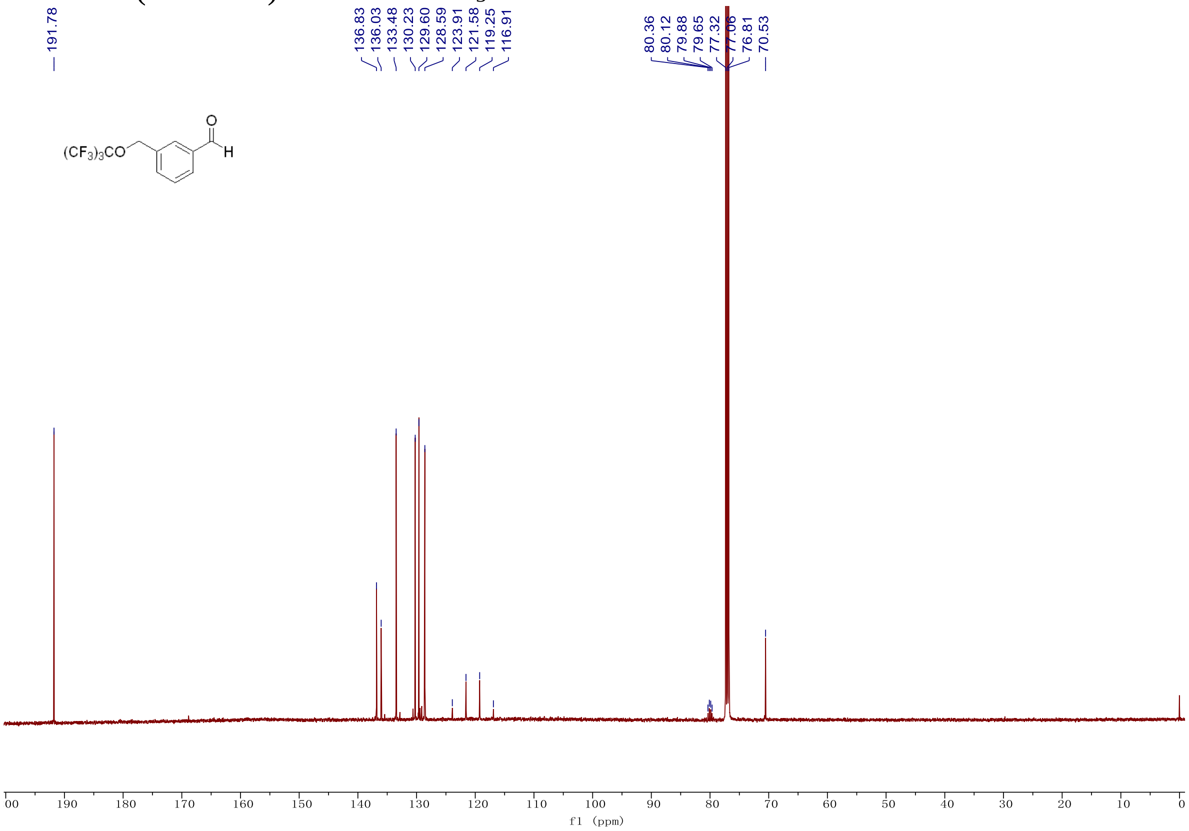

^{1}H NMR (500 MHz) of **C3** in $CDCl_3$

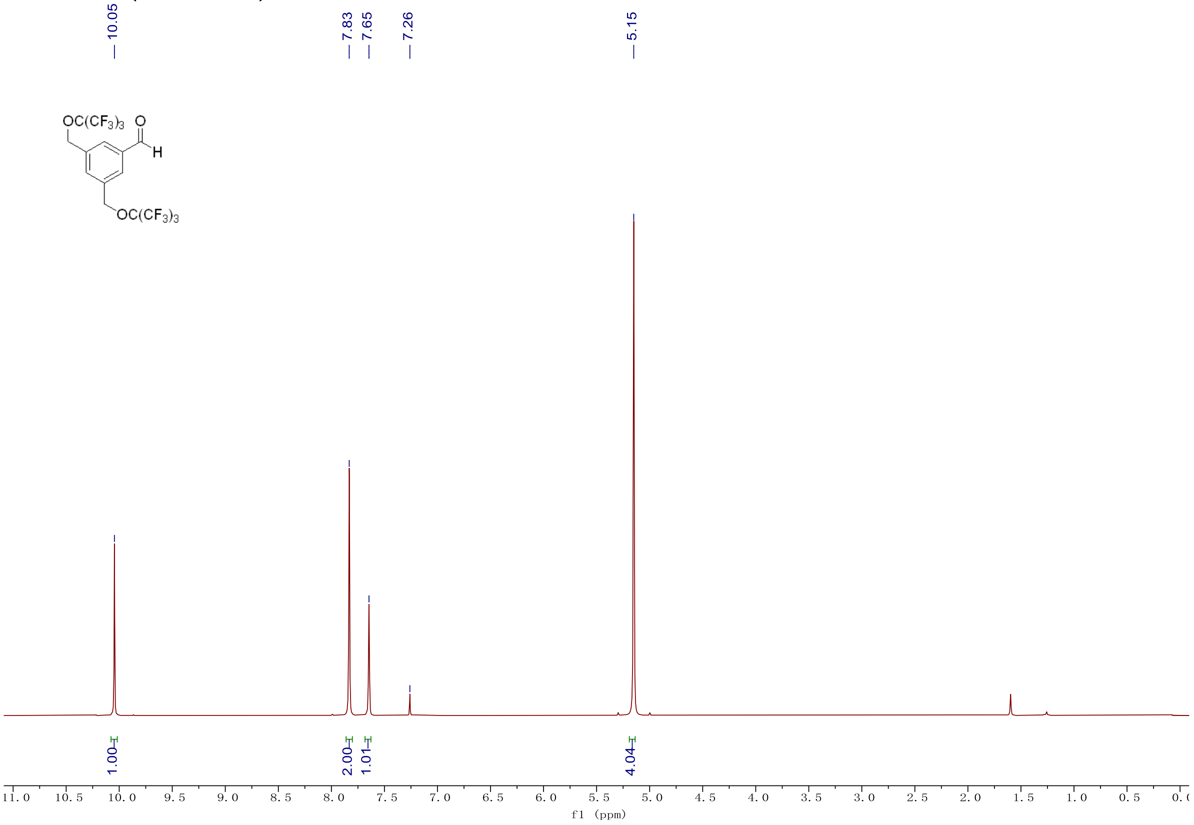

^{19}F NMR (471 MHz) of **C3** in $CDCl_3$

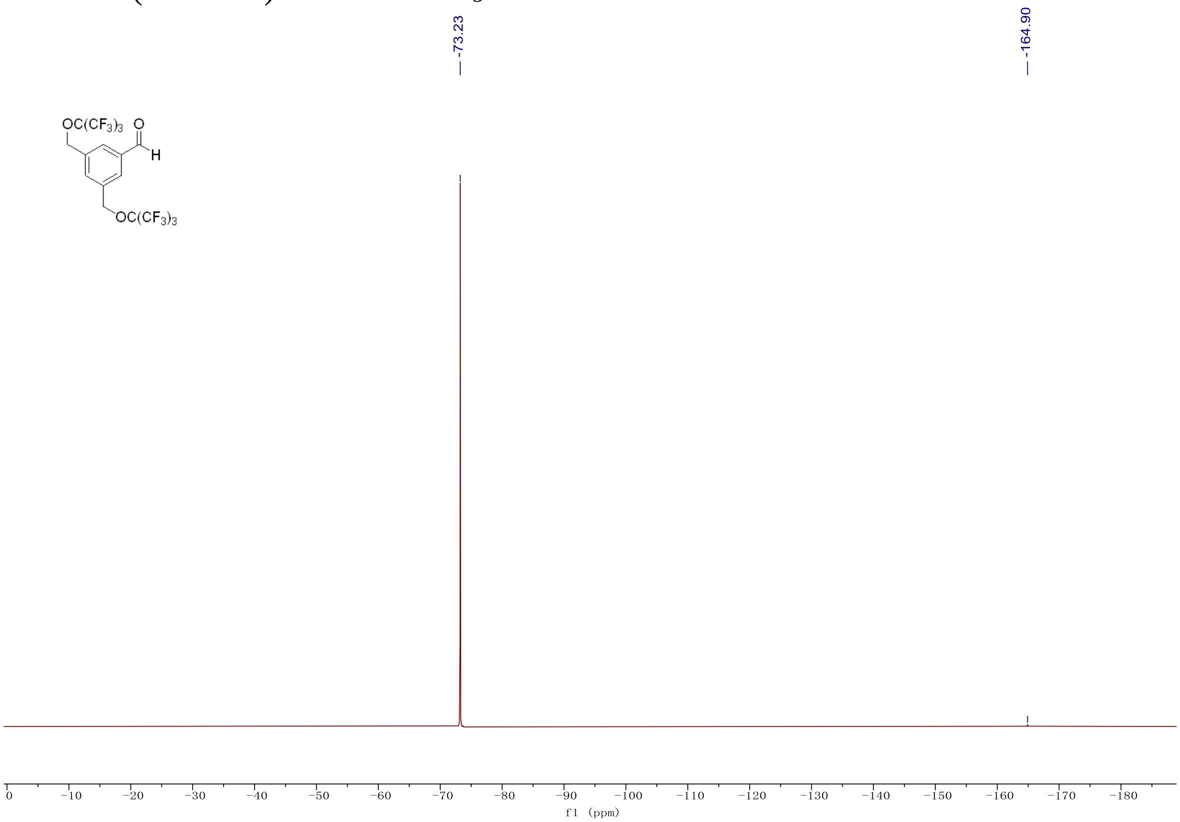

^{13}C NMR (126 MHz) of **C3** in $CDCl_3$

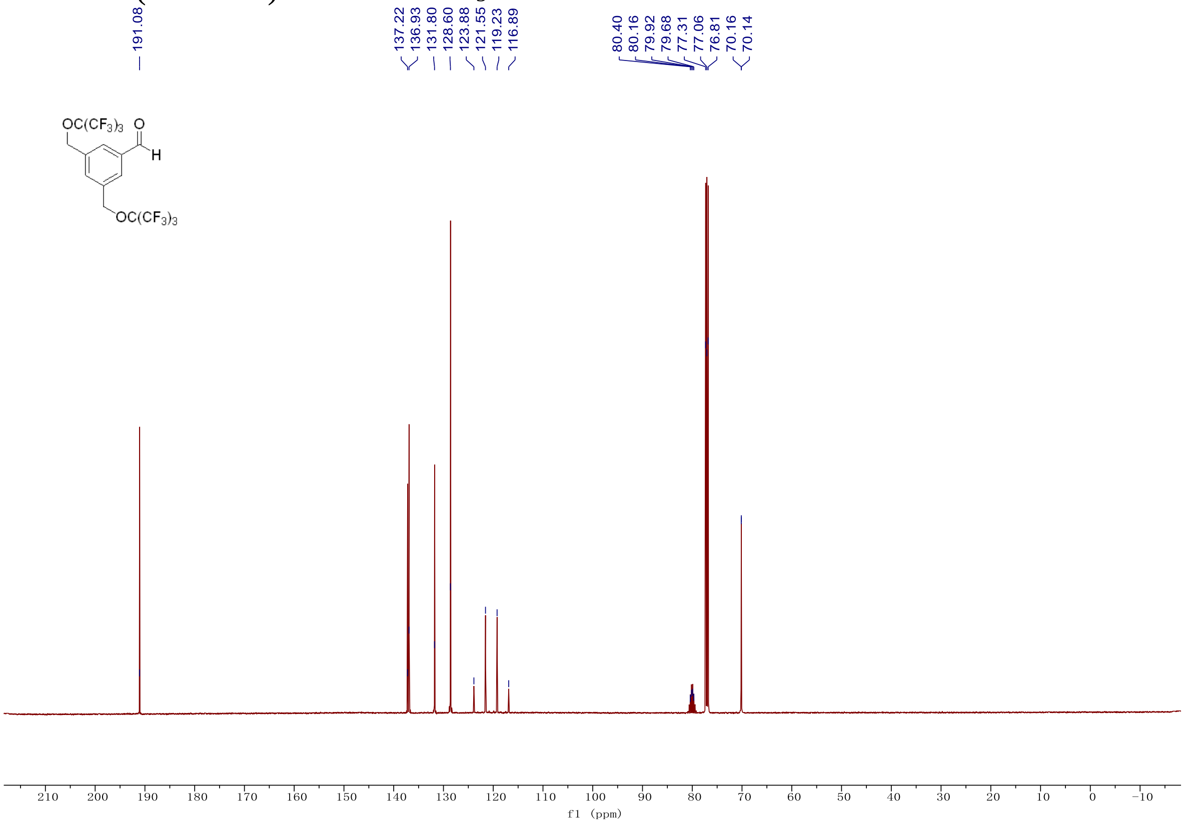

^{1}H NMR (400 MHz) of **1a** in $CDCl_3$

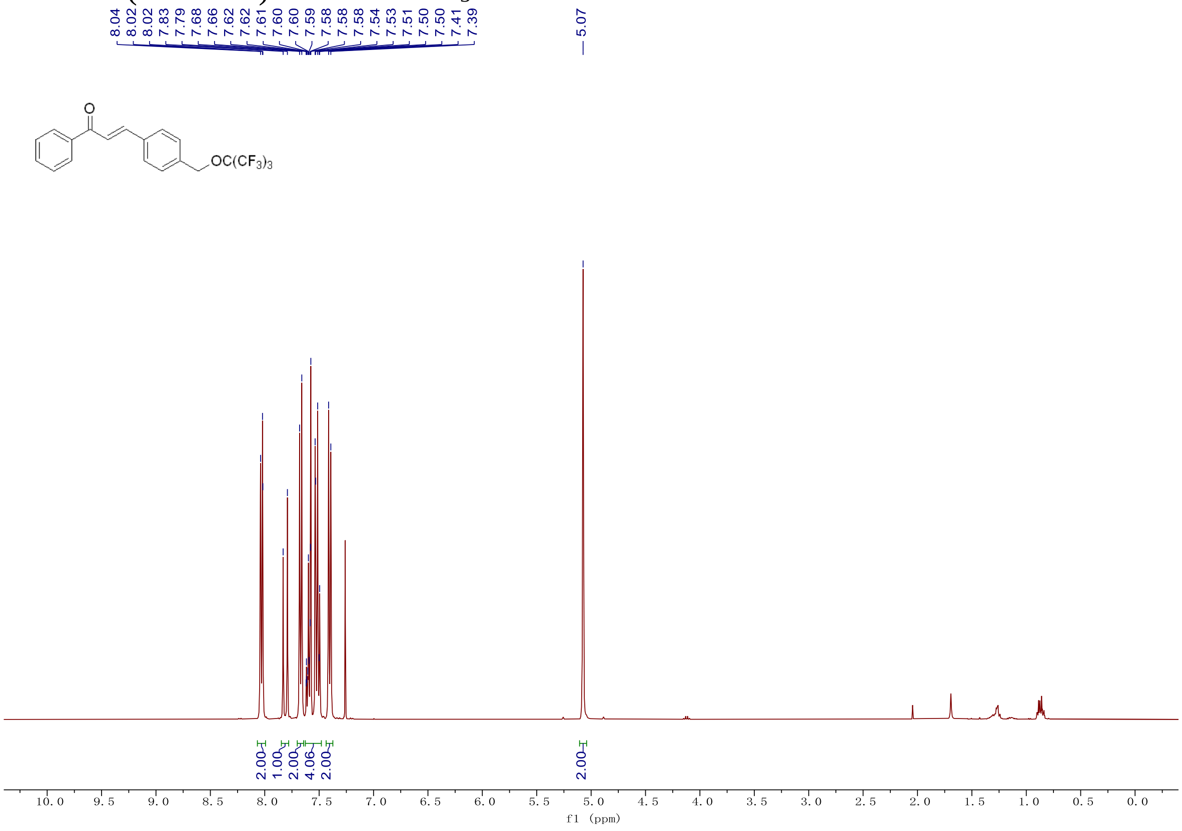

^{19}F NMR (376 MHz) of **1a** in $CDCl_3$

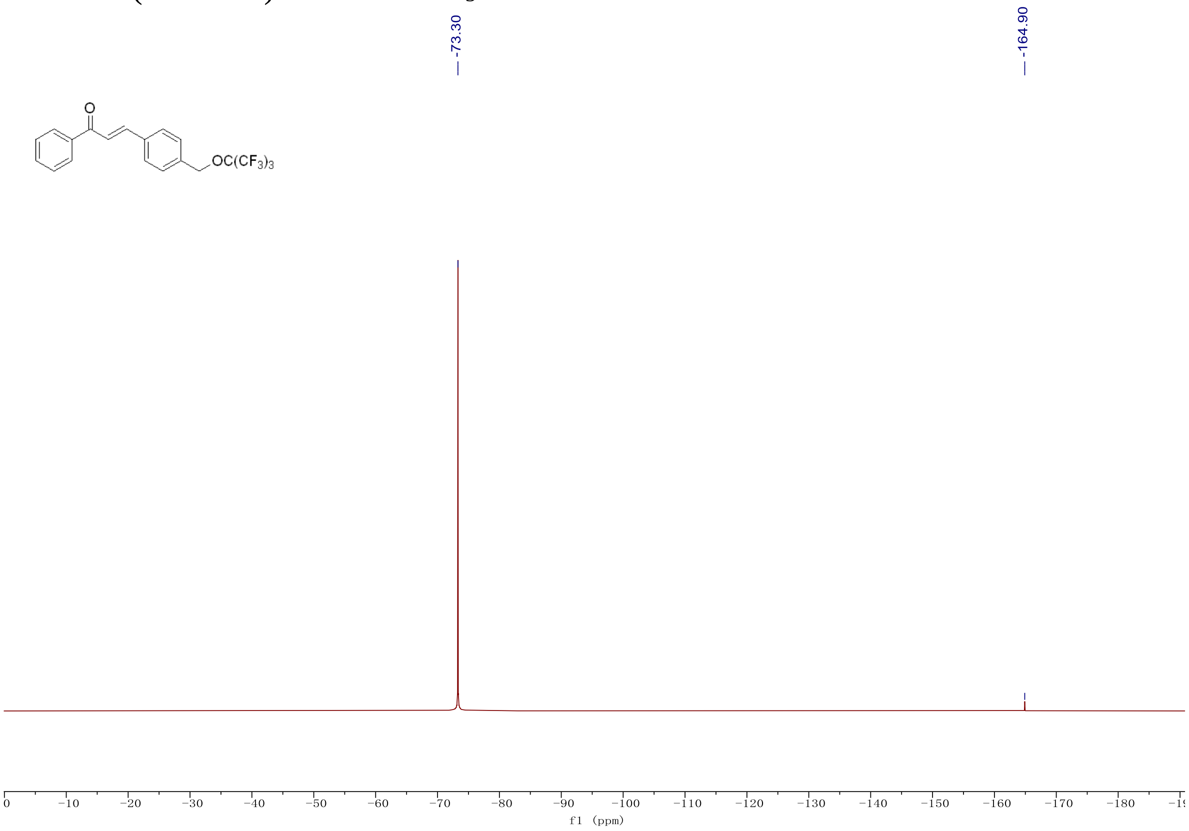

^{13}C NMR (101 MHz) of **1a** in $CDCl_3$

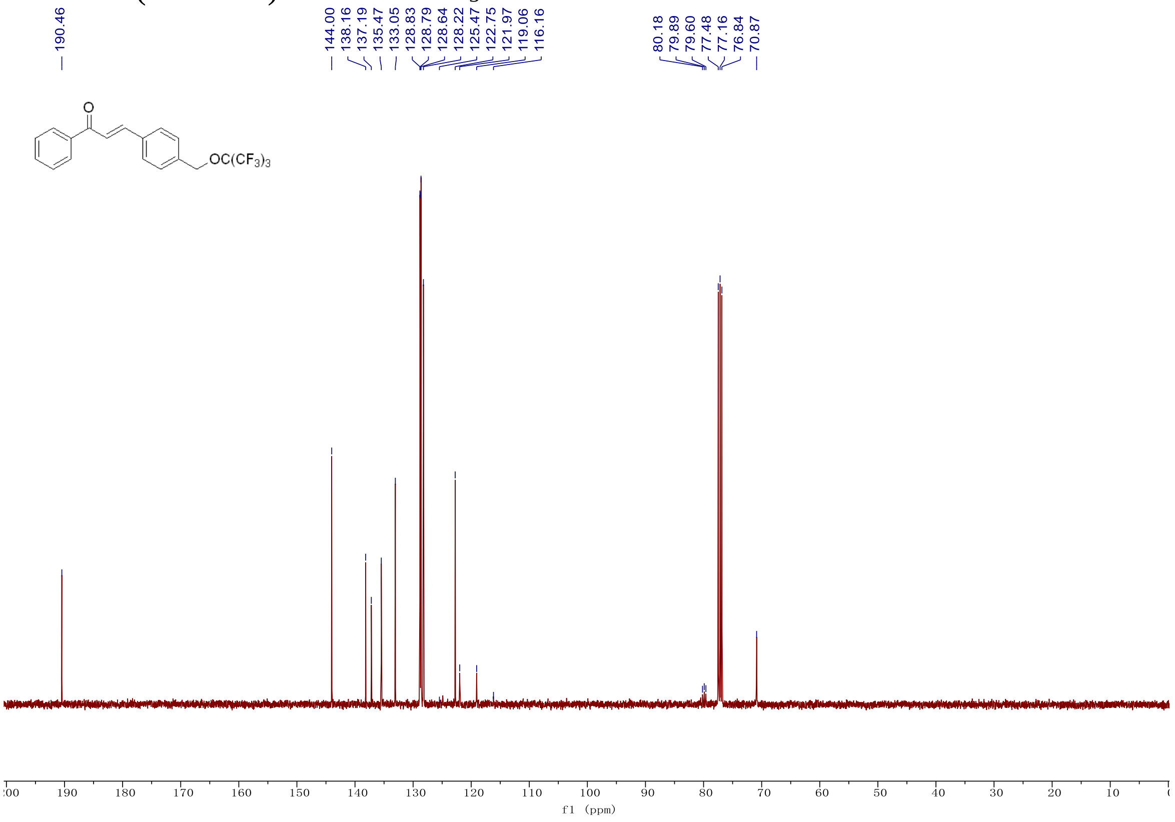

^{1}H NMR (400 MHz) of **2a** in $CDCl_3$

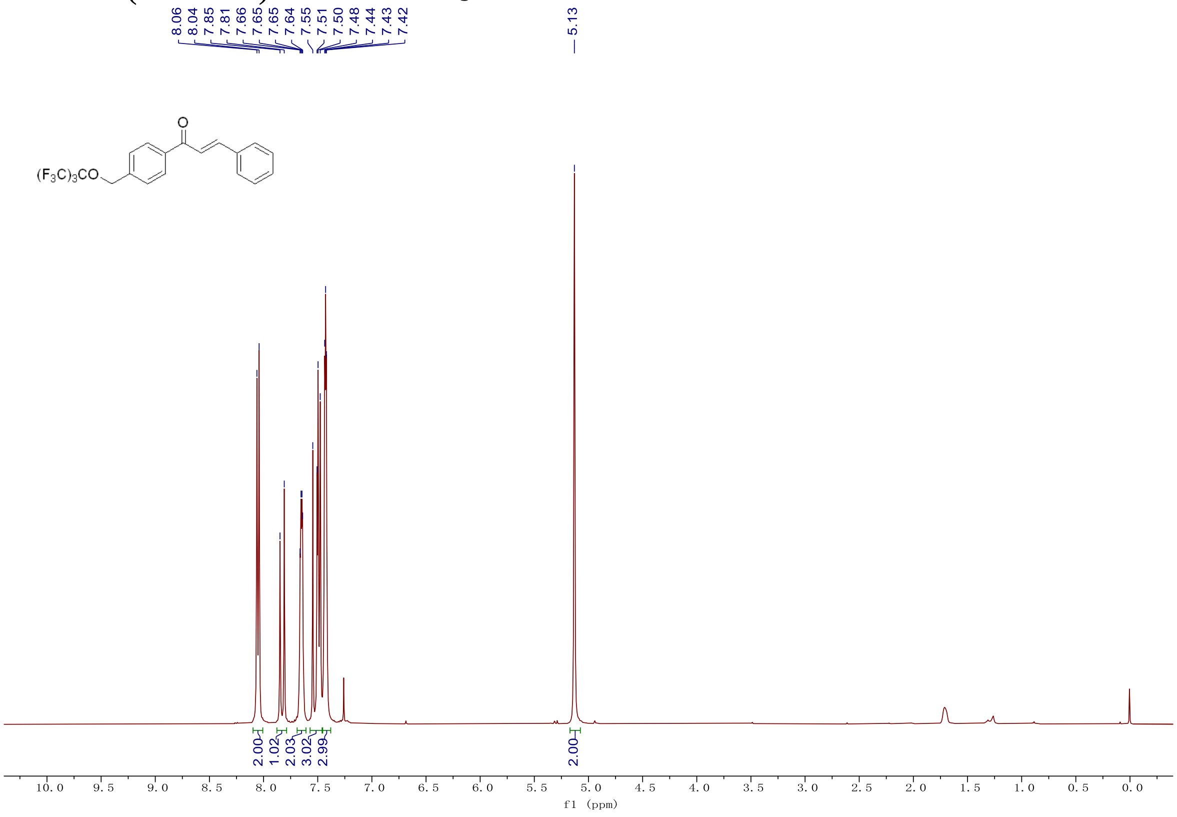

^{19}F NMR (376 MHz) of **2a** in $CDCl_3$

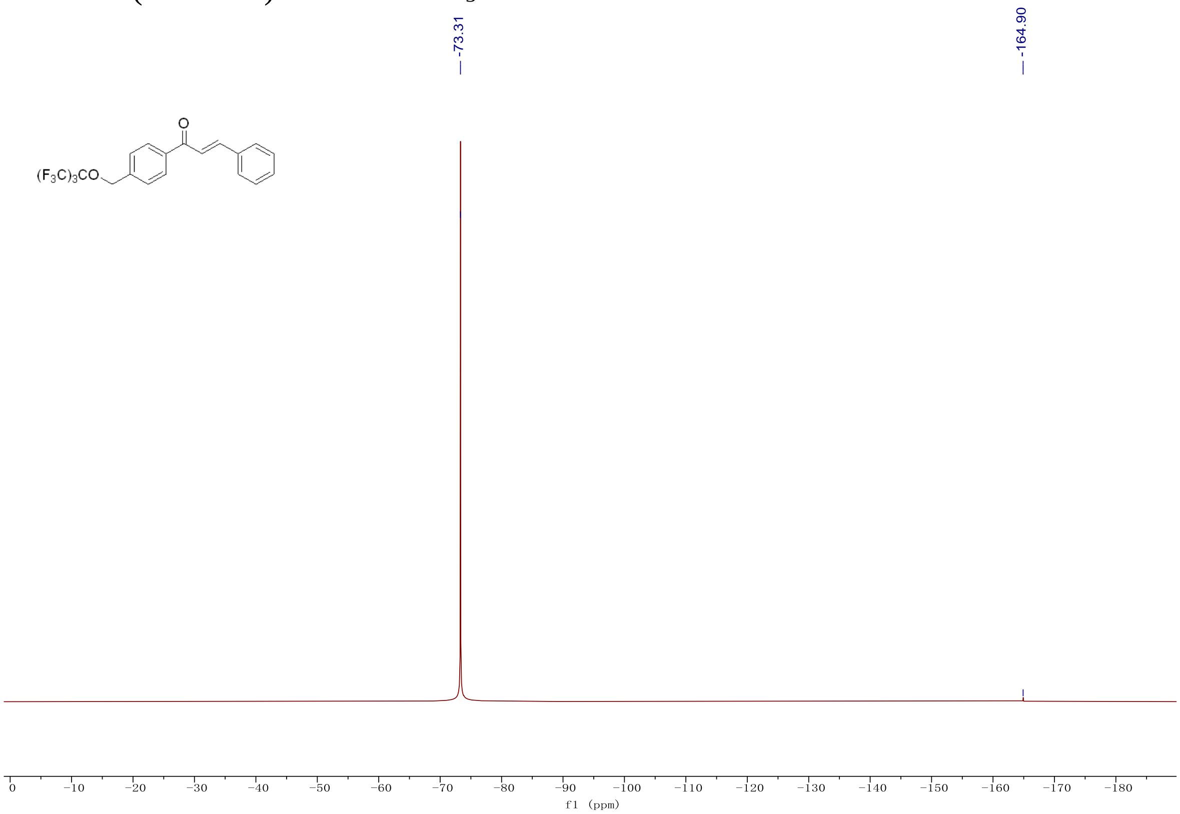

^{13}C NMR (101 MHz) of **2a** in $CDCl_3$

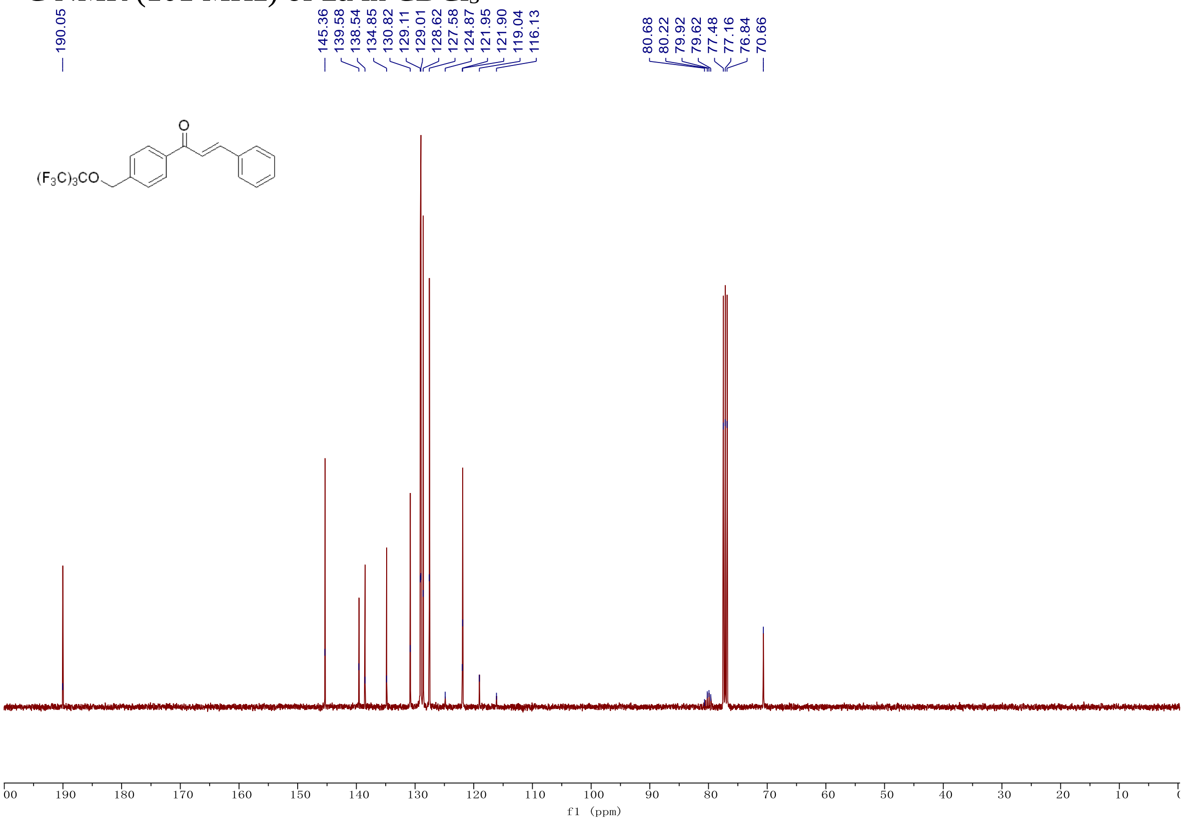

^{1}H NMR (500 MHz) of **3a** in $CDCl_3$

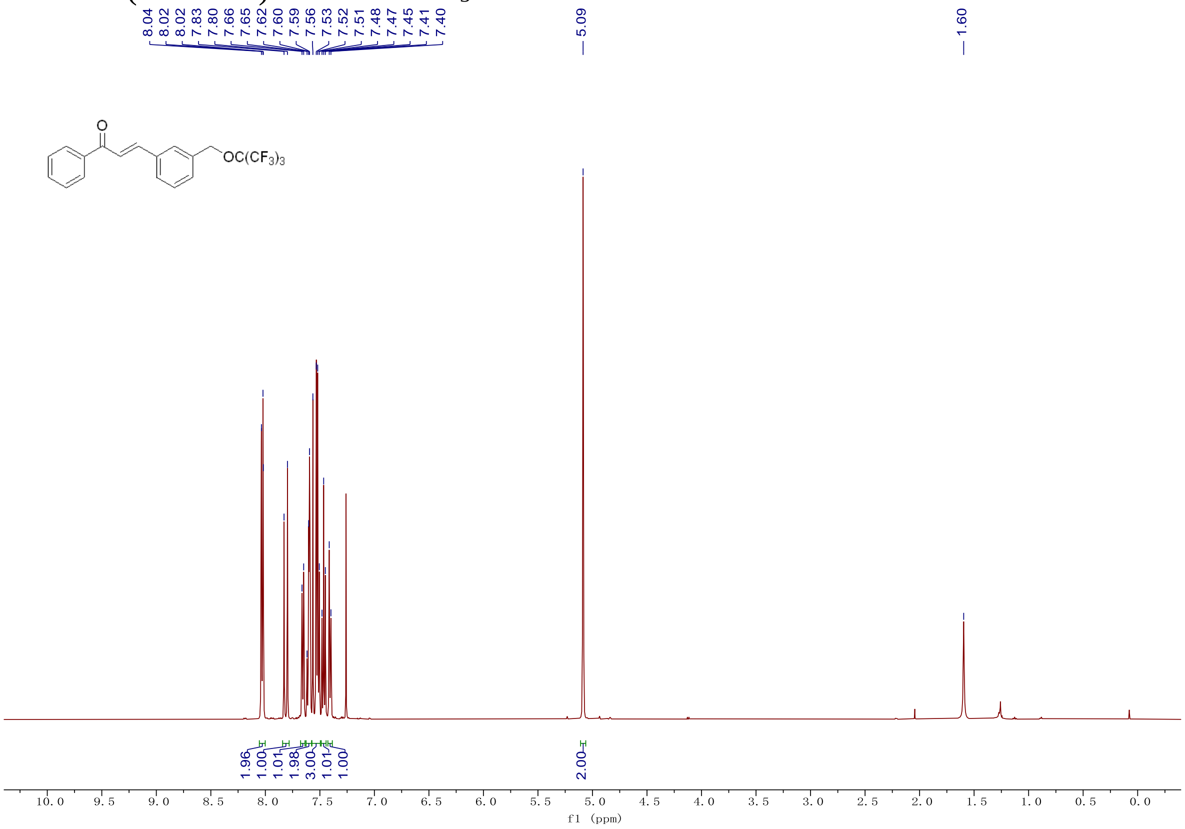

^{19}F NMR (471 MHz) of **3a** in $CDCl_3$

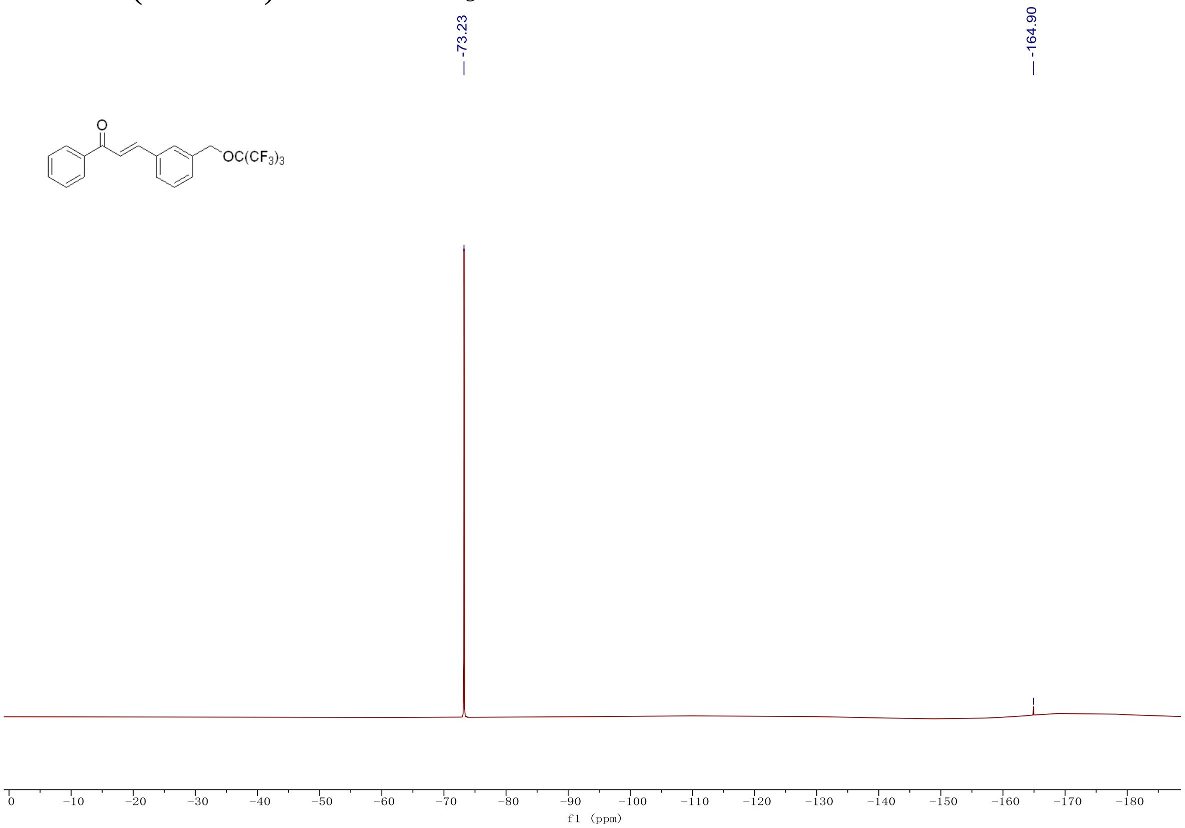

^{13}C NMR (126 MHz) of **3a** in $CDCl_3$

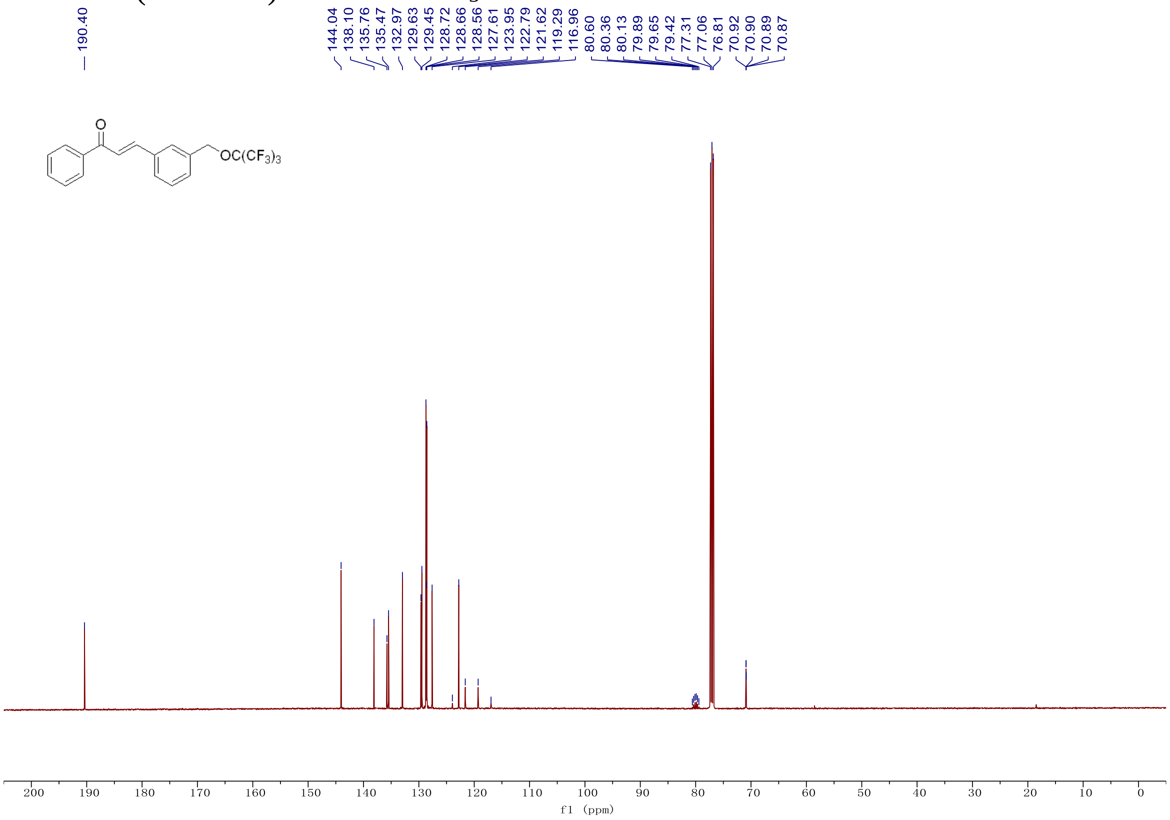

^{1}H NMR (500 MHz) of **4a** in $CDCl_3$

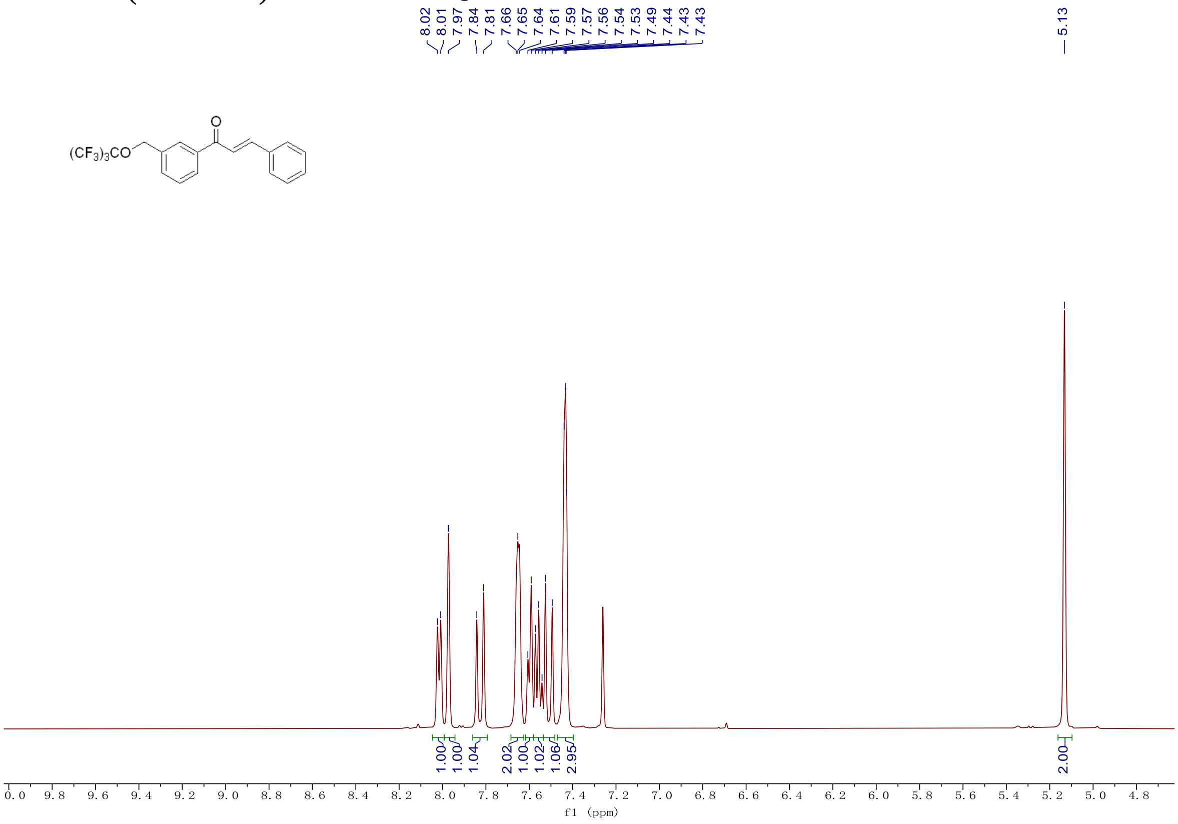

^{19}F NMR (471 MHz) of **4a** in $CDCl_3$

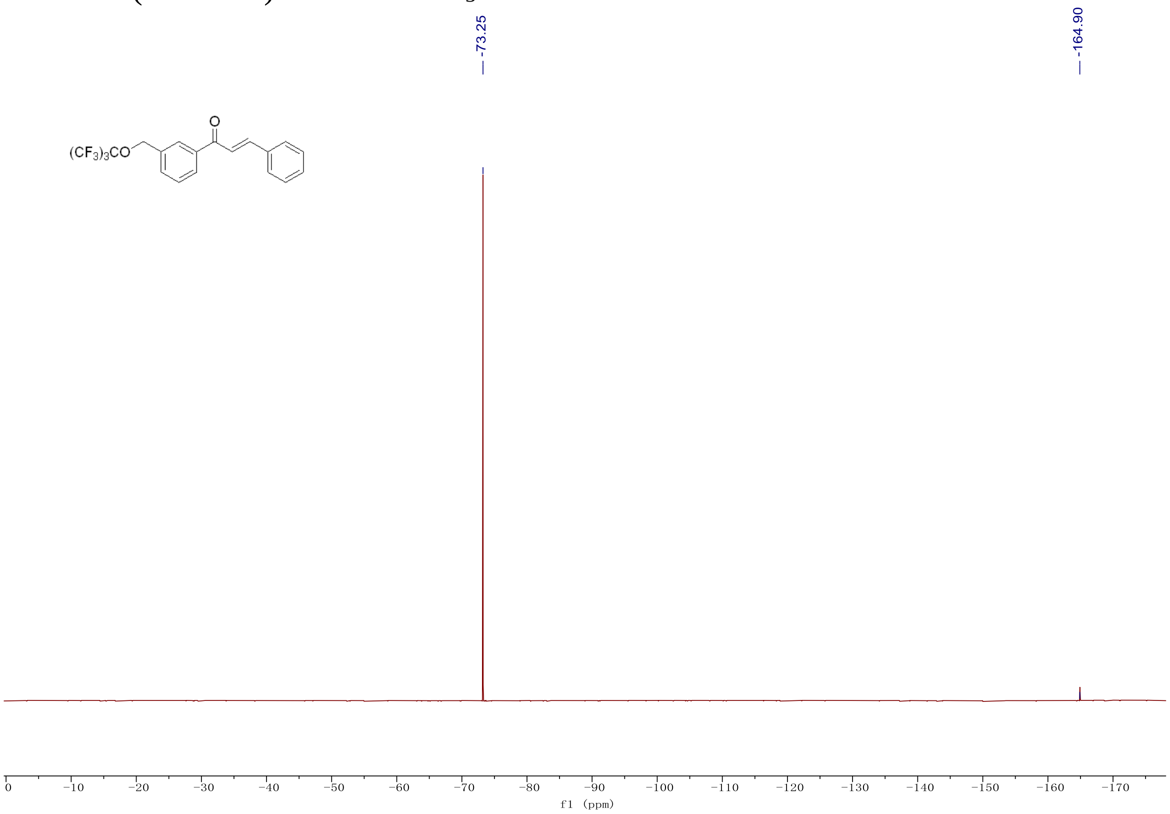

^{13}C NMR (126 MHz) of **4a** in $CDCl_3$

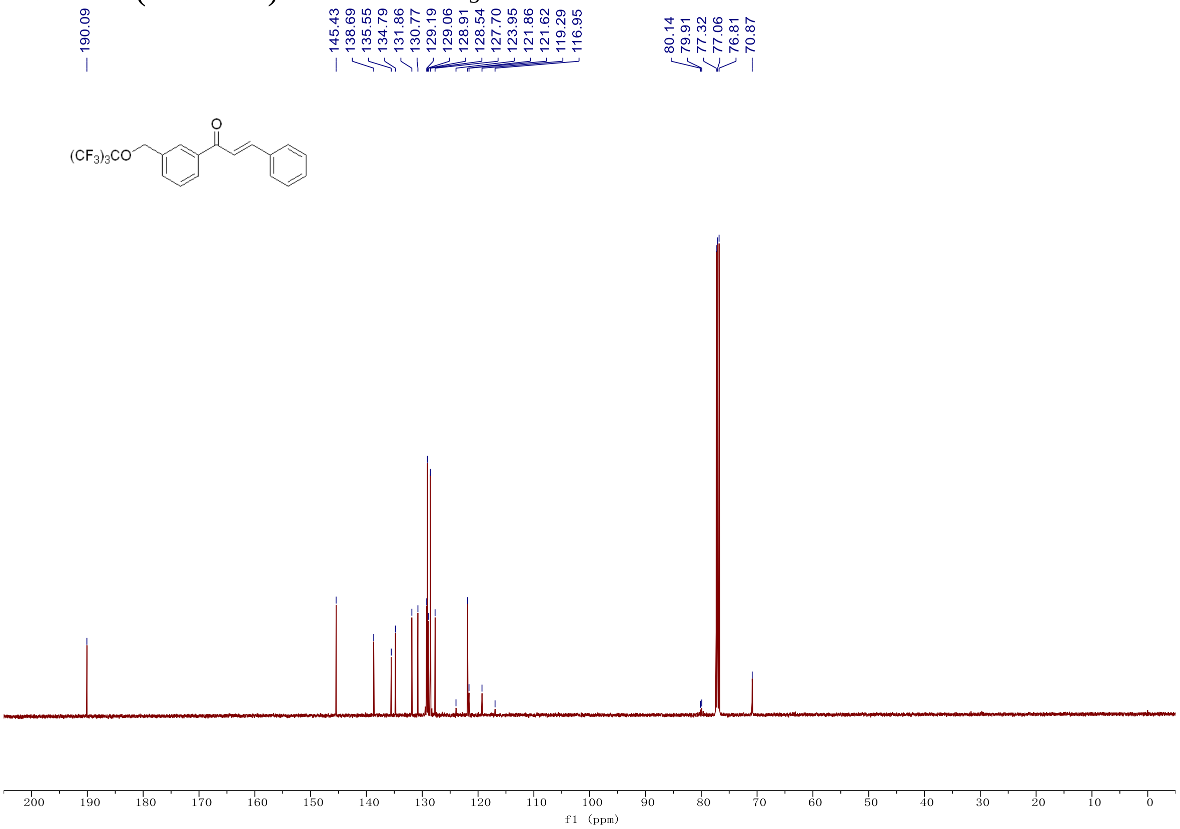

^1H NMR (400 MHz) of **5a** in $CDCl_3$

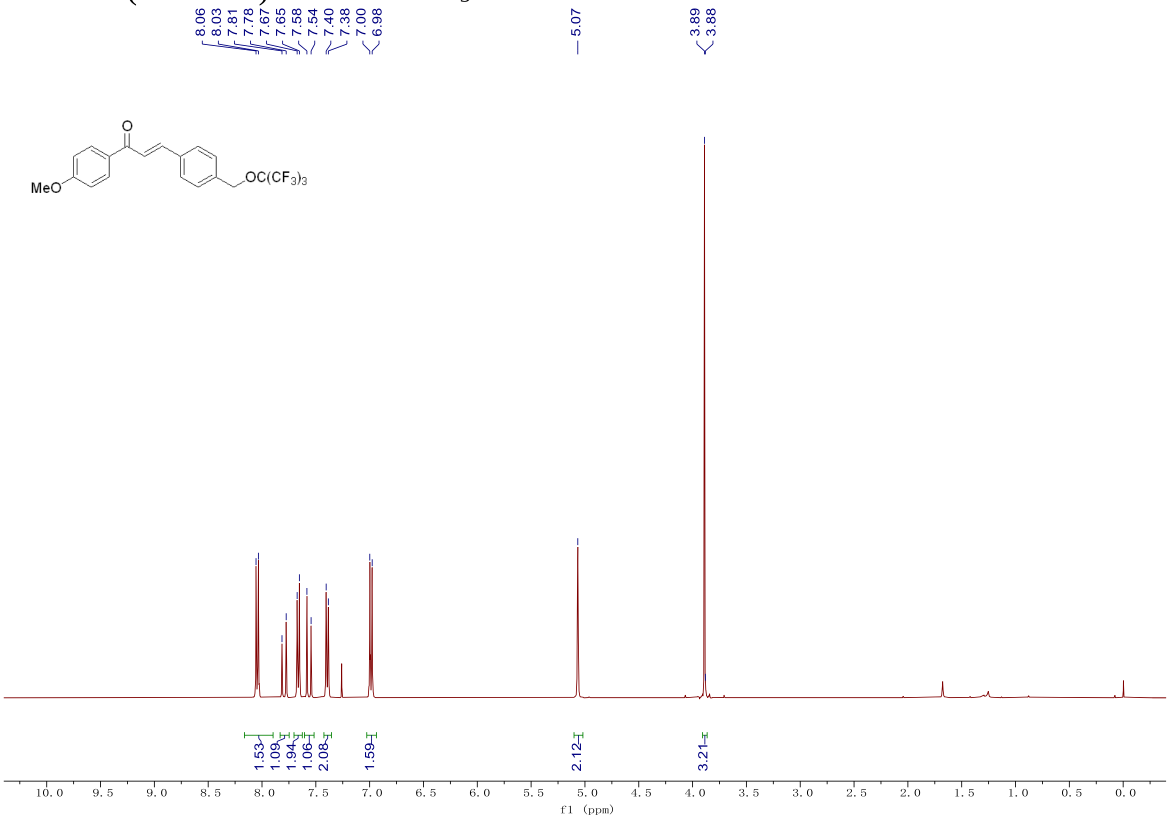

^{19}F NMR (376 MHz) of **5a** in $CDCl_3$

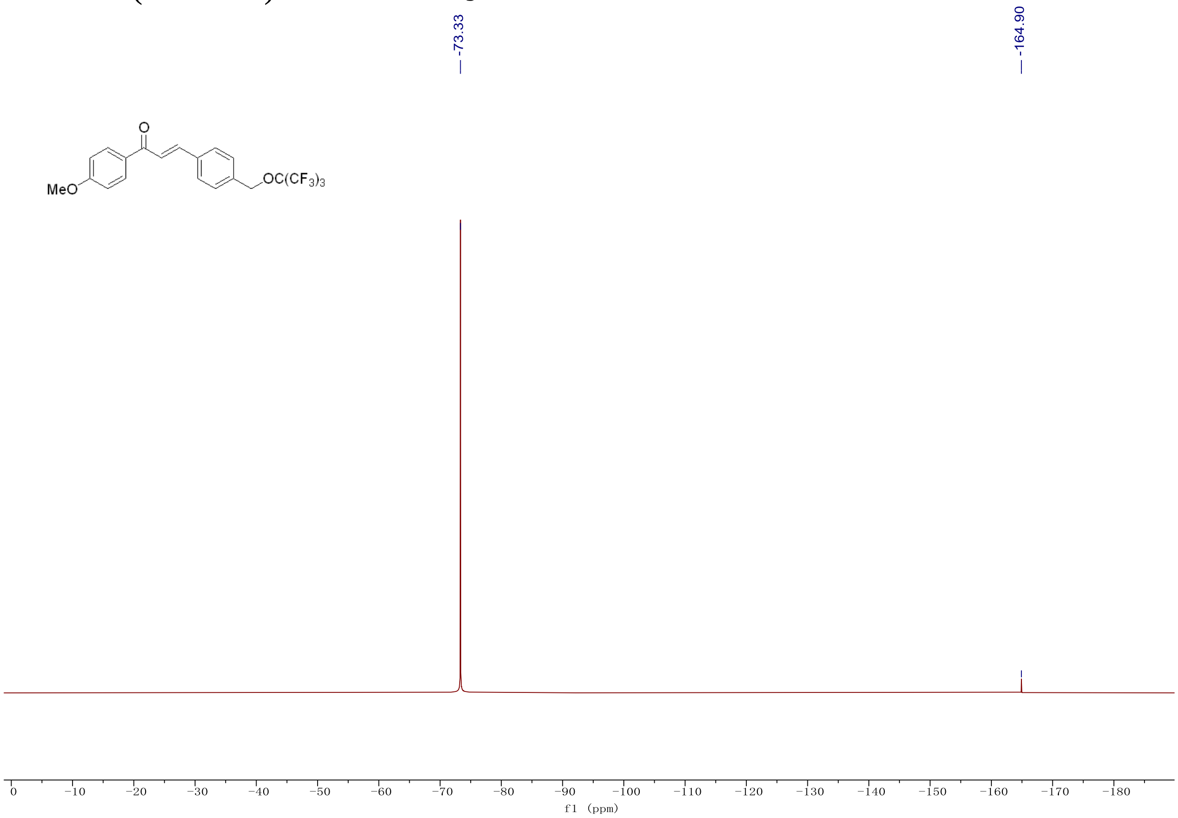

^{13}C NMR (151 MHz) of **5a** in $CDCl_3$

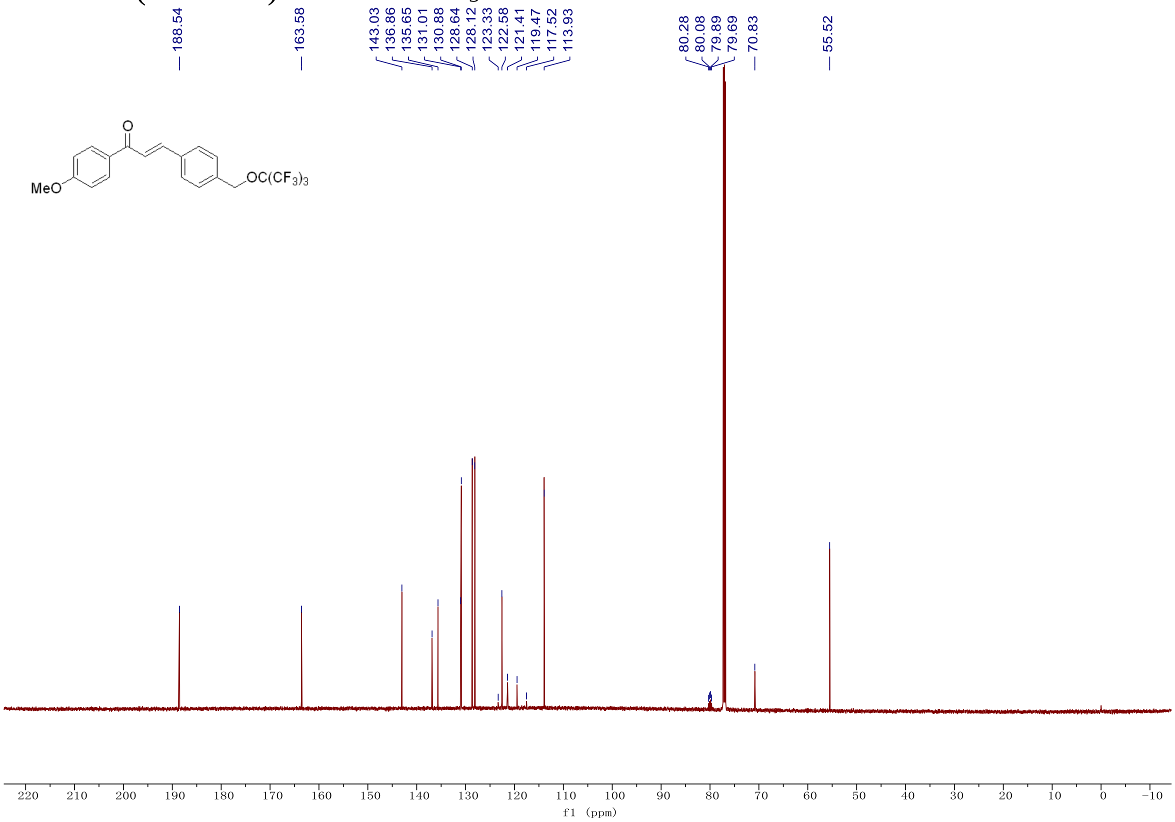

^{1}H NMR (400 MHz) of **6a** in $CDCl_3$

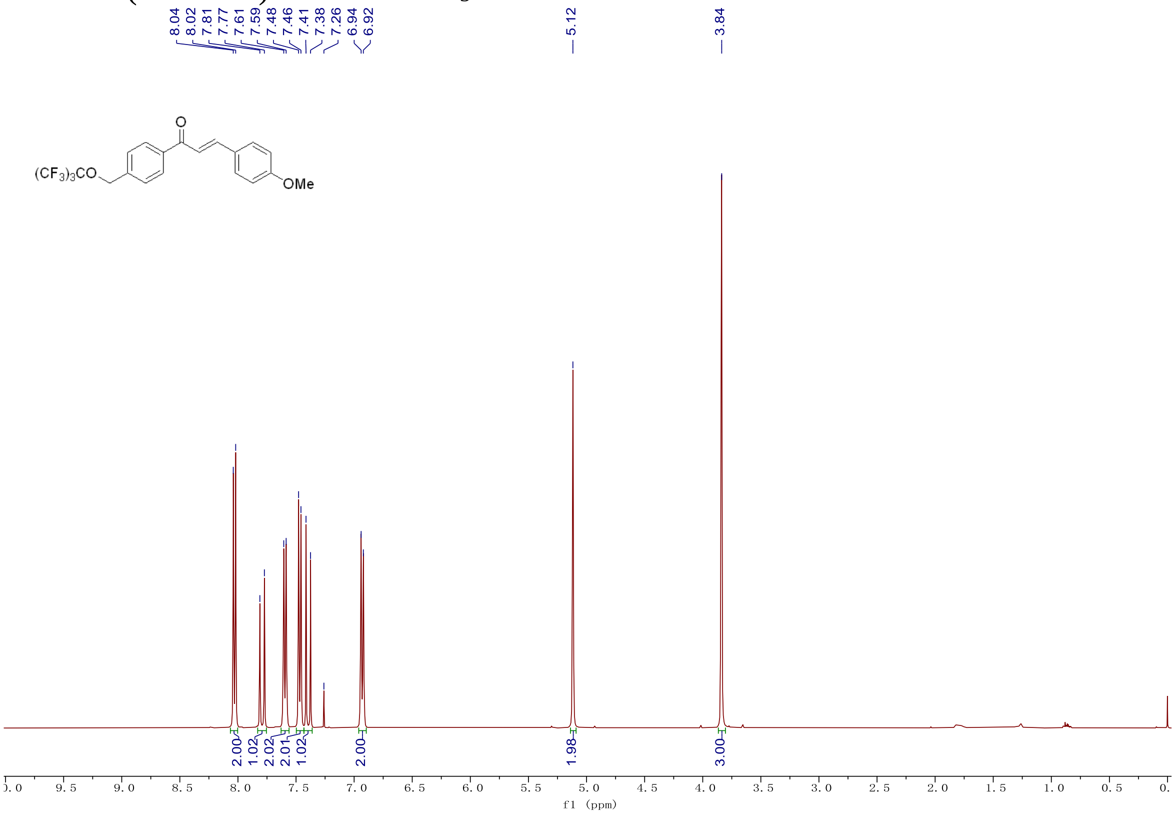

^{19}F NMR (376 MHz) of **6a** in $CDCl_3$

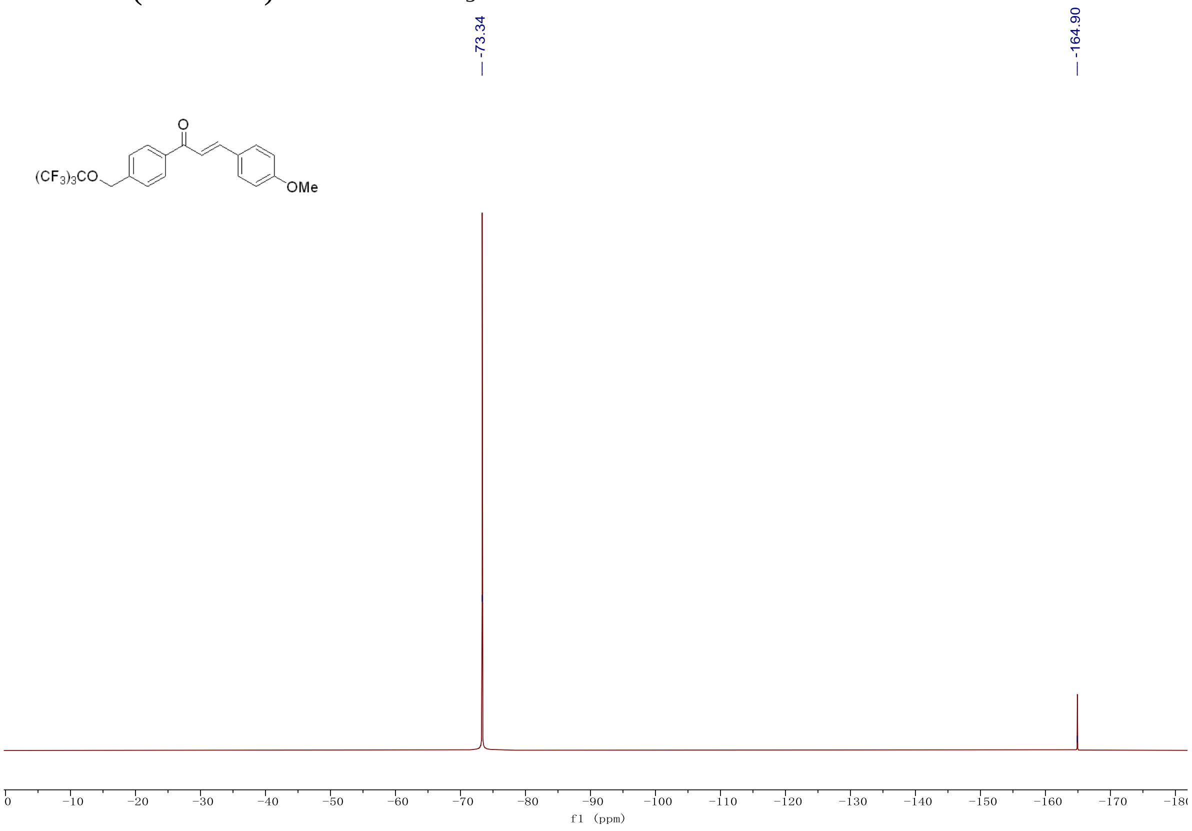

^{13}C NMR (1510 MHz) of **6a** in $CDCl_3$

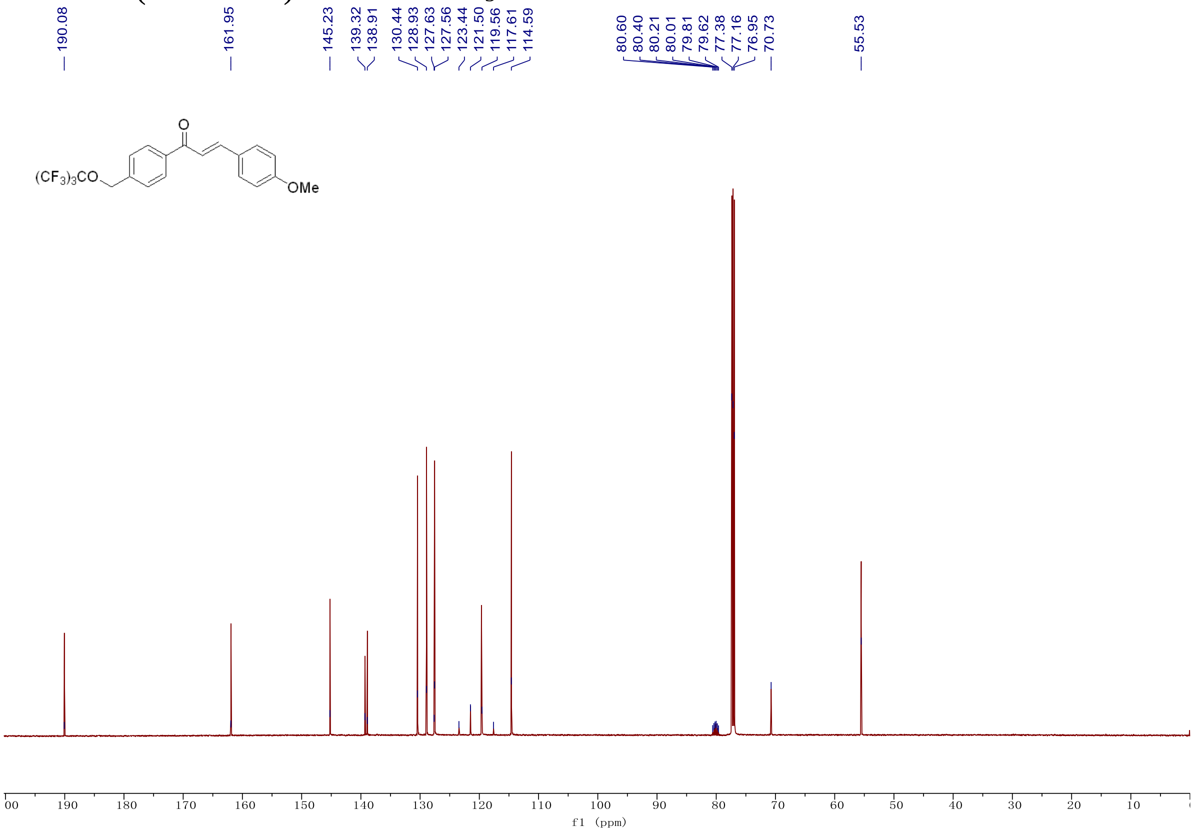

^{1}H NMR (500 MHz) of **7a** in $CDCl_3$

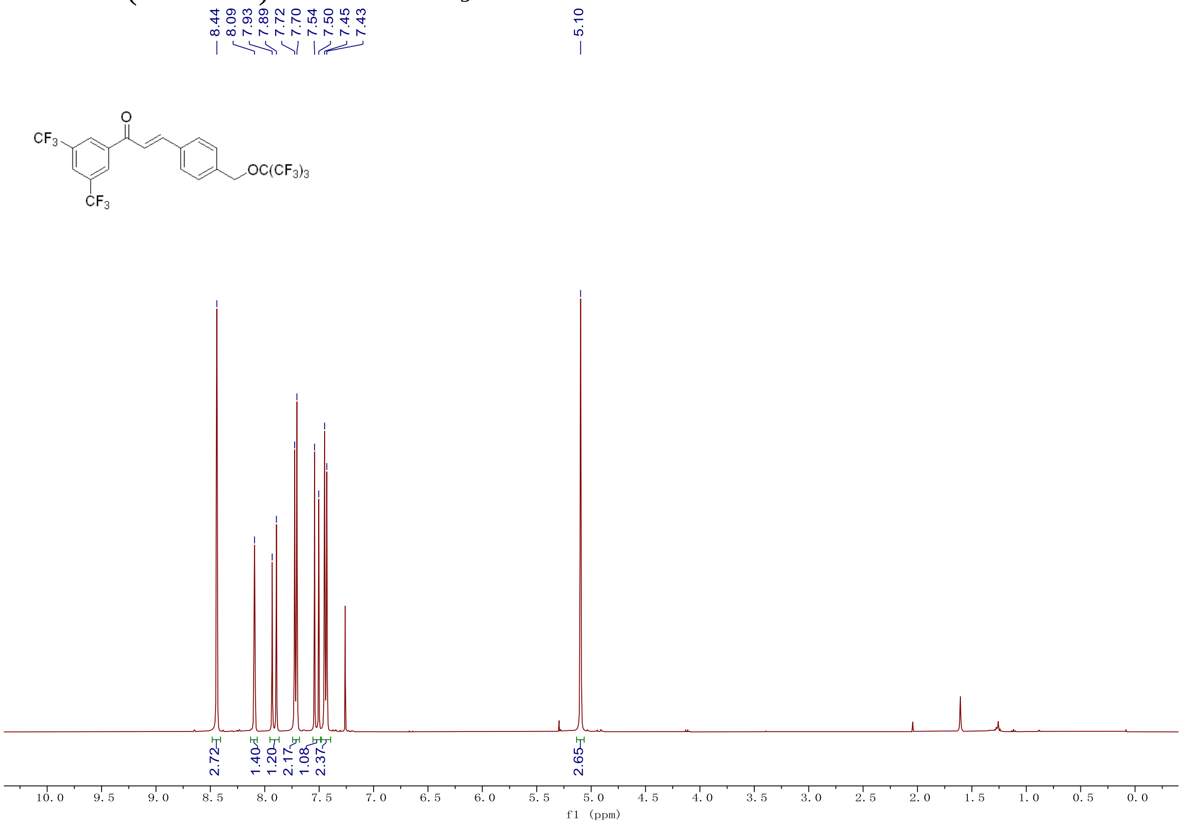

^{19}F NMR (376 MHz) of **7a** in $CDCl_3$

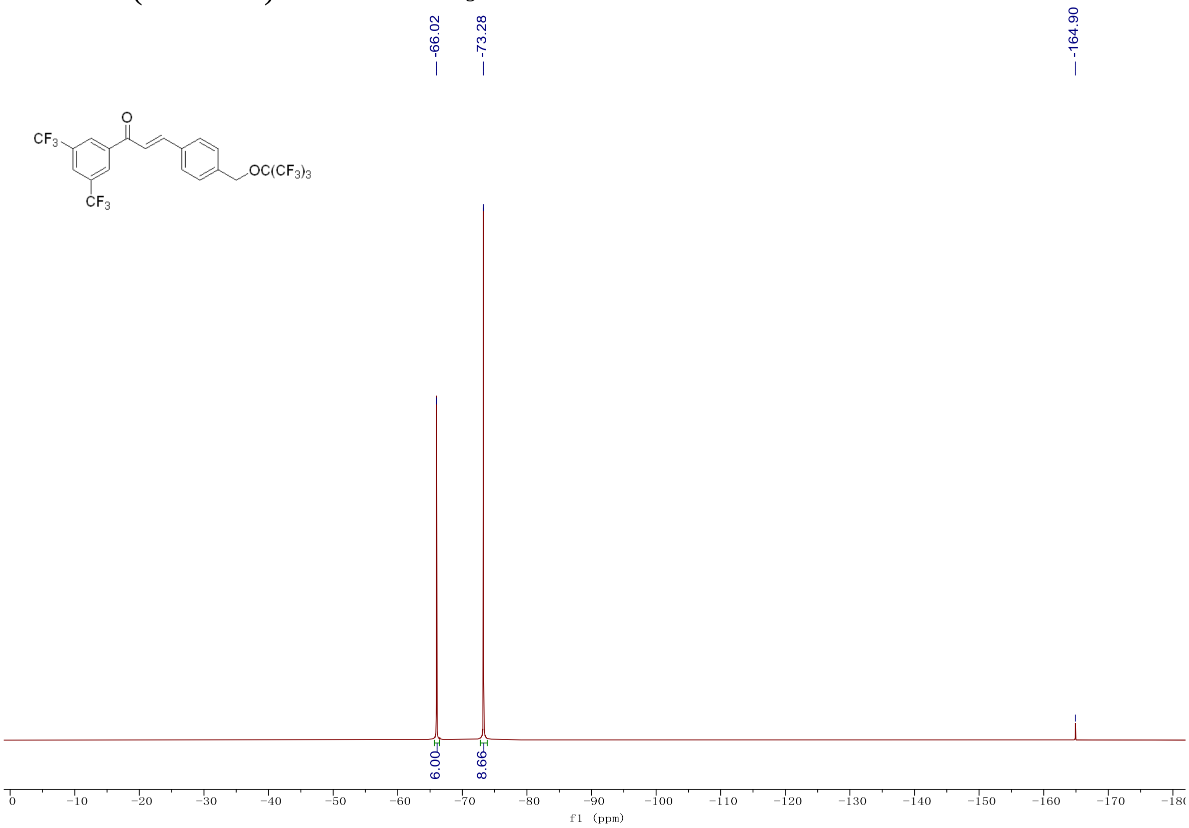

^{13}C NMR (126 MHz) of **7a** in $CDCl_3$

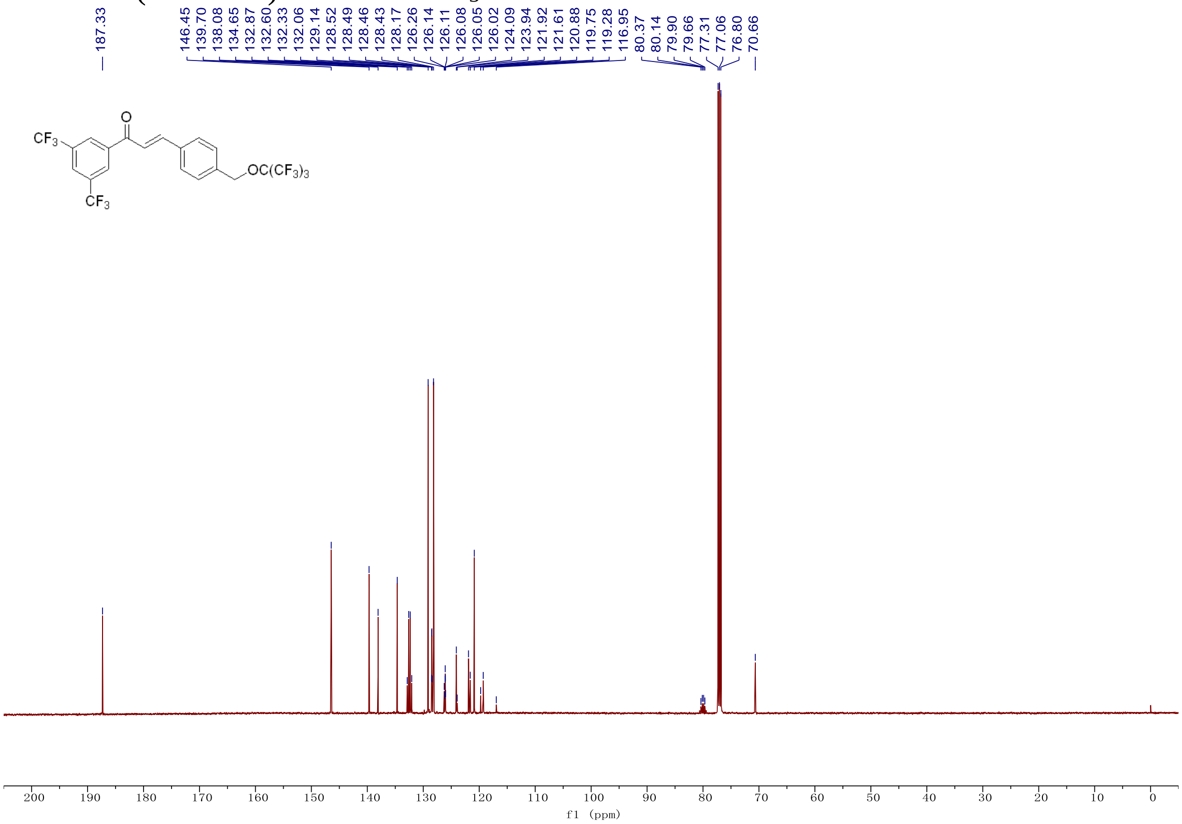

^{1}H NMR (500 MHz) of **8a** in $CDCl_3$

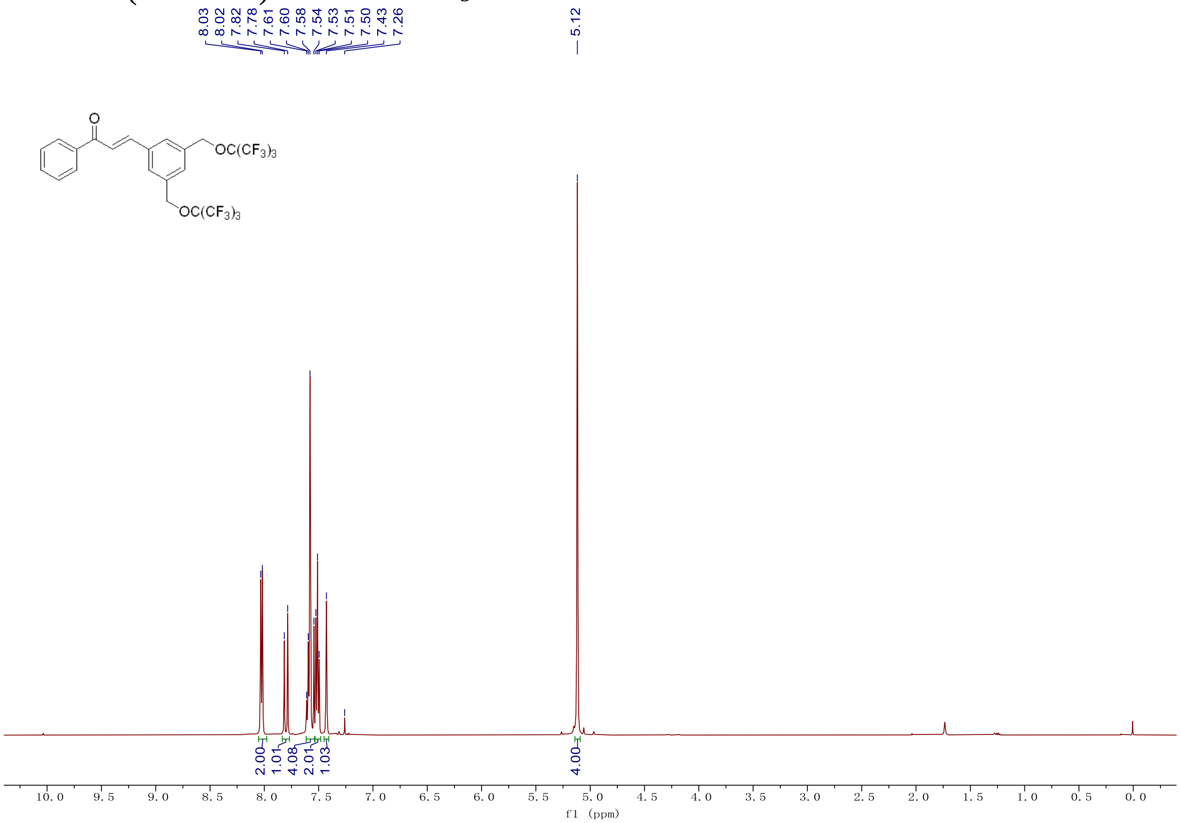

^{19}F NMR (471 MHz) of **8a** in $CDCl_3$

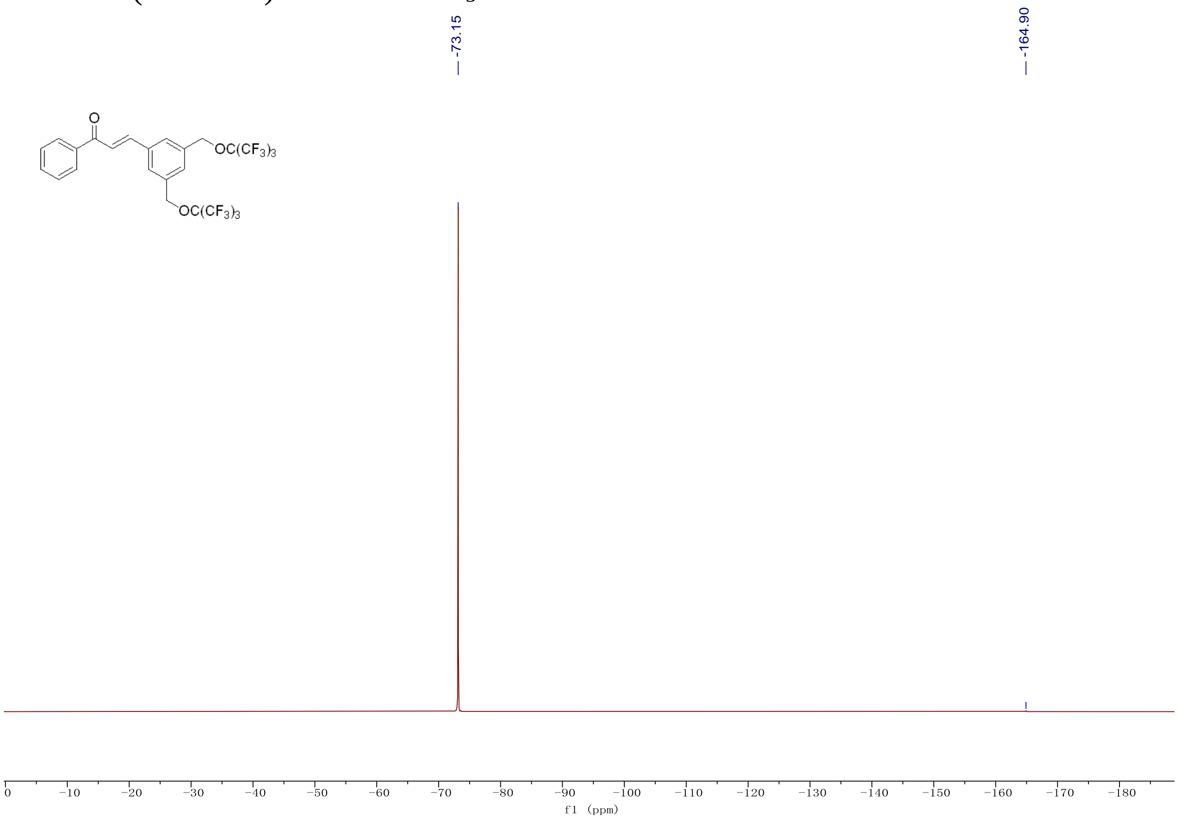

^{13}C NMR (126 MHz) of **8a** in $CDCl_3$

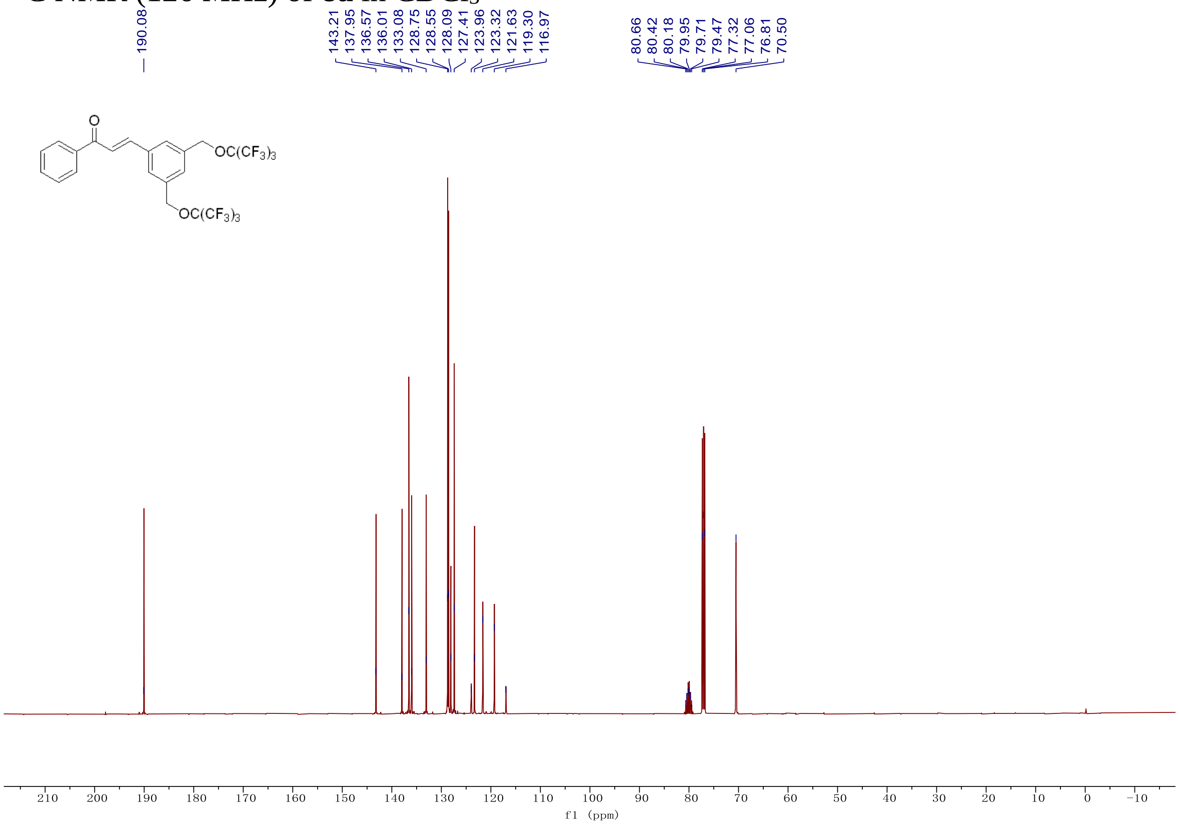

^{1}H NMR (500 MHz) of **9a** in $CDCl_3$

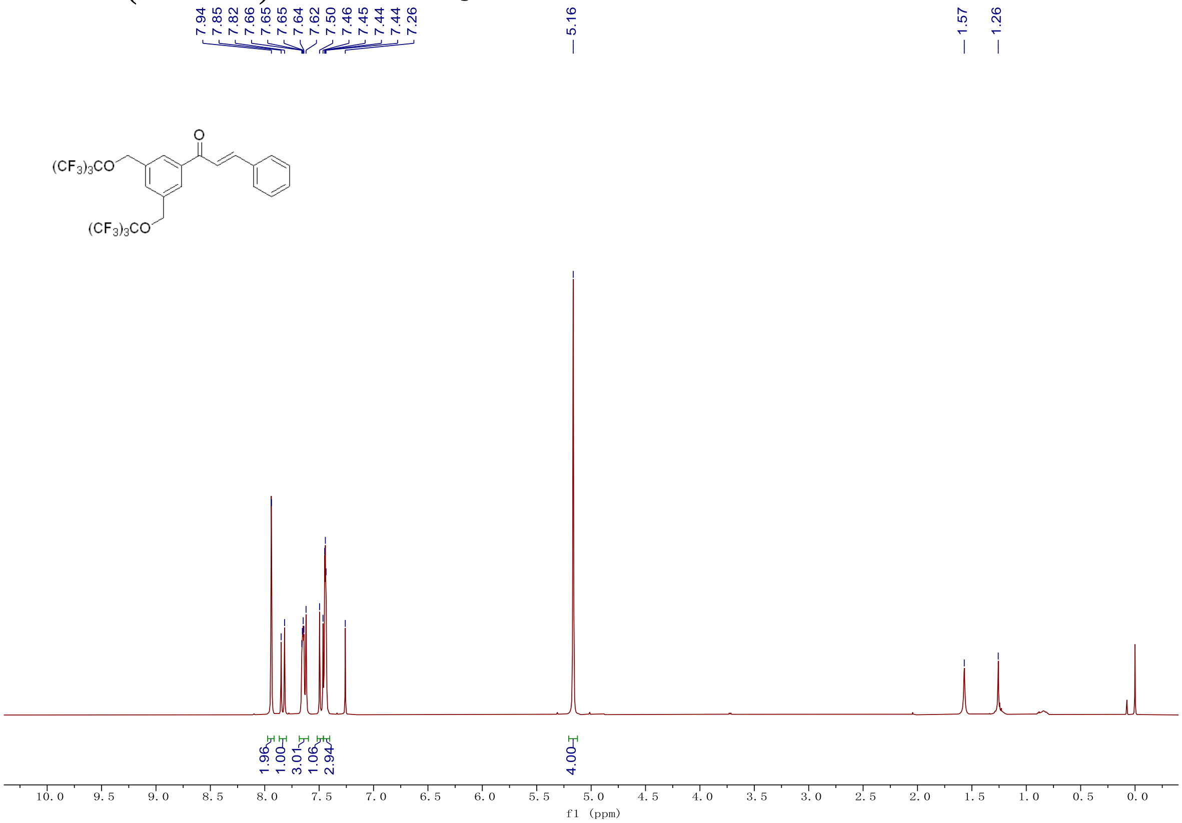

^{19}F NMR (471 MHz) of **9a** in $CDCl_3$

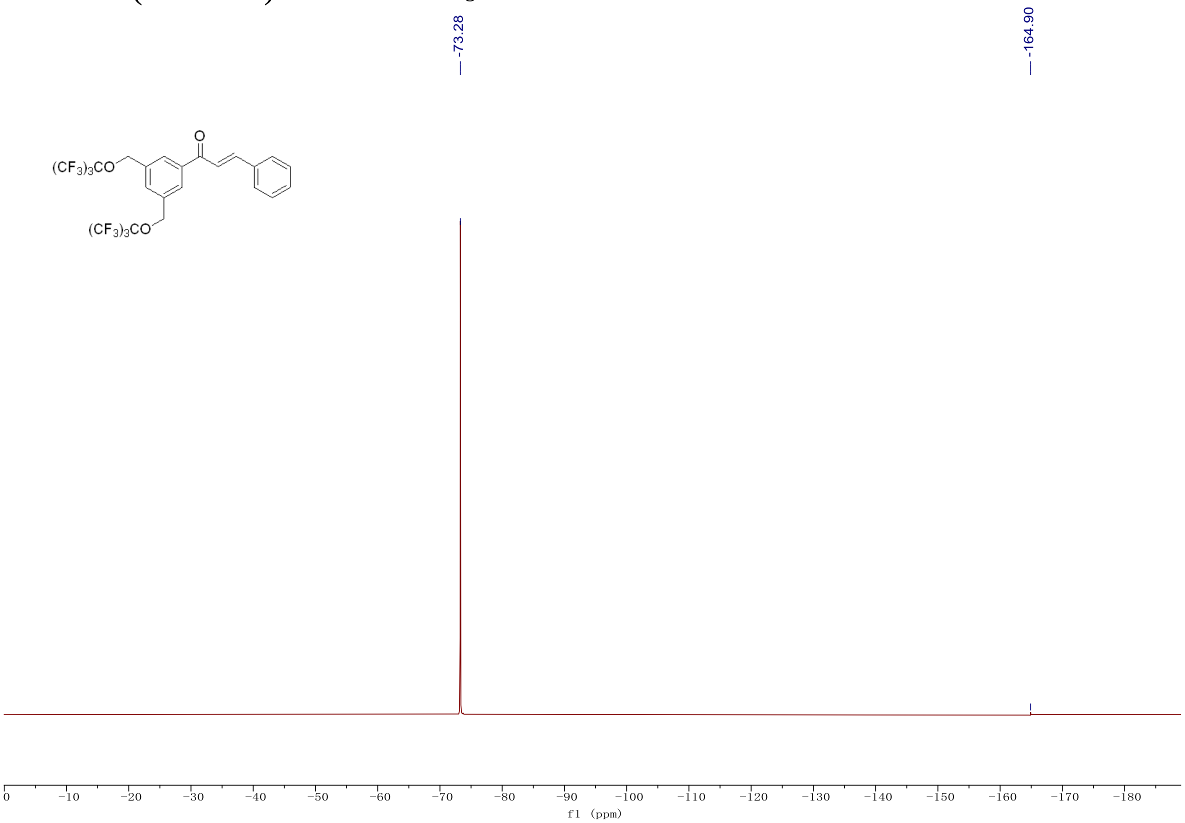

^{13}C NMR (126 MHz) of **9a** in $CDCl_3$

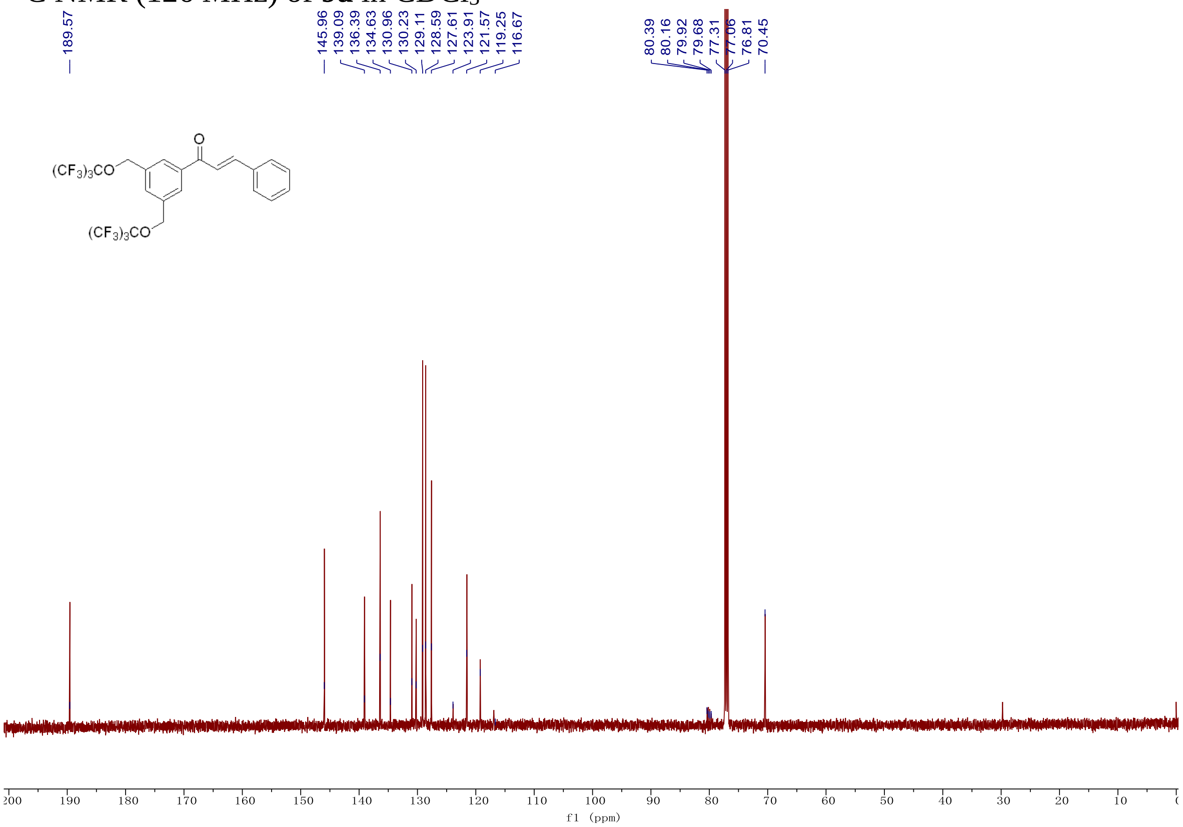

^{1}H NMR (500 MHz) of **10a** in $CDCl_3$

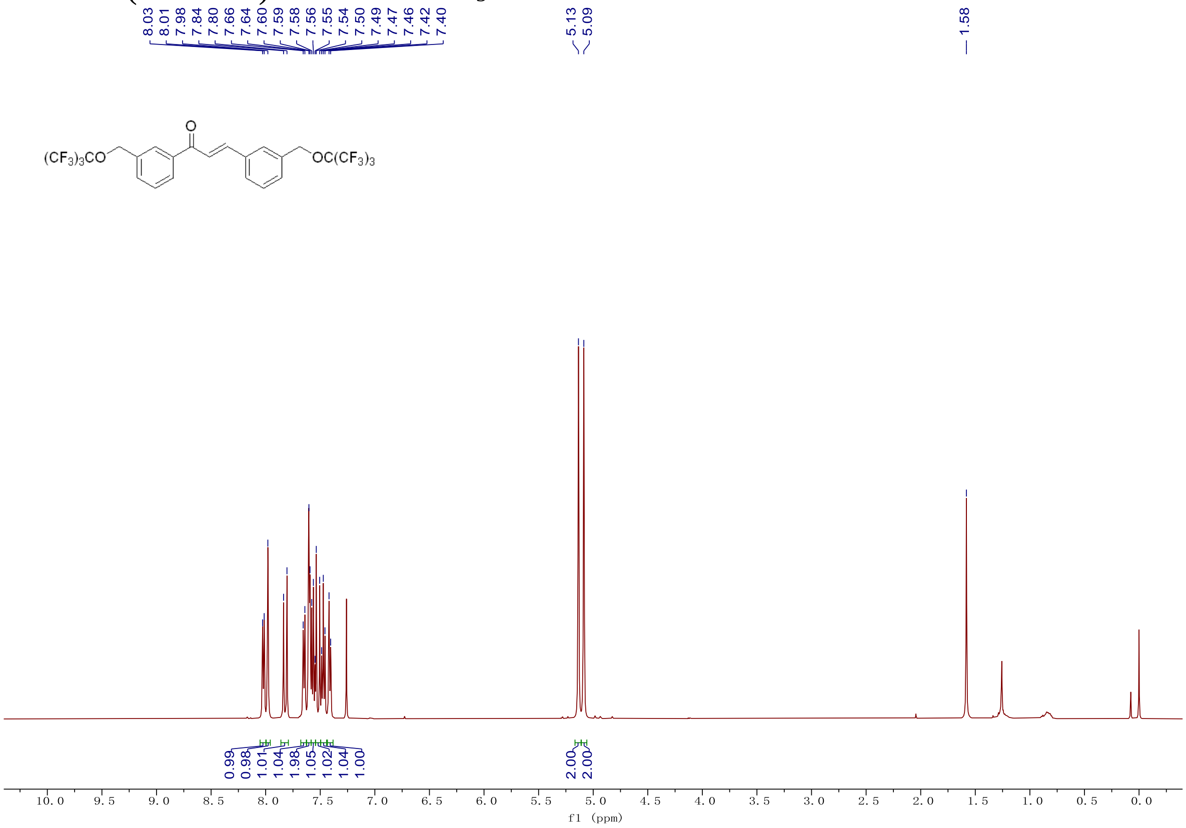

^{19}F NMR (471 MHz) of **10a** in $CDCl_3$

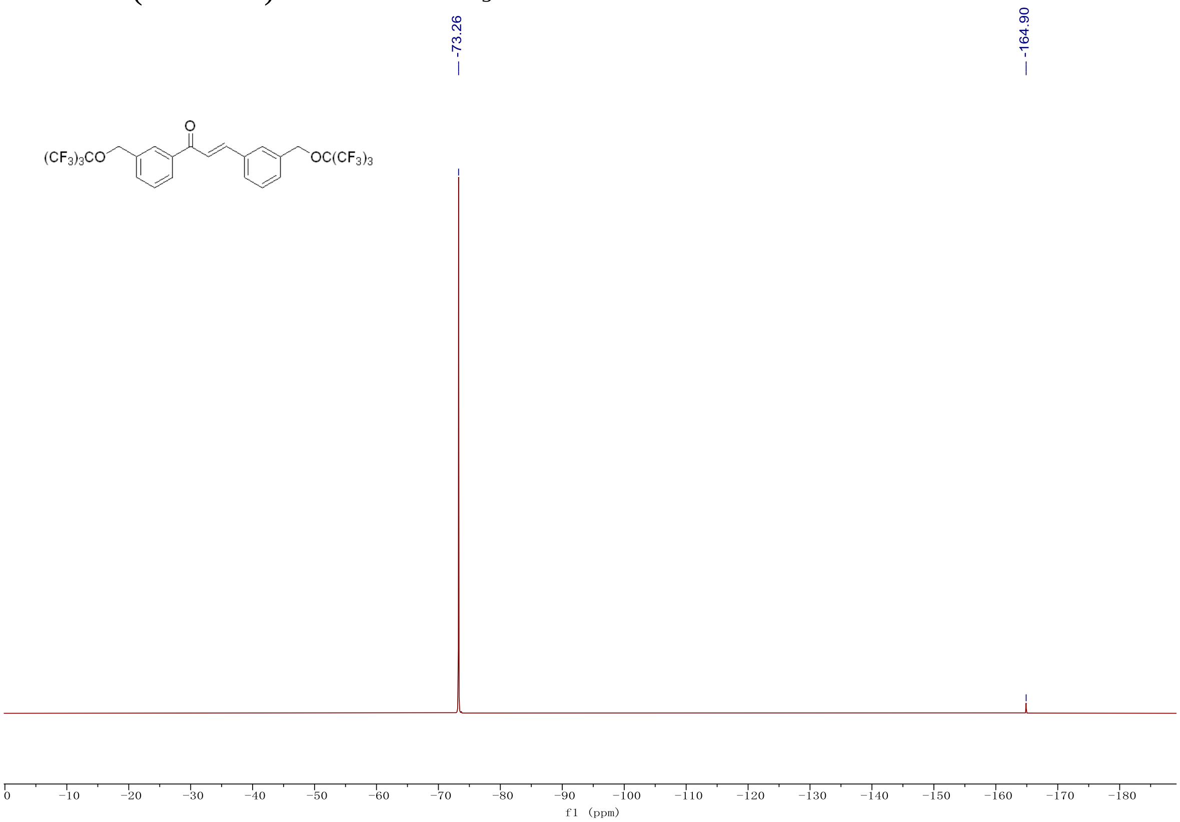

^{13}C NMR (126 MHz) of **10a** in $CDCl_3$

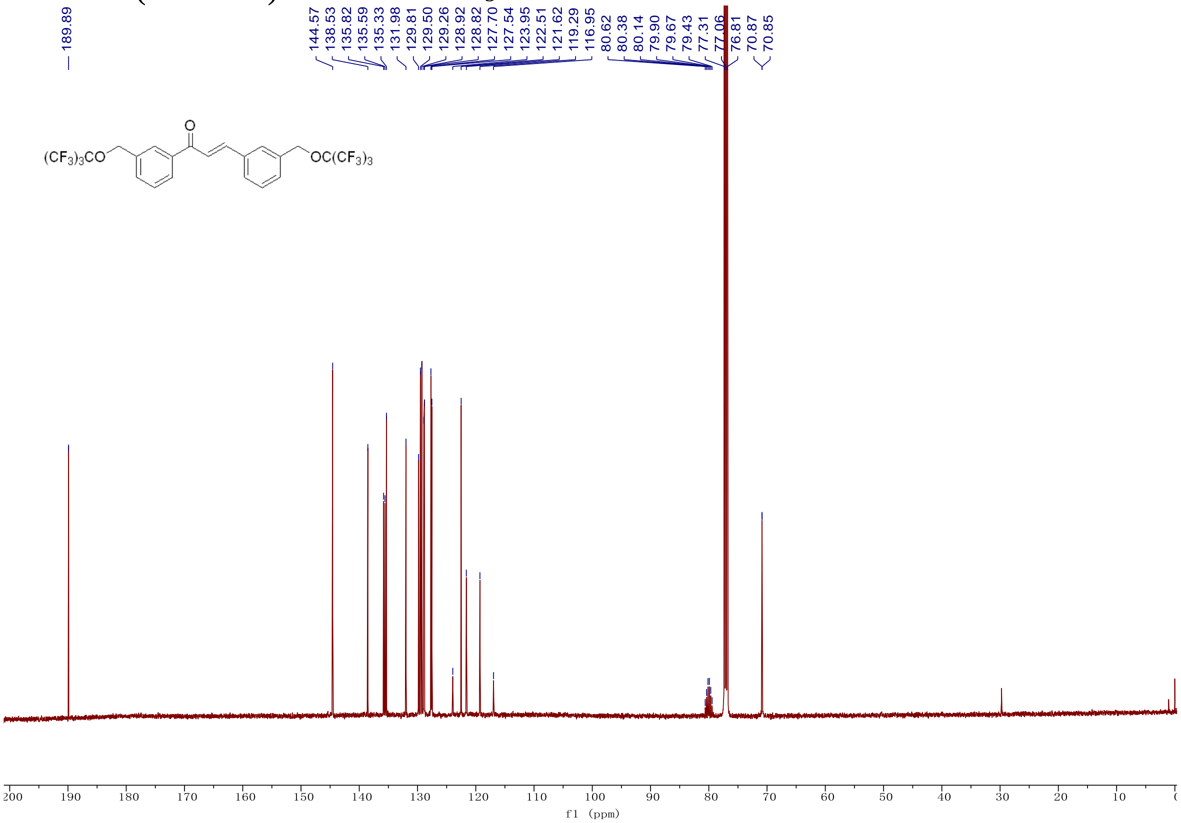

^{1}H NMR (500 MHz) of **1b** in $CDCl_3$

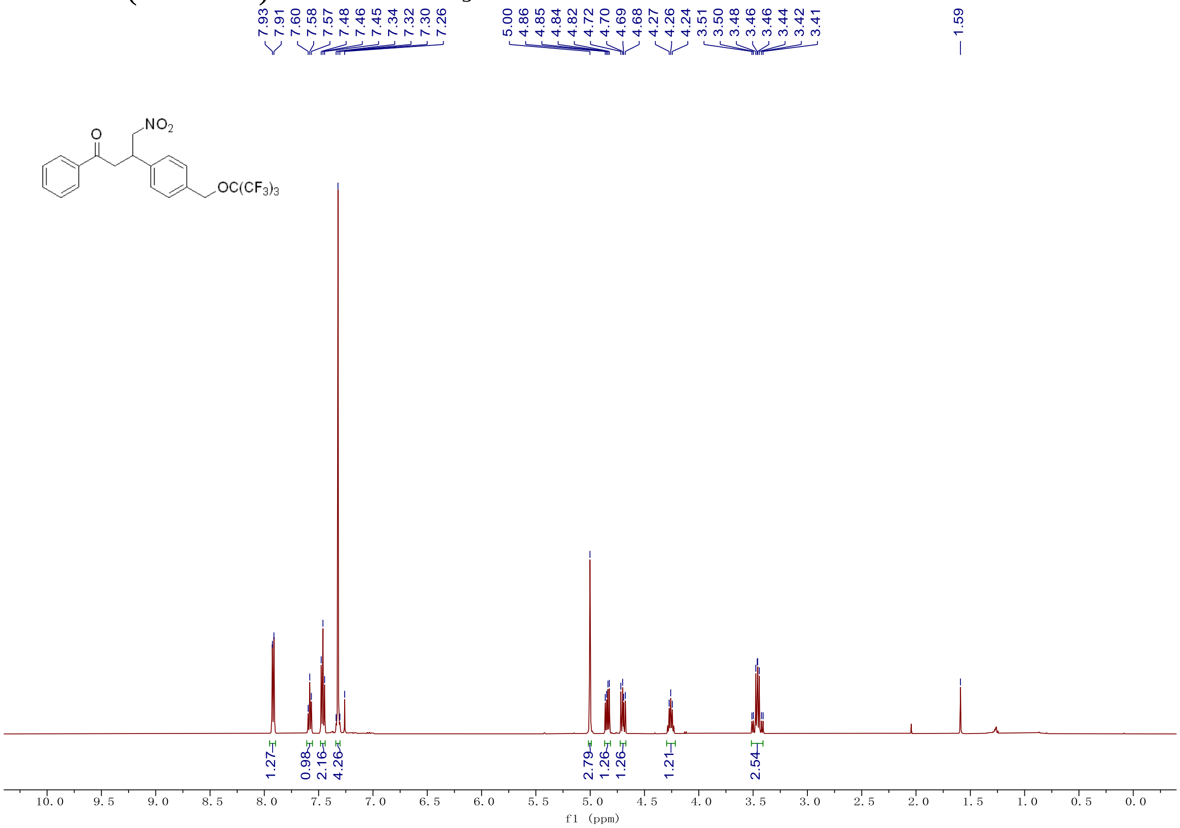

^{19}F NMR (376 MHz) of **1b** in $CDCl_3$

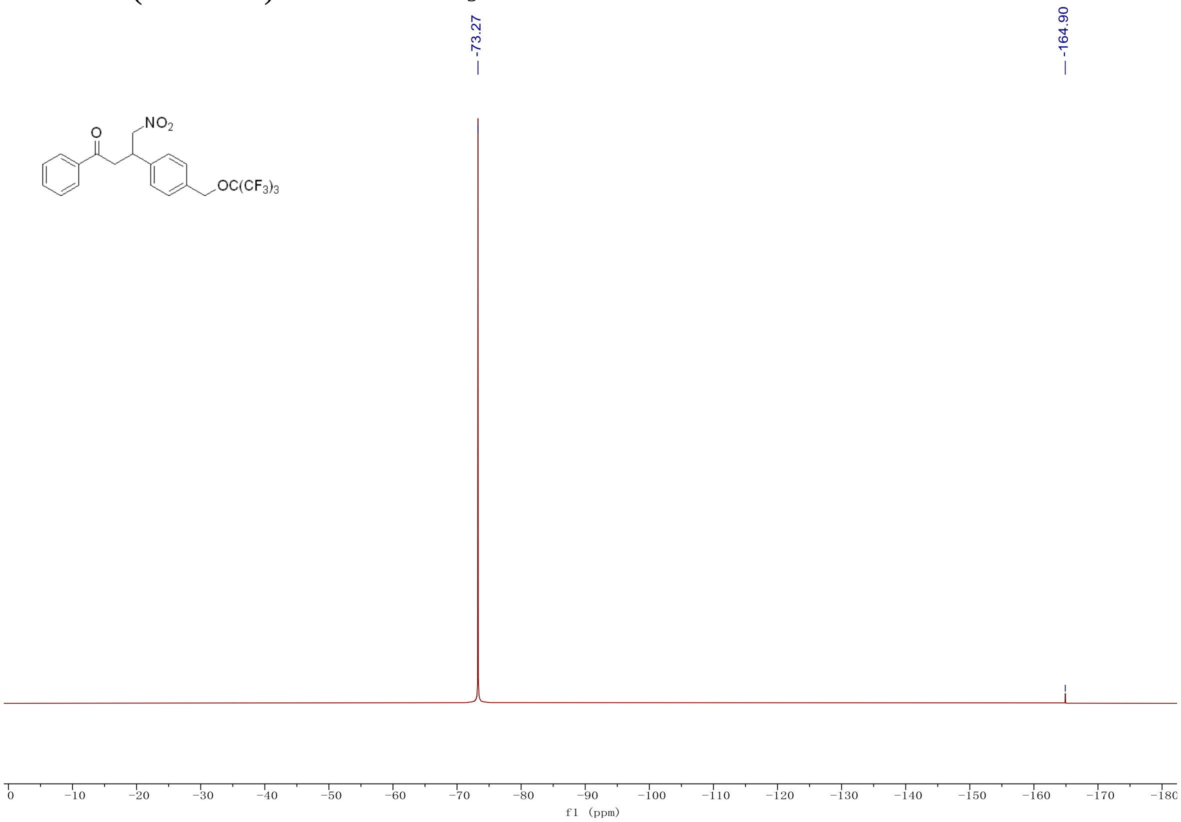

^{13}C NMR (126 MHz) of **1b** in $CDCl_3$

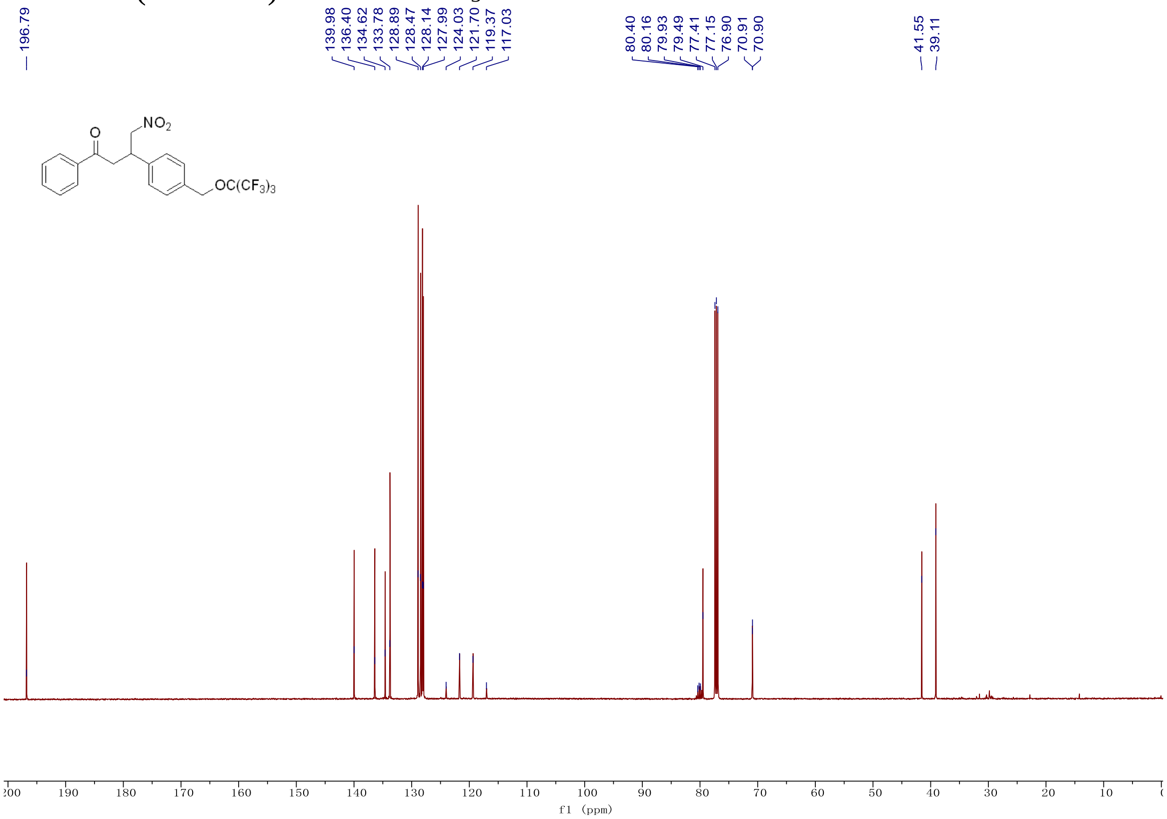

^{1}H NMR (400 MHz) of **2b** in $CDCl_3$

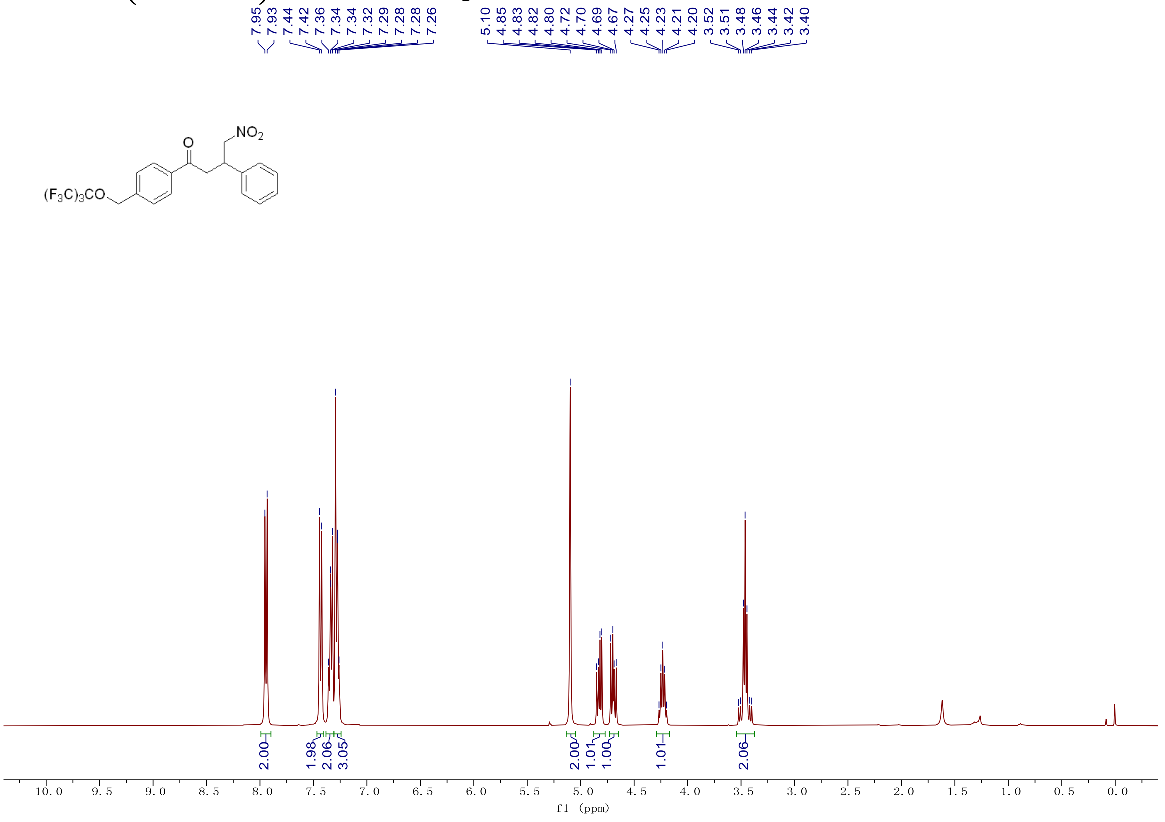

^{19}F NMR (376 MHz) of **2b** in $CDCl_3$

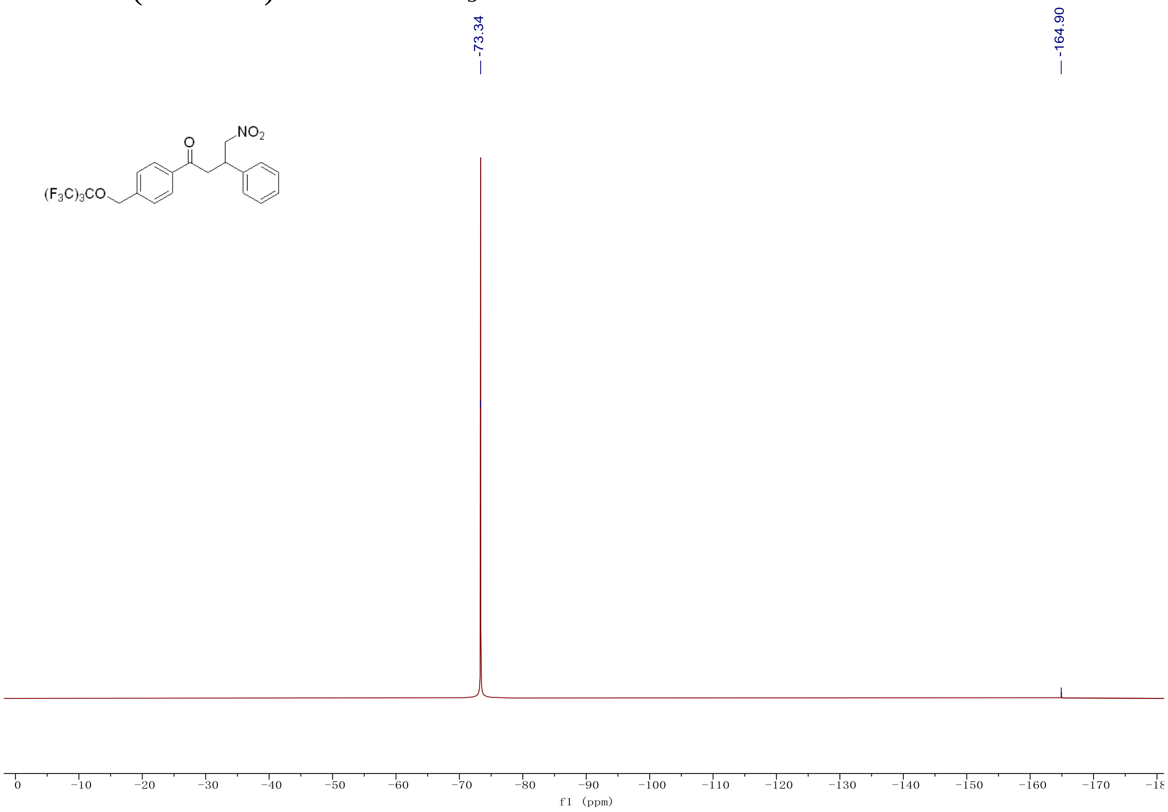

^{13}C NMR (151 MHz) of **2b** in $CDCl_3$

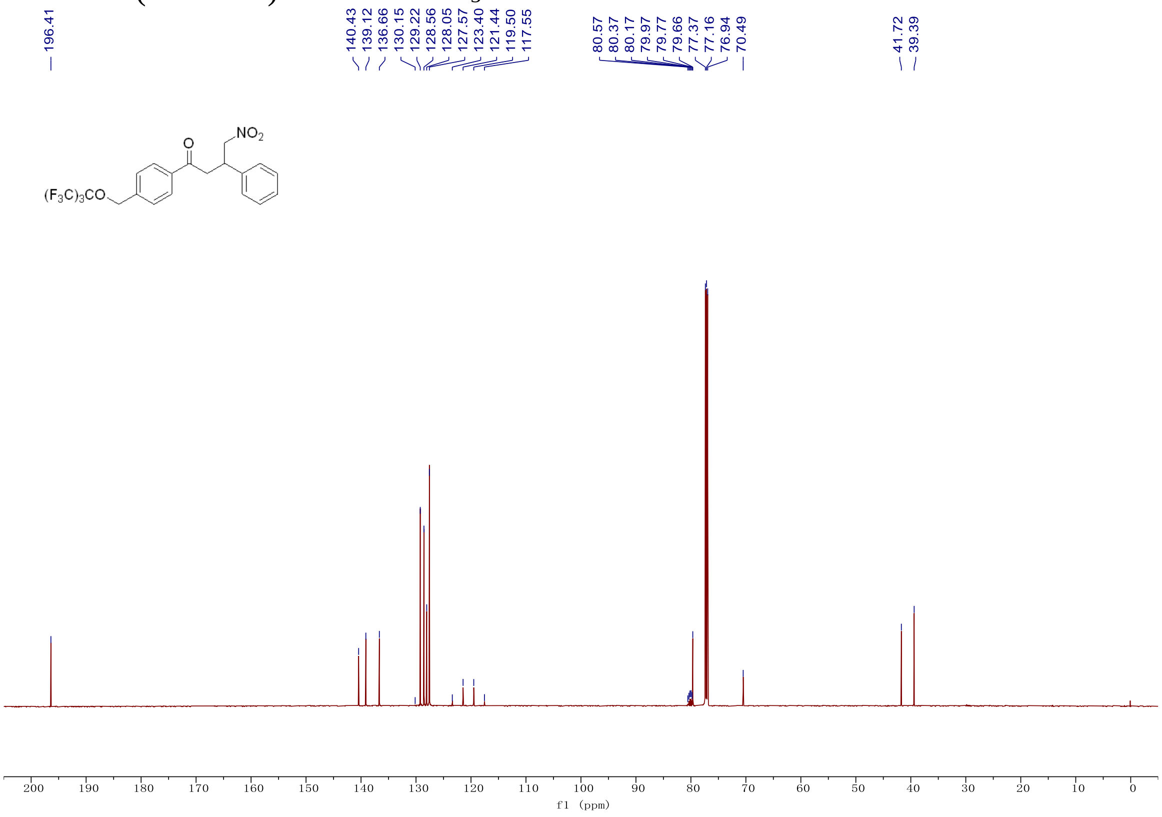

^{1}H NMR (500 MHz) of **3b** in $CDCl_3$

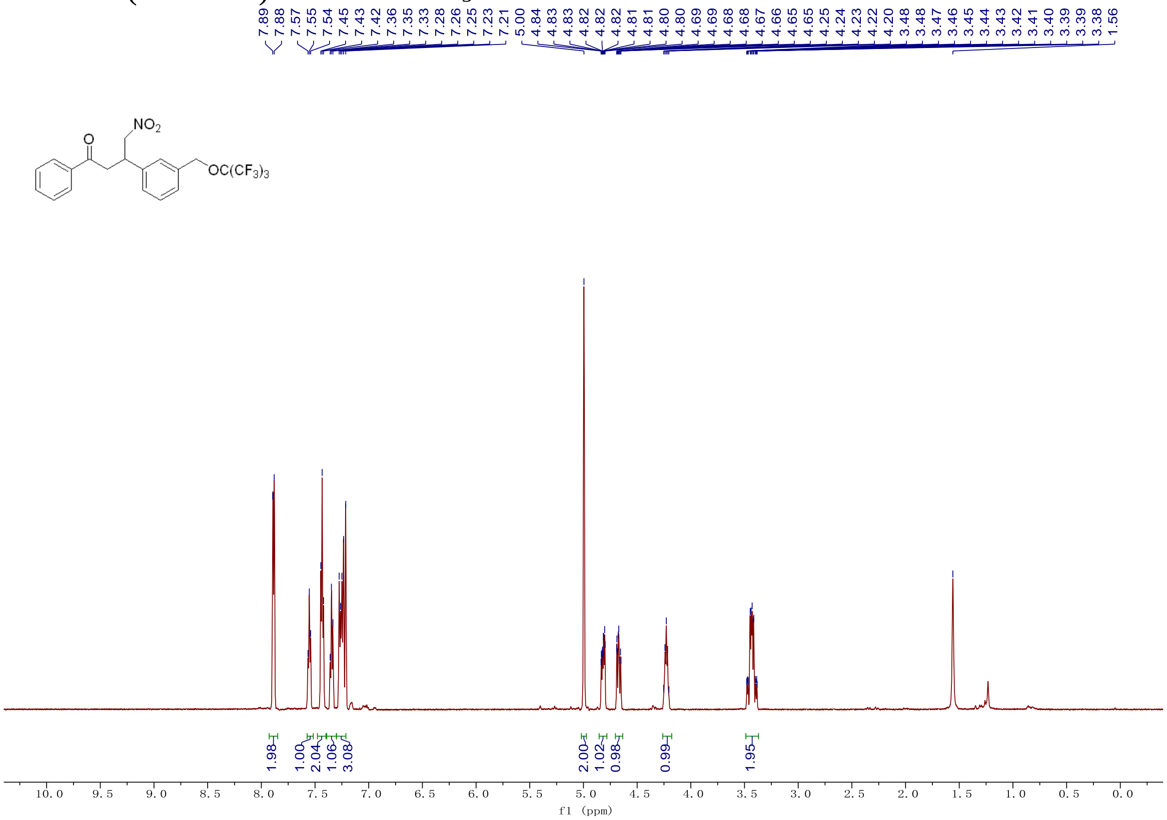

^{19}F NMR (471 MHz) of **3b** in $CDCl_3$

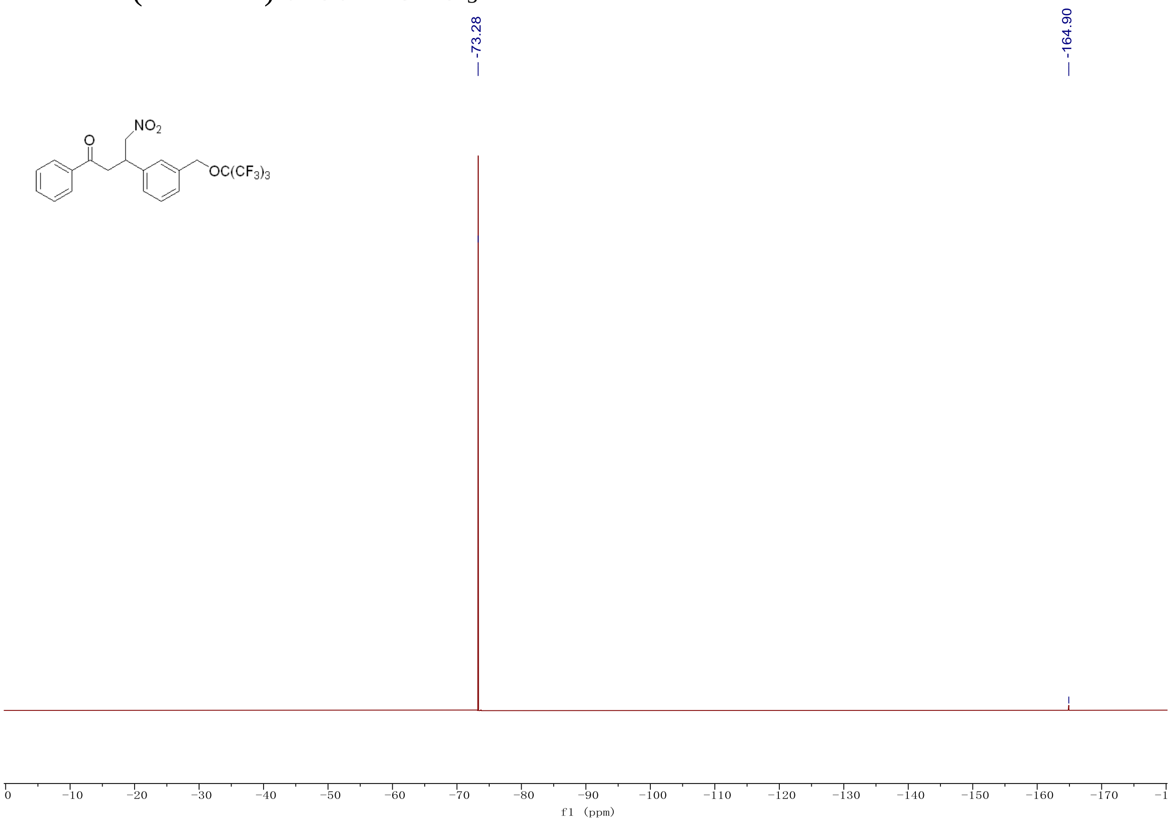

^{13}C NMR (126 MHz) of **3b** in $CDCl_3$

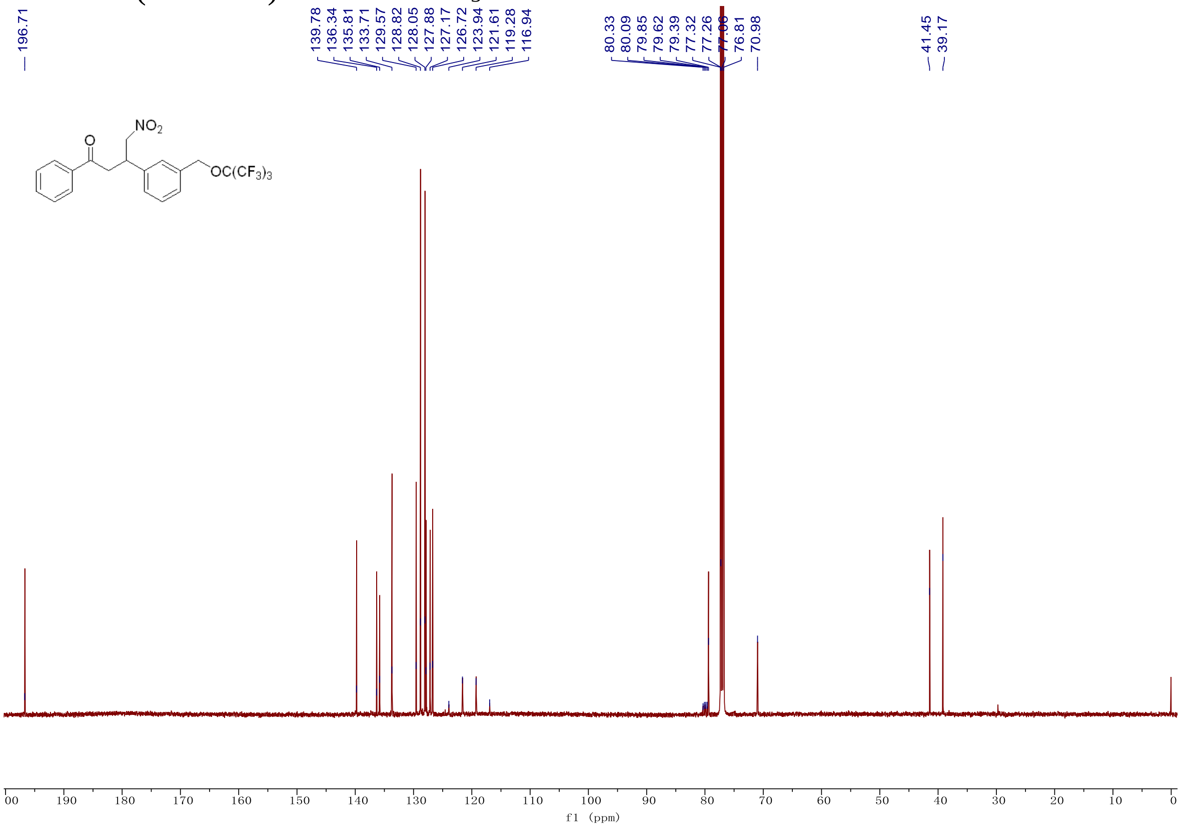

^{1}H NMR (600 MHz) of **4b** in $CDCl_3$

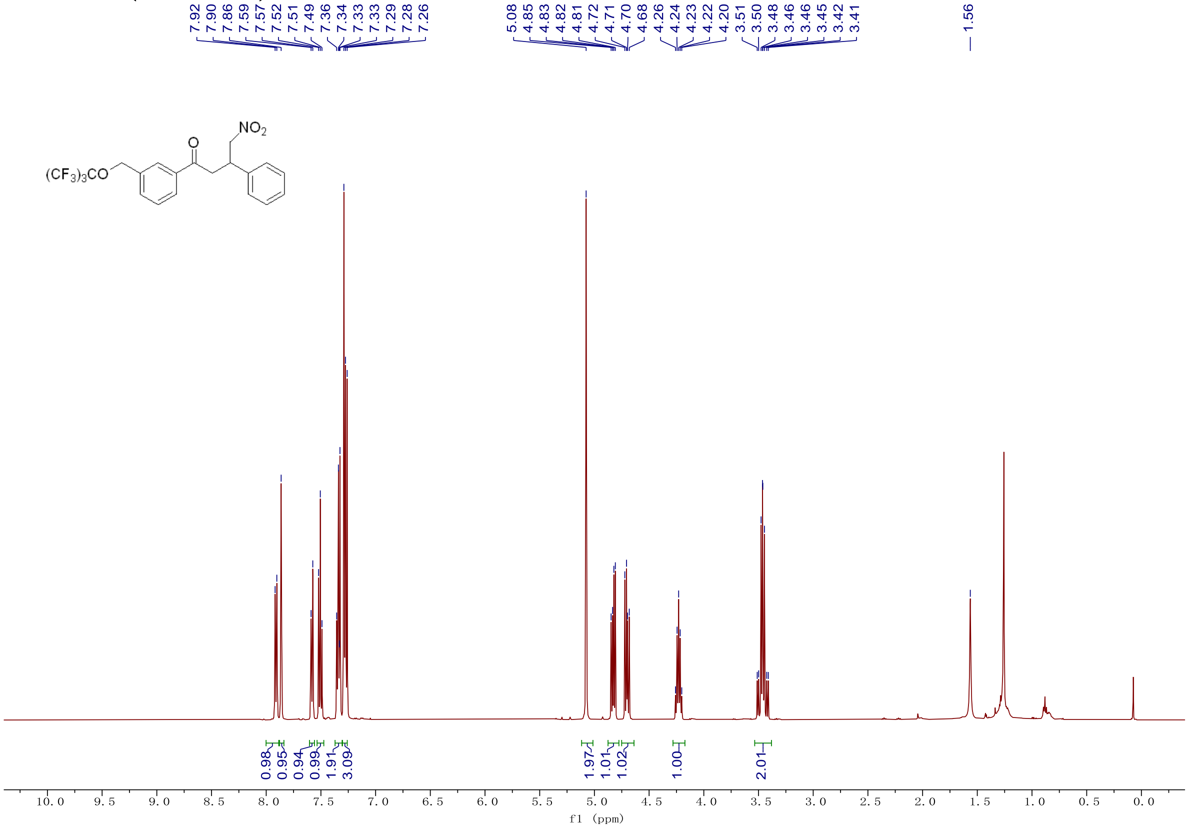

^{19}F NMR (471 MHz) of **4b** in $CDCl_3$

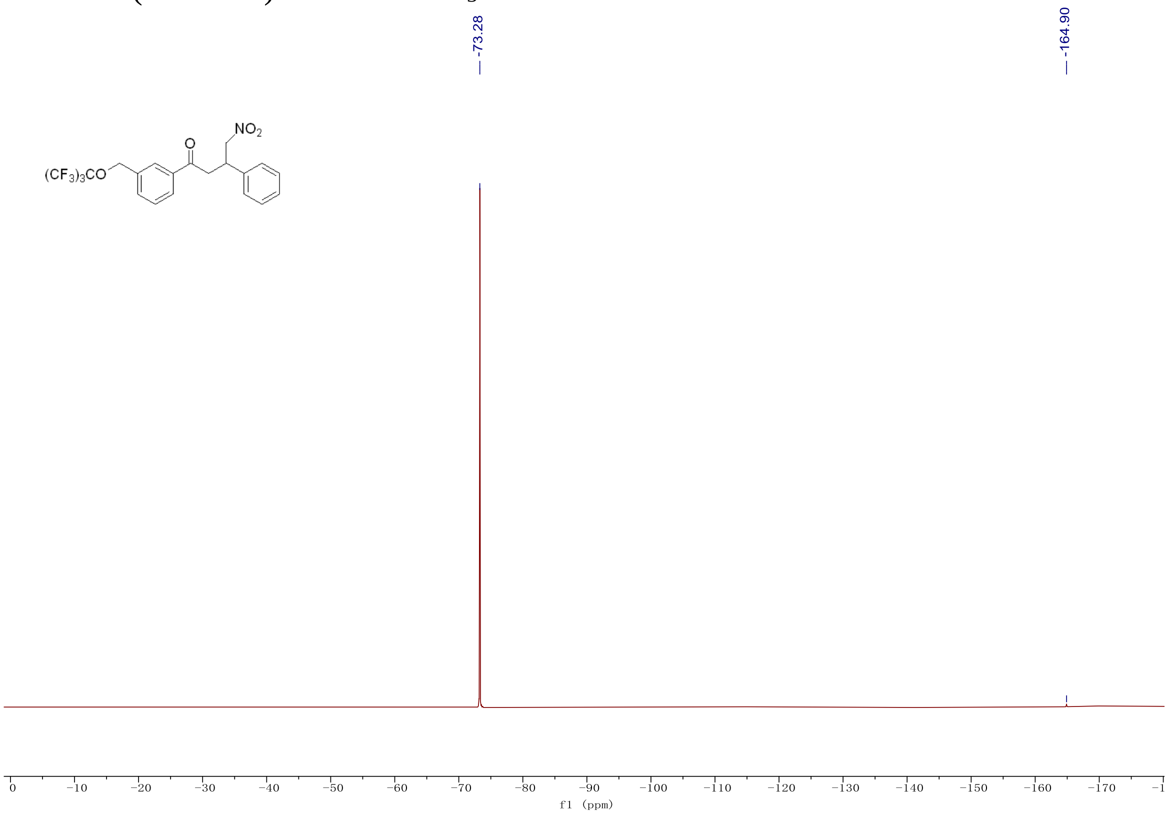

^{13}C NMR (126 MHz) of **4b** in $CDCl_3$

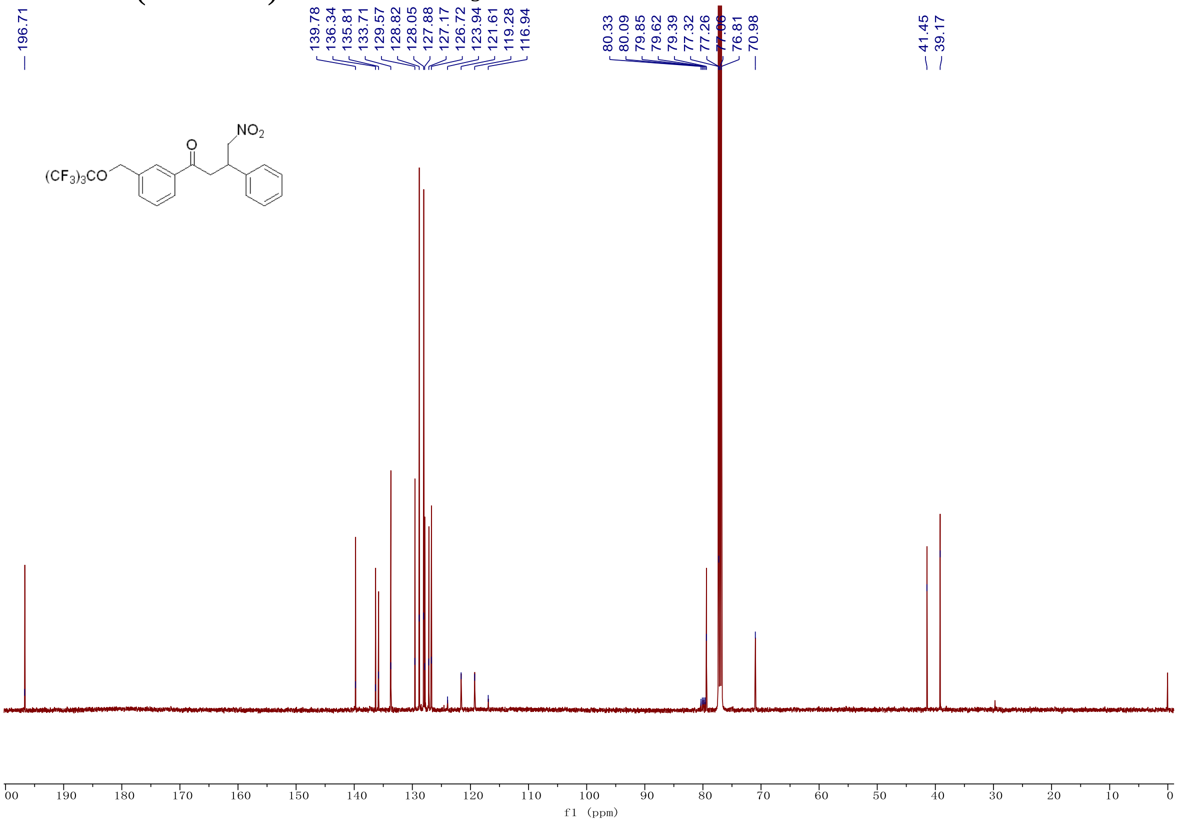

^{1}H NMR (400 MHz) of **5b** in $CDCl_3$

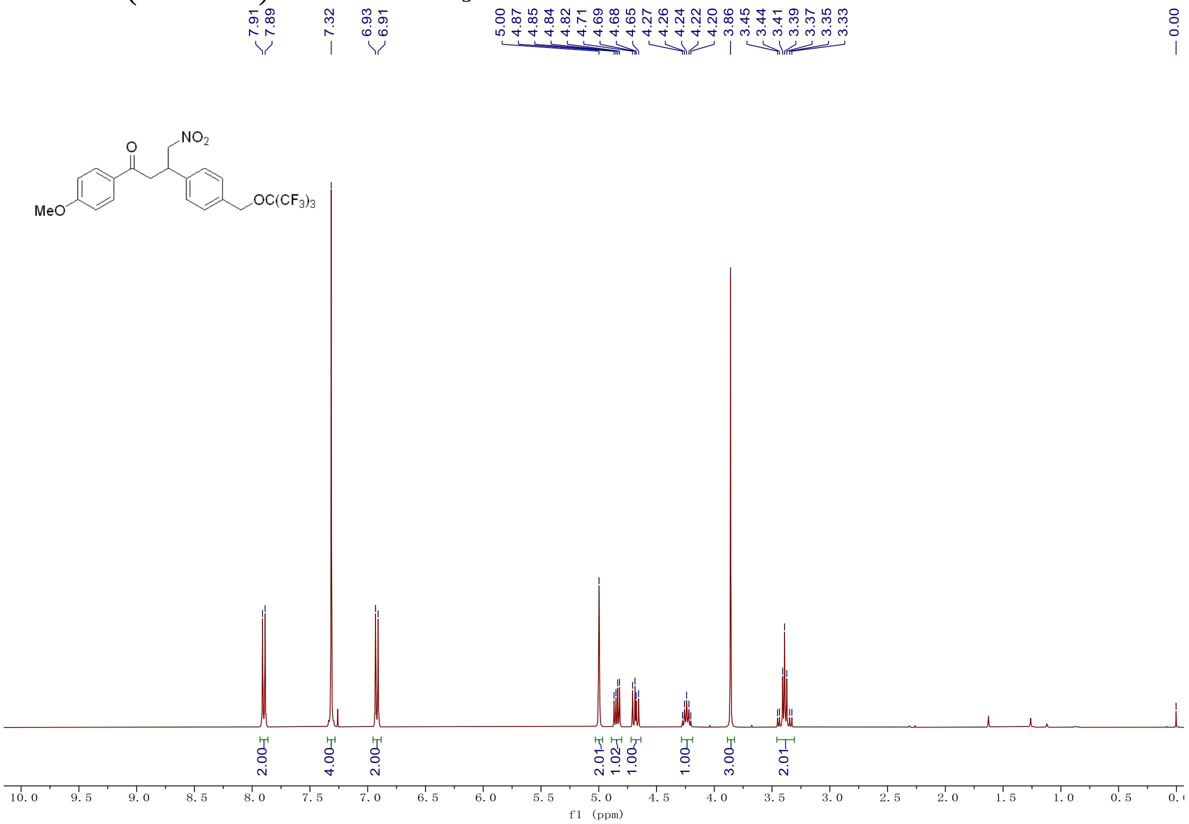

^{19}F NMR (376 MHz) of **5b** in $CDCl_3$

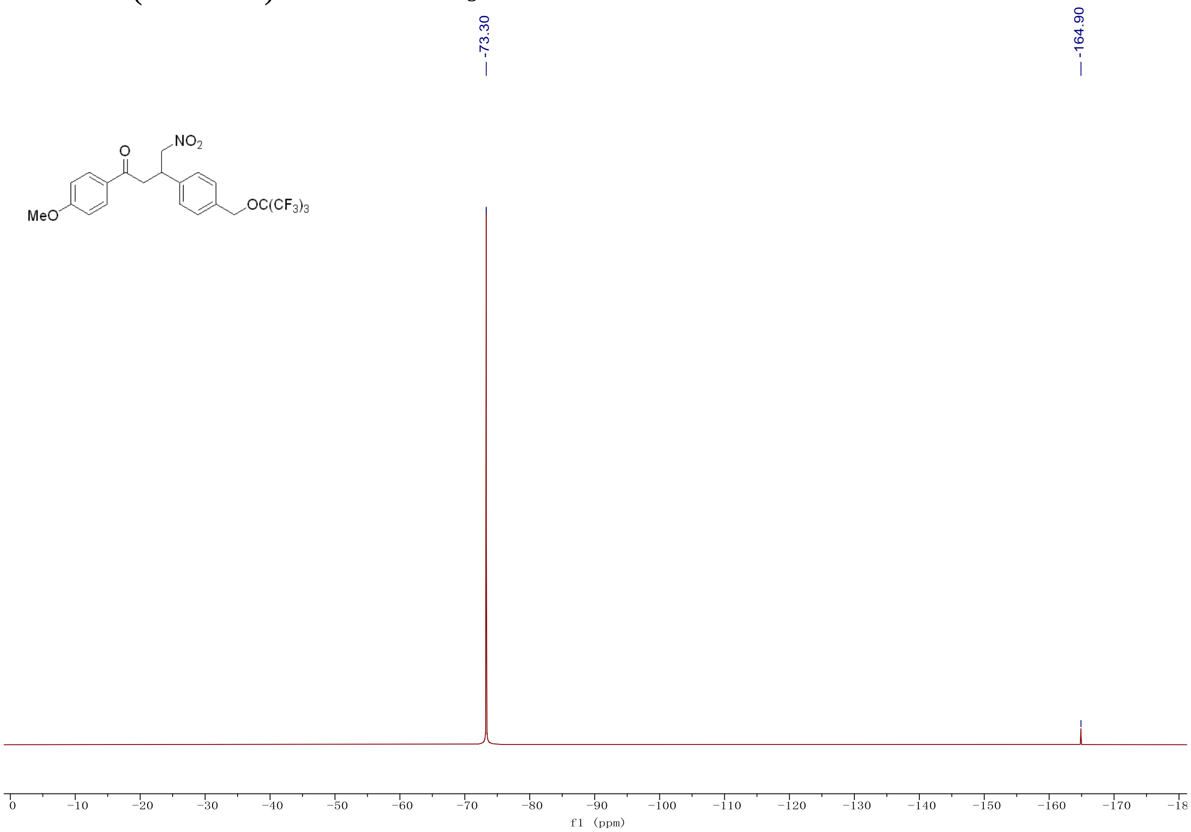

^{13}C NMR (151 MHz) of **5b** in $CDCl_3$

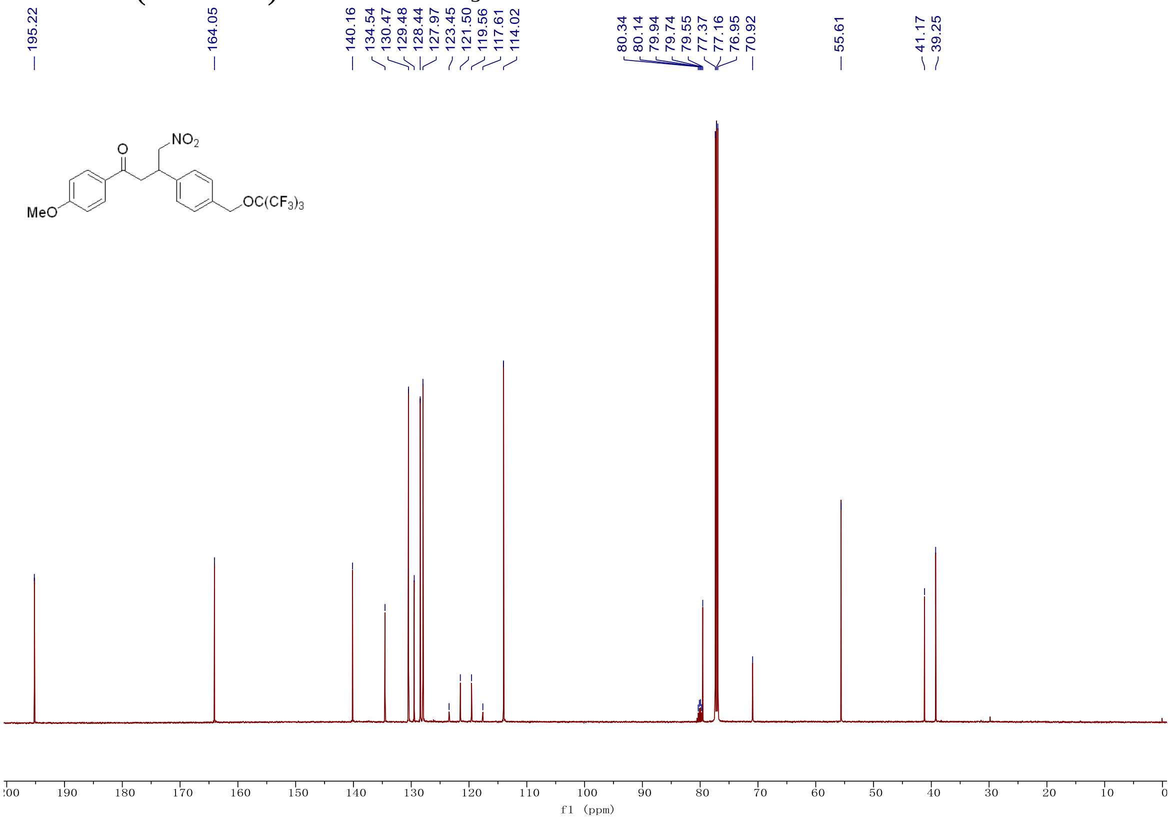

^{1}H NMR (400 MHz) of **6b** in $CDCl_3$

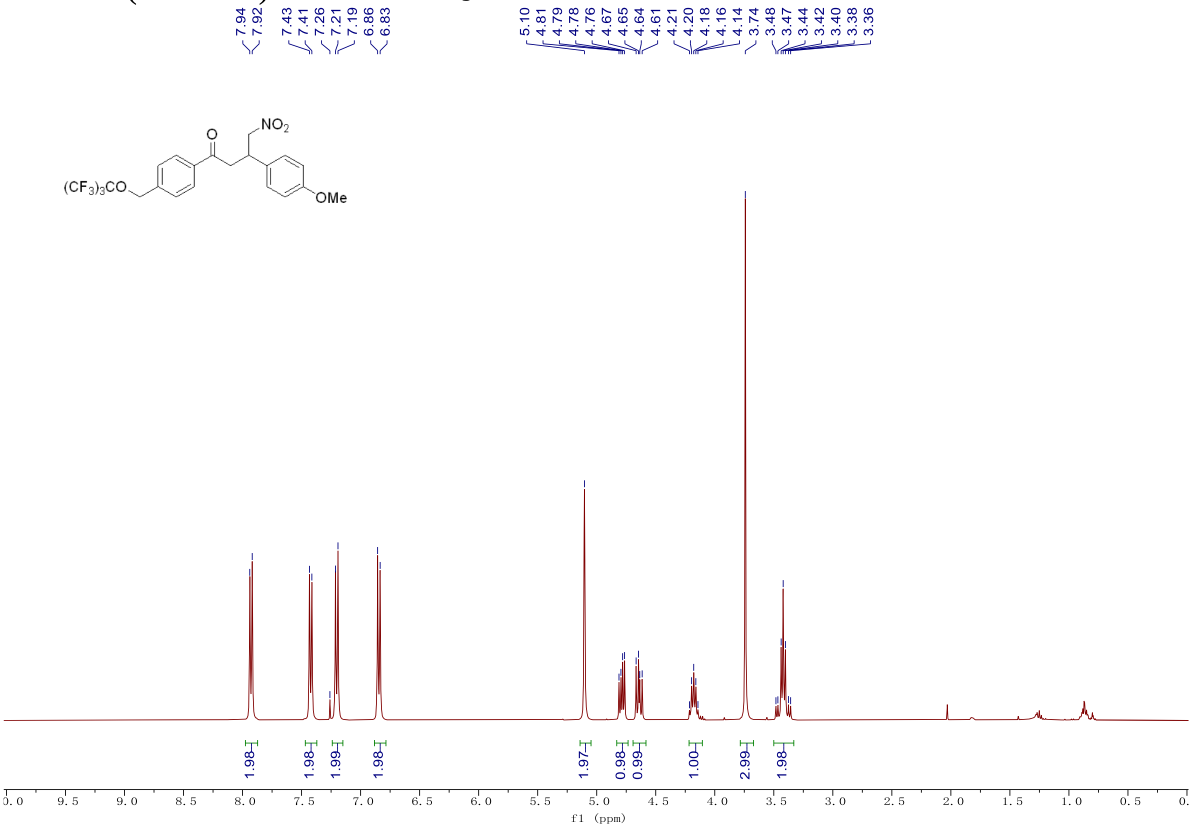

^{19}F NMR (376 MHz) of **6b** in $CDCl_3$

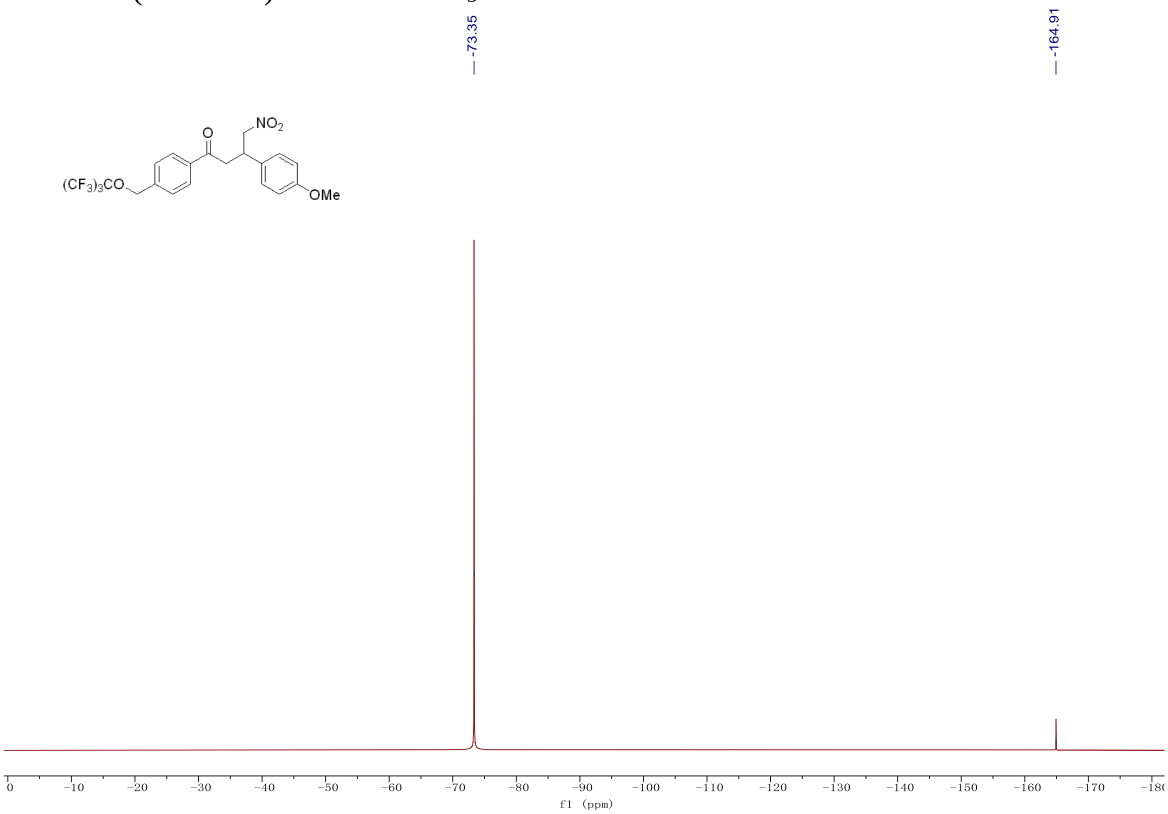

^{13}C NMR (101 MHz) of **6b** in $CDCl_3$

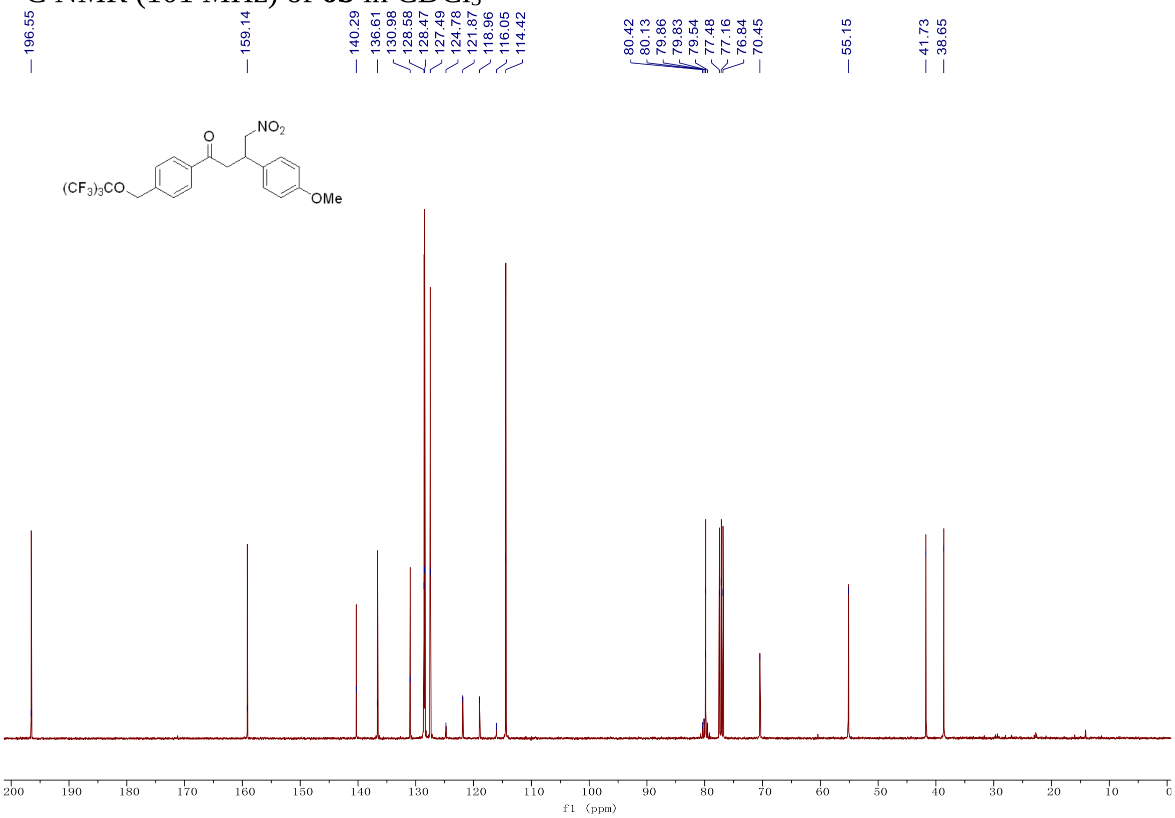

^{1}H NMR (400 MHz) of **7b** in $CDCl_3$

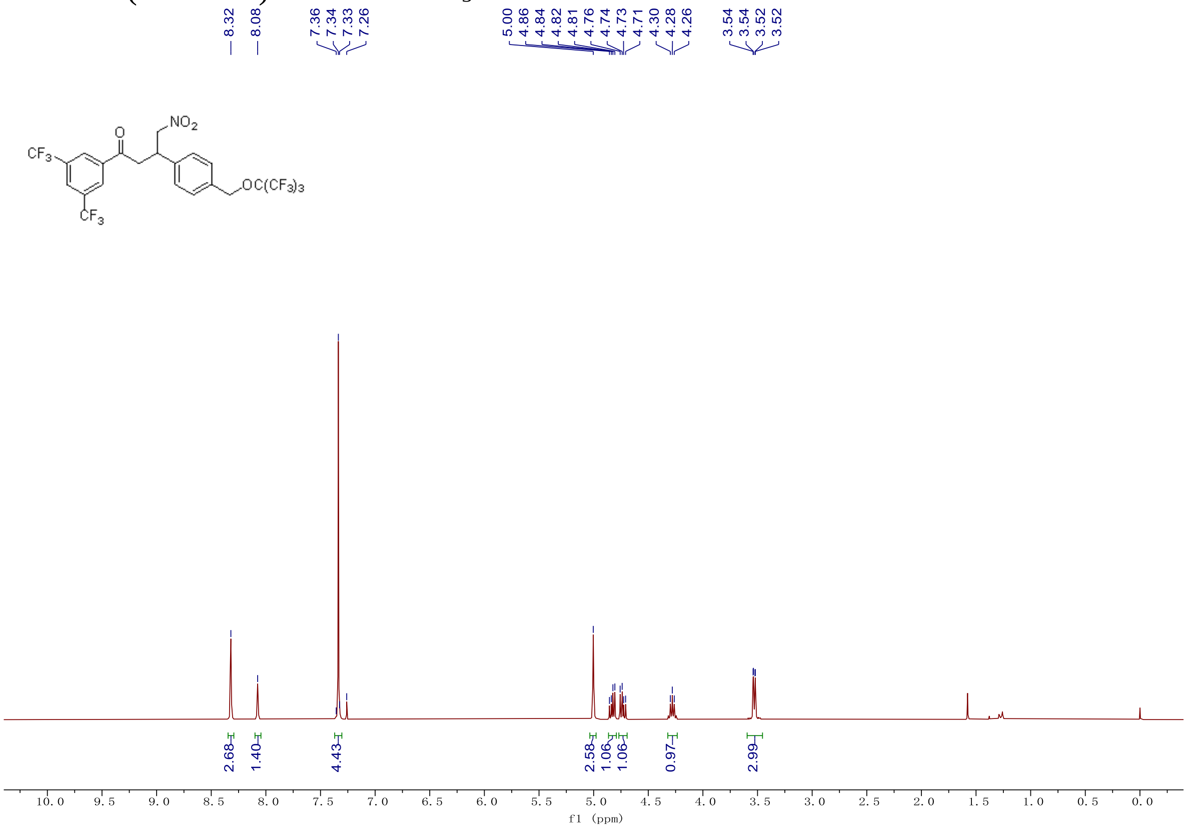

^{19}F NMR (376 MHz) of **7b** in $CDCl_3$

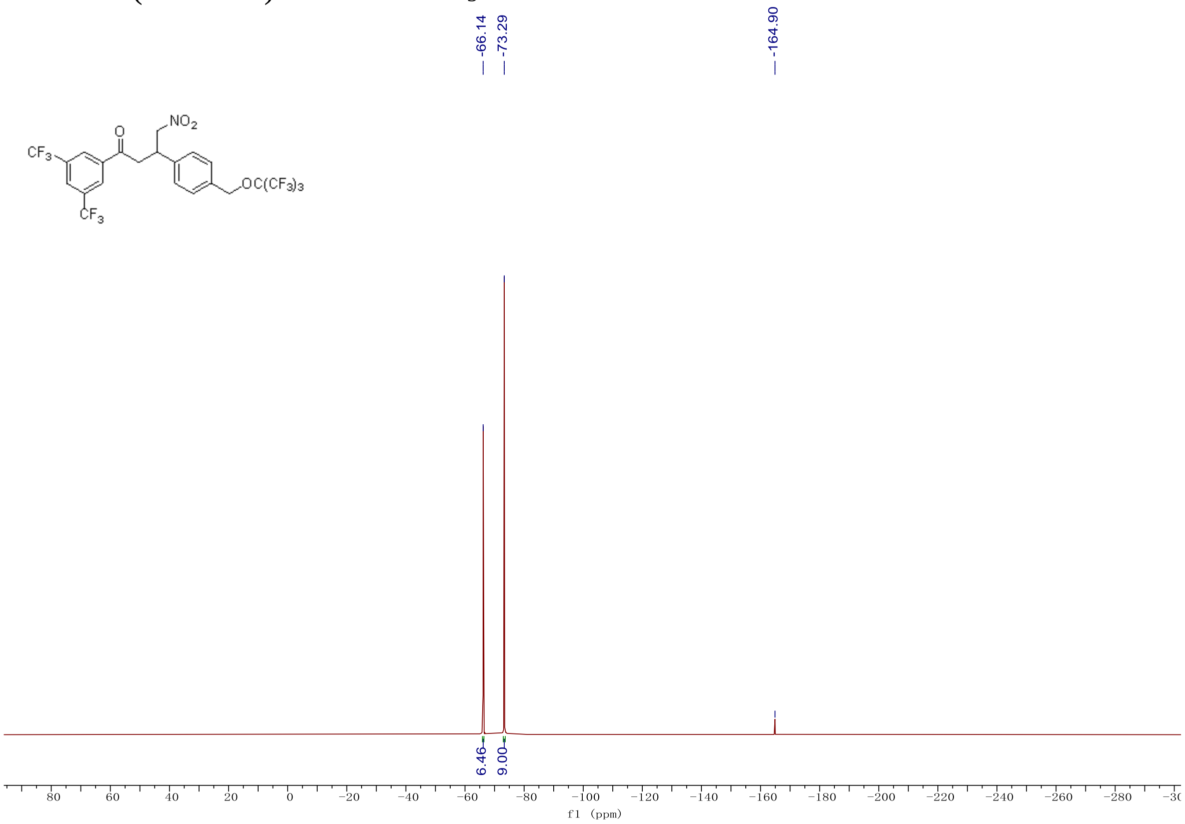

^{13}C NMR (151 MHz) of **7b** in $CDCl_3$

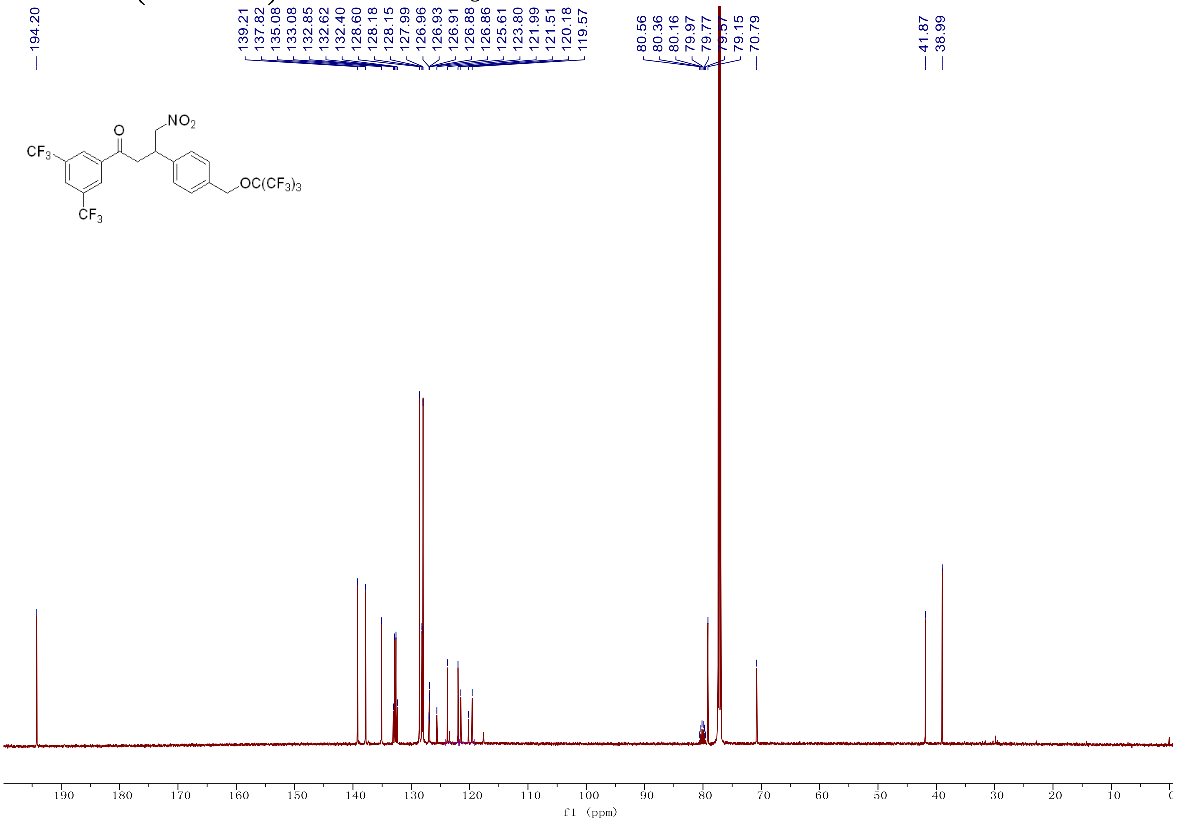

^{1}H NMR (500 MHz) of **8b** in $CDCl_3$

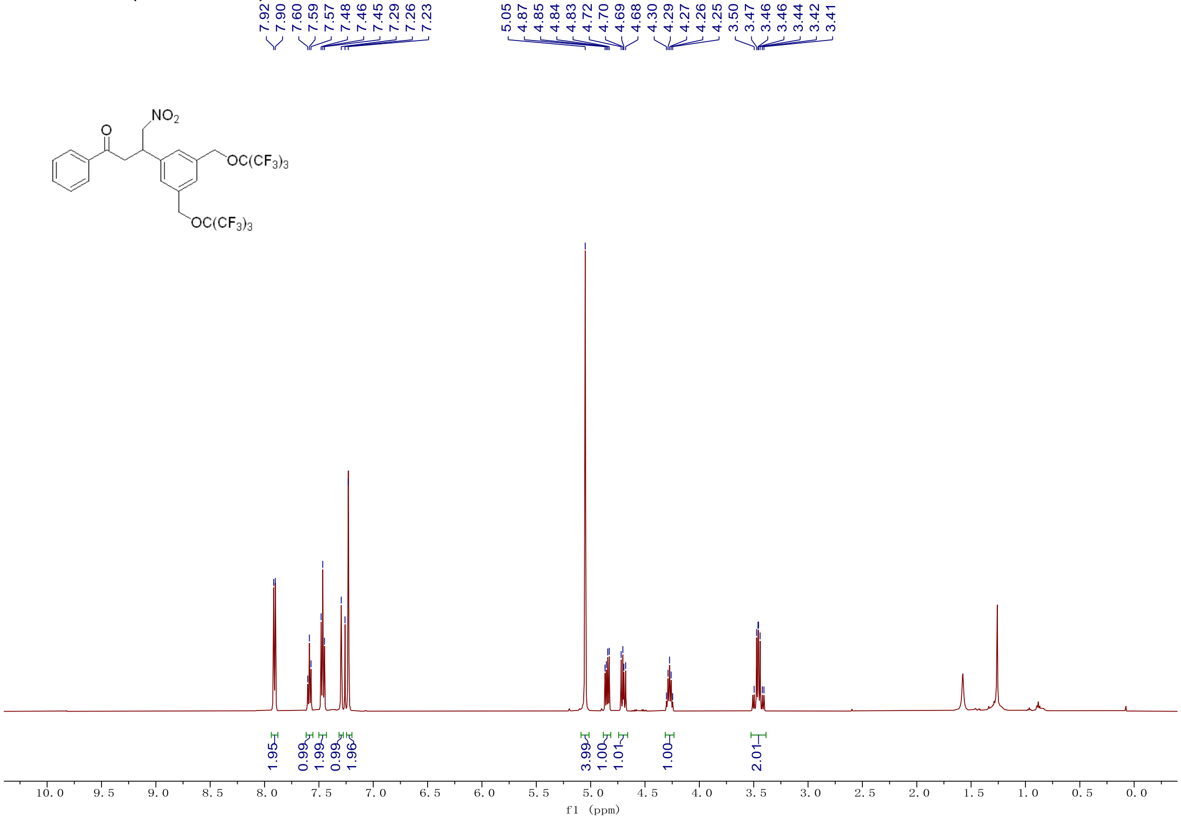

^{19}F NMR (471 MHz) of **8b** in $CDCl_3$

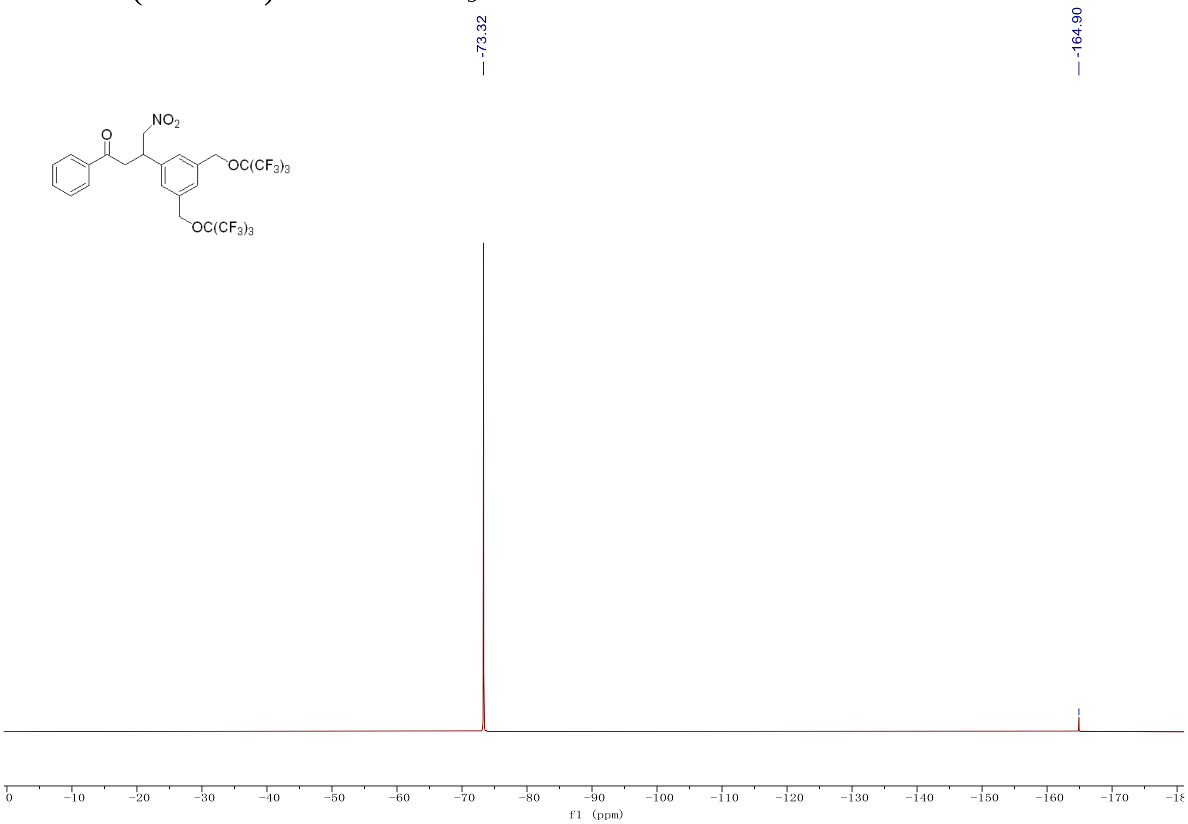

^{13}C NMR (126 MHz) of **8b** in $CDCl_3$

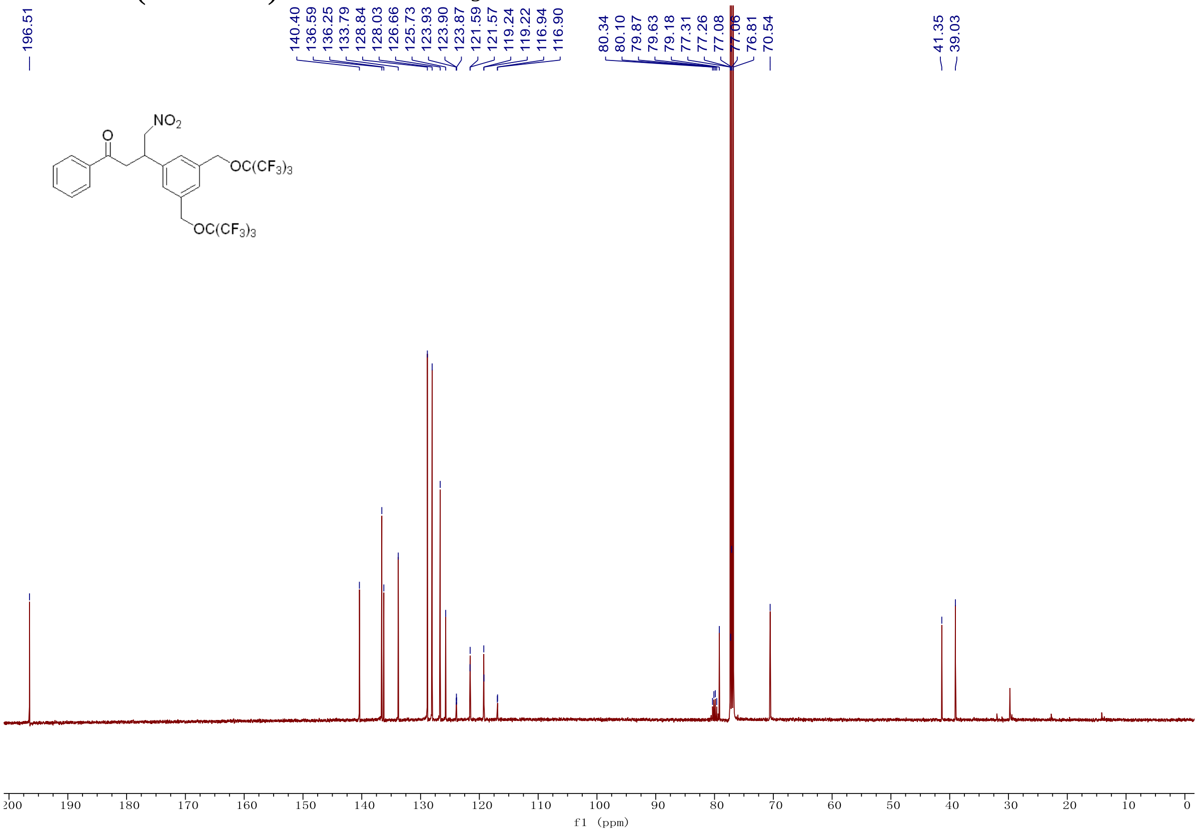

^{1}H NMR (500 MHz) of **9b** in $CDCl_3$

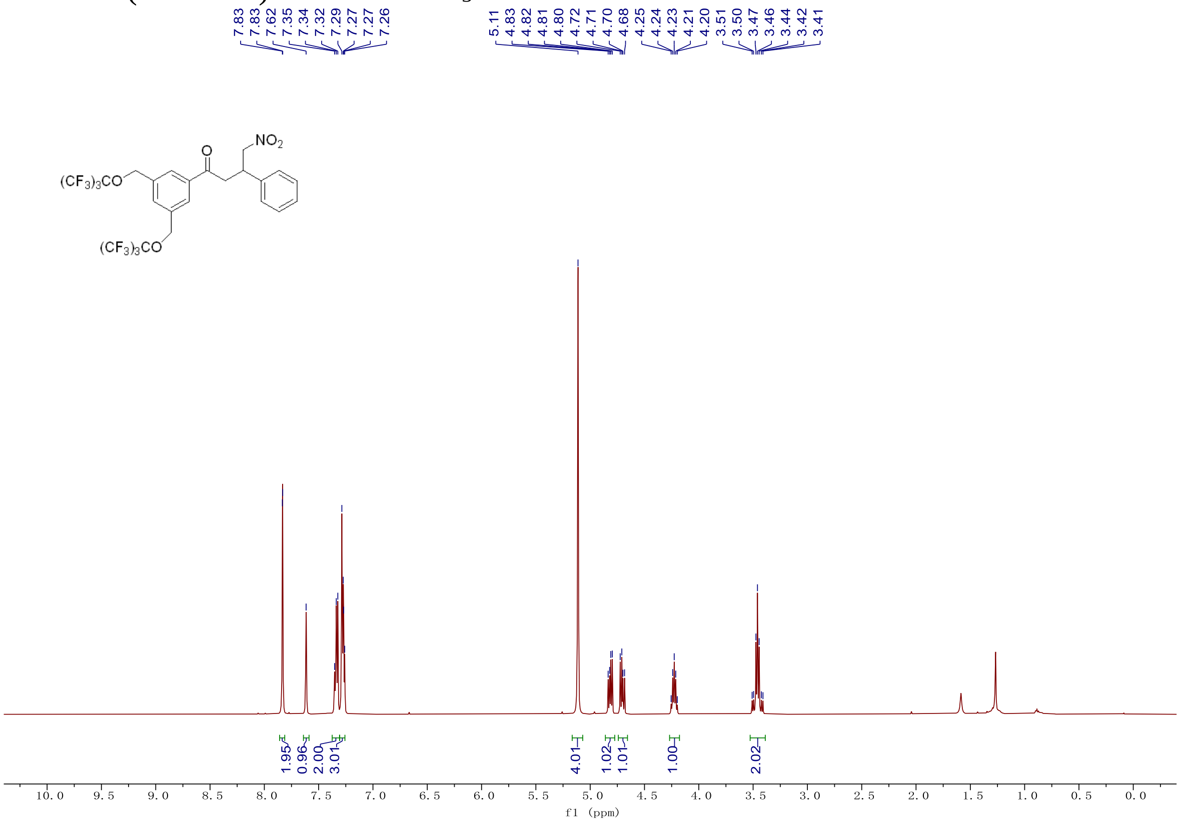

^{19}F NMR (471 MHz) of **9b** in $CDCl_3$

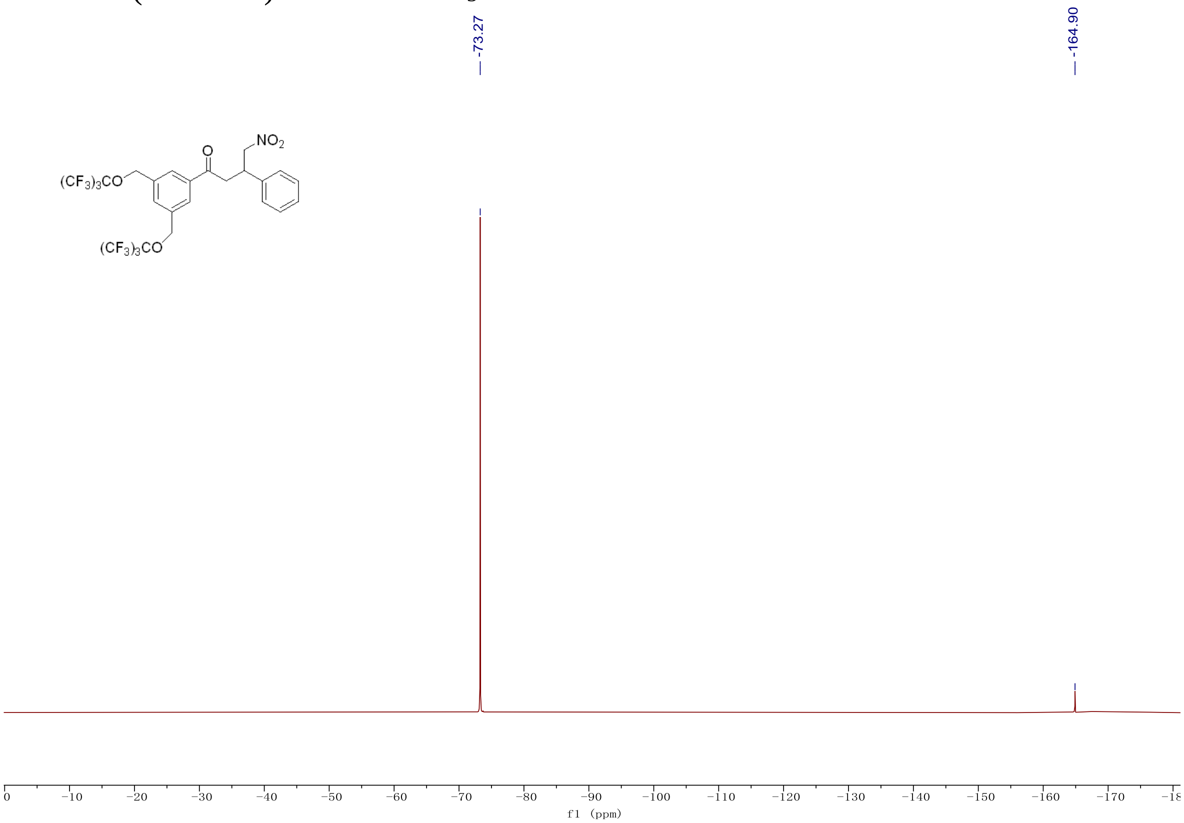

^{13}C NMR (126 MHz) of **9b** in $CDCl_3$

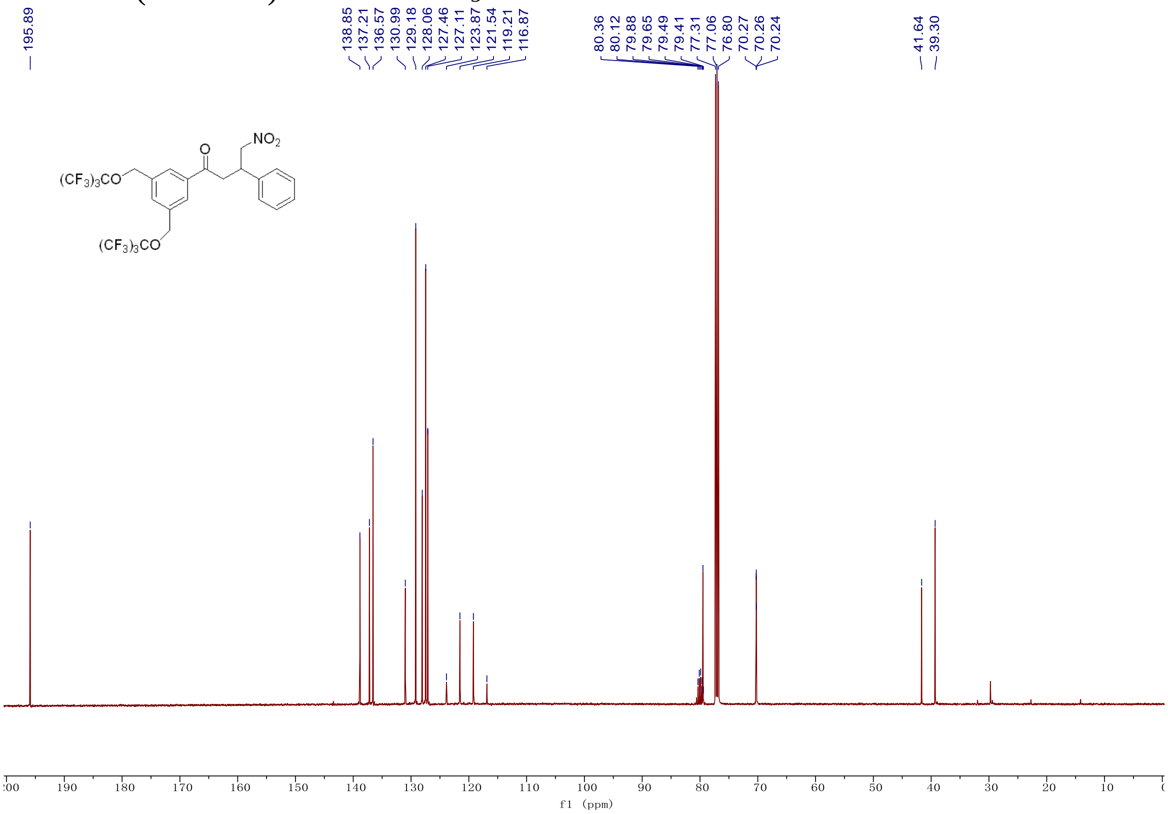

^{1}H NMR (500 MHz) of **10b** in $CDCl_3$

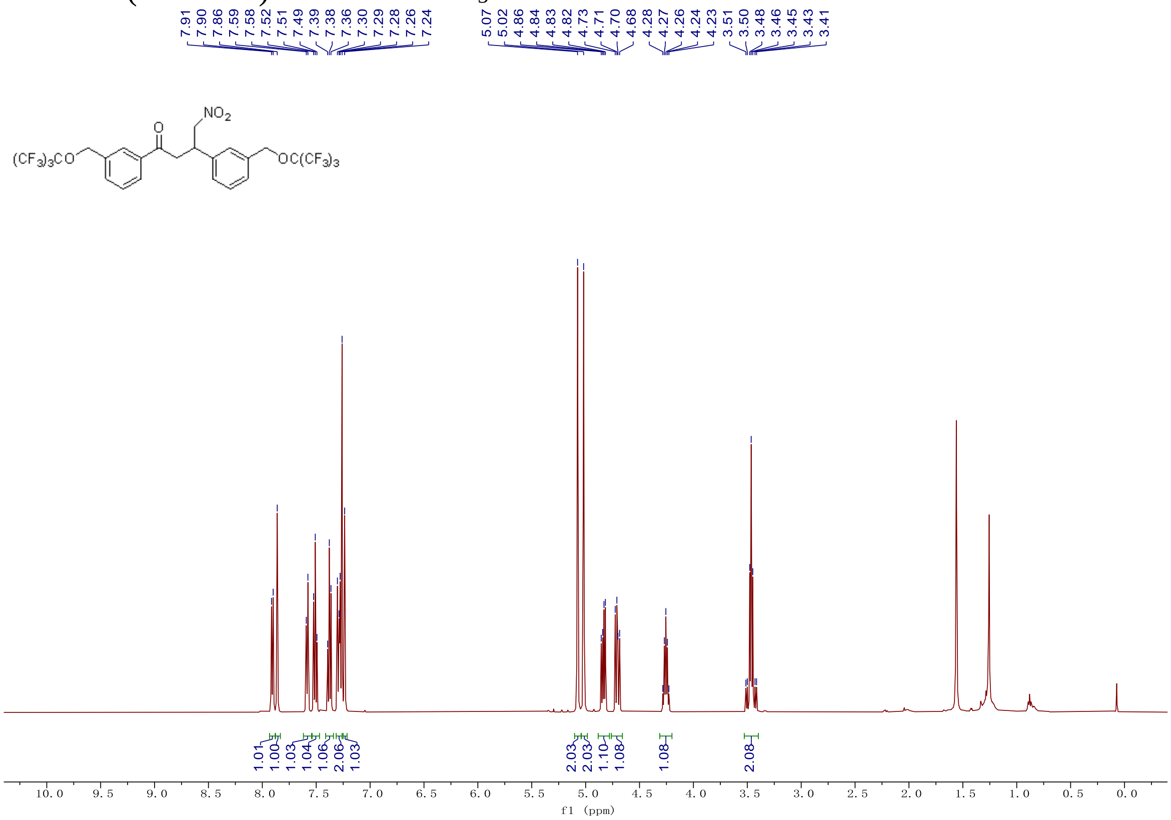

^{19}F NMR (471 MHz) of **10b** in $CDCl_3$

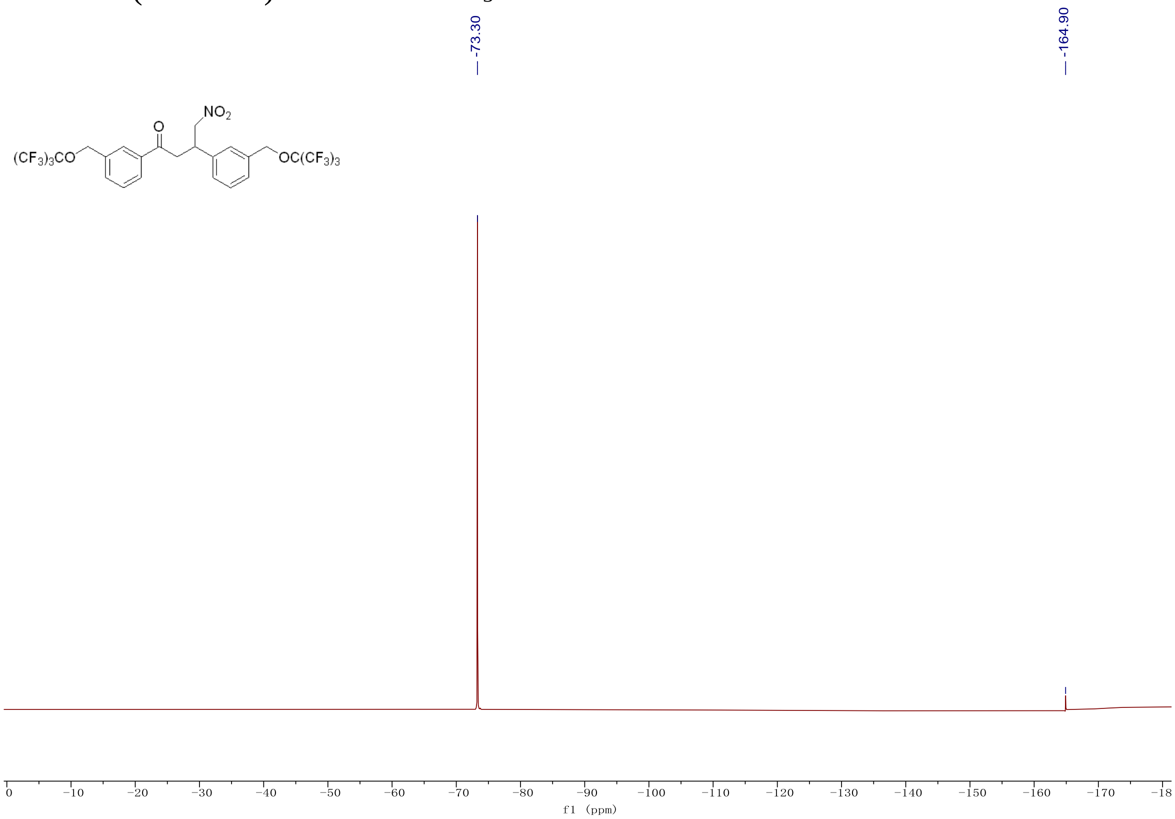

^{13}C NMR (126 MHz) of **10b** in $CDCl_3$

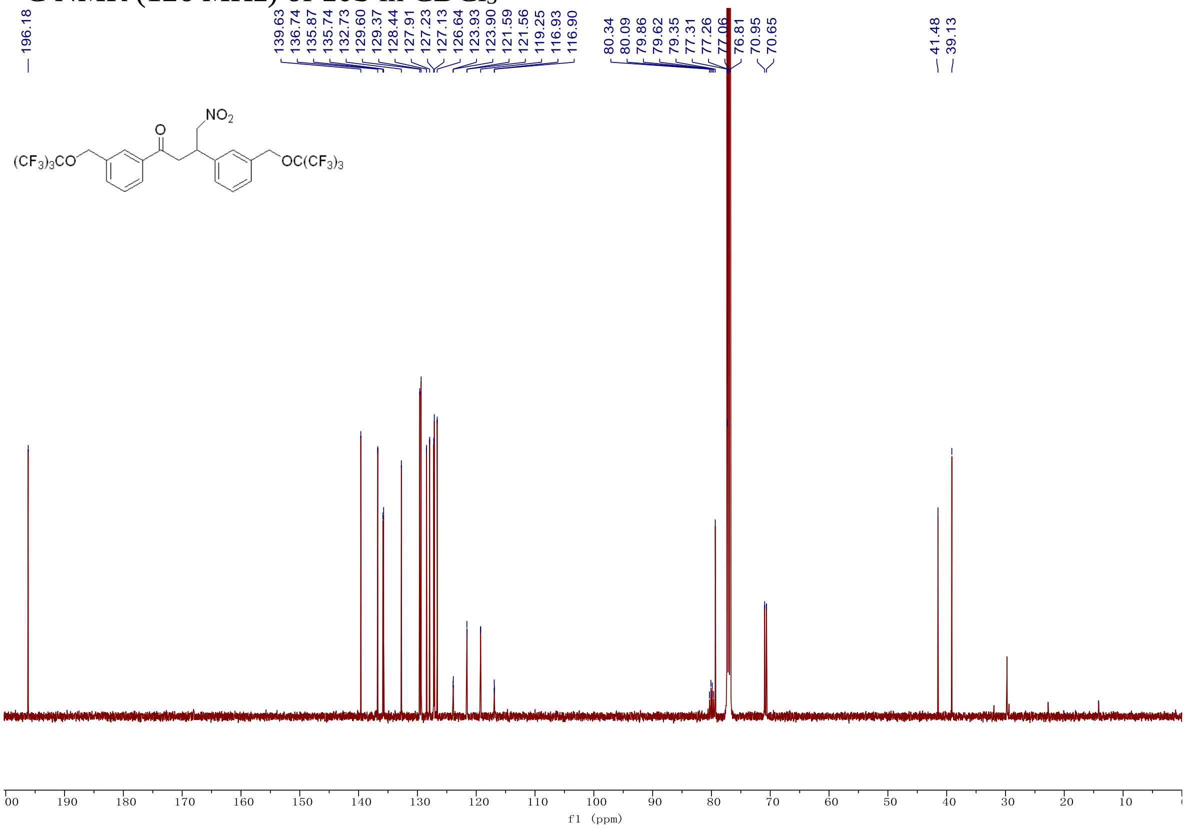

^{1}H NMR (500 MHz) of **PS1** in $CDCl_3$

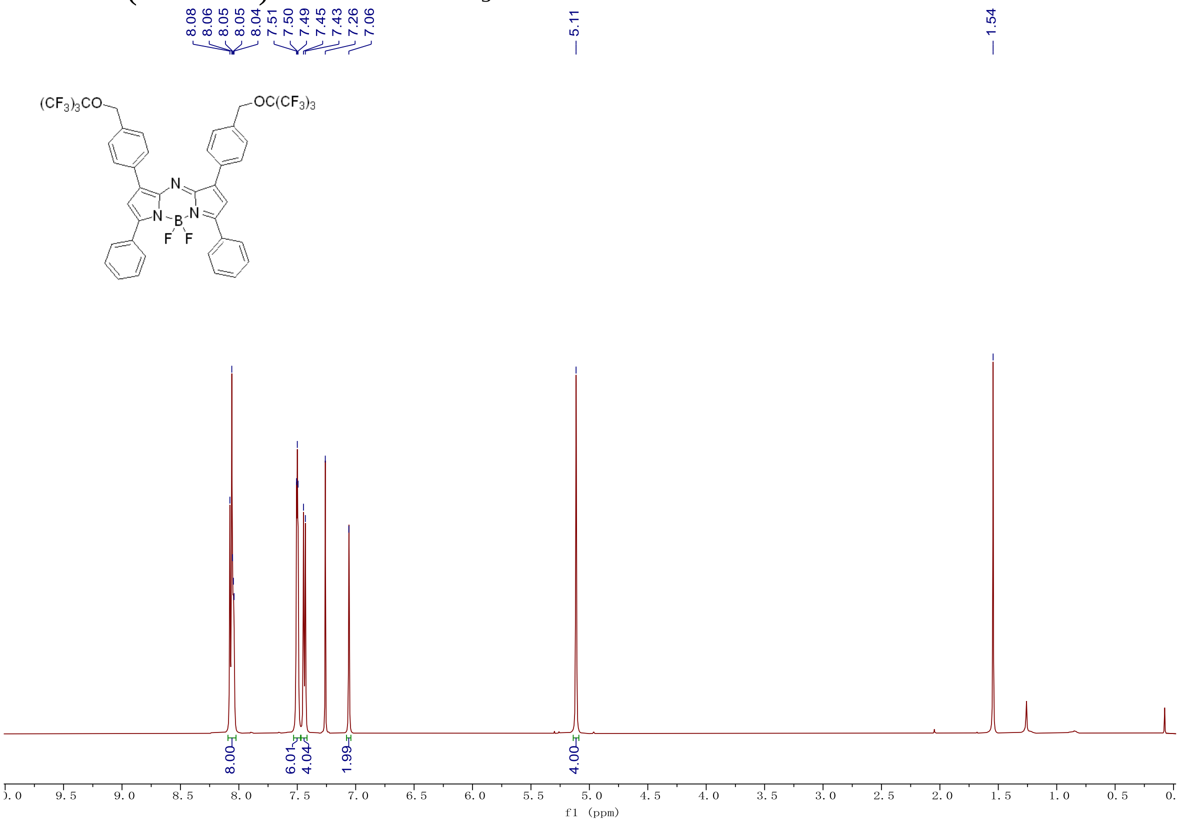

^{19}F NMR (471 MHz) of **PS1** in $CDCl_3$

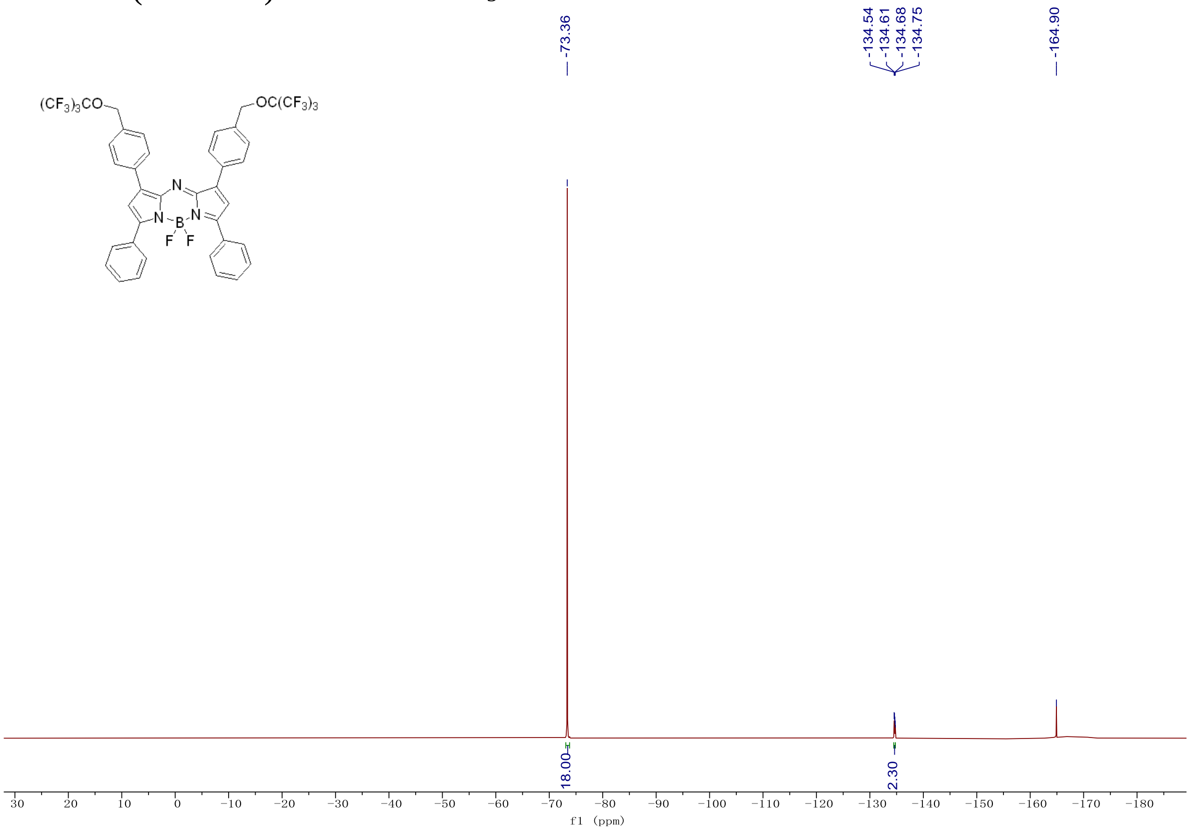

^{13}C NMR (126 MHz) of **PS1** in $CDCl_3$

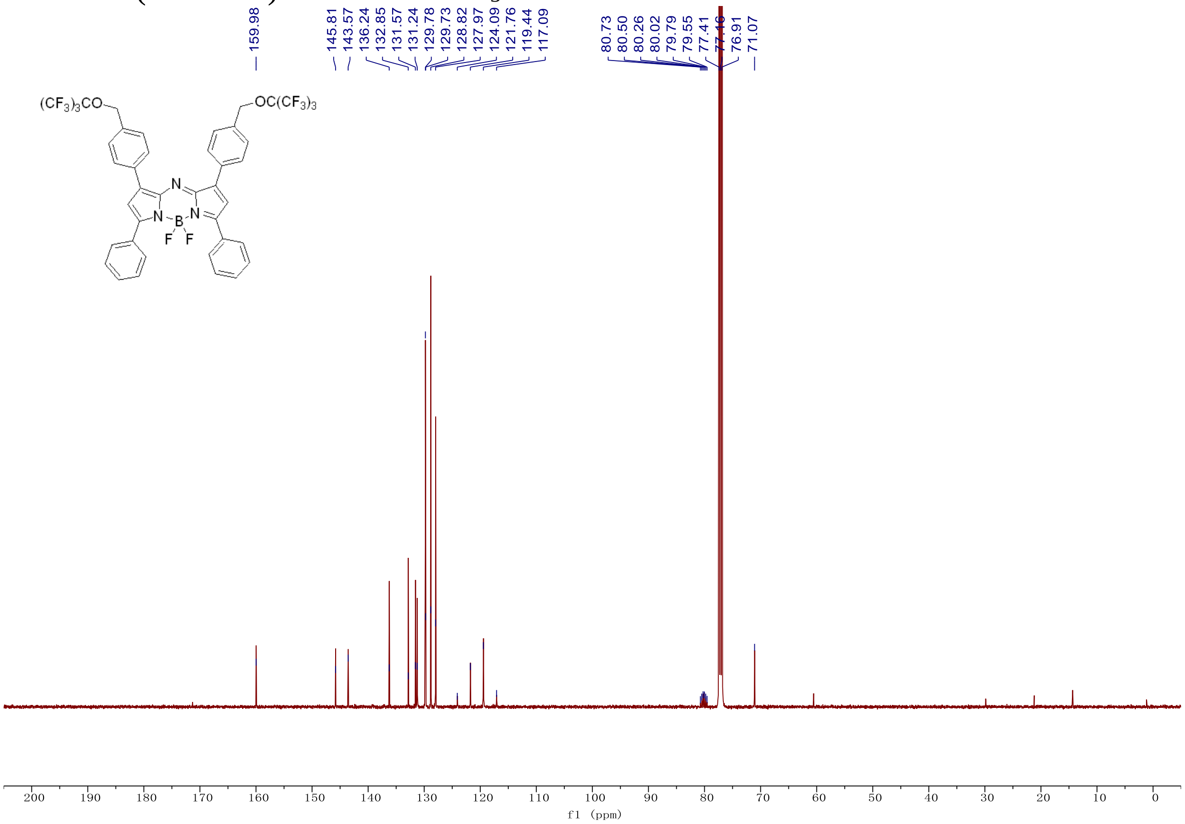

^{1}H NMR (400 MHz) of **PS2** in $CDCl_3$

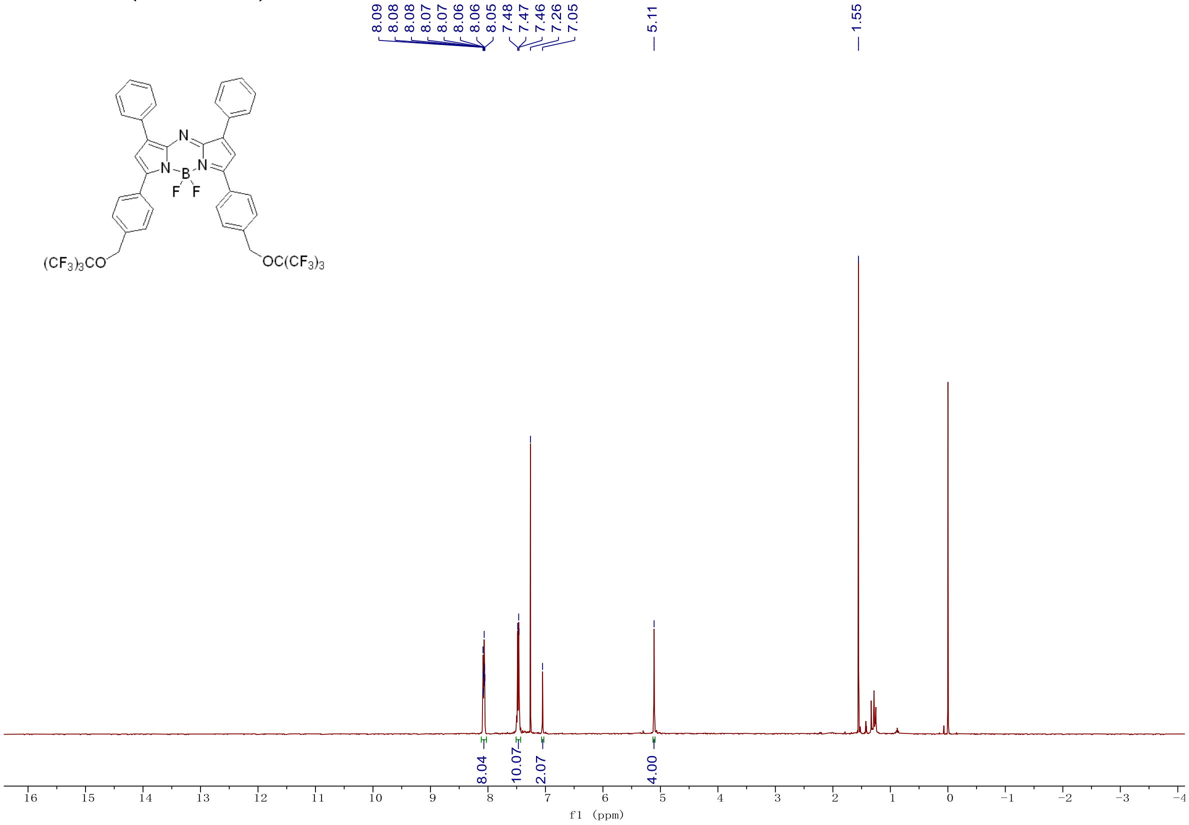

^{19}F NMR (376 MHz) of **PS2** in $CDCl_3$

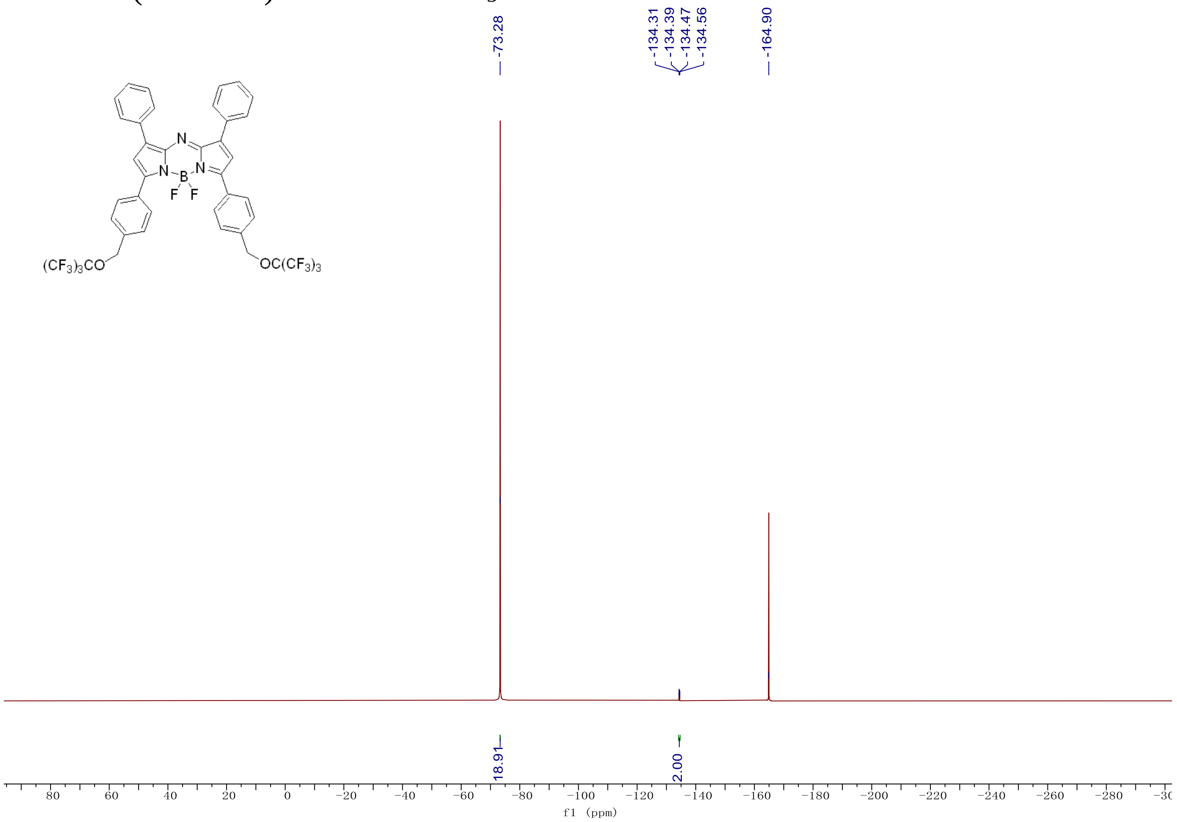

^{13}C NMR (151 MHz) of **PS2** in $CDCl_3$

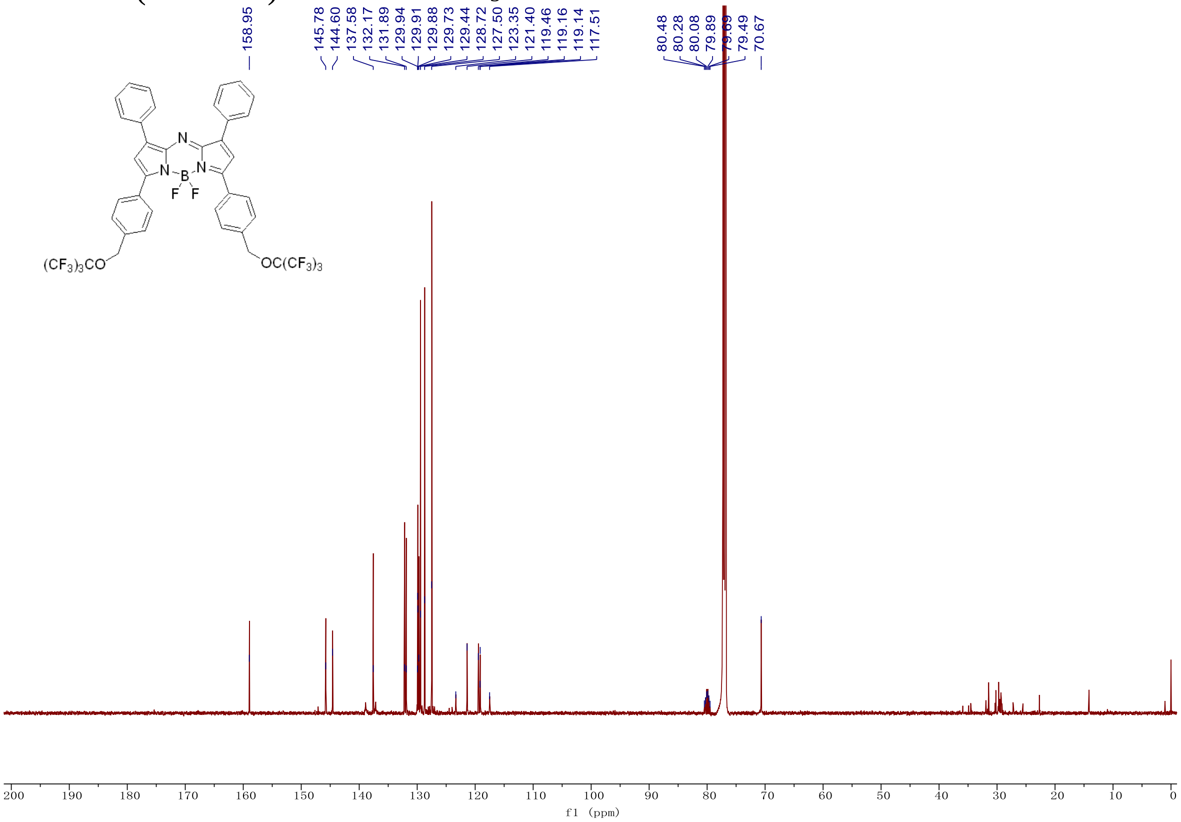

^{1}H NMR (500 MHz) of **PS3** in $CDCl_3$

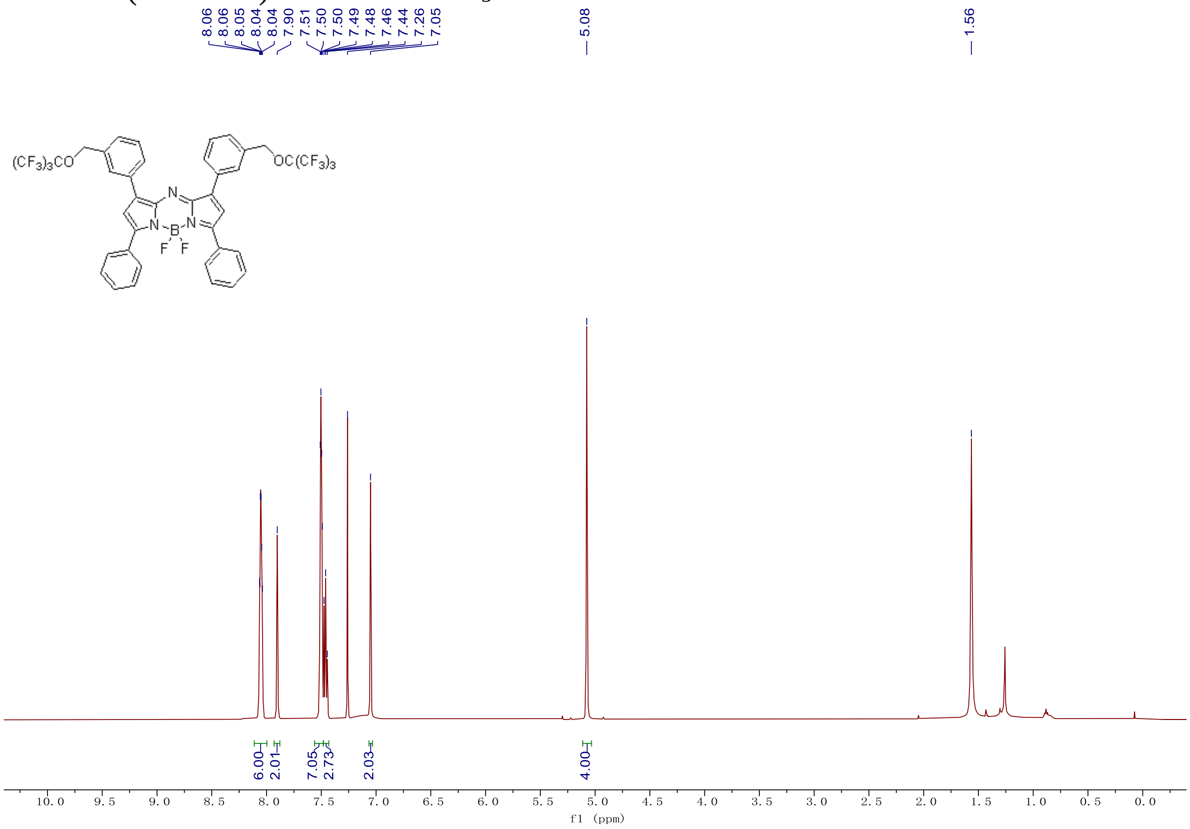

^{19}F NMR (471 MHz) of **PS3** in $CDCl_3$

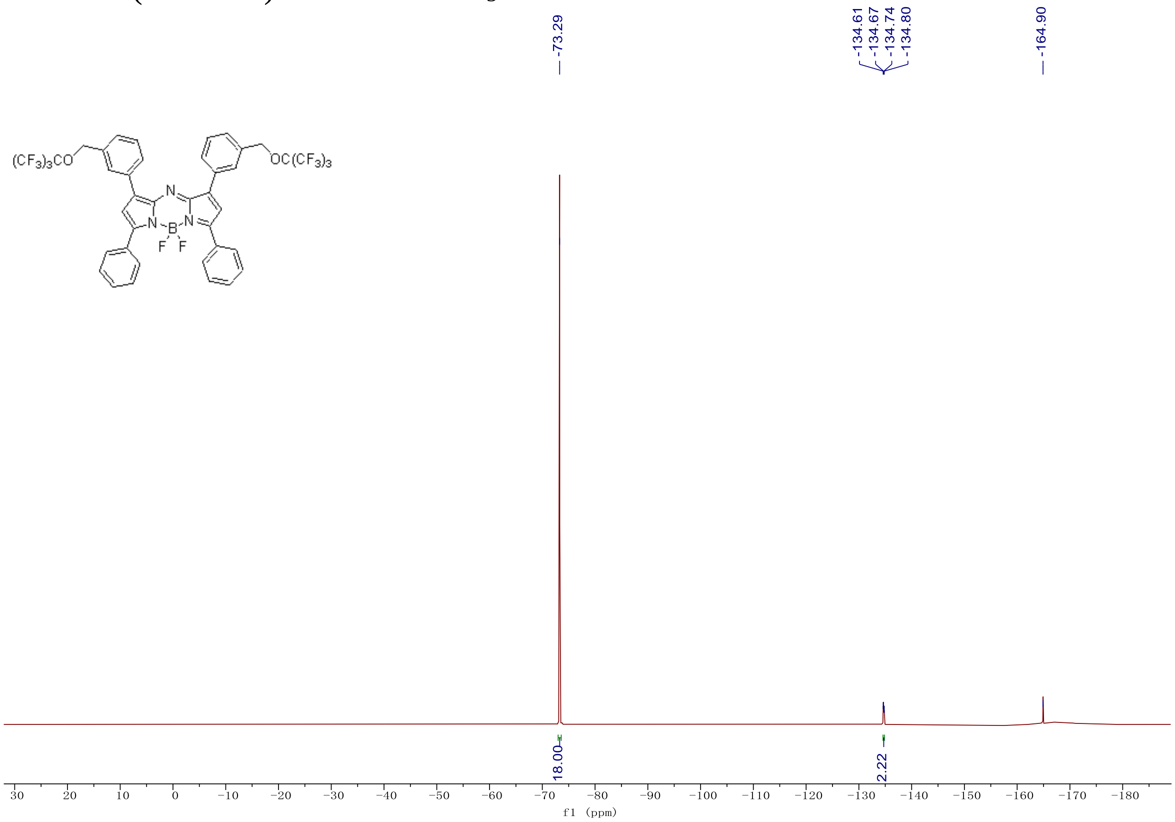

^{13}C NMR (151 MHz) of **PS3** in $CDCl_3$

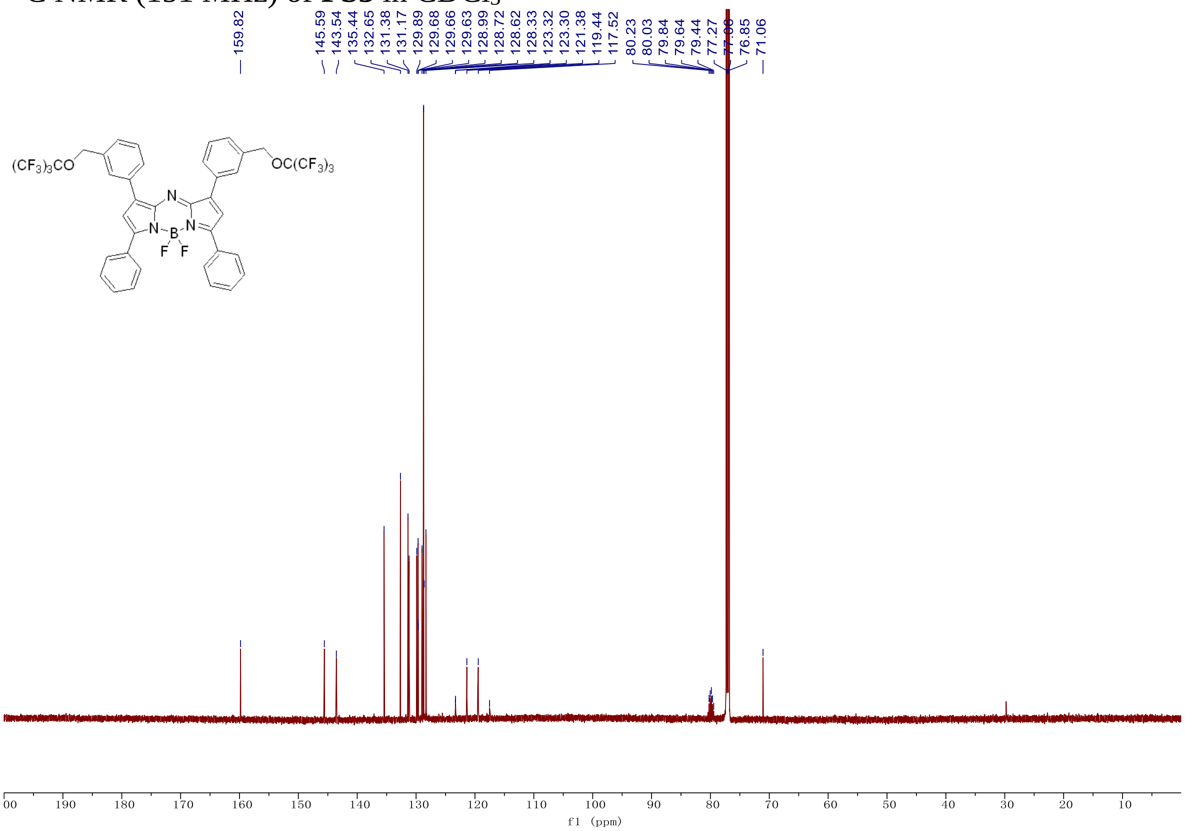

^{1}H NMR (500 MHz) of **PS4** in $CDCl_3$

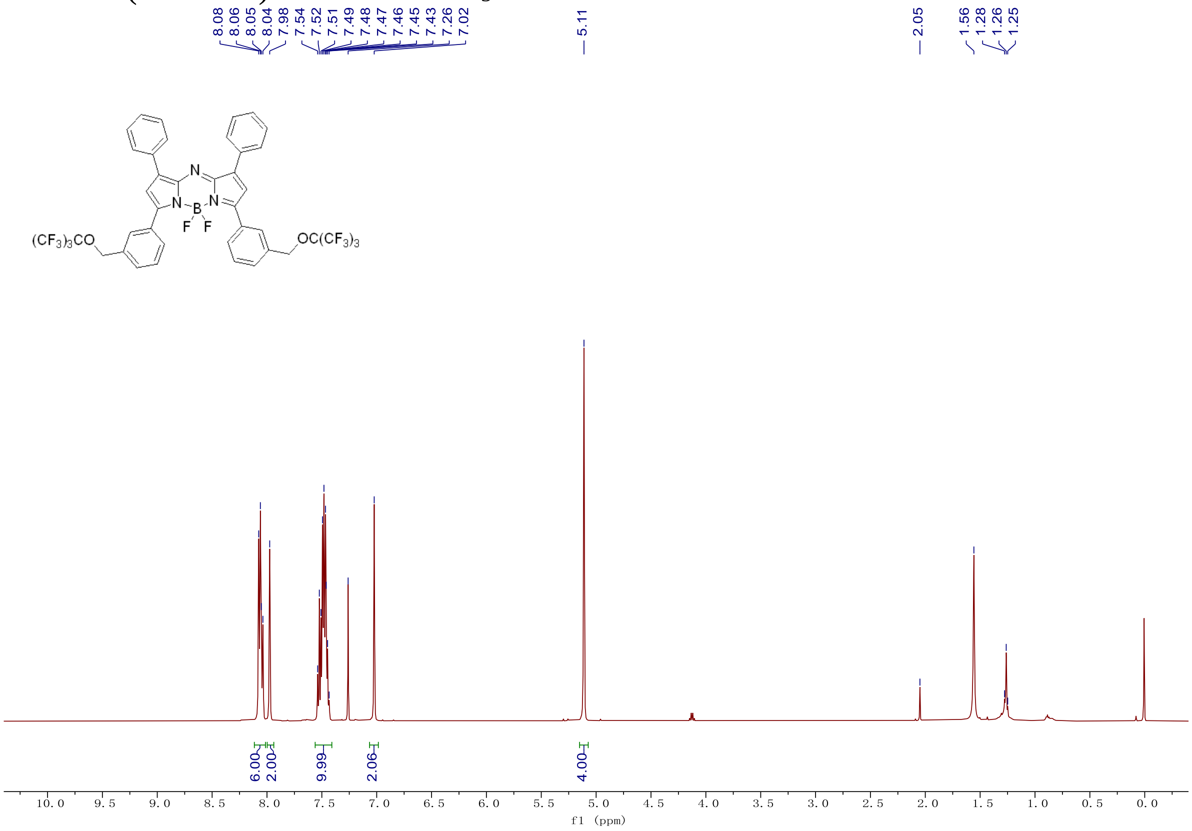

^{19}F NMR (471 MHz) of **PS4** in $CDCl_3$

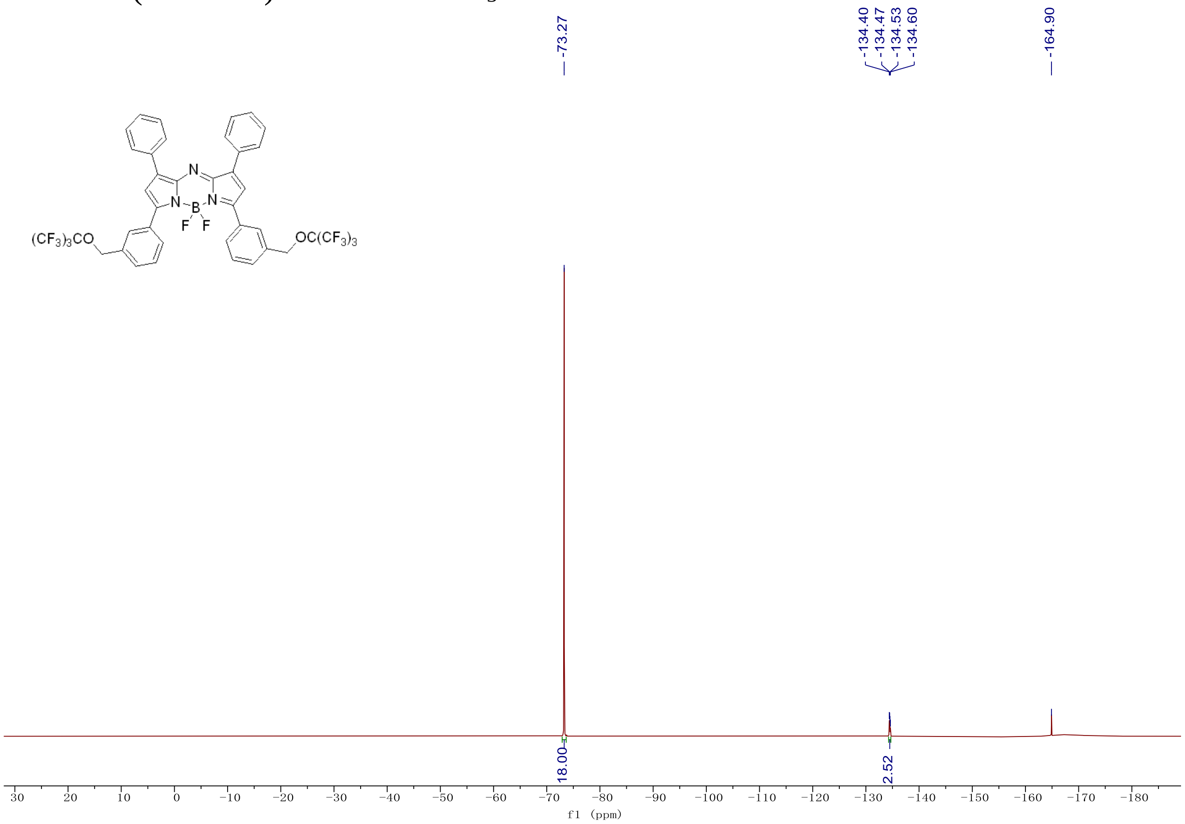

^{13}C NMR (126 MHz) of **PS4** in $CDCl_3$

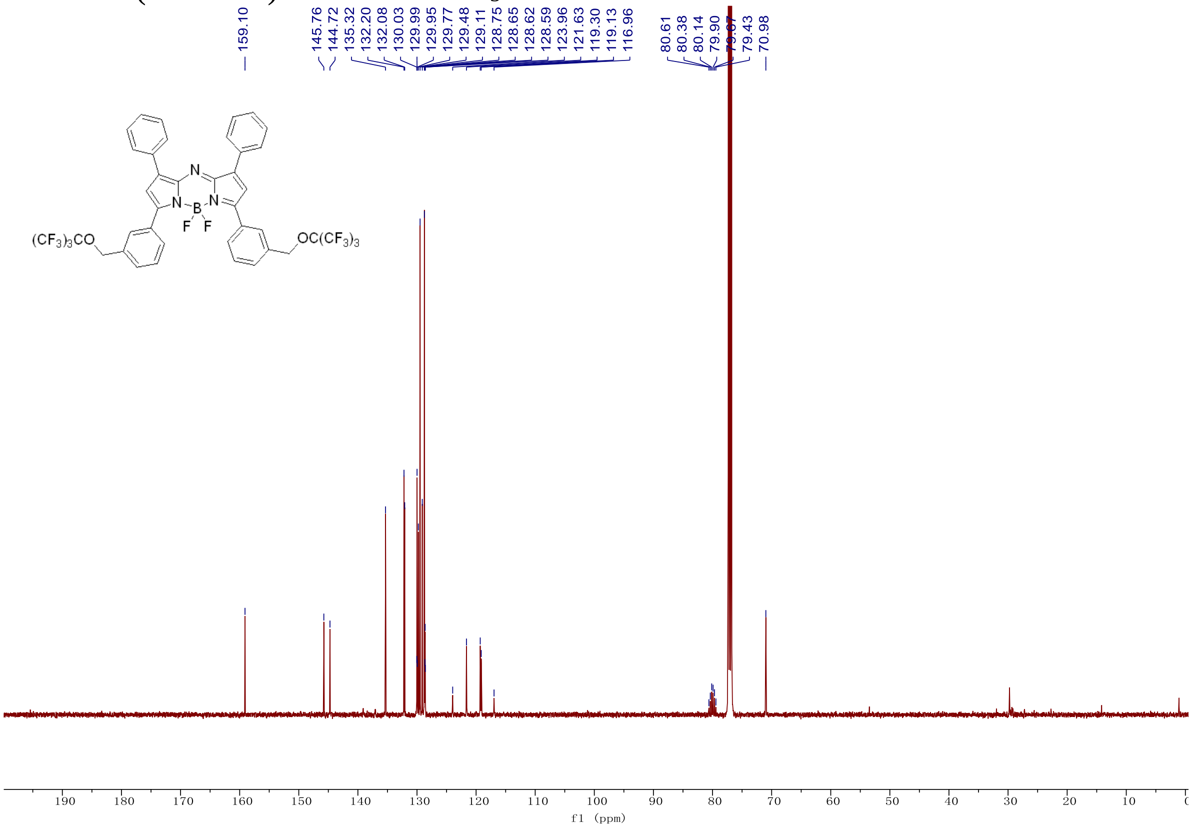

^1H NMR (500 MHz) of **PS5** in $CDCl_3$

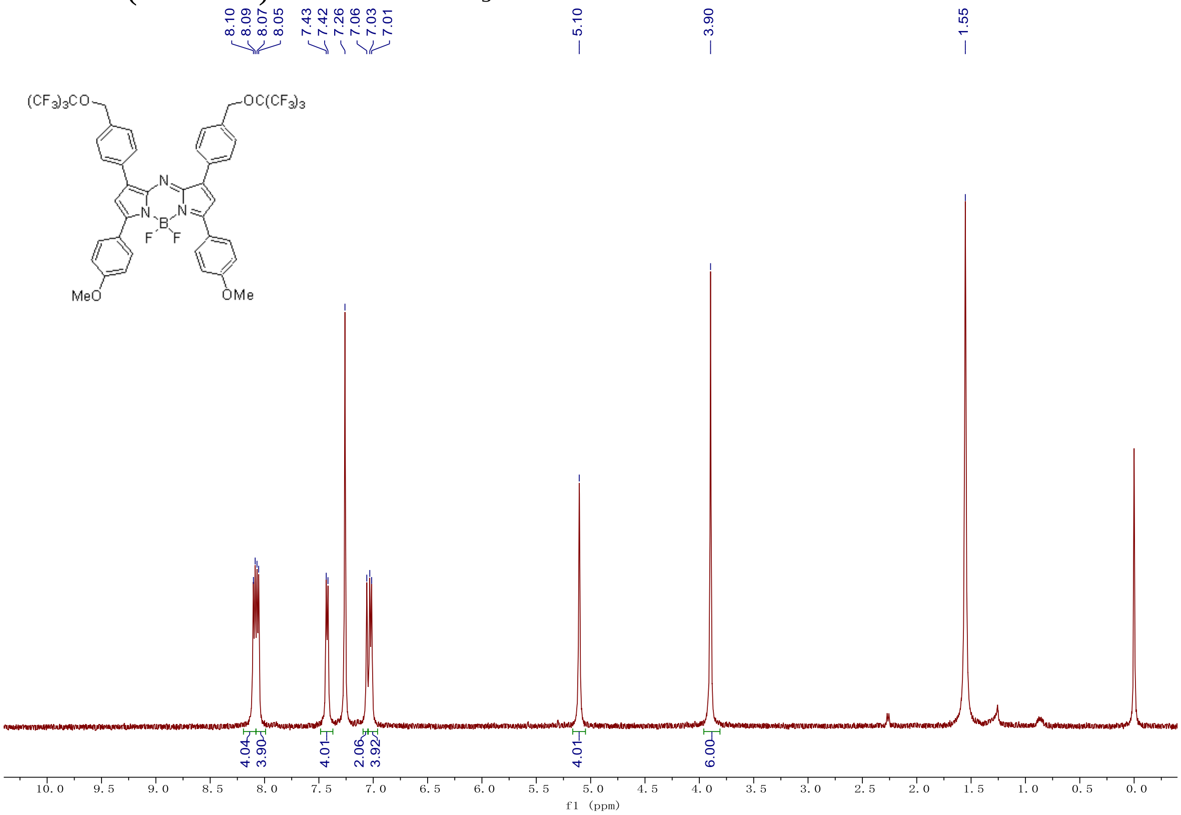

^{19}F NMR (376 MHz) of **PS5** in $CDCl_3$

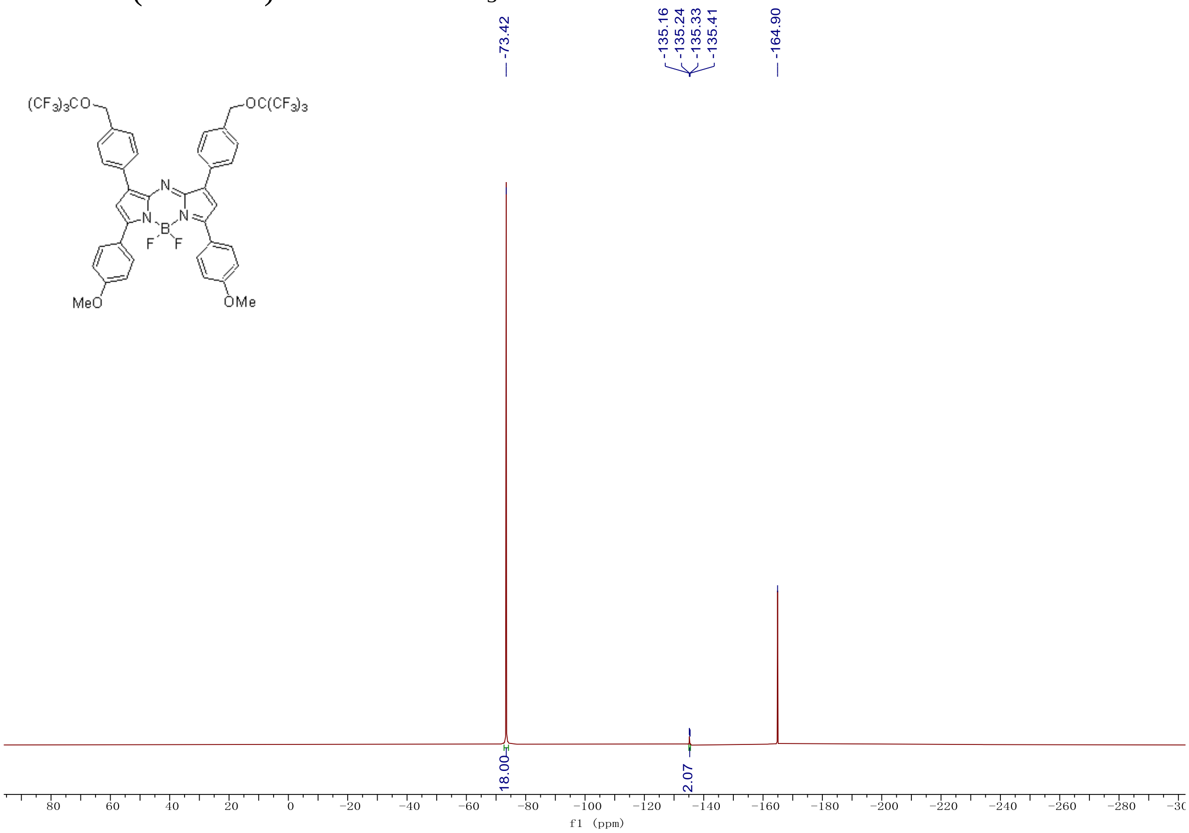

^{13}C NMR (126 MHz) of **PS5** in $CDCl_3$

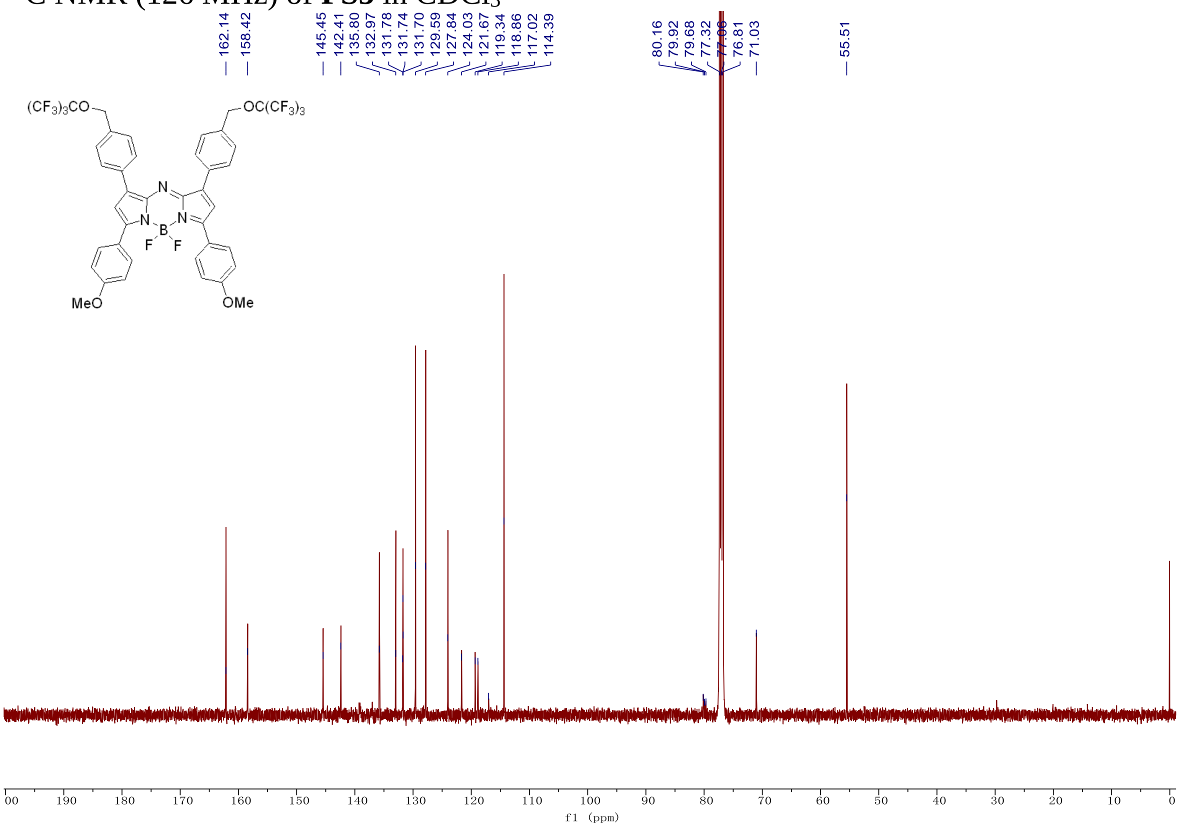

^{1}H NMR (400 MHz) of **PS6** in $CDCl_3$

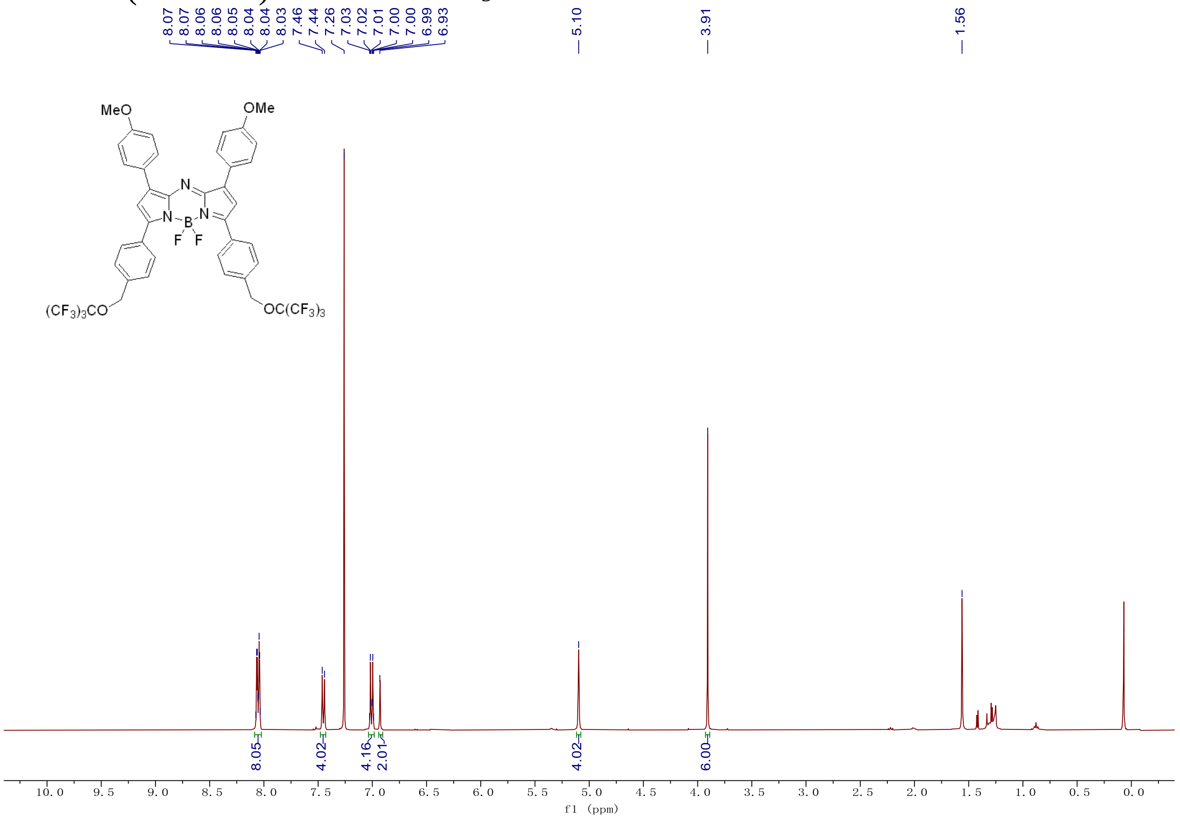

^{19}F NMR (376 MHz) of **PS6** in $CDCl_3$

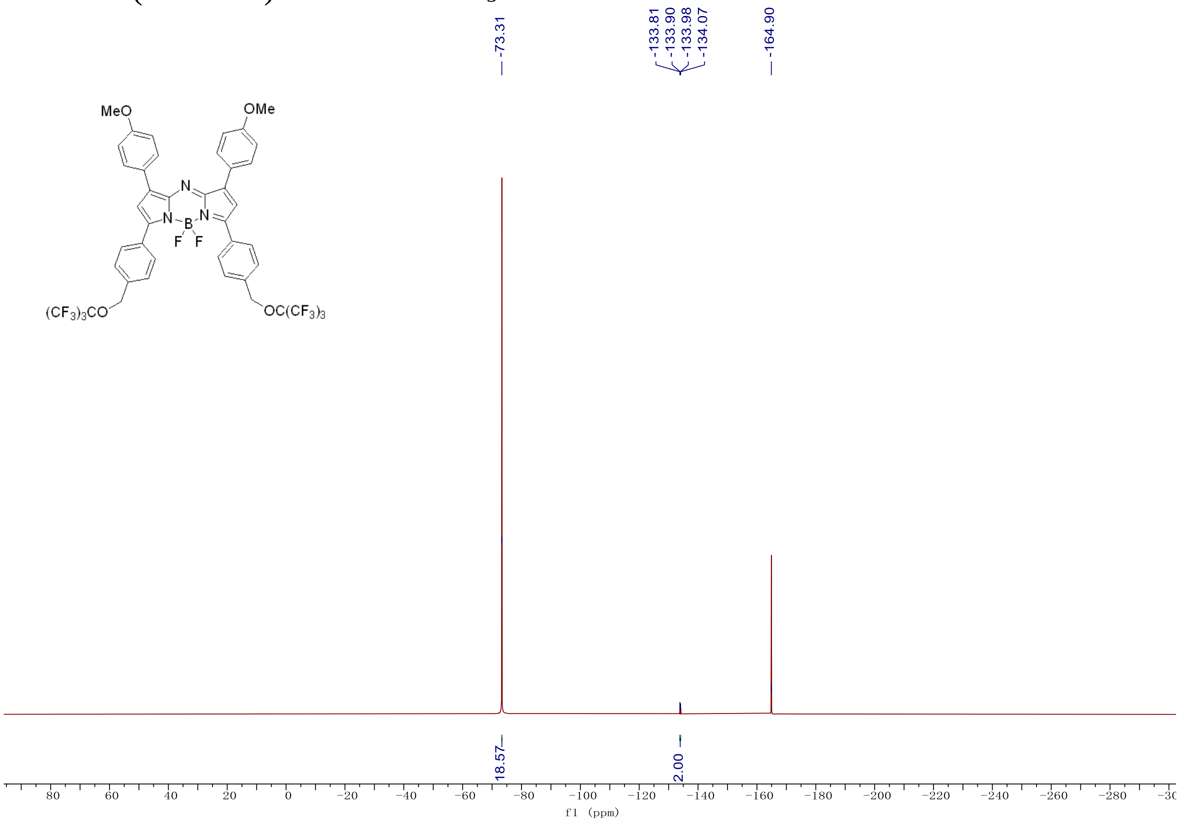

^{13}C NMR (126 MHz) of **PS6** in THF-d_8

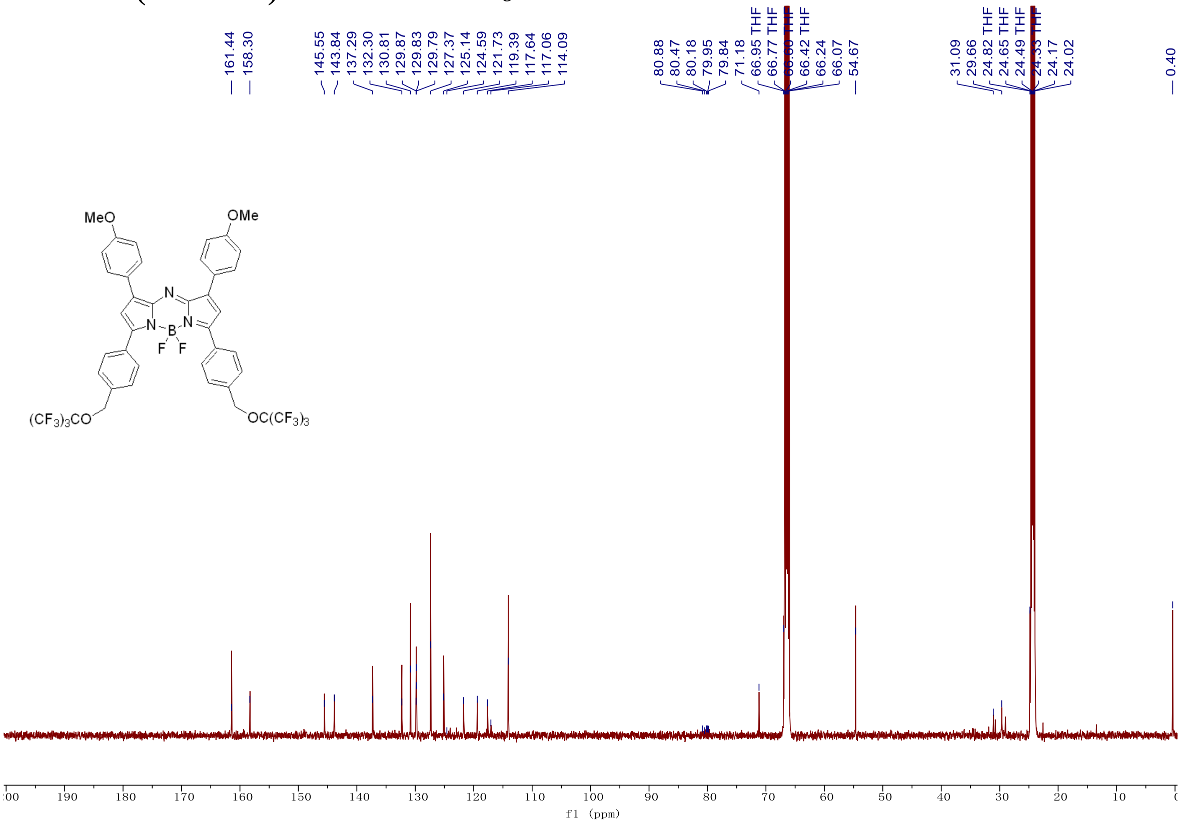

^{1}H NMR (500 MHz) of **PS7** in $CDCl_3$

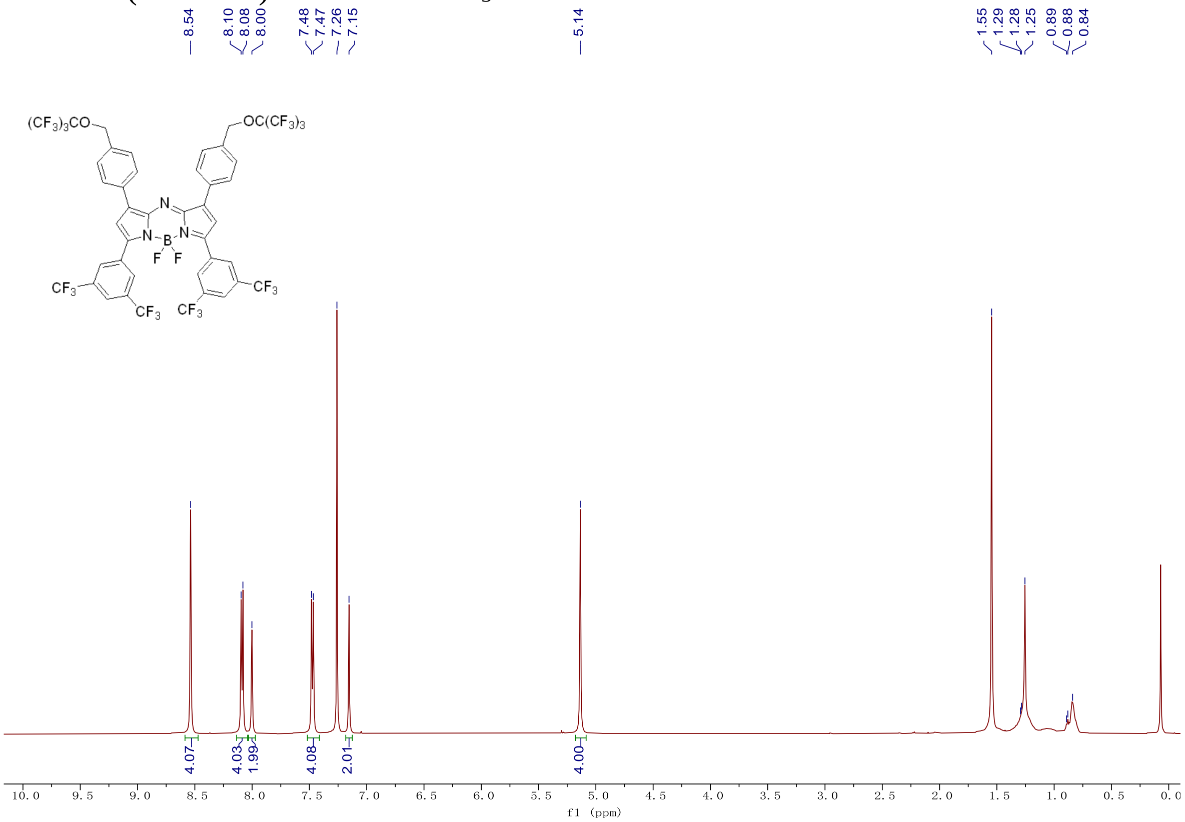

^{19}F NMR (471 MHz) of **PS7** in $CDCl_3$

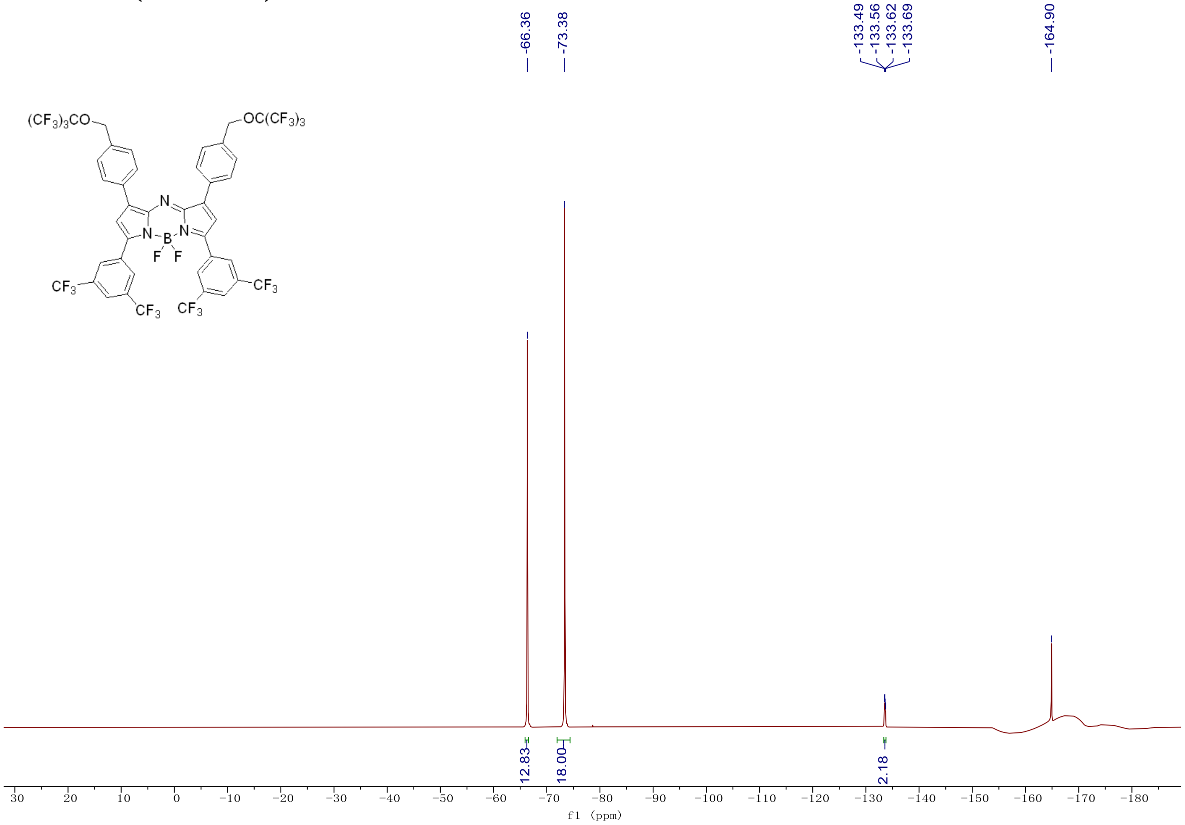

^{13}C NMR (151 MHz) of **PS7** in $CDCl_3$

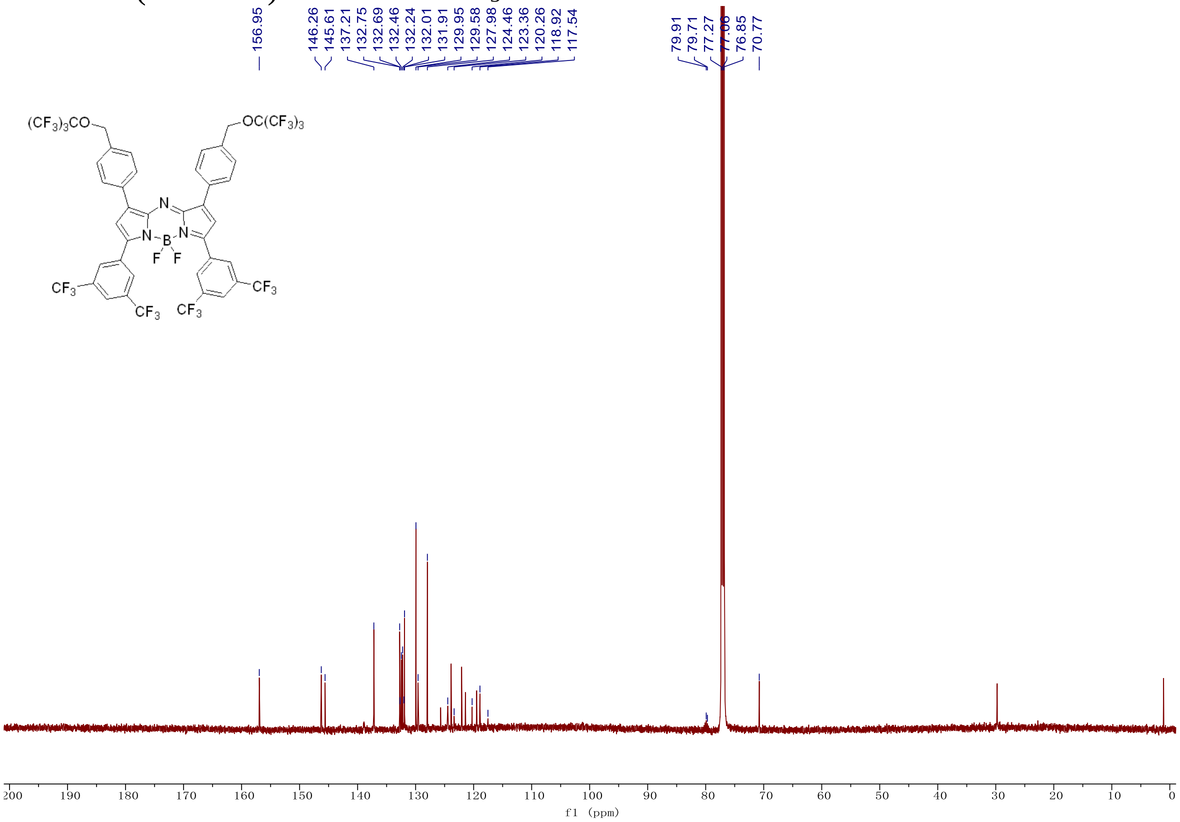

^{1}H NMR (500 MHz) of **PS8** in $CDCl_3$

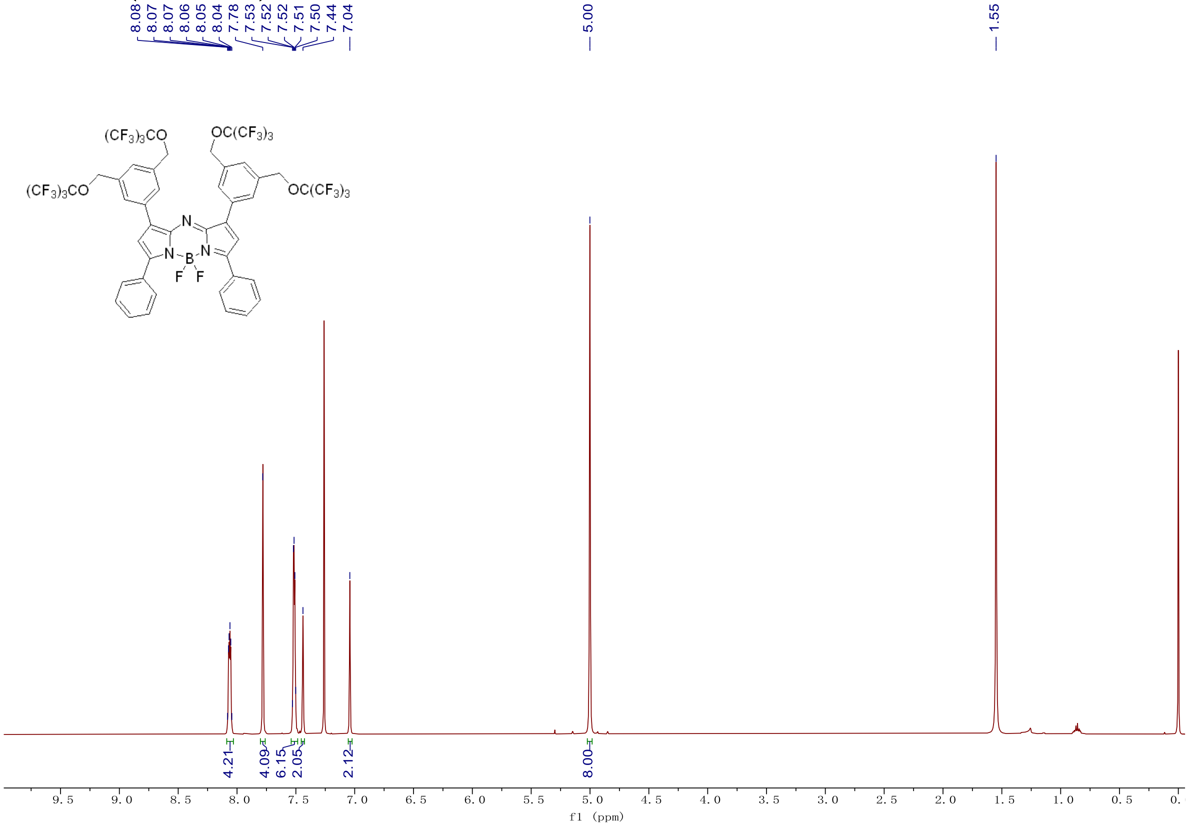

^{19}F NMR (471 MHz) of **PS8** in $CDCl_3$

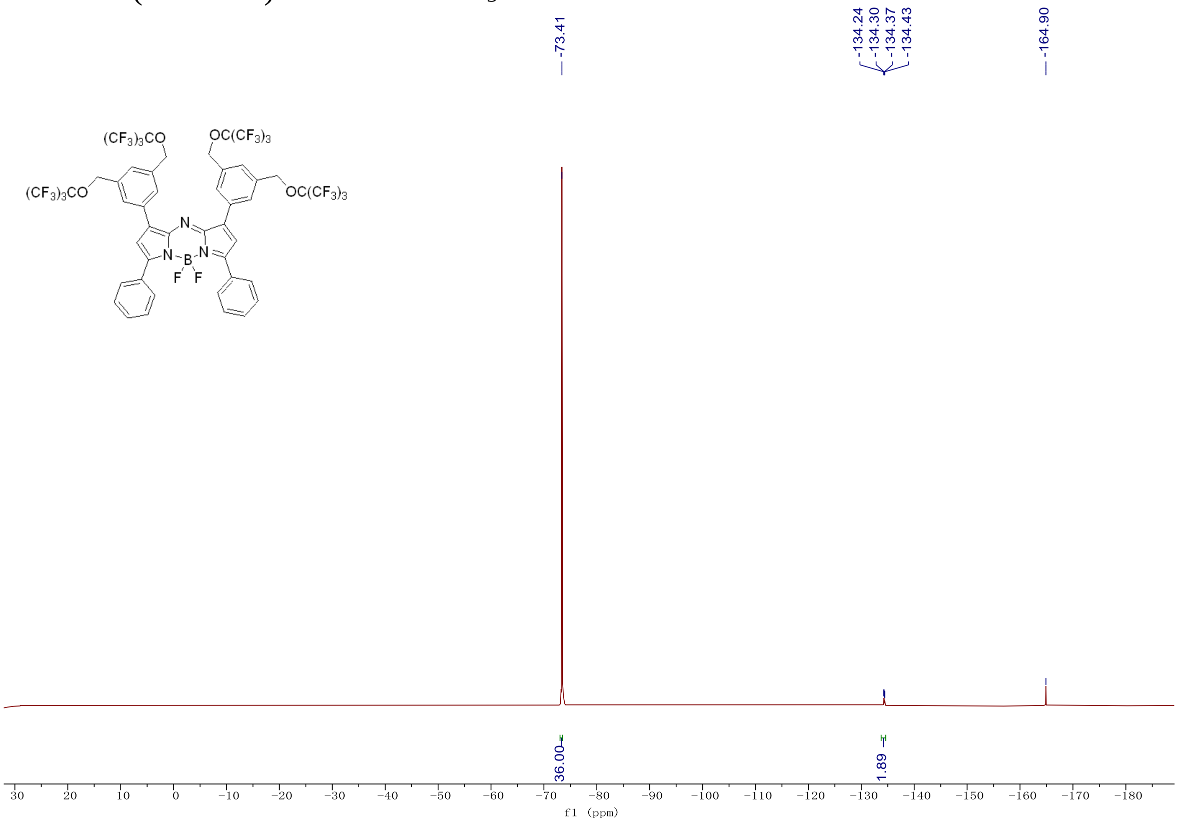

^{13}C NMR (126 MHz) of **PS8** in $CDCl_3$

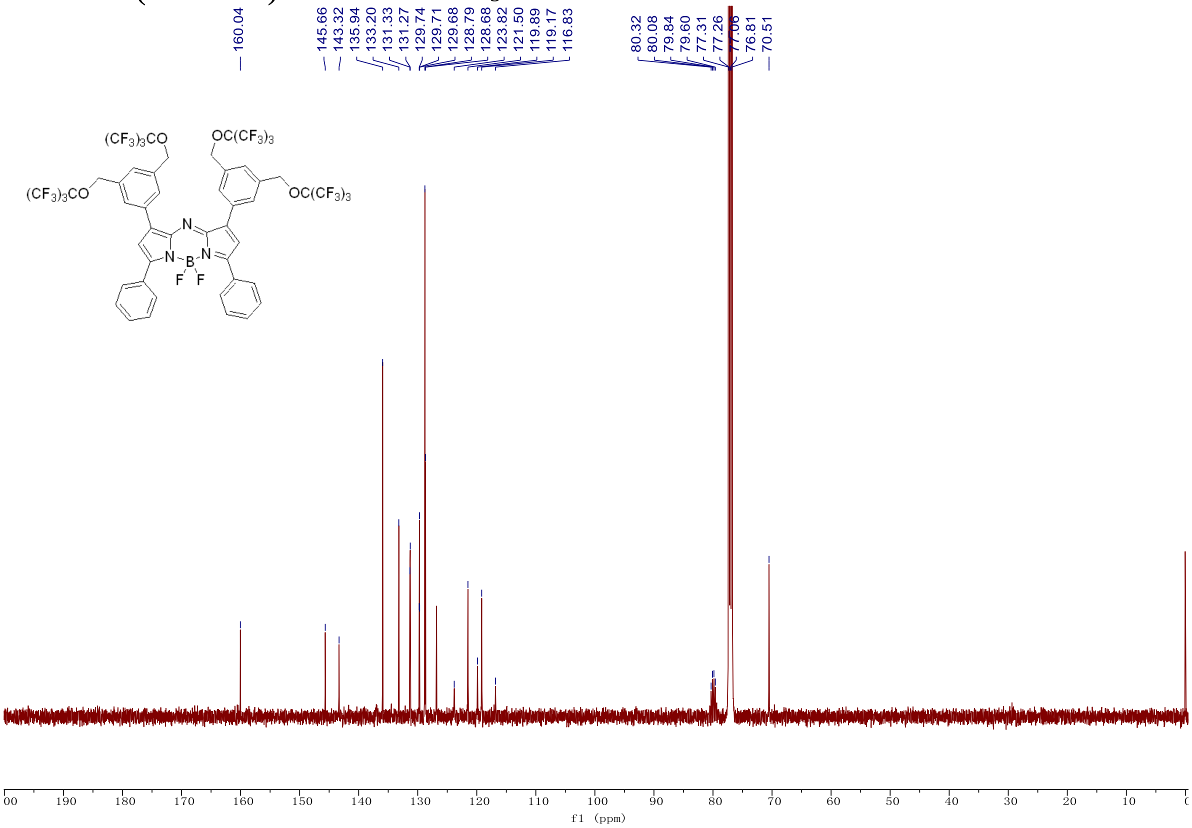

^{1}H NMR (600 MHz) of **PS9** in $CDCl_3$

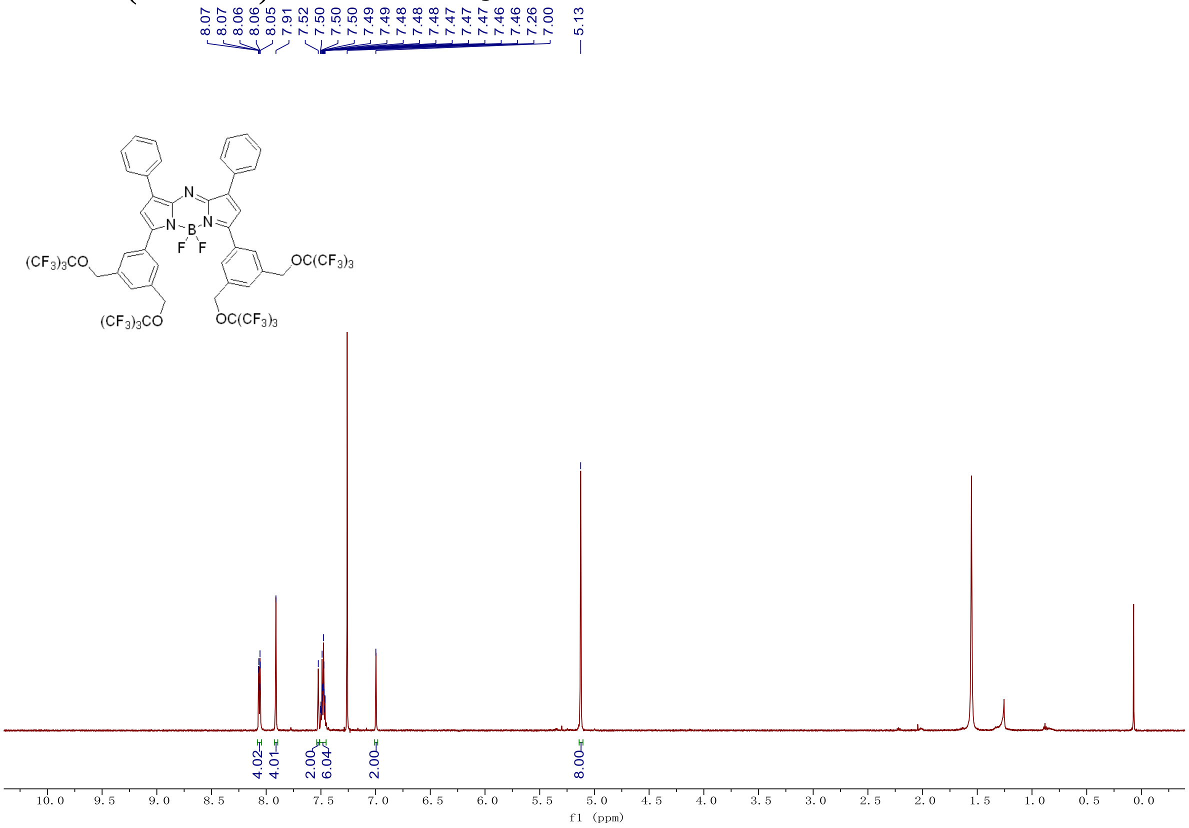

^{19}F NMR (471 MHz) of **PS9** in $CDCl_3$

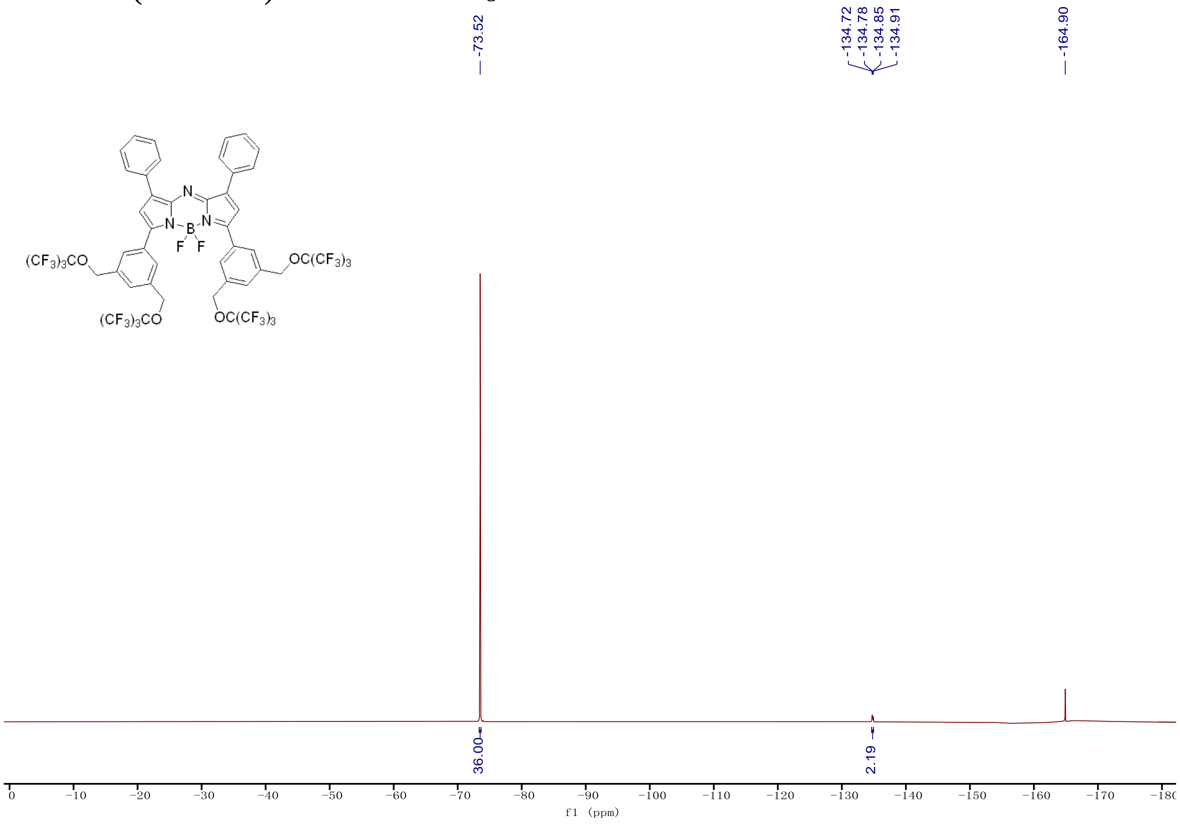

^{13}C NMR (126 MHz) of **PS9** in $CDCl_3$

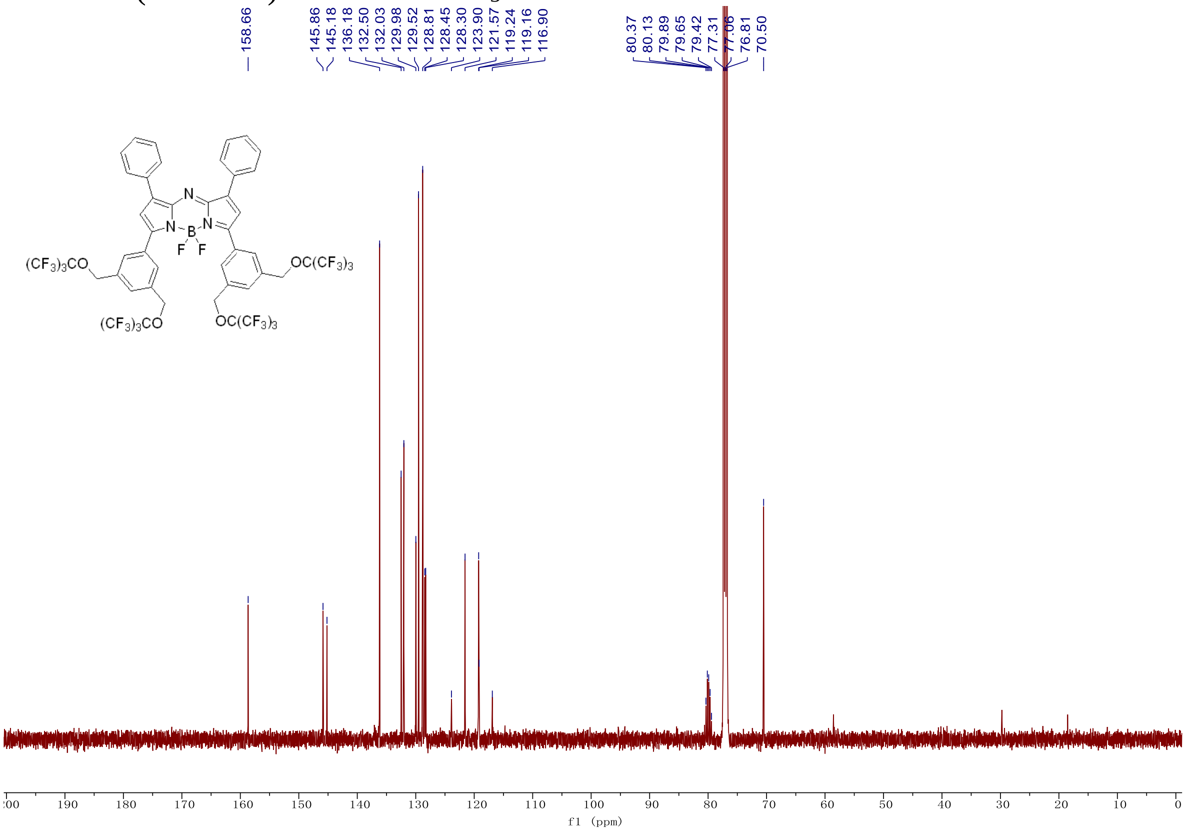

^{1}H NMR (500 MHz) of **PS10** in $CDCl_3$

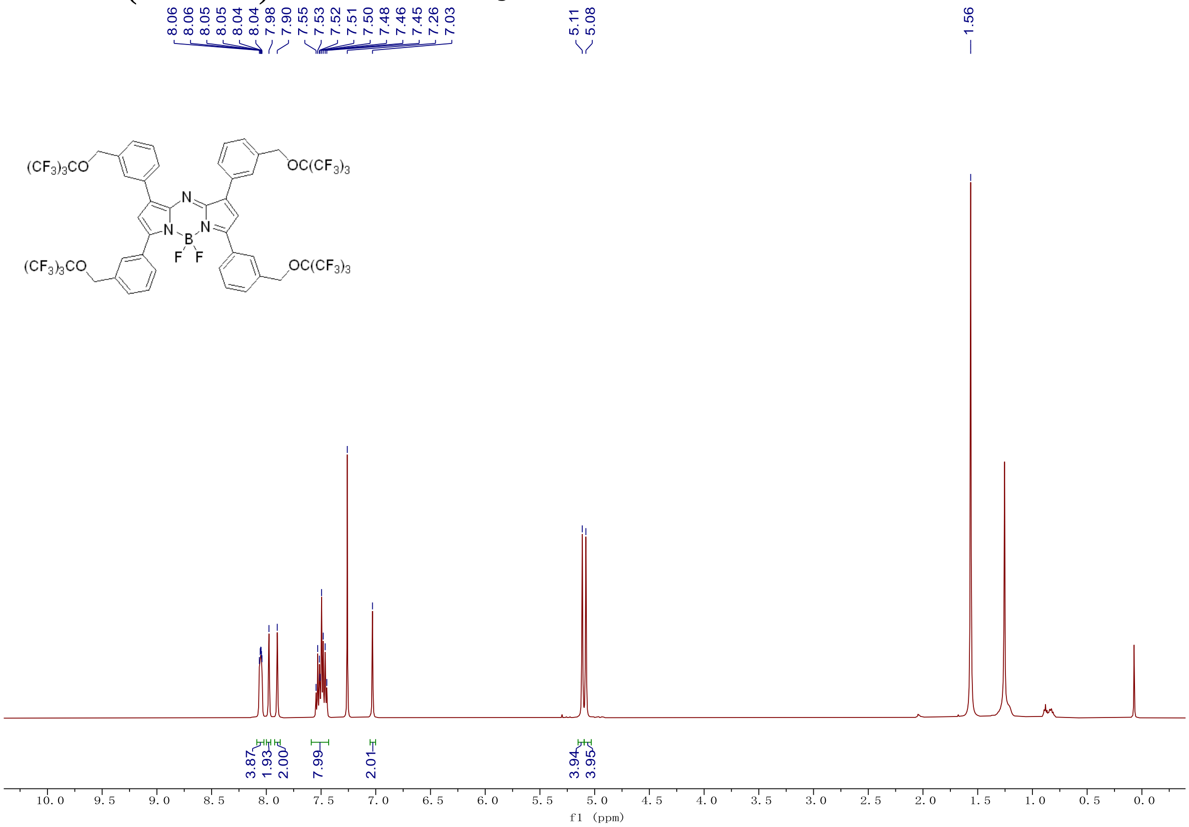

^{19}F NMR (471 MHz) of **PS10** in $CDCl_3$

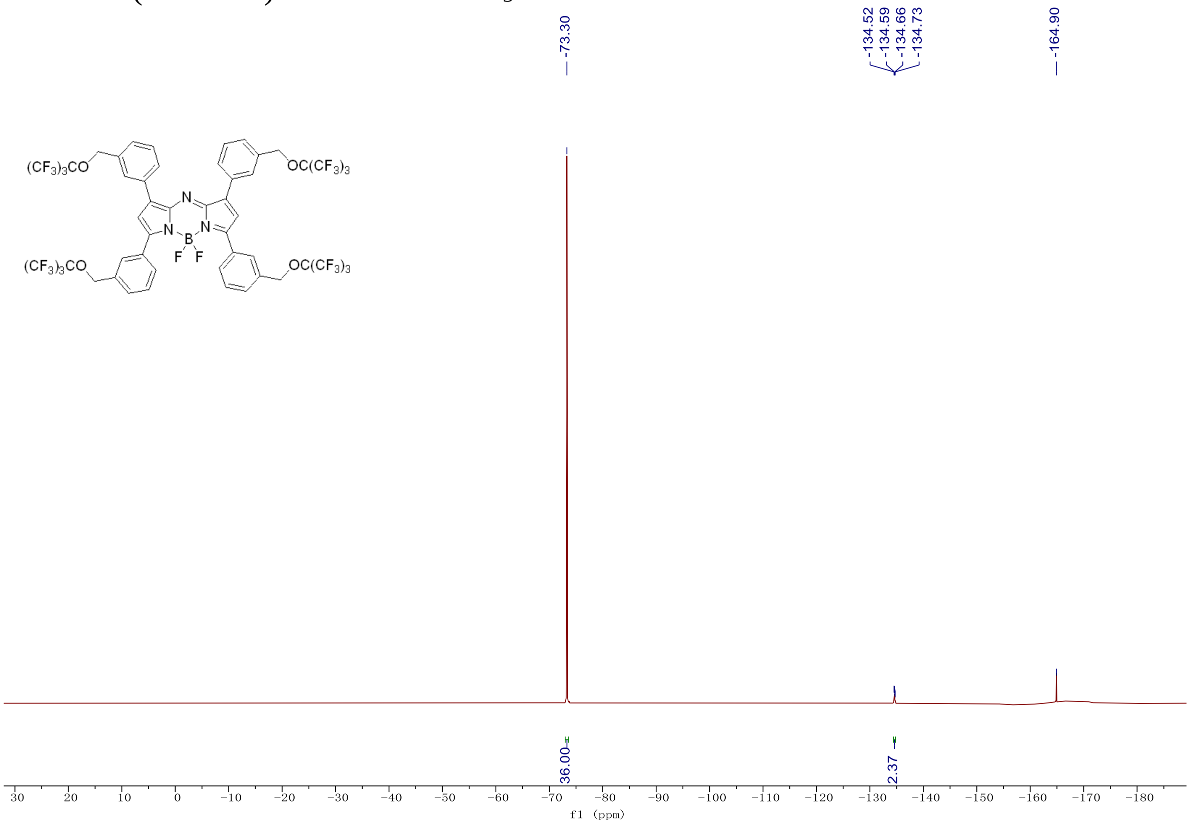

^{13}C NMR (151 MHz) of **PS10** in $CDCl_3$

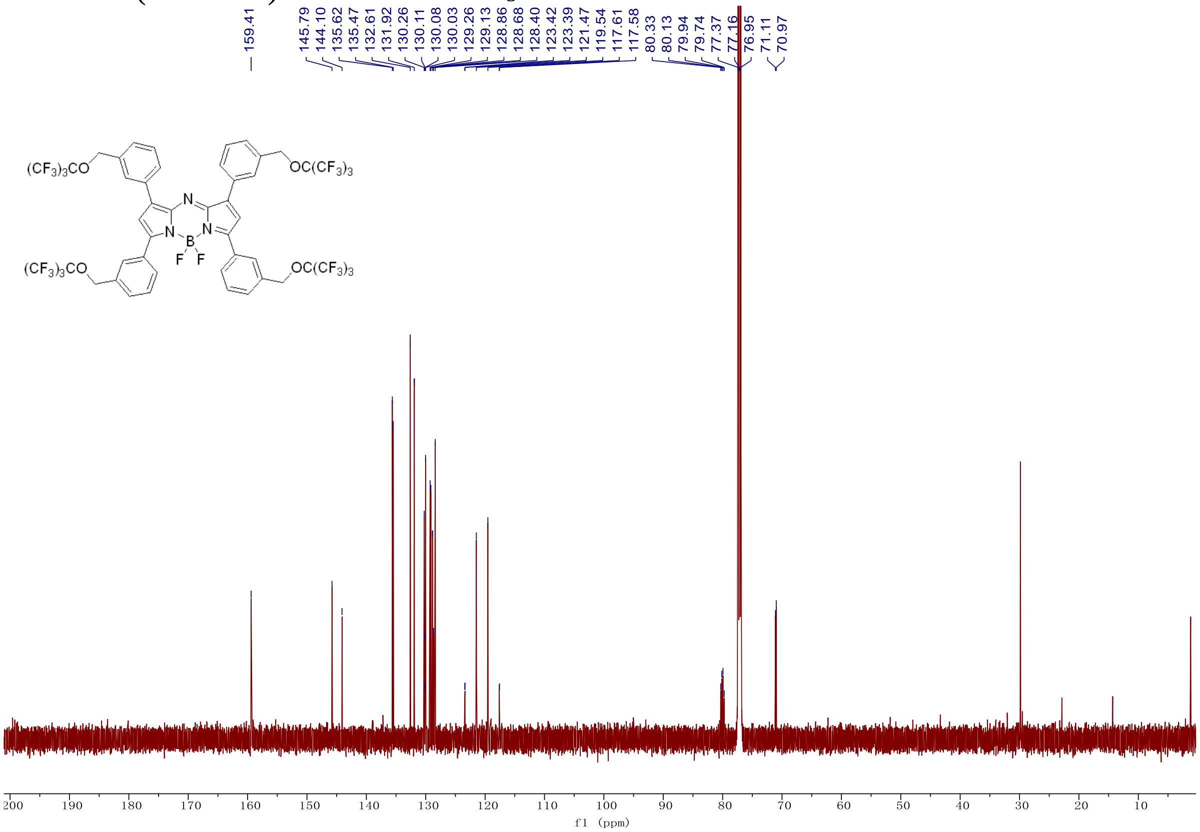

^{1}H NMR (500 MHz) of **11** in $CDCl_3$

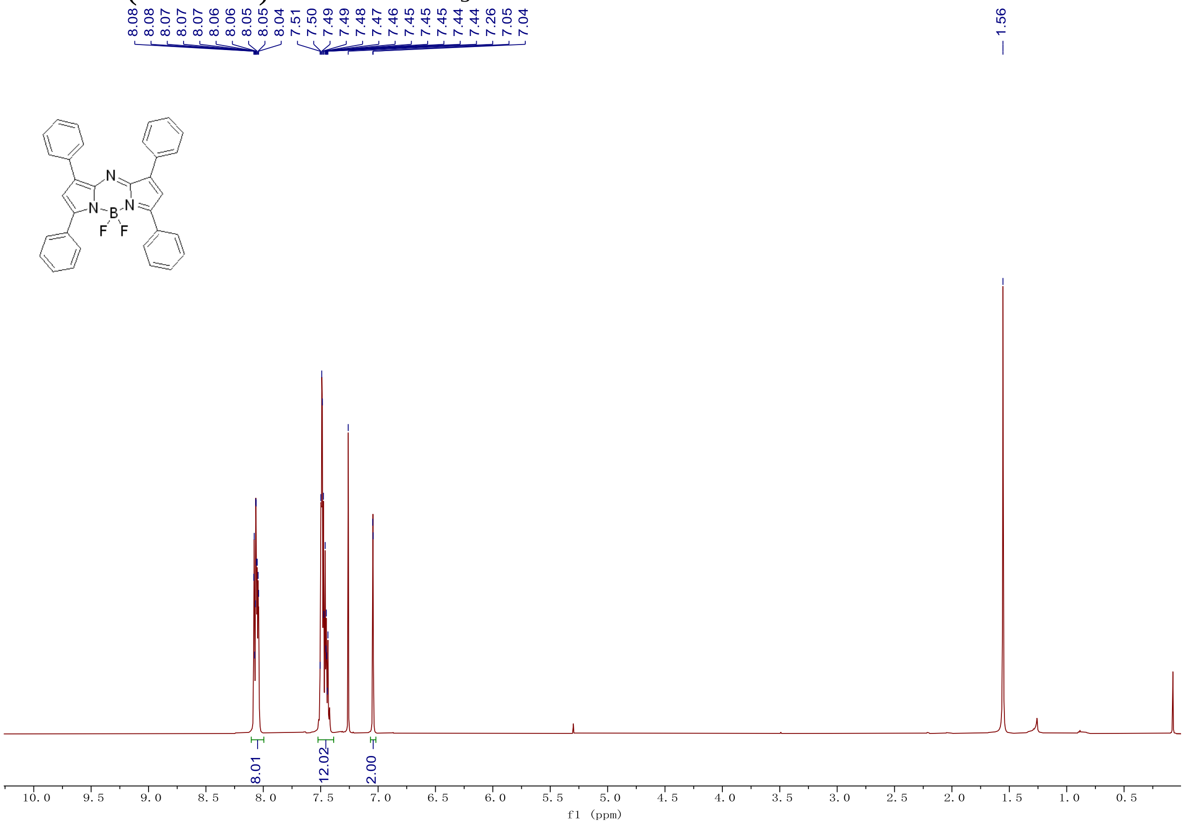

^{1}H NMR (500 MHz) of **12** in $CDCl_3$

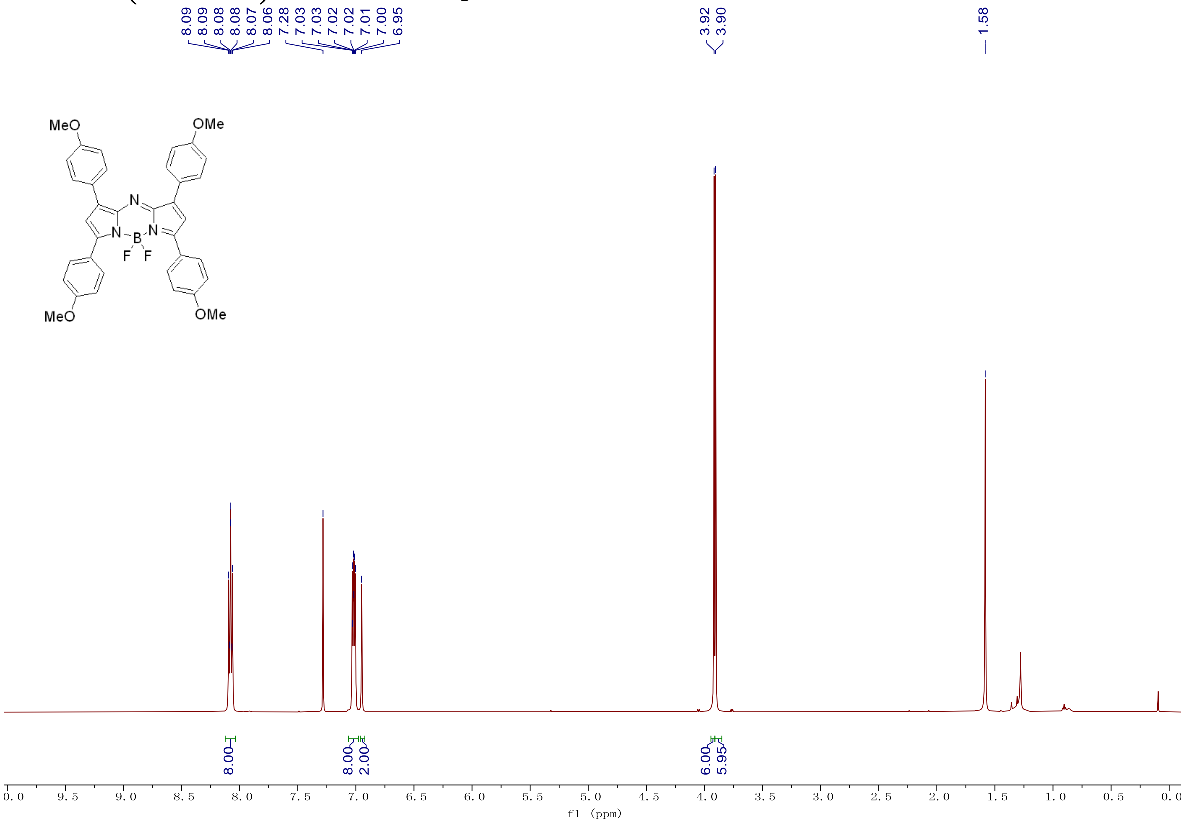

^{1}H NMR (500 MHz) of **13** in $CDCl_3$

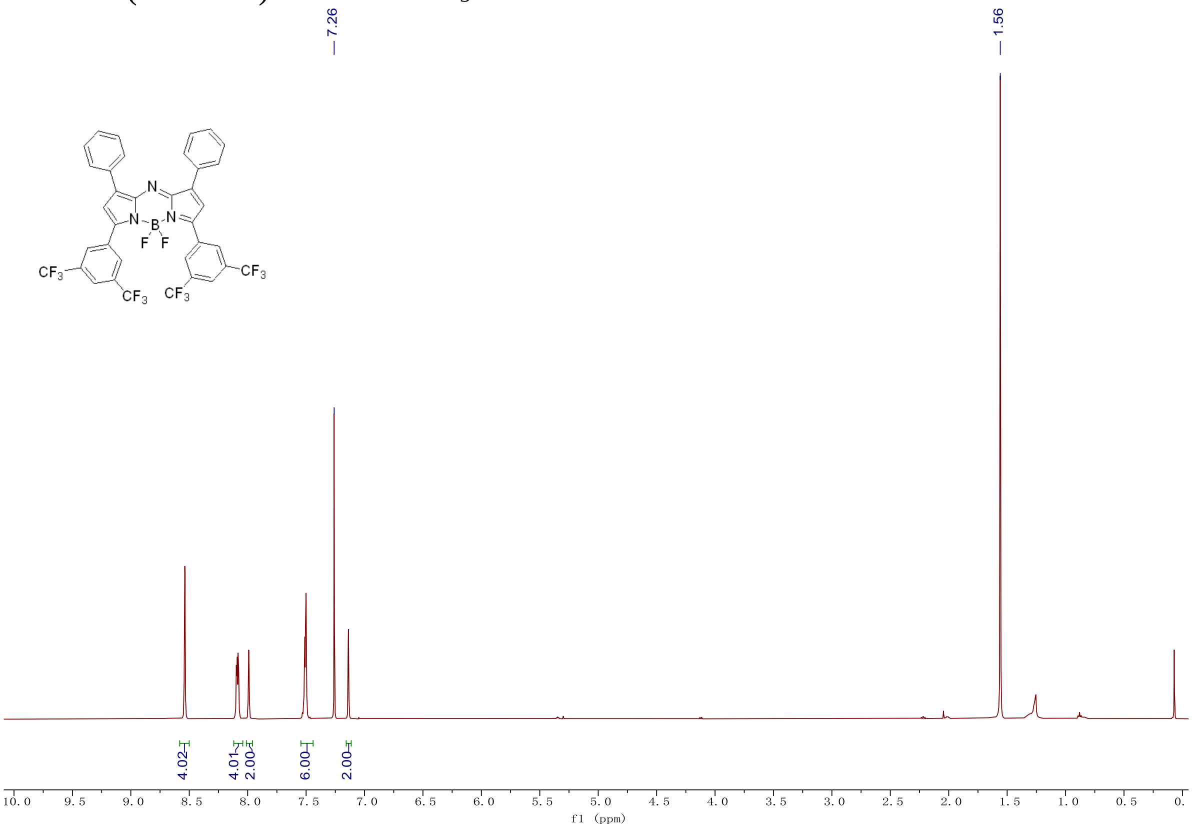

^{1}H NMR (500 MHz) of **F-oil** in $CDCl_3$

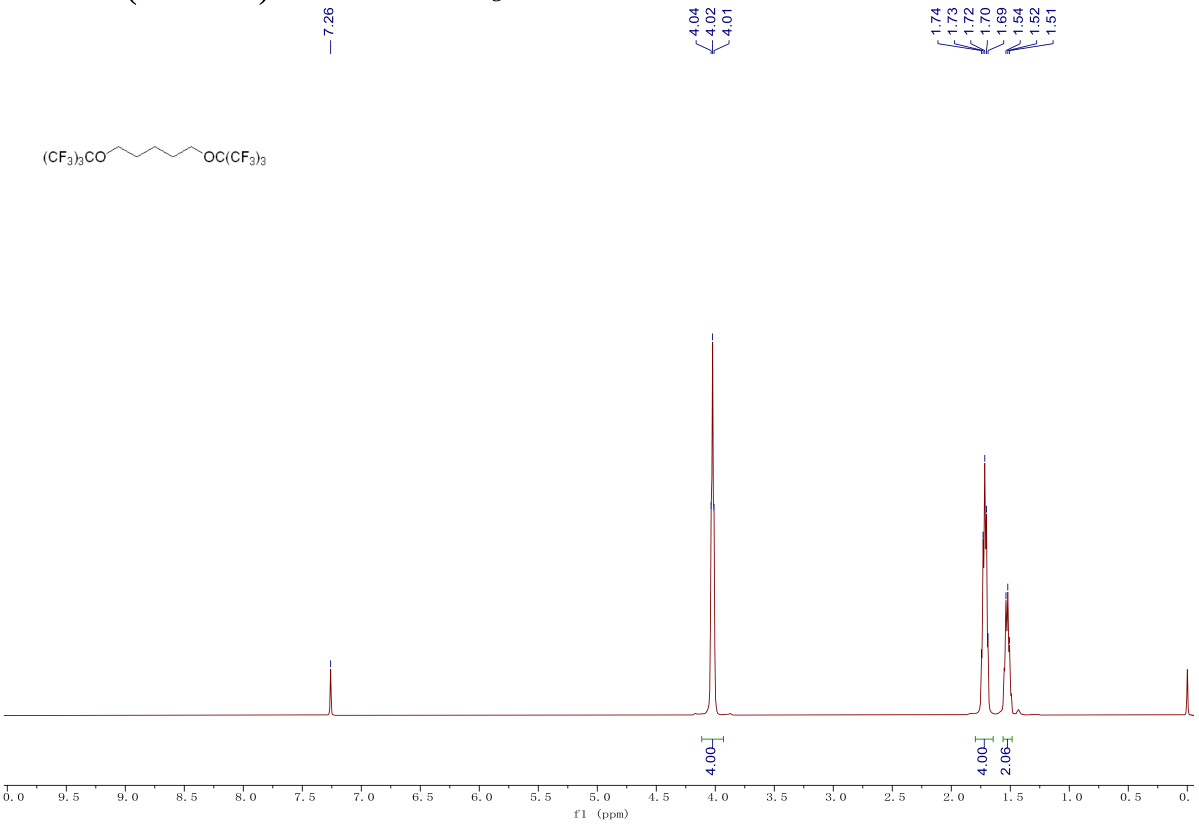

^{19}F NMR (471 MHz) of **F-oil** in $CDCl_3$

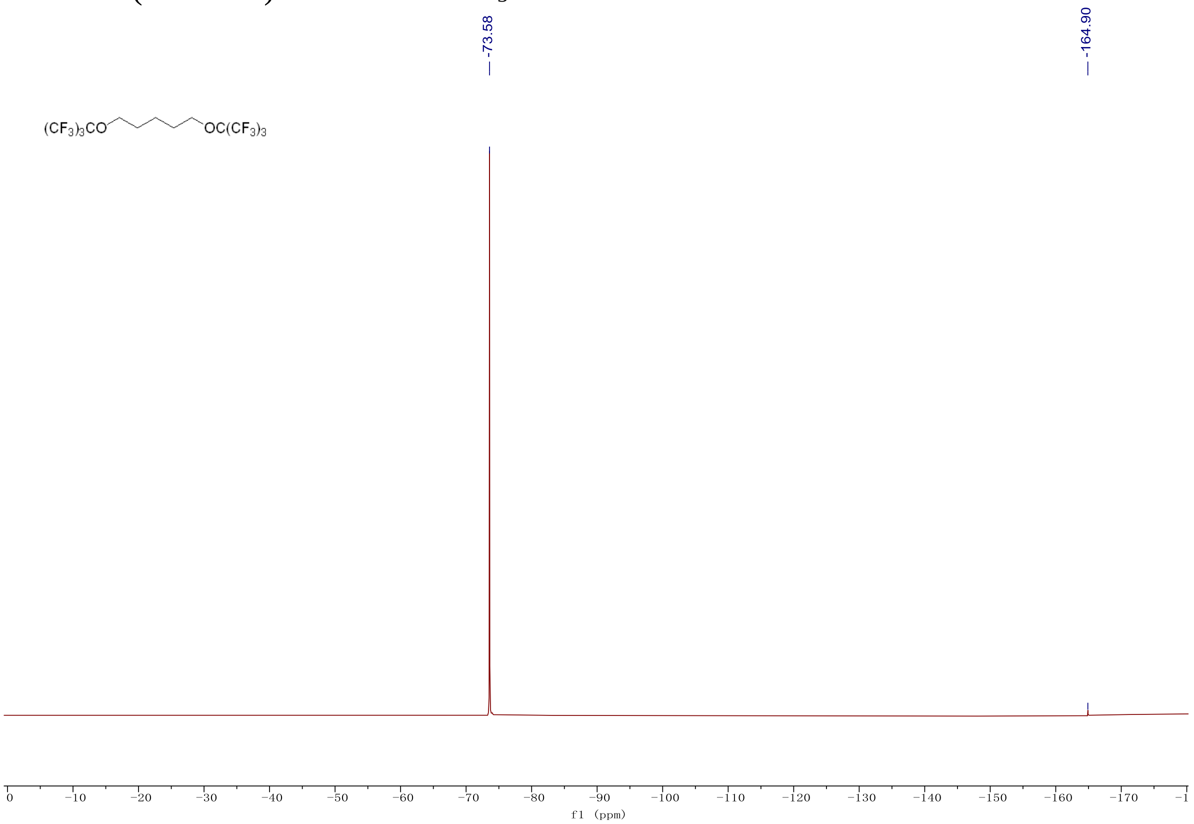

^{13}C NMR (126 MHz) of **F-oil** in $CDCl_3$

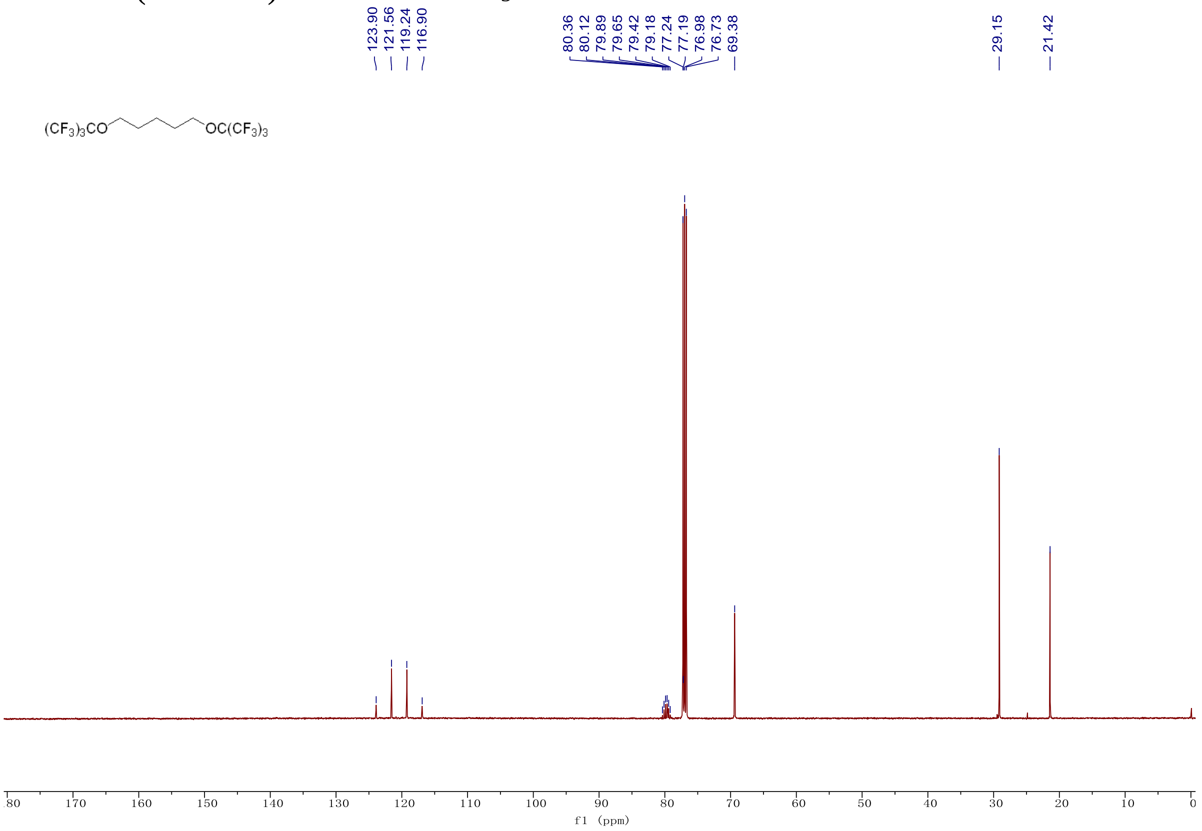

9. References

(1) Killoran, J.; Allen, L.; Gallagher, J. F.; Gallagher, W. M.; O'Shea, D. F. Synthesis of BF2 chelates of tetraarylazadipyrromethenes and evidence for their photodynamic therapeutic behaviour. *Chem. Commun*. **2002**, *7*, 1862-1863.

(2) Wang, J.; Wu, Y.; Sheng, W.; Yu, C.; Wei, Y.; Hao, E.; Jiao, L. Synthesis, structure, and properties of beta-Vinyl ketone/ester functionalized azaBODIPYs from formylazaBODIPYs. *ACS Omega* **2017**, *2*, 2568-2576.

(3) Helman, W. P.; Ross, A. B. Quantum yields for the photosensitized formation of the lowest electronically excited singlet state of molecular oxygen in solution. *J. Phys. Chem. Ref. Data* **1993**, *22*, 113-262.

(4) Adarsh, N.; Avirah, R. R.; Ramaiah, D. Tuning photosensitized singlet oxygen generation efficiency of novel aza-BODIPY dyes. *Org. Lett*. **2010**, *12*, 5720-5723.
